# Supplementary material for: Partial Pulpotomy in Young Permanent Teeth: A Systematic Review and Meta-Analysis
Source: Children (Basel). 2023 Aug 24;10(9):1447. doi: 10.3390/children10091447 (PMC10527738; doi:10.3390/children10091447)
Supplement: Supplementary file 1 [file children-10-01447-s001.zip › Table S2.pdf]

Table S2. Search string.

Pubmed	Vital pulp therapy [tiab] OR pulpotomy [mh] OR partial pulpotomy [tiab] OR coronal pulpotomy [tiab] OR pulp therapy [tiab] OR direct pulp therapy [tiab] OR pulp cavity [tiab] OR reversible pulpitis [tiab] OR irreversible pulpitis [tiab] OR pulpitis[tiab] OR minimal endodontic treatment [tiab] OR pulp treatment [tiab] OR pulp diagnosis [tiab] OR pulp exposure [tiab] OR pulp cavity[tiab] OR pulp survival [tiab] OR pulp symptoms [tiab]<br>AND<br>dental caries [mh] OR dental [tiab] AND caries [tiab]OR dental pulp cavity [mh] OR dental [tiab] AND pulp [tiab] AND cavity [tiab]) OR dental caries[mh] OR ICDAS [tiab] OR first molar [tiab] OR young permanent tooth [tiab] OR young permanent molar [tiab] OR deep caries [tiab]<br>Filters: results by year 2012-2022
Embase	(((('pulpotomy'/exp OR 'partial pulpotomy' OR (('partial' NEAR/3 'pulpotomy'):ab,ti) OR 'endodontic procedure' OR 'coronal pulpotomy' OR 'minimal endodontic procedure' OR 'pulp therapy' OR direct) AND pulp AND therapy OR reversible) AND pulpitis OR irreversible) AND pulpitis OR vital) AND pulp AND therapy OR 'endodontic procedure'/exp<br>AND<br>'dental caries'/exp OR 'dental caries' OR 'dental decay'/exp OR 'dental decay' OR ('dental' NEAR/3 'decay') OR 'mandibular first molar'/exp OR 'maxillary firs molar' OR 'mih' OR 'molar incisor hypomineralization' OR 'icdas'<br>Filters: results by year 2012-2022 and [article]/lim
Scopus	TITLE-ABS-KEY (((("vital pulp therapy" OR "partial pulpotomy" OR "pulpotomy" OR "coronal pulpotomy" OR "pulp therapy" OR "direct pulp therapy" OR "reversible pulpitis" OR "irreversible pulpitis" OR "pulpitis" OR "minimal endodontic treatment" OR (("therapy") W/7 (pulp* OR endo*)))) AND (("caries" OR "dental caries" OR "icdas" OR "first permanent molar" OR ("dental" W/7 (caries OR pulp*))))))<br>Filters: results by year 2012-2022 and DOCTYPE , "ar"
