# Supplementary material for: Partial Pulpotomy in Young Permanent Teeth: A Systematic Review and Meta-Analysis
Source: Children (Basel). 2023 Aug 24;10(9):1447. doi: 10.3390/children10091447 (PMC10527738; doi:10.3390/children10091447)
Supplement: Supplementary file 1 [file children-10-01447-s001.zip › Table S3.pdf]

Table S3. Extraction form.

Author	Sources	Location	Foundig sources	Type of study	Age	Patients (n)	Teeth (n)	Drop-out of teeth	Study design	Final restoration	Follow-up	Outcomes	Overall Succes rate test group	Overall Succes rate control group	Conclusion
