# Supplementary material for: Partial Pulpotomy in Young Permanent Teeth: A Systematic Review and Meta-Analysis
Source: Children (Basel). 2023 Aug 24;10(9):1447. doi: 10.3390/children10091447 (PMC10527738; doi:10.3390/children10091447)
Supplement: Supplementary file 1 [file children-10-01447-s001.zip › Table S4.pdf]

Table S4. Papers discarded after title and abstract evaluation.

Author Names	Title	Year	Journal
Reddy S.; Prakash V.; Subbiya A.; Mitthra S.	100 years of calcium hydroxide in dentistry: A review of literature	2020	Indian Journal of Forensic Medicine and Toxicology
Höhne C, Schmitter M.	3D Printed Teeth for the Preclinical Education of Dental Students	2019	J Dent Educ
Hong S.; Park Y.-H.; Lee J.; Moon J.; Kong E.; Jeon J.; Park J.-C.; Kim H.-R.; Kim P.	3d visualization of dynamic cellular reaction of pulpal cd11c+ dendritic cells against pulpitis in whole murine tooth	2021	International Journal of Molecular Sciences
Chen W.; Guan Y.; Xu F.; Jiang B.	4-Methylumbelliferone promotes the migration and odontogenetic differentiation of human dental pulp stem cells exposed to lipopolysaccharide in vitro	2021	Cell Biology International
Chen Y.-P., Hsieh C.-Y., Hsu W.-T., Wu F.-Y., Shih W.-Y.	A 10-year trend of dental treatments under general anesthesia of children in Taipei Veterans General Hospital	2017	Journal of the Chinese Medical Association
Cleary J, Al-Hadidi R, Scully A, Yahn W, Zaid Z, Boynton JR, Eckert GJ, Yanca E, Fontana M.	A 12-Month Randomized Clinical Trial of 38% SDF vs. Restorative Treatment	2022	JDR Clin Trans Res
Chen K.; Lei Q.; Xiong H.; Chen Y.; Luo W.; Liang Y.	A 2-year clinical evaluation of stainless steel crowns and composite resin restorations in primary molars under general anaesthesia in China's Guangdong province	2018	British Dental Journal
Gu Y, Xie X, Zhuang R, Weir MD, Oates TW, Bai Y, Zhao L, Xu HHK.	A Biphasic Calcium Phosphate Cement Enhances Dentin Regeneration by Dental Pulp Stem Cells and Promotes Macrophages M2 Phenotype In Vitro	2021	Tissue Eng Part A
Escobar PM, Kishen A, Lopes FC, Borges CC, Kegler EG, Sousa-Neto MD.	A CAD/CAM-based strategy for concurrent endodontic and restorative treatment	2019	Restor Dent Endod
Kunisada Y., Yoshioka N., Ibaragi S., Okui T., Nagatsuka H., Sasaki A.	A case of intramandibular neurofibroma resembling a radicular cyst in a neurofibromatosis type 1 patient	2021	International Journal of Surgery Case Reports
Arigbede A.O.; Adesuwa A.A.	A case of quackery and obsession for diastema resulting in avoidable endodontic therapy	2012	African Health Sciences
Torabinejad M.; Faras H.	A clinical and histological report of a tooth with an open apex treated with regenerative endodontics using platelet-rich plasma	2012	Journal of Endodontics
Munoz-Sanchez M.-L., Linas N., Decerle N., Nicolas E., Hennequin M., Cousson P.-Y.	A combination of full pulpotomy and chairside cad/cam endocrown to treat teeth with deep carious lesions and pulpitis in a single session: A preliminary study	2020	International Journal of Environmental Research and Public Health
Alsaikhan L.S.; Algarni R.A.; Alzahrani M.A.; Gufran K.; Alqahtani A.M.; Altammami M.; Mansy I.	A comparative analysis of periapical status by using cone beam computed tomography and periapical radiography	2022	European Review for Medical and Pharmacological Sciences
Winnier J.; Suresh R.	A comparative evaluation of a labial approach with a conventional palatal approach for endodontic access in primary maxillary incisors: A pilot study	2020	International Journal of Clinical Pediatric Dentistry
Selvi M.M.; Selvabalaji A.; Chonat A.; Ananthan V.; Mani E.; Chinnaswamy A.D.	A comparative evaluation of antimicrobial efficacy of triphala and calcium hydroxide as intracanal medicament: An in Vitro study	2022	Journal of Pharmacy and Bioallied Sciences
Kathal S.; Gupta S.; Bhayya D.P.; Rao A.; Roy A.P.; Sabhlok A.	A comparative evaluation of clinical and radiographic success rate of pulpotomy in primary molars using antioxidant mix and mineral trioxide aggregate: An in vivo 1-year follow-up study	2017	Journal of Indian Society of Pedodontics and Preventive Dentistry
Karthik E.V.G.; Pradeep S.; Ganapathy D.	A comparative evaluation of efficacy of apex locators vs radiovisiograph in determining the working length of single rooted teeth-an in-vitro study	2021	International Journal of Dentistry and Oral Science
Jose J.A.; Somaiah S.; Muddaiah S.; Shetty B.; Reddy G.; Roopa S.	A comparative evaluation of interleukin 1 beta and prostaglandin E2 with and without low-level laser therapy during en masse retraction	2018	Contemporary Clinical Dentistry

Mankeliya S.; Singhal R.K.; Gupta A.; Jaiswal N.; Pathak V.K.; Kushwah A.	A Comparative Evaluation of Smear Layer Removal by Using Four Different Irrigation Solutions like Root Canal Irrigants: An In Vitro SEM Study	2021	Journal of Contemporary Dental Practice
Jain P.; Yeluri R.; Garg N.; Mayall S.; Rallan M.; Gupta S.; Pathivada L.	A comparative evaluation of the effectiveness of three different irrigating solution on microorganisms in the root canal: An invivo study	2015	Journal of Clinical and Diagnostic Research
Chandrasekhar S., Prasad M.G., Radhakrishna A.N., Saujanya K., Raviteja N.V.K., Deepthi B., Ramakrishna J.	A comparative In vivo efficacy of three spiral techniques versus incremental technique in obturating primary teeth	2018	Journal of the Indian Society of Pedodontics and Preventive Dentistry
Haghanifar S, Moudi E, Mesgarani A, Bijani A, Abbaszadeh N.	A comparative study of cone-beam computed tomography and digital periapical radiography in detecting mandibular molars root perforations	2014	Imaging Sci Dent
Paul M.P., Amin S., Mayya A., Naik R., Mayya S.S.	A comparative study of surface hardness between two bioceramic materials in an experimental apexification model under wet and dry conditions	2020	Open Dentistry Journal
Moghadam K.N.; Aghili H.; Mohassel A.R.; Zahedpasha S.; Moghadamnia A.A.	A comparative study on sealing ability of mineral trioxide aggregate, calcium enriched cement and bone cement in forcai perforations	2014	Minerva Stomatologica
Marconyak L.J., Jr.; Kirkpatrick T.C.; Roberts H.W.; Roberts M.D.; Aparicio A.; Himel V.T.; Sabey K.A.	A comparison of coronal tooth discoloration elicited by various endodontic reparative materials	2016	Journal of Endodontics
Bastawala D.S.; Kapoor S.; Nathani P.	A comparison of coronal tooth discoloration elicited by various endodontic reparative materials MTA plus, bio MTA+, and biodentine: An ex vivo study	2020	International Journal of Clinical Pediatric Dentistry
Abazarpoor R, Parirokh M, Nakhaee N, Abbott PV.	A Comparison of Different Volumes of Articaine for Inferior Alveolar Nerve Block for Molar Teeth with Symptomatic Irreversible Pulpitis	2015	J Endod
Tootla S.; Owen C.P.	A comparison of endodontic treatment outcomes between HIV-positive and HIV-negative patients.	2012	SADJ : journal of the South African Dental Association = tydskrif van die Suid-Afrikaanse Tandheelkundige Vereniging
Kanaa M.D.; Whitworth J.M.; Meechan J.G.	A comparison of the efficacy of 4% articaine with 1:100,000 epinephrine and 2% lidocaine with 1:80,000 epinephrine in achieving pulpal anesthesia in maxillary teeth with irreversible pulpitis	2012	Journal of Endodontics
Bueno MR, Estrela C.	A computational modeling method for root canal endoscopy using a specific CBCT filter: A new era in the metaverse of endodontics begins	2022	Braz Dent J
Katge F.; Dixit U.B.	A cone-beam computed tomographic study of root and root canal morphology of primary maxillary and mandibular second molars in Indian Children: An in vitro study	2022	Journal of Indian Society of Pedodontics and Preventive Dentistry
Rankin R.; Lundy F.T.; Schock B.C.; Zhang S.-D.; Al-Natour B.; About I.; Irwin C.; Linden G.J.; El-Karim I.A.	A connectivity mapping approach predicted acetylsalicylic acid (aspirin) to induce osteo/odontogenic differentiation of dental pulp cells	2020	International Endodontic Journal
Richert R, Ducret M, Alliot-Licht B, Bekhouche M, Gobert S, Farges JC.	A critical analysis of research methods and experimental models to study pulpitis	2022	Int Endod J
Zehnder M, Belibasakis GN.	A critical analysis of research methods to study clinical molecular biomarkers in Endodontic research	2022	Int Endod J
Timmerman A., Calache H., Parashos P.	A cross sectional and longitudinal study of endodontic and periapical status in an Australian population	2017	Australian dental journal
Lee AE, Chu EY, Gardner PJ, Duverger O, Saikali A, Wang SK, Gafni RI, Hartley IR, Ten Hagen KG, Somerman MJ, Collins MT.	A Cross-Sectional Cohort Study of the Effects of FGF23 Deficiency and Hyperphosphatemia on Dental Structures in Hyperphosphatemic Familial Tumoral Calcinosis	2021	JBMR Plus
Fukai K., Ohno H., Blinkhorn A.	A cross-sectional survey investigating care of the primary dentition by paediatric dental specialists in Japan and the UK	2012	International Dental Journal
Lopes DS, Câmara AC, Aguiar CM, do Nascimento MD, Farias de Araújo L.	A C-Shaped Canal in a Maxillary Second Molar: Prexion 3D Cone-Beam Computed Tomography Analysis	2016	Acta Stomatol Croat

Li J.; Rao Z.; Zhao Y.; Xu Y.; Chen L.; Shen Z.; Bai Y.; Lin Z.; Huang Q.	A Decellularized Matrix Hydrogel Derived from Human Dental Pulp Promotes Dental Pulp Stem Cell Proliferation, Migration, and Induced Multidirectional Differentiation In Vitro	2020	Journal of Endodontics
Hiraiwa T.; Ariji Y.; Fukuda M.; Kise Y.; Nakata K.; Katsumata A.; Fujita H.; Ariji E.	A deep-learning artificial intelligence system for assessment of root morphology of the mandibular first molar on panoramic radiography	2019	Dentomaxillofacial Radiology
Li W.; Mao M.; Hu N.; Wang J.; Huang J.; Zhang W.; Gu S.	A graphene oxide-copper nanocomposite for the regeneration of the dentin-pulp complex: An odontogenic and neurovascularization-inducing material	2021	Chemical Engineering Journal
Rohrer M.D.; Prasad H.S.; Savord E.G.	A histologic assessment of a HYBENX® oral tissue decontaminant in vital pulp therapy in dogs	2016	Journal of Biological Regulators and Homeostatic Agents
Furfaro F.; Ang E.S.M.; Lareu R.R.; Murray K.; Goonewardene M.	A histological and micro-CT investigation in to the effect of NGF and EGF on the periodontal, alveolar bone, root and pulpal healing of replanted molars in a rat model - a pilot study	2014	Progress in Orthodontics
Borkent D, Smith S, Dixon PM.	A histological and ultrastructural study of equine peripheral caries	2020	Equine Vet J
Moazzami F.; Ghahramani Y.; Tamaddon A.M.; Dehghani Nazhavani A.; Adl A.	A histological comparison of a new pulp capping material and mineral trioxide aggregate in rat molars	2013	Iranian Endodontic Journal
Oliveira L.V.; da Silva G.R.; Souza G.L.; Magalhães T.E.A.; Barbosa G.L.R.; Turrioni A.P.; Moura C.C.G.	A laboratory evaluation of cell viability, radiopacity and tooth discoloration induced by regenerative endodontic materials	2020	International Endodontic Journal
Germain L.	A logical rationale for endodontic therapy: understanding the rules of the game.	2014	Dentistry today
Caprioglio A.; Conti V.; Caprioglio C.; Capriog D.	A long-term retrospective clinical study on MTA pulpotomies in immature permanent incisors with complicated crown fractures	2014	European Journal of Paediatric Dentistry
Liu W, Martinón-Torres M, Kaifu Y, Wu X, Kono RT, Chang CH, Wei P, Xing S, Huang W, Bermúdez de Castro JM.	A mandible from the Middle Pleistocene Hexian site and its significance in relation to the variability of Asian Homo erectus	2017	Am J Phys Anthropol
Nabavi S, Rezaei M, Rahbaryfar S.	A Maxillary Lateral Incisor with Type V Canal Morphology: A Case Report	2022	Iran Endod J
Geeta I.B.; Galagali G.; Sangeeta K.; Pushpa S.; Noushin F.	A natuaral meliorate: Revolutionary tissue engineering in endodontics	2013	Journal of Clinical and Diagnostic Research
Pinchi V, Pradella F, Buti J, Baldinotti C, Focardi M, Norelli GA.	A new age estimation procedure based on the 3D CBCT study of the pulp cavity and hard tissues of the teeth for forensic purposes: A pilot study	2015	J Forensic Leg Med
Elena R., Meri P., Marija S., Mira J., Radeska-Panovska A., Elizabeta G.	A new calcium-silicate cement in the endodontic treatment of immature permanent teeth: A report of two cases	2017	Research Journal of Pharmaceutical, Biological and Chemical Sciences
Beretta M.; Federici Canova F.	A new method for deep caries treatment in primary teeth using ozone: A retrospective study	2017	European Journal of Paediatric Dentistry
Gandolfi MG.	A New Method for Evaluating the Diffusion of Ca(2+) and OH(-) Ions through Coronal Dentin into the Pulp	2012	Iran Endod J
Mohanty R.; Lenka B.; Nayak R.; Satpathy A.; Das A.C.; Mohanty G.	A new pioneering advancement: Waterlase	2020	Indian Journal of Forensic Medicine and Toxicology
Lo Giudice G., Lizio A.S., Lo Giudice R.	A new software architecture proposal for an evidence-based decision support system in dentistry	2021	Minerva Dental and Oral Science
Robberecht L.; Chai F.; Dehurtevent M.; Marchandise P.; Bécavin T.; Hornez J.-C.; Deveaux E.	A novel anatomical ceramic root canal simulator for endodontic training	2017	European Journal of Dental Education
Iohara K, Murakami M, Takeuchi N, Osako Y, Ito M, Ishizaka R, Utunomiya S, Nakamura H, Matsushita K, Nakashima M.	A novel combinatorial therapy with pulp stem cells and granulocyte colony-stimulating factor for total pulp regeneration	2013	Stem Cells Transl Med

Turkkahraman H, Galindo F, Tulu US, Helms JA.	A novel hypothesis based on clinical, radiological, and histological data to explain the dentinogenesis imperfecta type II phenotype	2020	Connect Tissue Res
Li J.; Wang Z.	A novel NUTM2A-AS1/miR-769–5p axis regulates LPS-evoked damage in human dental pulp cells via the TLR4/MYD88/NF-κB signaling	2022	Journal of Dental Sciences
Cassim I.	A novel use of the Reciproc R25 Endodontic file for root canal obturation	2014	SADJ : journal of the South African Dental Association = tydskrif van die Suid-Afrikaanse Tandheelkundige Vereniging
Foley J.I.	A pan-European comparison of the use of mineral trioxide aggregate (MTA) by postgraduates in paediatric dentistry	2013	European Archives of Paediatric Dentistry
Sánchez-Lara y Tajonar R.G.; Vergara-Tinoco J.V.; Dammaschke T.; Domínguez-Pérez R.A.	A Pilot Feasibility Study to Establish Full Pulpotomy in Mature Permanent Teeth with Symptomatic Irreversible Pulpitis as a Routine Treatment in Mexican Public Healthcare Services	2022	Healthcare (Switzerland)
Shallal-Ayzin M.; Trinh T.; Yeung W.; Tawil P.Z.; Haggerty C.L.; Wu D.; Khan A.A.	A Prospective Analysis of the Correlation Between Postoperative Pain and Vital Pulp Therapy	2021	Frontiers in Dental Medicine
Mente J.; Petrovic J.; Gehrig H.; Rampf S.; Michel A.; Schürz A.; Pfefferle T.; Saure D.; Erber R.	A Prospective Clinical Pilot Study on the Level of Matrix Metalloproteinase-9 in Dental Pulpal Blood as a Marker for the State of Inflammation in the Pulp Tissue	2016	Journal of Endodontics
Gufran K, Mirza MB, Robaian A, Alqahtani AS, Alqhtani NR, Alasqah M, Alsakr AM.	A Prospective Clinical Study Evaluating the Efficacy of Intra-Ligamentary Anesthetic Solutions in Mandibular Molars Diagnosed as Symptomatic Irreversible Pulpitis with Symptomatic Apical Periodontitis	2022	Healthcare (Basel)
Careddu R, Duncan HF.	A prospective clinical study investigating the effectiveness of partial pulpotomy after relating preoperative symptoms to a new and established classification of pulpitis	2021	Int Endod J
Willershausen B, Willershausen I, Ehlers V, Azaripour A, Briseño B.	A prospective clinical trial on the influence of a triamcinolone/demeclocycline and a calcium hydroxide based temporary cement on pain perception	2012	Head Face Med
Kanaa M.D.; Whitworth J.M.; Meechan J.G.	A prospective randomized trial of different supplementary local anesthetic techniques after failure of inferior alveolar nerve block in patients with irreversible pulpitis in mandibular teeth	2012	Journal of Endodontics
Lee J.H.	A radiologic view of migration of a foreign body in the maxillary sinus by mucociliary movement	2015	Ear, Nose and Throat Journal
Petel R.; Ziskind K.; Bernfeld N.; Suliman H.; Fuks A.B.; Moskovitz M.	A randomised controlled clinical trial comparing pure Portland cement and formocresol pulpotomies followed from 2 to 4 years	2021	European Archives of Paediatric Dentistry
Alsanouni M.; Bawazir O.A.	A Randomized Clinical Trial of NeoMTA Plus in Primary Molar Pulpotomies	2019	Pediatric dentistry
Rubanenko M.; Petel R.; Tickotsky N.; Fayer I.; Fuks A.B.; Moskovitz M.	A Randomized Controlled Clinical Trial Comparing Tricalcium Silicate and Formocresol Pulpotomies Followed for Two to Four Years	2019	Pediatric dentistry
Song M.; Kang M.; Kim H.-C.; Kim E.	A randomized controlled study of the use of proroot mineral trioxide aggregate and endocem as direct pulp capping materials	2015	Journal of Endodontics
Jang Y., Song M., Yoo I.-S., Song Y., Roh B.-D., Kim E.	A Randomized Controlled Study of the Use of ProRoot Mineral Trioxide Aggregate and Endocem as Direct Pulp Capping Materials: 3-month versus 1-year Outcomes	2015	Journal of endodontics
Cordell S.; Kratunova E.; Marion I.; Alrayyes S.; Alapati S.B.	A randomized controlled trial comparing the success of mineral trioxide aggregate and ferric sulfate as pulpotomy medicaments for primary molars	2021	Journal of Dentistry for Children
Dhyani V.K., Chhabra S., Sharma V.K., Dhyani A.	A randomized controlled trial to evaluate the incidence of postoperative pain and flare-ups in single and multiple visits root canal treatment	2022	Medical Journal Armed Forces India

Yassaei S., Sharifi M., Ebrahiminik Z.	A rare case of bilateral double incisors; Early orthodontic management	2021	Clinical Case Reports
Bonavolontà P.; Dell'Aversana Orabona G.; Seidita F.; Donna P.; Committeri U.; Abbate V.; D'Andrea L.; Califano L.	A rare case of blindness due to an odontogenic abscess	2020	Chirurgia (Turin)
Saxena A.; Singh A.; Ikhar A.; Chandak M.	A rare case of maxillary first molar with single root and single canal diagnosed using spiral computed tomographic scan	2014	Journal of Indian Society of Pedodontics and Preventive Dentistry
Assunção GSM, Ocarino NM, Sofal LC, Serakides R.	A Rare Case of Radicular Dens Invaginatus (Dens in Dente) in a Dog	2020	J Comp Pathol
Karwa S., Shiggaon L., Waghmare A., Dhavan M.	A regenerative approach using xenograft and PRF membrane in the management of muscosal fenestration in posterior maxilla-A rare case report	2021	Journal of Indian Society of Periodontology
Manivannan S., Mani G., Thamaraiselvan M.	A retrospective analysis of the most frequently root canal treated tooth in pediatric patients visiting a university hospital in Chennai	2020	Indian Journal of Forensic Medicine and Toxicology
Ciftci V.; Yazicioglu İ.	A retrospective comparison of dental treatment under general anesthesia provided for uncooperative healthy patients and patients with special health care needs	2020	Journal of Clinical Pediatric Dentistry
Kaya B.U., Kececi A.D., Guldas H.E., Orhan H.	A retrospective radiographic study of coronal-periapical status and root canal filling quality in a selected adult turkish population	2013	Medical Principles and Practice
Sultana A., Joysuwal R., Nikhita A., Rajeshwari R., Fathima A., Kadam A.	A review on potassium nitrate, chlorhexidine gluconate and hydrogen peroxide toothpaste formulation to treatsensitivity, bleeding gums & whitening of teeth	2021	Journal of Global Trends in Pharmaceutical Sciences
Rathi C., Chandak M., Chandak M., Bajaj P., Chandak P.	A review on safety endodontic management in pregnancy	2020	International Journal of Research in Pharmaceutical Sciences
Meng H, Hu L, Zhou Y, Ge Z, Wang H, Wu CT, Jin J.	A Sandwich Structure of Human Dental Pulp Stem Cell Sheet, Treated Dentin Matrix, and Matrigel for Tooth Root Regeneration	2020	Stem Cells Dev
Gaurav V.; Srivastava N.; Rana V.; Adlakha V.	A study of root canal morphology of human primary incisors and molars using cone beam computerized tomography: An in vitro study	2013	Journal of Indian Society of Pedodontics and Preventive Dentistry
Wellman K.Y.; Dixon P.M.	A Study on the Potential Role of Occlusal Fissure Fractures in the Etiopathogenesis of Equine Cheek Teeth Apical Infections	2019	Journal of Veterinary Dentistry
Chin J.S., Thomas M.B., Locke M., Dummer P.M.	A survey of dental practitioners in Wales to evaluate the management of deep carious lesions with vital pulp therapy in permanent teeth	2016	British dental journal
Foley J.	A survey of paediatric caries management teaching within dental therapy programmes in the UK	2017	British Dental Journal
Halawany H.S., Salama F., Jacob V., Abraham N.B., Moharib T.N.B., Alazmah A.S., Al Harbi J.A.	A survey of pediatric dentists' caries-related treatment decisions and restorative modalities – A web-based survey	2017	Saudi Dental Journal
Lopes-Fatturi A., de Souza J.F., Menezes J.V.N.B., Fraiz F.C., Assunção L.R.D.S.	A survival analysis of different pulp therapies in decayed primary teeth	2020	Pesquisa Brasileira em Odontopediatria e Clinica Integrada
Coll J.A., Vargas K., Marghalani A.A., Chen C.-Y., AlShamali S., Dhar V., Crystal Y.O.	A Systematic Review and Meta-Analysis of Nonvital Pulp Therapy for Primary Teeth	2020	Pediatric dentistry
do Couto A.M.; Espaladori M.C.; Leite A.P.P.; Martins C.C.; de Aguiar M.C.F.; Abreu L.G.	A Systematic Review of Pulp Revascularization Using a Triple Antibiotic Paste	2019	Pediatric dentistry
Fathi Z, Rahimi S, Tavakoli R, Amini M.	A Three-rooted Mandibular Second Premolar: A Case Report	2014	J Dent Res Dent Clin Dent Prospects
Deepalakshmi M.; Rajasekaran M.S.; Kumar A.; Indira R.; Ramachandran S.	Aberrant canal configuration of the maxillary first molar: A case report	2012	Journal of Clinical and Diagnostic Research
Rajalbandi S.; Shingte S.N.; Sundaresh K.J.; Mallikarjuna R.	Aberration in the palatal root of the maxillary first molar	2013	BMJ Case Reports
Wang Y.; Zhai S.; Wang H.; Jia Q.; Jiang W.; Zhang X.; Zhang A.; Liu J.; Ni L.	Absent in melanoma 2 (AIM2) in rat dental pulp mediates the inflammatory response during pulpitis	2013	Journal of Endodontics

Efthymiou A.; Marques M.M.; Franzen R.; Moreira M.S.; Gutknecht N.	Acceptance and efficiency of anesthesia by photobiomodulation therapy during conventional cavity preparation in permanent teeth: a pilot randomized crossover clinical study	2017	Lasers in Dental Science
Lugliè P.F.; Grabesu V.; Spano G.; Lumbau A.	Accessory foramina in the furcation area of primary molars. A SEM investigation	2012	European Journal of Paediatric Dentistry
Libotte F., Ciolfi C., Romeo U.	Accessory root canals: Tips and tricks	2017	Dental Cadmos
Li J, Pan YH, Zhao Y, Li L.	Accuracy of 3D reconstruction of pulp cavity in mandibular premolars based on cone-beam CT	2018	Shanghai Kou Qiang Yi Xue
Hazard M.L.; Wicker C.; Qian F.; Williamson A.E.; Teixeira F.B.	Accuracy of cold sensibility testing on teeth with full-coverage restorations: a clinical study	2021	International Endodontic Journal
Matos F.S.; Cunha T.C.; Ribeiro M.A.G.; Araujo C.S.; Bernardino Í.M.; Moura C.C.G.; Paranhos L.R.	Accuracy of the dental pulp sensibility test using cold spray for the diagnosis of pulp diseases: An observational clinical study	2021	Bioscience Journal
Medina-Fernandez I , Celiz AD .	Acellular biomaterial strategies for endodontic regeneration	2019	Biomater Sci
Vu T.T., Nguyen M.T., Sangvanich P., Nguyen Q.N., Thunyakitpisal P.	Acemannan used as an implantable biomaterial for vital pulp therapy of immature permanent teeth induced continued root formation	2020	Pharmaceutics
Azim A.A.; Azim K.A.; Deutsch A.S.; Huang G.T.-J.	Acquisition of anatomic parameters concerning molar pulp chamber landmarks using cone-beam computed tomography	2014	Journal of endodontics
Zhai Y.; Wang Y.; Rao N.; Li J.; Li X.; Fang T.; Zhao Y.; Ge L.	Activation and Biological Properties of Human β Defensin 4 in Stem Cells Derived From Human Exfoliated Deciduous Teeth	2019	Frontiers in Physiology
Ma L.; Wang S.C.; Tong J.; Hu Y.; Zhang Y.Q.; Yu Q.	Activation and dynamic expression of Notch signalling in dental pulp cells after injury in vitro and in vivo	2016	International Endodontic Journal
He Q.; Wang H.; Fan M.; Zhang L.; Huang S.; Li Y.	Activation of autophagy in pulpitis is associated with TLR4	2017	International Journal of Clinical and Experimental Pathology
Yang F.; Li Y.; Duan H.; Wang H.; Pei F.; Chen Z.; Zhang L.	Activation of mitophagy in inflamed odontoblasts	2019	Oral Diseases
Jiang W.; Lv H.; Wang H.; Wang D.; Sun S.; Jia Q.; Wang P.; Song B.; Ni L.	Activation of the NLRP3/caspase-1 inflammasome in human dental pulp tissue and human dental pulp fibroblasts	2015	Cell and Tissue Research
Filippini H.F.; Scalzilli P.A.; Costa K.M.; Freitas R.D.S.; Campos M.M.	Activation of trigeminal ganglion satellite glial cells in CFA-induced tooth pulp pain in rats	2018	PLoS ONE
Selvakumar DR, Krishnamoorthy S, Venkatesan K, Ramanathan A, Abbott PV, Angambakkam Rajasekaran P.	Active Bacteria in Carious Dentin of Mandibular Molars with Different Pulp Conditions: An In Vivo Study	2021	J Endod
Katifelis H, Sioziou A, Gazouli M, Emmanouil D.	ACTN2 (rs6656267) and MPPED2 (rs11031093 and rs536007) polymorphisms in primary dentition caries: A case-control study	2020	Int J Paediatr Dent
Haug S.R.; Marthinussen M.C.	Acute dental pain and salivary biomarkers for stress and inflammation in patients with pulpal or periapical inflammation	2019	Journal of Oral and Facial Pain and Headache
Gürler E.B.; İriboz E.; Kaya ÖT.Ç.; Türkaydin D.; Öveçoğlu H.S.	Acute dental pain elevates salivary oxytocin in women: a risk factor during pregnancy	2021	General dentistry
Vailati F.; Gruetter L.; Belser U.C.	Adhesively restored anterior maxillary dentitions affected by severe erosion: up to 6-year results of a prospective clinical study.	2013	The European journal of esthetic dentistry : official journal of the European Academy of Esthetic Dentistry
Xu X, Liang C, Gao X, Huang H, Xing X, Tang Q, Yang J, Wu Y, Li M, Li H, Liao L, Tian W.	Adipose Tissue-derived Microvascular Fragments as Vascularization Units for Dental Pulp Regeneration	2021	J Endod
Winderlich J.N.; Kremer K.L.; Koblar S.A.	Adult human dental pulp stem cells promote blood-brain barrier permeability through vascular endothelial growth factor-a expression	2016	Journal of Cerebral Blood Flow and Metabolism
Rôças I.N.; Lima K.C.; Assunção I.V.; Gomes P.N.; Bracks I.V.; Siqueira J.F., Jr.	Advanced Caries Microbiota in Teeth with Irreversible Pulpitis	2015	Journal of Endodontics

Andrade VM, Fontenele RC, de Souza AC, Almeida CA, Vieira AC, Groppo FC, Freitas DQ, Junior ED.	Age and sex estimation based on pulp cavity volume using cone beam computed tomography: development and validation of formulas in a Brazilian sample	2019	Dentomaxillofac Radiol
Nemsi H, Haj Salem N, Bouanene I, Ben Jomaa S, Belhadj M, Mosrati MA, Aissaoui A, Ben Amor F, Chadly A.	Age assessment in canine and premolar by cervical axial sections of cone-beam computed tomography	2017	Leg Med (Tokyo)
Sakuma A, Saitoh H, Suzuki Y, Makino Y, Inokuchi G, Hayakawa M, Yajima D, Iwase H.	Age estimation based on pulp cavity to tooth volume ratio using postmortem computed tomography images	2013	J Forensic Sci
Gulsahi A, Kulah CK, Bakirarar B, Gulen O, Kamburoglu K.	Age estimation based on pulp/tooth volume ratio measured on cone-beam CT images	2018	Dentomaxillofac Radiol
Alharbi HS Sr, Alharbi AM, Alenazi AO, Kolarkodi SH, Elmoazen R.	Age Estimation by Kvaal's Method Using Digital Panoramic Radiographs in the Saudi Population	2022	Cureus
Koranne VV, Mhapuskar AA, Marathe SP, Joshi SA, Saddiwal RS, Nisa SU.	Age estimation in Indian adults by the coronal pulp cavity index	2017	J Forensic Dent Sci
Badar SB, Ghafoor R, Khan FR, Hameed MH.	Age estimation of a sample of Pakistani population using Coronal Pulp Cavity Index in molars and premolars on Orthopantomogram	2016	J Pak Med Assoc
Karkhanis S, Mack P, Franklin D.	Age estimation standards for a Western Australian population using the coronal pulp cavity index	2013	Forensic Sci Int
Talabani RM, Baban MT, Mahmood MA.	Age estimation using lower permanent first molars on a panoramic radiograph: A digital image analysis	2015	J Forensic Dent Sci
Timme M, Borkert J, Nagelmann N, Streeter A, Karch A, Schmeling A.	Age-dependent decrease in dental pulp cavity volume as a feature for age assessment: a comparative in vitro study using 9.4-T UTE-MRI and CBCT 3D imaging	2021	Int J Legal Med
Ning T.; Shao J.; Zhang X.; Luo X.; Huang X.; Wu H.; Xu S.; Wu B.; Ma D.	Ageing affects the proliferation and mineralization of rat dental pulp stem cells under inflammatory conditions	2020	International Endodontic Journal
Aslantas E.E.; Buzoglu H.D.; Karapinar S.P.; Cehreli Z.C.; Muftuoglu S.; Atilla P.; Aksoy Y.	Age-related Changes in the Alkaline Phosphatase Activity of Healthy and Inflamed Human Dental Pulp	2016	Journal of Endodontics
Sue M, Oda T, Sasaki Y, Ogura I.	Age-related changes in the pulp chamber of maxillary and mandibular molars on cone-beam computed tomography images	2018	Oral Radiol
Dosedělová H, Štěpánková K, Zikmund T, Lesot H, Kaiser J, Novotný K, Štembírek J, Knotek Z, Zahradníček O, Buchtová M.	Age-related changes in the tooth-bone interface area of acrodont dentition in the chameleon	2016	J Anat
Vehkalahti M.M.; Palotie U.; Valaste M.	Age-specific findings on endodontic treatments performed by private dentists in Finland in 2012 and 2017: a nationwide register-based observation	2020	International Endodontic Journal
Shen S.; Shang L.; Liu H.; Liang Q.; Liang W.; Ge S.	AGGF1 inhibits the expression of inflammatory mediators and promotes angiogenesis in dental pulp cells	2021	Clinical Oral Investigations
Lee Y.-H.; Kim G.-E.; Cho H.-J.; Yu M.-K.; Bhattarai G.; Lee N.-H.; Yi H.-K.	Aging of in vitro pulp illustrates change of inflammation and dentinogenesis	2013	Journal of Endodontics
Huang S.; Song Z.; Huang Q.; Jiang L.; Chen L.; Wang R.; Lin Z.	AIM2 Inflammasome Is Critical for dsDNA-Induced IL-1β Secretion in Human Dental Pulp Cells	2018	Inflammation
Ho S.-W.; Lue K.-H.; Ku M.-S.	Allergic rhinitis, rather than asthma, might be associated with dental caries, periodontitis, and other oral diseases in adults	2019	PeerJ
Elheeny A.A.H.	Allium sativum extract as an irrigant in pulpectomy of primary molars: A 12-month short-term evaluation	2019	Clinical and experimental dental research
Cordero C.B.; Santander G.M.; González D.U.; Quezada A.; Silva C.I.; Vásquez C.; Jara R.; Jara D.; Khoury M.	Allogeneic Cellular Therapy in a Mature Tooth with Apical Periodontitis and Accidental Root Perforation: A Case Report	2020	Journal of Endodontics
Iohara K.; Utsunomiya S.; Kohara S.; Nakashima M.	Allogeneic transplantation of mobilized dental pulp stem cells with the mismatched dog leukocyte antigen type is safe and efficacious for total pulp regeneration	2018	Stem Cell Research and Therapy
Grawish M.E.-A.; Khounganian R.; Hamam M.K.; Zaher A.R.; Hegazy D.; El-Negoly S.A.E.-R.; Hassan G.; Zyada M.M.	Altered coronal tissue of the human dental pulp in chronic hepatitis C virus infected patients	2013	Journal of Endodontics

Gonzalez Marrero Y.; Kobayashi Y.; Ihsan M.S.; Pilch L.A.; Chen L.; Jiang S.; Ye Y.; Fine D.H.; Falcon C.Y.; Falcon P.A.; Hirschberg C.S.; Shimizu E.	Altered Prevalence of Pulp Diagnoses in Diabetes Mellitus Patients: A Retrospective Study	2022	Journal of Endodontics
Mello B.Z., Stafuzza T.C., Vitor L.L., Rios D., Machado M.A., Oliveira T.M.	Alternative approach for carious tissue removal in primary teeth	2016	European archives of paediatric dentistry : official journal of the European Academy of Paediatric Dentistry
Agarwal R.; Chaudhry K.; Yeluri R.; Singh C.; Munshi A.K.	Alternative approach to management of early loss of second primary molar: a clinical case report.	2014	Journal of the California Dental Association
Santamaría R.M.; Innes N.P.T.; Machiulskiene V.; Schmoeckel J.; Alkilzy M.; Splieth C.H.	Alternative Caries Management Options for Primary Molars: 2.5-Year Outcomes of a Randomised Clinical Trial	2018	Caries Research
Hussain M.I.; Bashar A.K.M.; Zakir Hossain Shikder A.H.M.	Alternative Management of Acute Irreversible Pulpitis of an Adult HIV-Positive Patient: A Case Report	2021	Bangladesh Medical Research Council Bulletin
de Amorim R.G., Leal S.C., Mulder J., Creugers N.H., Frencken J.E.	Amalgam and ART restorations in children: a controlled clinical trial	2014	Clinical oral investigations
Huang Y, Goldberg M, Le T, Qiang R, Warner D, Witkowska HE, Liu H, Zhu L, Denbesten P, Li W.	Amelogenin exons 8 and 9 encoded peptide enhances leucine rich amelogenin peptide mediated dental pulp repair	2012	Cells Tissues Organs
Abou Chedid JC, Mchayleh N, Khalil I, Melki B, Hardan LS.	An 18 month evaluation of MM-MTA pulpotomy on primary decayed molars	2015	Odontostomatol Trop
Subramanyam D.	An aesthetic reconstruction of grossly decayed primary anterior teeth - A case report	2020	International Journal of Pharmaceutical Sciences and Research
Hughes-Stamm S, Warnke F, van Daal A.	An alternate method for extracting DNA from environmentally challenged teeth for improved DNA analysis	2016	Leg Med (Tokyo)
Yong J.B., Sivarajan S., Abbott P.V.	An analysis of the timing and materials associated with pulp disease following restorative dental treatment	2018	International endodontic journal
Akram A.; Zamzam R.; Mohamad N.B.; Abdullah D.; Meerah S.M.	An assessment of the prescribing skills of undergraduate dental students in malaysia	2012	Journal of Dental Education
Fujii R, Muramatsu T, Yamaguchi Y, Asai T, Aida N, Suehara M, Morinaga K, Furusawa M.	An endodontic-periodontal lesion with primary periodontal disease: a case report on its bacterial profile	2014	Bull Tokyo Dent Coll
Othman N.I.; Ismail H.U.; Mohammad N.; Ghazali N.; Alauddin M.S.	An Evaluation on Deep Caries Removal Method and Management Performed by Undergraduate Dental Students: A Malaysia Experience	2021	European Journal of Dentistry
Khirtika S.G., Ramesh S.	An evidence based decision analysis for criteria for selection of post endodontic restoration	2017	Journal of Pharmaceutical Sciences and Research
Pourhajibagher M.; Raoofian R.; Ghorbanzadeh R.; Bahador A.	An experimental study for rapid detection and quantification of endodontic microbiota following photo-activated disinfection via new multiplex real-time PCR assay	2018	Photodiagnosis and Photodynamic Therapy
Zhang YA, Guan WQ, Li Q, Liu LL.	An experimental study of simvastatin-collagen composite sponge as a direct pulp capping material in rat molars	2019	Hua Xi Kou Qiang Yi Xue Za Zhi
Bashyam R.; Krishnan R.; Murali K.; Selvarajan N.B.; Vasaviah S.K.; Duraisamy V.	An in vitro assessment of the apical sealing ability of mta plus and biodentin	2021	International Journal of Current Research and Review
Singh G.; Elshamy F.M.M.; Homeida H.E.; Boreak N.; Gupta I.	An in vitro comparison of antimicrobial activity of three endodontic sealers with different composition	2016	Journal of Contemporary Dental Practice
Mandrol P.S.; Bhat K.; Prabhakar A.R.	An in vitro evaluation of cytotoxicity of curcumin against human dental pulp fibroblasts	2016	Journal of Indian Society of Pedodontics and Preventive Dentistry
Sangwan B.; Rishi R.; Sea M.; Jain K.; Dutt P.; Talukdar P.	An in vitro evaluation of fracture resistance of endodontically treated teeth with different restorative materials	2016	Journal of Contemporary Dental Practice
Datta P.; Zahir S.; Kundu G.K.; Dutta K.	An in vitro study of root canal system of human primary molars by using multidetector computed tomography	2019	Journal of Indian Society of Pedodontics and Preventive Dentistry

Paludo L.; de Souza S.L.; Só M.V.R.; da Rosa R.A.; Vier-Pelisser F.V.; Duarte M.A.H.	An in vivo radiographic evaluation of the accuracy of apex and ipex electronic apex locators	2012	Brazilian Dental Journal
Pereira KF, Silva PG, Vicente FS, Arashiro FN, Coldebella CR, Ramos CA.	An in vivo study of working length determination with a new apex locator	2014	Braz Dent J
Nugraha A.P., Ramadhani N.F., Saputra D., Mappananrang R.A., Purnamasari A.E., Anwar A.A., Ridwan R.D., Kharisma V.D., Nugraha A.P., Noor T.N.E.T.A., Nawira, Widjiastuti I., Yuanita T.	An Insight of Proanthocyanidin and Polyamidoamine-Calcium Phosphate Nanoparticles as Biomaterial Candidate for Dentin regeneration in Dental Pulp Capping: A Narrative Review	2022	Research Journal of Pharmacy and Technology
Liaw J.J.-L., Park J.H., Chang C.-C., Wang S.-H., Tsai B.M.-Y.	An interdisciplinary approach to orthodontic treatment of a mutilated Class III malocclusion with mini-implants, dental implants, and an autotransplant	2022	Journal of Esthetic and Restorative Dentistry
Ahmed F.; Anupama Nayak P.; Rao A.; Rao A.	An overview of non-syndromic mesotaurodontism in bilateral primary mandibular first molars: A rare case report	2020	International Journal of Clinical Dentistry
Dantas Costa L.E.; de Sousa S.A.; Serpa E.B.M.; Duarte R.C.	An overview of teaching of pulp therapy in primary teeth in undergraduate dental courses; Panorama do ensino da terapia pulpar em dentes decíduos nos cursos de graduação em odontologia	2012	Pesquisa Brasileira em Odontopediatria e Clinica Integrada
Botelho J.; Mascarenhas P.; Viana J.; Proença L.; Orlandi M.; Leira Y.; Chambrone L.; Mendes J.J.; Machado V.	An umbrella review of the evidence linking oral health and systemic noncommunicable diseases	2022	Nature Communications
Alklayb S.A., Divakar D.D.	An uncommon tooth fusion of mandibular primary lateral incisor with canine	2021	Clinics and Practice
Rajasekaran S.; Sargod S.S.; Bhat S.S.	AN UNUSUAL CASE OF DENS IN DENTE WITH RAREFYING OSTEITIS	2021	International Journal of Clinical Dentistry
Laham A.; Clouet R.; del Valle G.A.; Gaudin A.; Prud'homme T.	Anaesthetic efficacy and influence on cardiovascular parameters change of intraosseous computerised anaesthesia versus inferior alveolar nerve block anaesthesia in acute irreversible pulpitis of mandibular molars: study protocol for a prospective randomised controlled trial	2022	Trials
Romero S.S.B.; Lee Y.-C.; Fuh L.-J.; Chung H.-Y.; Hung S.-Y.; Chen Y.-H.	Analgesic and neuroprotective effects of electroacupuncture in a dental pulp injury model-a basic research	2020	International Journal of Molecular Sciences
Pavithra P.; Dhanraj M.; Sekhar P.	Analgesic effectiveness of Ibuprofen and Aceclofenac in the management of acute pulpitis - A randomized double blind trial	2015	International Journal of Pharmaceutical Sciences Review and Research
Cinthura C.; Ganapathy D.; Rajasekar A.	Analgesic prescription practice for Pulpal pain — an institution-based retrospective study	2020	International Journal of Research in Pharmaceutical Sciences
Feigin K.; Bell C.; Shope B.; Henzel S.; Snyder C.	Analysis and Assessment of Pulp Vitality of 102 Intrinsically Stained Teeth in Dogs	2022	Journal of Veterinary Dentistry
Salas-López E.K.; Casas-Flores S.; López-Lozano N.E.; Layseca-Espinosa E.; García-Sepúlveda C.A.; Niño-Moreno P.C.; Pozos-Guillén A.	Analysis of bacterial communities of infected primary teeth in a Mexican population	2020	Medicina Oral Patologia Oral y Cirugia Bucal
Mashyakhy M.; Abu-Melha A.S.	Analysis of Bilateral Symmetry of Root Canal Anatomy in Permanent Dentition: An In Vivo CBCT Study in a Saudi Arabian Population	2021	Journal of Contemporary Dental Practice
Wu XR, Xia B, Ge LH, Qin M, Li RZ, Wang B, Ge FQ, Wang XJ, Chen X, Song GT, Shao LQ, Wang J, Zou J, Lin JJ, Zhao YM, Mei YF, Huang H, Zeng SJ.	Analysis of caries experience and the dental treatments under general anesthesia in 103 cases of children with autism spectrum disorders	2020	Zhonghua Kou Qiang Yi Xue Za Zhi
Costa V.A., Paixão L.C., Ferreira E.F., Sobrinho A.P.R., Martins R.C.	Analysis of dental teleconsulting in the pediatric dentistry field of telehealth minas gerais: A cross-sectional study	2021	Pesquisa Brasileira em Odontopediatria e Clinica Integrada
Ahad M., Pradeep S.	Analysis of factors causing extraction of endodontically treated teeth-a retroprospective study	2020	Indian Journal of Public Health Research and Development
Wang N.-N.; Ge J.-Y.; Xie S.-J.; Chen G.; Zhu M.	Analysis of Mtwo Rotary Instrument Separation During Endodontic Therapy: A Retrospective Clinical Study	2014	Cell Biochemistry and Biophysics

Wang F, Qiao W, Wang F, Meng L.	Analysis of Online Consultations and Emergent Treatments of Operative Dentistry and Endodontics during the COVID-19 Epidemic	2022	Int J Environ Res Public Health
Yi Q, Liu O, Yan F, Lin X, Diao S, Wang L, Jin L, Wang S, Lu Y, Fan Z.	Analysis of Senescence-Related Differentiation Potentials and Gene Expression Profiles in Human Dental Pulp Stem Cells	2017	Cells Tissues Organs
Chen Y.; Li H.; Li M.; Yang L.; Sun Q.; Chen K.	Analysis of survival and factors associated with failure of primary tooth pulpectomies performed under general anaesthesia in children from South China	2020	International Journal of Paediatric Dentistry
Shi R.; Yang H.; Lin X.; Cao Y.; Zhang C.; Fan Z.; Hou B.	Analysis of the characteristics and expression profiles of coding and noncoding RNAs of human dental pulp stem cells in hypoxic conditions	2019	Stem Cell Research and Therapy
Yang S.-E.; Jo A.-R.; Lee H.-J.; Kim S.-Y.	Analysis of the characteristics of cracked teeth and evaluation of pulp status according to periodontal probing depth	2017	BMC Oral Health
Ke J.; Cai Q.; Zhang C.; Du O.; Wang F.; Luo Y.; Li W.; Ou D.	Analysis of the Efficacy and Safety of Pulpitis Treated with Different Root Canal Flushing Fluids Based on VAS and Temporomandibular Joint Function	2022	Contrast Media and Molecular Imaging
Sharif R.A., Chaturvedi S., Suleman G., Elmahdi A.E., Elagib M.F.A.	Analysis of tooth extraction causes and patterns	2020	Open Access Macedonian Journal of Medical Sciences
Krishnamurthy N.H., Jacob C.D., Thimmegowda U., Ramachandra J.A., Arali V., Bhat P.K.	Anatomical configuration of roots and canal morphology of primary mandibular first molars: A CBCT study	2017	Journal of Clinical and Diagnostic Research
Argueta-Figueroa L.; Arzate-Sosa G.; Mendieta-Zeron H.	Anesthetic efficacy of articaine for inferior alveolar nerve blocks in patients with symptomatic versus asymptomatic irreversible pulpitis	2012	General Dentistry
Shakoui S, Ghodrati M, Ghasemi N, Pourlak T, Abdollahi AA.	Anesthetic efficacy of articaine/epinephrine plus mannitol in comparison with articaine/epinephrine anesthesia for inferior alveolar nerve block in patients with symptomatic irreversible pulpitis: A randomized controlled clinical trial	2019	J Dent Res Dent Clin Dent Prospects
Kreimer T.; Kiser II R.; Reader A.; Nusstein J.; Drum M.; Beck M.	Anesthetic efficacy of combinations of 0.5 mol/L mannitol and lidocaine with epinephrine for inferior alveolar nerve blocks in patients with symptomatic irreversible pulpitis	2012	Journal of Endodontics
Mohajeri L.; Salehi F.; Mehrvarzfar P.; Arfaee H.; Bohluli B.; Hamedy R.	Anesthetic efficacyofmeperidineinteeth with symptomatic irreversible pulpitis	2015	Anesthesia Progress
Bindal P.; Gnanasegaran N.; Bindal U.; Haque N.; Ramasamy T.S.; Chai W.L.; Kasim N.H.A.	Angiogenic effect of platelet-rich concentrates on dental pulp stem cells in inflamed microenvironment	2019	Clinical Oral Investigations
Li W.-J.; Shen J.	Antagonism of G protein-coupled receptor 55 prevents lipopolysaccharide-induced damages in human dental pulp cells	2022	Human and Experimental Toxicology
Lee S.-H.; Baek D.-H.	Antibacterial and neutralizing effect of human β-defensins on enterococcus faecalis and enterococcus faecalis lipoteichoic acid	2012	Journal of Endodontics
Saatchi M.; Shokraneh A.; Navaei H.; Maracy M.R.; Shojaei H.	Antibacterial effect of calcium hydroxide combined with chlorhexidine on Enterococcus faecalis: A systematic review and meta-analysis	2014	Journal of Applied Oral Science
Tinoco J.M.; Liss N.; Zhang H.; Nissan R.; Gordon W.; Tinoco E.; Sassone L.; Stevens R.	Antibacterial effect of genetically-engineered bacteriophage ϕEf11/ϕFL1C(Δ36)PnisA on dentin infected with antibiotic-resistant Enterococcus faecalis	2017	Archives of Oral Biology
Shrestha A.; Kishen A.	Antibacterial efficacy of photosensitizer functionalized biopolymeric nanoparticles in the presence of tissue inhibitors in root canal	2014	Journal of Endodontics
Valverde M.E.; Baca P.; Ceballos L.; Fuentes M.V.; Ruiz-Linares M.; Ferrer-Luque C.M.	Antibacterial efficacy of several intracanal medicaments for endodontic therapy	2017	Dental Materials Journal
Soligo L.T.; Lodi E.; Farina A.P.; Souza M.A.; Vidal C.M.P.; Cecchin D.	Antibacterial efficacy of synthetic and natural-derived novel endodontic irrigant solutions	2018	Brazilian Dental Journal
Del Carpio-Perochena A.; Kishen A.; Shrestha A.; Bramante C.M.	Antibacterial Properties Associated with Chitosan Nanoparticle Treatment on Root Dentin and 2 Types of Endodontic Sealers	2015	Journal of Endodontics
Yamamoto L.Y.; Loureiro C.; Cintra L.T.A.; Leonardo R.D.T.; Banci H.A.; Ribeiro A.P.F.; Sivieri-Araujo G.; Jacinto R.D.C.	Antibiofilm activity of laser ablation with indocyanine green activated by different power laser parameters compared with photodynamic therapy on root canals infected with Enterococcus faecalis	2021	Photodiagnosis and Photodynamic Therapy

Afrasiabi S.; Pourhajibagher M.; Chiniforush N.; Aminian M.; Bahador A.	Anti-biofilm and anti-metabolic effects of antimicrobial photodynamic therapy using chlorophyllin-phycocyanin mixture against Streptococcus mutans in experimental biofilm caries model on enamel slabs	2020	Photodiagnosis and Photodynamic Therapy
Marra F.; George D.; Chong M.; Sutherland S.; Patrick D.M.	Antibiotic prescribing by dentists has increased Why?	2016	Journal of the American Dental Association
Abraham S.B.; Abdulla N.; Himratul-Aznita W.H.; Awad M.; Samaranayake L.P.; Ahmed H.M.A.	Antibiotic prescribing practices of dentists for endodontic infections; a cross-sectional study	2020	PLoS ONE
Rôças I.N.; Siqueira J.F.	Antibiotic resistance genes in anaerobic bacteria isolated from primary dental root canal infections	2012	Anaerobe
Relan K., Chandak M., Chandak P., Chandak M., Rathi C., Mishra S.	Antibiotics: A changing scenario in regenerative endodontics	2020	International Journal of Research in Pharmaceutical Sciences
Yan W.; Yang F.; Liu Z.; Wen Q.; Gao Y.; Niu X.; Zhao Y.	Anti-Inflammatory and Mineralization Effects of an ASP/PLGA-ASP/ACP/PLLA-PLGA Composite Membrane as a Dental Pulp Capping Agent	2022	Journal of Functional Biomaterials
Hong J.-H.; Kim M.-R.; Lee B.-N.; Oh W.-M.; Min K.-S.; Im Y.-G.; Hwang Y.-C.	Anti-inflammatory and mineralization effects of bromelain on lipopolysaccharide-induced inflammation of human dental pulp cells	2021	Medicina (Lithuania)
Meto A, Meto A, Bimbari B, Shytaj K, Özcan M.	Anti-Inflammatory and Regenerative Effects of Albanian Propolis in Experimental Vital Amputations	2016	Eur J Prosthodont Restor Dent
Wang W.; Wang X.; Li L.; Liu Y.	Anti-Inflammatory and Repairing Effects of Mesoporous Silica-Loaded Metronidazole Composite Hydrogel on Human Dental Pulp Cells	2022	Journal of Healthcare Engineering
Dennis D.; Abidin T.; Tarigan R.; Zudhistira C.	Anti-inflammatory effect of mangosteen rind extract in rabbit teeth with reversible pulpitis (In vivo study)	2018	International Journal of Clinical Dentistry
Horie N.; Hashimoto K.; Hino S.; Kato T.; Shimoyama T.; Kaneko T.; Kusama K.; Sakagami H.	Anti-inflammatory potential of rikkosan based on IL-1β network through macrophages to oral tissue cells	2014	In Vivo
Nara K.; Kawashima N.; Noda S.; Fujii M.; Hashimoto K.; Tazawa K.; Okiji T.	Anti-inflammatory roles of microRNA 21 in lipopolysaccharide-stimulated human dental pulp cells	2019	Journal of Cellular Physiology
Pourhajibagher M.; Chiniforush N.; Bahador A.	Antimicrobial action of photoactivated C-Phycocyanin against Enterococcus faecalis biofilms: Attenuation of quorum-sensing system	2019	Photodiagnosis and Photodynamic Therapy
Long J.; Kreft J.U.; Camilleri J.	Antimicrobial and ultrastructural properties of root canal filling materials exposed to bacterial challenge	2020	Journal of Dentistry
Jimenez-Gonzalez H.A.; Nakagoshi-Cepeda M.A.A.; Nakagoshi-Cepeda S.E.; Urrutia-Baca V.H.; De La Garza-Ramos M.A.; Solis-Soto J.M.; Gomez-Flores R.; Tamez-Guerra P.	Antimicrobial Effect of Calcium Hydroxide Combined with Electrolyzed Superoxidized Solution at Neutral pH on Enterococcus faecalis Growth	2021	BioMed Research International
de Miranda R.G.; Gusman H.D.S.; Colombo A.P.V.	Antimicrobial efficacy of the EndoVac system plus PDT against intracanal Candida albicans: an ex vivo study	2015	Brazilian Oral Research
Kiran N.K., Chowdhary N., Sharada Devi Mannur Y., Varghese N.E., Sridhara A., Pavana M.P.	Antimicrobial efficacy of triphala as root canal irrigating solution in infected primary teeth: An ex vivo study	2020	Indian Journal of Public Health Research and Development
Tavares W.L.F.; Ferreira M.V.L.; de Carvalho Machado V.; Braga T.; Amaral R.R.; Cohen S.	Antimicrobial photodynamic therapy and guided endodontics: A case report	2020	Photodiagnosis and Photodynamic Therapy
Okamoto C.B.; Motta L.J.; Prates R.A.; da Mota A.C.C.; Gonçalves M.L.L.; Horliana A.C.R.T.; Mesquita Ferrari R.A.; Fernandes K.P.S.; Bussadori S.K.	Antimicrobial Photodynamic Therapy as a Co-adjuvant in Endodontic Treatment of Deciduous Teeth: Case Series	2018	Photochemistry and Photobiology
da Silva C.C.; Chaves Júnior S.P.; Pereira G.L.D.; Fontes K.B.F.C.; Antunes L.A.A.; Póvoa H.C.C.; Antunes L.S.; Iorio N.L.P.P.	Antimicrobial Photodynamic Therapy Associated with Conventional Endodontic Treatment: A Clinical and Molecular Microbiological Study	2018	Photochemistry and Photobiology
Silva L.A.B.; Novaes Jr. A.B.; De Oliveira R.R.; Nelson-Filho P.; Santamaria Jr. M.; Silva R.A.B.	Antimicrobial photodynamic therapy for the treatment of teeth with apical periodontitis: A histopathological evaluation	2012	Journal of Endodontics

Fernandes M.L.D.M.F.; Maia C.A.; Santos A.M.C.; Vilela C.R.; Araujo F.R.; Mohallen M.L.; Silveira L.B.; Fernandes A.M.	Antimicrobial photodynamic therapy in the endodontic treatment of deciduous teeth: In vivo pilot study	2020	Pesquisa Brasileira em Odontopediatria e Clinica Integrada
Lins RX, de Oliveira Andrade A, Hirata Junior R, Wilson MJ, Lewis MA, Williams DW, Fidel RA.	Antimicrobial resistance and virulence traits of Enterococcus faecalis from primary endodontic infections	2013	J Dent
Micallef S.; Azzopardi C.M.	Antiphospholipid syndrome masquerading as a case of infective endocarditis	2018	BMJ Case Reports
Costa T., Ferreira E., Antunes L., Dinis P.B.	Antral Bony Wall Erosion, Trigeminal Nerve Injury, and Enophthalmos after Root Canal Surgery	2016	Allergy and Rhinology
Quiñones Pedraza J.A., Flores Treviño J.J., Cruz Fierro N., González Meléndez R., Elizondo Elizondo J., Zavala Vargas L.A., De La Rosa Moreno D.A.	Apexification in a mandibular first molar with a middle mesial canal	2018	Clinical Case Reports
Paulo A.O.; Tanomaru-Filho M.; Leonardo R.T.; Chávez-Andrade G.M.; Guerreiro-Tanomaru J.M.	Apexification with white MTA in an immature permanent tooth with dens invaginatus	2013	Brazilian Journal of Oral Sciences
Shalan L.A., Al-Huwaizi H.F., Fatalla A.A.	Apical Extrusion of Debris and Irrigants after using Different Irrigation Needles and Systems with Different Depth of Penetration: (A Comparative Study)	2018	Biomedical and Pharmacology Journal
Sağlam B.C.; Çiçek E.; Koçak M.M.; Koçak S.; Kıvanç B.H.	Apical transportation during removal of root canal filling with different retreatment systems	2016	International Journal of Artificial Organs
Hatice BD, Nihal A, Nursel A, Humeyra Ozge Y, Goksuluk D.	Applicability of Cameriere's and Drusini's age estimation methods to a sample of Turkish adults	2017	Dentomaxillofac Radiol
Wei Y., Wang J., Dai D., Wang H., Zhang M., Zhang Z., Zhou X., He L., Cheng L.	Application of a Caries Treatment Difficulty Assessment System in Dental Caries Management	2022	International Journal of Environmental Research and Public Health
Zhang F.; Wu S.; Qu M.; Zhou L.	Application of a Remotely Controlled Artificial Intelligence Analgesic Pump Device in Painless Treatment of Children	2022	Contrast Media and Molecular Imaging
Chakka L.R.J.; Vislisel J.; Vidal C.M.P.; Biz M.T.; K. Salem A.; Cavalcanti B.N.	Application of BMP-2/FGF-2 gene–activated scaffolds for dental pulp capping	2020	Clinical Oral Investigations
Vineet A., Nimisha S.	Application of caries assessment spectrum and treatment instrument for staging and evaluating treatment needs of an adult population-A hospital based cross-sectional study	2021	African Health Sciences
Handa K.; Koike T.; Hayashi K.; Saito T.	Application of high-frequency radio waves to direct pulp capping	2013	Journal of Endodontics
Erbudak HÖ, Ozbek M, Uysal S, Karabulut E.	Application of Kvaal et al.'s age estimation method to panoramic radiographs from Turkish individuals	2012	Forensic Sci Int
Davoudi A.; Sanei M.; Badrian H.	Application of laser irradiation for restorative treatments	2016	Open Dentistry Journal
Kondo H.; Tobita T.; Ueno T.; Yoshimura H.; Katase N.; Ohba S.; Nagatsuka H.; Sano K.	Application of multiplanar computed tomography and the osirix imaging software for precise analysis of dens invaginatus in the maxillary third molar	2012	Journal of Hard Tissue Biology
Ding J, Ebihara A, Watanabe S, Iino Y, Kokuzawa C, Anjo T, Suda H, Sumi Y.	Application of optical coherence tomography to identify pulp exposure during access cavity preparation using an Er:YAG laser	2014	Photomed Laser Surg
Xi X.; Liu L.; Chen W.; Han G.	Application of platelet-rich plasma in regenerated pulp treatment of young permanent teeth: An animal study	2020	Indian Journal of Pharmaceutical Sciences
Ran L., Zhao N., Fan L., Zhou P., Zhang C., Yu C.	Application of virtual reality on non-drug behavioral management of short-term dental procedure in children	2021	Trials
Shetty C., Shetty A., Shetty S., Kaur G., Hegde M.N., Nidhi L.	Applications of chitosan in dentistry	2020	Indian Journal of Public Health Research and Development
Shobhana R., Karthick A., Mitthra S., Anuradha B.	Applications of laser in the field of endodontics: An update	2020	European Journal of Molecular and Clinical Medicine
Najeeb S, Khurshid Z, Zafar MS, Ajlal S.	Applications of Light Amplification by Stimulated Emission of Radiation (Lasers) for Restorative Dentistry	2016	Med Princ Pract
Aldhilan S.; Ali S.A.-H.	Approaches used to care for carious primary molars among pediatric dentists and general dental practitioners in Saudi Arabia	2018	Journal of Clinical and Experimental Dentistry
Waterhouse P.	Are different pulp treatment techniques and associated medicaments effective for the treatment of extensive decay in primary teeth?	2021	Evid Based Dent

Lu P.-C.; Wu J.-H.; Chen C.-M.; Du J.-K.	Arsenic trioxide-induced mandibular osteomyelitis	2015	Journal of Oral and Maxillofacial Surgery
Pereira L.A.P.; Groppo F.C.; Bergamaschi C.D.C.; Meechan J.G.; Ramacciato J.C.; Motta R.H.L.; Ranali J.	Articaine (4%) with epinephrine (1:100,000 or 1:200,000) in intraosseous injections in symptomatic irreversible pulpitis of mandibular molars: Anesthetic efficacy and cardiovascular effects	2013	Oral Surgery, Oral Medicine, Oral Pathology and Oral Radiology
Zheng L, Wang H, Mei L, Chen Q, Zhang Y, Zhang H.	Artificial intelligence in digital cariology: a new tool for the diagnosis of deep caries and pulpitis using convolutional neural networks	2021	Ann Transl Med
Bei Y.; Tianqian H.; Fanyuan Y.; Haiyun L.; Xueyang L.; Jing Y.; Chenglin W.; Ling Y.	ASH1L Suppresses Matrix Metalloproteinase through Mitogen-activated Protein Kinase Signaling Pathway in Pulpitis	2017	Journal of Endodontics
Cantekin K, Yildirim MD, Cantekin I.	Assessing change in quality of life and dental anxiety in young children following dental rehabilitation under general anesthesia	2014	Pediatr Dent
Mishra S.; Sharma D.S.; Bhusari C.	Assessing inflammatory status of pulp in irreversible pulpitis cases with pulse oximeter and dental hemogram	2019	Journal of Clinical Pediatric Dentistry
Gudipaneni RK, Alkuwaykibi AS, Ganji KK, Bandela V, Karobari MI, Hsiao CY, Kulkarni S, Thambar S.	Assessment of caries diagnostic thresholds of DMFT, ICDAS II and CAST in the estimation of caries prevalence rate in first permanent molars in early permanent dentition-a cross-sectional study	2022	BMC Oral Health
Parthasarathy P., Dharman S.	Assessment of dental patterns in digital panoramic radiographs-an aid in personal identification	2020	International Journal of Pharmaceutical Research
Almasri M.	Assessment of extracting molars and premolars after root canal treatment: A retrospective study	2019	Saudi Dental Journal
Taha N.A., Ahmad M.B., Ghanim A.	Assessment of Mineral Trioxide Aggregate pulpotomy in mature permanent teeth with carious exposures	2017	International endodontic journal
Kritikou K.; Imre M.; Tanase M.; Vinereanu A.; Totan A.R.; Spinu T.-C.; Miricescu D.; Stanescu-Spinu I.-I.; Bordea M.; Greabu M.	Assessment of Mineralization, Oxidative Stress, and Inflammation Mechanisms in the Pulp of Primary Teeth	2022	Applied Sciences (Switzerland)
Reis T.D.C.; Bortolotti F.; Innocentini L.M.A.R.; Ferrari T.C.; Ricz H.M.A.; Cunha R.L.G.; Costa T.C.D.M.; Macedo L.D.D.	Assessment of oral health condition in recipients of allogeneic hematopoietic cell transplantation	2022	Hematology, Transfusion and Cell Therapy
Assiri K.I., Sandeepa N.C., Asiri R.S., Mulawi S.A., Najmi S.M., Srivastava K.C.	Assessment of Oral-Systemic Disease Association amongst Dental Patients: A Retrospective Panoramic Radiographic Study	2020	The journal of contemporary dental practice
Abdelwahed A., Roshdy N.N.	Assessment of Post-operative Pain after using EdgeFile X7 and Protaper Next Rotary Systems in Patients with Symptomatic Pulpitis in Mandibular Molars: A Randomized Controlled Clinical Trial	2022	Open Access Macedonian Journal of Medical Sciences
Anusha B.; Madhusudhana K.; Chinni S.K.; Paramesh Y.	Assessment of pulp oxygen saturation levels by pulse oximetry for pulpal diseases -a diagnostic study	2017	Journal of Clinical and Diagnostic Research
Iohara K.; Fujita M.; Ariji Y.; Yoshikawa M.; Watanabe H.; Takashima A.; Nakashima M.	Assessment of pulp regeneration induced by stem cell therapy by magnetic resonance imaging	2016	Journal of Endodontics
Mittal N.; Baranwal H.C.; Kumar P.; Gupta S.	Assessment of pulp sensibility in the mature necrotic teeth using regenerative endodontic therapy with various scaffolds - Randomised clinical trial	2021	Indian Journal of Dental Research
Igna A.; Igna C.; Miron M.I.; Schuszler L.; Dascălu R.; Moldovan M.; Voicu A.A.; Todea C.D.; Boariu M.; Mârțu M.-A.; Stratul Ș.-I.	Assessment of Pulpal Status in Primary Teeth Following Direct Pulp Capping in an Experimental Canine Model	2022	Diagnostics
Abdullah M.M., Mohsen C.	Assessment of Retention and Marginal Accuracy Of Endocrown Retained Fixed Partial Dentures	2022	NeuroQuantology
Janani K.; Teja K.V.; Ajitha P.; Sandhya R.	Assessment of sCD14 levels in patients with endodontic pathology requiring root canal treatment; Avaliação dos níveis de sCD14 em pacientes que necessitam de tratamento endodôntico	2022	Brazilian Dental Science
Nino-Barrera JL, Ardila E, Guaman-Pacheco F, Gamboa-Martinez L, Alzate-Mendoza D.	Assessment of the relationship between the maxillary sinus floor and the upper posterior root tips: Clinical considerations	2018	J Investig Clin Dent

Meghana Reddy J., Gurunathan D., Rajendran D.	Assessment of working length of root canal in mandibular first primary molar in children aged between 2 to 6 years	2020	Indian Journal of Forensic Medicine and Toxicology
Bekes K, Steuber A, Challakh N, Schmidt J, Haak R, Hraský V, Ziebolz D.	Associated factors to caries experience of children undergoing general anaesthesia and treatment needs characteristics over a 10 year period	2020	BMC Oral Health
Prado IA, Costa CM, Costa SA, Carmo CDSD, Thomaz EBAF, Souza SFC, Ribeiro CCC.	Association Between Anaemia and Dental Caries in Brazilian Adolescents	2020	Oral Health Prev Dent
Sharma R.; Kumar V.; Logani A.; Chawla A.; Mir R.A.; Sharma S.; Kalaivani M.	Association between concentration of active MMP-9 in pulpal blood and pulpotomy outcome in permanent mature teeth with irreversible pulpitis – a preliminary study	2021	International Endodontic Journal
Kuligowski P., Jaroń A., Preuss O., Gabrysz-Trybek E., Bladowska J., Trybek G.	Association between odontogenic and maxillary sinus conditions: A retrospective cone-beam computed tomographic study	2021	Journal of Clinical Medicine
Vitali FC, Cardoso IV, Mello FW, Flores-Mir C, Andrada AC, Dutra-Horstmann KL, Duque TM.	Association between Orthodontic Force and Dental Pulp Changes: A Systematic Review of Clinical and Radiographic Outcomes	2022	J Endod
Nissrin B, Basma R, Majid S.	Association between Periodontitis and Pulp Calcifications: Radiological Study	2022	Int J Dent
Franciscatto G.J.; Brennan D.S.; Gomes M.S.; Rossi-Fedele G.	Association between pulp and periapical conditions and dental emergency visits involving pain relief: epidemiological profile and risk indicators in private practice in Australia	2020	International Endodontic Journal
Neves ÉTB, Perazzo MF, Gomes MC, Ribeiro ILA, Paiva SM, Granville-Garcia AF.	Association between sense of coherence and untreated dental caries in preschoolers: a cross-sectional study	2019	Int Dent J
Kawamura K, Doi T, Kano K, Matsui M, Hattori Y, Onishi F, Fukata H, Miyake T.	Association between smoking habits and dental care utilization and cost using administrative claims database and specific medical check-up data	2022	BMC Oral Health
Gaêta-Araujo H, Fontenele RC, Nascimento EHL, Nascimento MDCC, Freitas DQ, de Oliveira-Santos C.	Association between the Root Canal Configuration, Endodontic Treatment Technical Errors, and Periapical Hypodensities in Molar Teeth: A Cone-beam Computed Tomographic Study	2019	J Endod
Santhanam P., Sharma S., Sandhya R.	Association of age and gender distribution in patient having class III/ class IV caries resulting in root canal treatment in upper and lower anteriors	2020	European Journal of Molecular and Clinical Medicine
Ashwin Kumar S.P., Sugumaran S., Dharman S.	Association of age and gender in patients with class v caries indicated for root canal treatment in the age group 60-80 years	2020	International Journal of Pharmaceutical Research
Rajawat A.; Majeti C.; Podugu U.; Kaushik M.; Nagamaheshwari X.; Mehra N.	Association of hormonal fingerprints and dental caries: A pilot study	2020	Journal of Conservative Dentistry
Shivanni S.S.; Anjaneyulu K.; Balakrishna R.N.	Association of irreversible pulpitis and single visit root canal treatment	2020	International Journal of Dentistry and Oral Science
An G.K., Morse D.E., Kunin M., Goldberger R.S., Psoter W.J.	Association of Radiographically Diagnosed Apical Periodontitis and Cardiovascular Disease: A Hospital Records-based Study	2016	Journal of endodontics
Maqbool M, Noorani TY, Samsudin NA, Awang Nawi MA, Rossi-Fedele G, Karobari MI, Messina P, Scardina GA.	Association of Vital Pulp Therapy Outcomes with Tooth Type, Arch Location, Treatment Type, and Number of Surfaces Destroyed in Deciduous Teeth: A Retrospective Study	2021	Int J Environ Res Public Health
Erdogan O.; Malek M.; Gibbs J.L.	Associations between Pain Severity, Clinical Findings, and Endodontic Disease: A Cross-Sectional Study	2021	Journal of Endodontics
Wu H.; He M.; Yang R.; Zuo Y.; Bian Z.	Astrocyte elevated gene-1 participates in the production of pro-inflammatory cytokines in dental pulp cells via NF-κB signalling pathway	2018	International Endodontic Journal
Ren H.; Wen Q.; Zhao Q.; Wang N.; Zhao Y.	Atlas of human dental pulp cells at multiple spatial and temporal levels based on single-cell sequencing analysis	2022	Frontiers in Physiology
Leal S, Bonifacio C, Raggio D, Frencken J.	Atraumatic Restorative Treatment: Restorative Component	2018	Monogr Oral Sci

Shashmurina V.R., Nikolaev A.I., Vasiltsova O.A., Dmitriev M.V., Gladarevskaya E.I., Shashmurina A.B., Tyurin S.M.	Attitude to antibiotic therapy in dentists conducting therapeutic treatment of patients	2022	Kliniceskaa Mikrobiologia i Antimikrobnaa Himioterapia
Schwendicke F, Meyer-Lueckel H, Dörfer C, Paris S.	Attitudes and behaviour regarding deep dentin caries removal: a survey among German dentists	2013	Caries Res
Khaloufi O.; Khalaf L.H.; Akerzoul N.; Hassani F.Z.I.M.; Toure B.	Attitudes of dental practitioners from Northern Morocco on the prescription of antibiotics during endodontic treatment: A survey	2022	Saudi Endodontic Journal
Huang Y.; Tang X.; Cehreli Z.C.; Dai X.; Xu J.; Zhu H.	Autologous transplantation of deciduous tooth pulp into necrotic young permanent teeth for pulp regeneration in a dog model	2019	Journal of International Medical Research
Srinisha M.; Ramakrishnan M.	Average time in between appointments for multivisit pulpectomy in patients reported to a university dental hospital	2022	Journal of Advanced Pharmaceutical Technology and Research
Yuvaraj S., Ganapathy D.	Awareness about primary teeth and their care among general dentists a survey	2020	International Journal of Research in Pharmaceutical Sciences
Chandukutty D., Peedikayil F.C., Premkumar C.T., Narasimhan D., Jose D.	Awareness of dental trauma management among school teachers of Kannur, Kerala, India	2017	Journal of Clinical and Diagnostic Research
Azima Hanin S.M.; Muralidoss H.	Awareness of hot tooth and its management: A questionnaire survey	2021	International Journal of Dentistry and Oral Science
Pooja Umaiyal. M.	Awareness of root canal treatment among people	2016	Research Journal of Pharmacy and Technology
Endo M.S.; Cardoso E.R.; Pavan N.N.O.; de Morais C.A.H.; Martinho F.C.	Bacterial diversity in primary and secondary/persistent endodontic infections by Checkerboard DNA-DNA Hybridization technique	2017	Dental Press Endodontics
Beltrame A.P.; Bolan M.; Serratine A.C.; Rocha M.J.	Bacterial intensity and localization in primary molars with caries disease	2012	Journal of Indian Society of Pedodontics and Preventive Dentistry
Ricucci D, Siqueira JF Jr, Abdelsayed RA, Lio SG, Rôças IN.	Bacterial Invasion of Pulp Blood Vessels in Teeth with Symptomatic Irreversible Pulpitis	2021	J Endod
Afkhami F.; Nasri S.; Valizadeh S.	Bacterial leakage assessment in root canals sealed with AH Plus sealer modified with silver nanoparticles	2021	BMC Oral Health
Snigdha N.T., Kamarudin A.	BACTERIAL LEAKAGE OF BIOCERAMICS PULP DRESSING MATERIALS: A REVIEW OF LITERATURE	2022	Journal of Pharmaceutical Negative Results
Caneppele T.M.F.; de Souza L.G.; Spinola M.S.; de Oliveira F.E.; de Oliveira L.D.; Carvalho C.A.T.; Bresciani E.	Bacterial levels and amount of endotoxins in carious dentin within reversible pulpitis scenarios	2021	Clinical Oral Investigations
Dai S.; Xiao G.; Dong N.; Liu F.; He S.; Guo Q.	Bactericidal effect of a diode laser on Enterococcus faecalis in human primary teeth-an in vitro study	2018	BMC Oral Health
Osman A.; Gnanasegaran N.; Govindasamy V.; Kathivaloo P.; Wen A.S.; Musa S.; Abu Kasim N.H.	Basal expression of growth-factor-associated genes in periodontal ligament stem cells reveals multiple distinctive pathways	2014	International Endodontic Journal
Kwon Y.S.; Lee S.H.; Hwang Y.C.; Rosa V.; Lee K.W.; Min K.S.	Behaviour of human dental pulp cells cultured in a collagen hydrogel scaffold cross-linked with cinnamaldehyde	2017	International Endodontic Journal
Song J.; Wu Q.; Jiang J.; Sun D.; Wang F.; Xin B.; Cui Q.	Berberine reduces inflammation of human dental pulp fibroblast via miR-21/KBTBD7 axis	2020	Archives of Oral Biology
Duggal M, Gizani S, Albadri S, Krämer N, Stratigaki E, Tong HJ, Seremidi K, Kloukos D, BaniHani A, Santamaría RM, Hu S, Maden M, Amend S, Boutsiouki C, Bekes K, Lygidakis N, Frankenberger R, Monteiro J, Anttonnen V, Leith R, Sobczak M, Rajasekharan S, Parekh S.	Best clinical practice guidance for treating deep carious lesions in primary teeth: an EAPD policy document	2022	Eur Arch Paediatr Dent
Paglia L.	Bibliometrics to improve our patients' care!	2022	Eur J Paediatr Dent
Li J., Li Y., Xie J., Geng W., Gao X., Wang N., Zhang Y.	Bilateral mandibular second molar impaction with paradental cyst: A case report and literature review	2017	Journal of Jilin University Medicine Edition

Simila H.O.; Karpukhina N.; Hill R.G.	Bioactivity and fluoride release of strontium and fluoride modified Biodentine	2018	Dental Materials
Koch K.A.; Brave D.G.	Bioceramics, part I: the clinician's viewpoint.	2012	Dentistry today
Masthan K.M.K.; Aravindha Babu N.; Bhattacharjee T.; Elumalai Dr. M.	Biochemical markers - A tool to detect oral diseases	2013	International Journal of Pharma and Bio Sciences
Guerrero-Gironés J.; Alcaina-Lorente A.; Ortiz-Ruiz C.; Ortiz-Ruiz E.; Pecci-Lloret M.P.; Ortiz-Ruiz A.J.; Rodríguez-Lozano F.J.; Pecci-Lloret M.R.	Biocompatibility of a ha/β-tcp/c scaffoldas a pulp-capping agent for vital pulp treatment: An in vivo study in rat molars	2021	International Journal of Environmental Research and Public Health
Alsubait S, Aljarbou F.	Biodentine or Mineral Trioxide Aggregate as Direct Pulp Capping Material in Mature Permanent Teeth with Carious Exposure? A Systematic Review and Meta-analysis	2021	Oper Dent
Soni H.K.	Biodentine pulpotomy in mature permanent molar: A case report	2016	Journal of Clinical and Diagnostic Research
El Karim I.A., McCrudden M.T., McGahon M.K., Curtis T.M., Jeanneau C., Giraud T., Irwin C.R., Linden G.J., Lundy F.T., About I.	Biodentine Reduces Tumor Necrosis Factor Alpha-induced TRPA1 Expression in Odontoblastlike Cells	2016	Journal of endodontics
Shayegan A.; Jurysta C.; Atash R.; Petein M.; Abbeele A.V.	Biodentine used as a pulp-capping agent in primary pig teeth	2012	Pediatric Dentistry
Chinadet W, Sutharaphan T, Chompu-Inwai P.	Biodentine™ Partial Pulpotomy of a Young Permanent Molar with Signs and Symptoms Indicative of Irreversible Pulpitis and Periapical Lesion: A Case Report of a Five-Year Follow-Up	2019	Case Rep Dent
Nasrallah H.; Noueiri B.E.	Biodentine™ Pulpotomy in Stage I Primary Molars: A 12-month Follow-up	2022	International Journal of Clinical Pediatric Dentistry
Kale Y.; Yadav S.; Dadpe M.; Dahake P.; Kendre S.	Bioinductive and anti-inflammatory properties of Propolis and Biodentine on SHED	2022	Saudi Dental Journal
Manhas M.; Mittal S.; Sharma A.; Gupta K.; Pathania V.; Thakur V.	Biological approach in repair of partially inflamed dental pulp using second-generation platelet-rich fibrin and mineral trioxide aggregate as a pulp medicament in primary molars	2019	Journal of Indian Society of Pedodontics and Preventive Dentistry
Ziauddin S.M.; Nakashima M.; Watanabe H.; Tominaga M.; Iohara K.	Biological characteristics and pulp regeneration potential of stem cells from canine deciduous teeth compared with those of permanent teeth	2022	Stem Cell Research and Therapy
Ma L.; Li M.-W.; Bai Y.; Guo H.-H.; Wang S.-C.; Yu Q.	Biological characteristics of fluorescent superparamagnetic iron oxide labeled human dental pulp stem cells	2017	Stem Cells International
Paschalidou M.; Athanasiadou E.; Arapostathis K.; Kotsanos N.; Koidis P.T.; Bakopoulou A.; Theocharidou A.	Biological effects of low-level laser irradiation (LLLI) on stem cells from human exfoliated deciduous teeth (SHED)	2020	Clinical Oral Investigations
Lee M.; Kang C.-M.; Song J.S.; Shin Y.S.; Kim S.Y.; Kim S.-O.; Choi H.-J.	Biological efficacy of two mineral trioxide aggregate (Mta)-based materials in a canine model of pulpotomy	2017	Dental Materials Journal
Oliveira L.V.; de Souza G.L.; da Silva G.R.; Magalhães T.E.A.; Freitas G.A.N.; Turrioni A.P.; de Rezende Barbosa G.L.; Moura C.C.G.	Biological parameters, discolouration and radiopacity of calcium silicate-based materials in a simulated model of partial pulpotomy	2021	International Endodontic Journal
Zhu N., Chatzistavrou X., Ge L., Qin M., Papagerakis P., Wang Y.	Biological properties of modified bioactive glass on dental pulp cells	2019	Journal of dentistry
Rodrigues N.S.; França C.M.; Tahayeri A.; Ren Z.; Saboia V.P.A.; Smith A.J.; Ferracane J.L.; Koo H.; Bertassoni L.E.	Biomaterial and Biofilm Interactions with the Pulp-Dentin Complex-on-a-Chip	2021	Journal of Dental Research
Wang X., Zhang Y., Li X., Huang Z., Cui M., Huang Z., Zhang X., Hu X.	Biometric analysis of apical surgery-related anatomy of mandibular first molars: a cone-beam computed tomography study in a Mongoloid population	2019	Journal of International Medical Research
Chen Z, Cao S, Wang H, Li Y, Kishen A, Deng X, Yang X, Wang Y, Cong C, Wang H, Zhang X.	Biomimetic remineralization of demineralized dentine using scaffold of CMC/ACP nanocomplexes in an in vitro tooth model of deep caries	2015	PLoS One
Rodríguez-Lozano F.J.; Lozano A.; López-García S.; García-Bernal D.; Sanz J.L.; Guerrero-Gironés J.; Llena C.; Forner L.; Melo M.	Biomineralization potential and biological properties of a new tantalum oxide (Ta2O5)–containing calcium silicate cement	2022	Clinical Oral Investigations
Kobayashi K., Koyama N., Nagano Y., Usami I., Fujimura K., Heike T.	Biopsychosocial assessment of dental neglect in a pediatric cancer patient from the perspectives of developmental trauma disorder	2022	Pediatric Dental Journal

Assiry A.	Bi-rooted primary maxillary canines: A case report	2019	Journal of Medical Case Reports
Shmueli A, Guelmann M, Tickotsky N, Ninio-Harush R, Noy AF, Moskovitz M.	Blood Gas Tension and Acidity Level of Caries Exposed Vital Pulps in Primary Molars	2020	J Clin Pediatr Dent
Huang J.I.-S.; Chang H.-H.; Liao W.-C.; Lin C.-P.; Kao C.-T.; Huang T.-H.	Blood pressure reduction in patients with irreversible pulpitis teeth treated by non-surgical root canal treatment	2017	Journal of Dental Sciences
Mohamad S.A.; Milward M.R.; Hadis M.A.; Kuehne S.A.; Cooper P.R.	Blue light photobiomodulation of dental pulp cells	2022	Lasers in Dental Science
Lopes G.C.; Ballarin A.; Baratieri L.N.	Bond strength and fracture analysis between resin cements and root canal dentin	2012	Australian Endodontic Journal
Dell'Olio F., Lorusso P., Barile G., Favia G.	Brugada Syndrome Updated Risk Assessment and Perioperative Management in Oral Surgery: A Case Series	2021	Journal of Oral and Maxillofacial Surgery
Bilgin M.S.; Erdem A.; Tanriver M.	CAD/CAM endocrown fabrication from a polymer-infiltrated ceramic network block for primary molar: A case report	2016	Journal of Clinical Pediatric Dentistry
Kuramoto H.; Hirao K.; Yumoto H.; Hosokawa Y.; Nakanishi T.; Takegawa D.; Washio A.; Kitamura C.; Matsuo T.	Caffeic Acid Phenethyl Ester (CAPE) Induces VEGF Expression and Production in Rat Odontoblastic Cells	2019	BioMed Research International
Kuramoto H.; Nakanishi T.; Takegawa D.; Mieda K.; Hosaka K.	Caffeic Acid Phenethyl Ester Induces Vascular Endothelial Growth Factor Production and Inhibits CXCL10 Production in Human Dental Pulp Cells	2022	Current issues in molecular biology
Tavassoli-Hojjati S.; Kameli S.; Rahimian-Emam S.; Ahmadyar M.; Asgary S.	Calcium enriched mixture cement for primary molars exhibiting root perforations and extensive root resorption: report of three cases	2014	Pediatric dentistry
Prasetyo E.P.; Kuntjoro M.; Goenharto S.; Juniarti D.E.; Cahyani F.; Hendrijantini N.; Nugraha A.P.; Hariyani N.; Rantam F.A.	Calcium hydroxide increases human umbilical cord mesenchymal stem cells expressions of apoptotic protease-activating factor-1, caspase-3 and caspase-9	2021	Clinical, Cosmetic and Investigational Dentistry
ElSebaai A, Wahba AH, Grawish ME, Elkalla IH.	Calcium Hydroxide Paste, Mineral Trioxide Aggregate, and Formocresol as Direct Pulp Capping Agents in Primary Molars: A Randomized Controlled Clinical Trial	2022	Pediatr Dent
Schwendicke F., Brouwer F., Stolpe M.	Calcium Hydroxide versus Mineral Trioxide Aggregate for Direct Pulp Capping: A Cost-effectiveness Analysis	2015	Journal of endodontics
Huang H.; Luo L.; Li L.; Guan Y.; Yan Y.; Jiang Z.; Jiang B.	Calcium Phosphate Cement Promotes Odontoblastic Differentiation of Dental Pulp Cells In Vitro and In Vivo	2022	Coatings
Gandolfi M.G.; Spagnuolo G.; Siboni F.; Procino A.; Rivieccio V.; Pelliccioni G.A.; Prati C.; Rengo S.	Calcium silicate/calcium phosphate biphasic cements for vital pulp therapy: chemical-physical properties and human pulp cells response	2015	Clinical Oral Investigations
Lee J.-W., Lee E.-S., Kim B.-I.	Can red fluorescence be useful in diagnostic decision making of residual dentin caries?	2019	Photodiagnosis and Photodynamic Therapy
Cancio V., Carvalho Ferreira D., Cavalcante F.S., Rosado A.S., Teixeira L.M., Braga Oliveira Q., Barcelos R., Gleiser R., Santos H.F., Dos Santos K.R.N., Primo L.G.	Can the Enterococcus faecalis identified in the root canals of primary teeth be a cause of failure of endodontic treatment?	2017	Acta odontologica Scandinavica
Naseri M, Kharazifard MJ, Hosseinpour S.	Canal Configuration of Mesiobuccal Roots in Permanent Maxillary First Molars in Iranian Population: A Systematic Review	2016	J Dent (Tehran)
Saberi E.A., Mollashahi N.F., Farahi F.	Canal transportation caused by one single-file and two multiple-file rotary systems: A comparative study using cone-beam computed tomography	2018	Giornale Italiano di Endodonzia
Kramer P.R.; Woodmansey K.F.; White R.; Primus C.M.; Opperman L.A.	Capping a pulpotomy with calcium aluminosilicate cement: comparison to mineral trioxide aggregates	2014	Journal of endodontics
Kundzina R., Stangvaltaite L., Eriksen H.M., Kerosuo E.	Capping carious exposures in adults: a randomized controlled trial investigating mineral trioxide aggregate versus calcium hydroxide	2017	International endodontic journal
Ghaderi F, Jowkar Z, Tadayon A.	Caries Color, Extent, and Preoperative Pain as Predictors of Pulp Status in Primary Teeth	2020	Clin Cosmet Investig Dent
Habib F, Chaly PE, Junaid M, Musthafa HM.	Caries experience, clinical consequences of untreated dental caries and associated factors among school going children - A cross-sectional study	2020	Indian J Dent Res
Santamaria R.M.; Innes N.P.T.; Machiulskiene V.; Evans D.J.P.; Splieth C.H.	Caries management strategies for primary molars: 1-yr randomized control trial results	2014	Journal of Dental Research

Aïem E, Joseph C, Garcia A, Smaïl-Faugeron V, Muller-Bolla M.	Caries removal strategies for deep carious lesions in primary teeth: Systematic review	2020	Int J Paediatr Dent
Zhou W., Niu L.-N., Hu L., Jiao K., Chang G., Shen L.-J., Tay F.R., Chen J.-H.	Caries-resistant bonding layer in dentin	2016	Scientific reports
Çelik B.N.; Sari S.	Carious Exposure versus Mechanical Exposure for MTA Pulpotomy in Primary Teeth	2016	BioMed Research International
Pavlič A, Vrecl M, Jan J, Bizjak M, Nemec A.	Case report of a molar-root incisor malformation in a patient with an autoimmune lymphoproliferative syndrome	2019	BMC Oral Health
da Mota A.C.C.; Leal C.R.L.; Olivan S.; Gonçalves M.L.L.; de Oliveira V.A.; Pinto M.M.; Bussadori S.K.	Case report of photodynamic therapy in the treatment of dental caries on primary teeth	2016	Journal of Lasers in Medical Sciences
Nagi P., Waly N., Elbardissy A., Khalifa M.	Case report: Immediate pain relief after partial pulpotomy of cariously exposed young permanent molar using mineral trioxide aggregate and root maturation, with two years follow-up.	2018	F1000Research
Azab M.M., Moheb D.M., El Shahawy O.I.	Case report: Root resorption caused after pulp death of adjacent primary molar.	2018	F1000Research
Zhu L, Cao X, Tao XF.	Case-based learning based on CBCT software in clinical teaching of oral and maxillofacial radiology	2017	Shanghai Kou Qiang Yi Xue
Topçu K.; Kirici D.; Evcil M.	Catalase activity in healthy and inflamed pulp tissues of permanent teeth in young people	2016	Nigerian Journal of Clinical Practice
Eigbobo J.O.; Gbujie D.C.; Onyeaso C.O.	Causes and pattern of tooth extractions in children treated at the University of Port Harcourt Teaching Hospital.	2014	Odonto-stomatologie tropicale = Tropical dental journal
Olatosi O.O.; Sote E.O.	Causes and pattern of tooth loss in children and adolescents in a Nigerian tertiary hospital.	2012	Nigerian quarterly journal of hospital medicine
Kalender A.; Oztan M.D.; Basmaci F.; Aksoy U.; Orhan K.	CBCT evaluation of multiple idiopathic internal resorptions in permanent molars: Case report	2014	BMC Oral Health
Wang L, Li JP, Ge ZP, Li G.	CBCT image based segmentation method for tooth pulp cavity region extraction	2019	Dentomaxillofac Radiol
De Almeida F.J.M.; Hassan D.; Abdulrahman G.N.; Brundin M.; Vestman N.R.	Cbct influences endodontic therapeutic decision-making in immature traumatized teeth with suspected pulp necrosis: A before-after study	2021	Dentomaxillofacial Radiology
Matsui M.; Kobayashi T.; Tsutsui T.W.	CD146 positive human dental pulp stem cells promote regeneration of dentin/pulp-like structures	2018	Human Cell
Marques N.C.T.; Neto N.L.; Prado M.T.O.; Vitor L.L.R.; Rodini C.O.; Sakai V.T.; Machado M.A.A.M.; Santos C.F.; Oliveira T.M.	CD31 expression in human primary teeth treated with photobiomodulation therapy	2018	Lasers in Dental Science
Galler K.M.; Eidt A.; Schmalz G.	Cell-free approaches for dental pulp tissue engineering	2014	Journal of Endodontics
Rosa JT, Witten PE, Huysseune A.	Cells at the Edge: The Dentin-Bone Interface in Zebrafish Teeth	2021	Front Physiol
Victoria-Escandell A.; Ibañez-Cabellos J.S.; De Cutanda S.B.-S.; Berenguer-Pascual E.; Beltrán-García J.; García-López E.; Pallardél F.V.; García-Giménez J.L.; Pallarés-Sabater A.; Zarzosa-López I.; Monterde M.	Cellular responses in human dental pulp stem cells treated with three endodontic materials	2017	Stem Cells International
Bajaj N.; Madan N.; Rathnam A.	Cessation in root development: Ramifications of 'Stevens-Johnson' syndrome	2012	Journal of Indian Society of Pedodontics and Preventive Dentistry
Moore E.R.; Michot B.; Erdogan O.; Ba A.; Gibbs J.L.; Yang Y.	CGRP and Shh Mediate the Dental Pulp Cell Response to Neuron Stimulation	2022	Journal of Dental Research
Dursun E, Monnier-Da Costa A, Moussally C.	Chairside CAD/CAM Composite Onlays for the Restoration Of Primary Molars	2018	J Clin Pediatr Dent
Xu R, Zhou C, Zhang Y, Zhang S, Xie J, Yuan Q.	Challenges of Stem-cell-based Craniofacial Regeneration	2021	Curr Stem Cell Res Ther
Álvarez-Quiceno D., Rojas-Martínez P.-A., Cruz-González A.-C.	Change of dental color and temperature through two bleaching agents boosted with light emitted by diodes	2021	Journal of Esthetic and Restorative Dentistry
Mesquita-Guimarães K.S.F.; De Rossi A.; Freitas A.C.; Nelson-Filho P.; Da Silva R.A.; De Queiroz A.M.	Changes in caries risk and activity of a 9-year-old patient with Niemann-Pick disease type C	2015	Case Reports in Dentistry

Santana M.D.R.; De Souza A.C.A.; De Assis E.V.; De Abreu L.C.; Valenti V.E.	Changes in heart period during endodontic treatment	2014	Experimental and Clinical Cardiology
Stangvaltaite-Mouhat L, Stankeviciene I, Brukiene V, Puriene A, Drachev SN.	Changes in Management Preference of Deep Carious Lesions and Exposed Pulps: Questionnaire Studies with a 10-Year Interval among Dentists in Lithuania	2022	Caries Res
Liu Q.; Gao Z.; Zhu X.; Wu Z.; Li D.; He H.; Huang F.; Fan W.	Changes in nitric oxide synthase isoforms in the trigeminal ganglion of rat following chronic tooth pulp inflammation	2016	Neuroscience Letters
Yang J.; Yang G.; Jin R.; Song G.; Yuan G.	Changes in paediatric dental clinic after reopening during COVID-19 pandemic in Wuhan: A retrospective study	2022	BMJ Open
Wu K.; Li C.; Yang Z.; Yang S.; Yang W.; Hua C.	Changes in the characteristics of dental emergencies under the influence of SARS-CoV-2 pandemic: a retrospective study	2021	BMC Oral Health
Esmaeili-Mahani S.; Raoof M.; Abbasnejad M.; Nourzadeh M.	Changes in the levels of hippocampal BDNF expression are accompanied with inflammatory dental pain-induced learning and memory impairment	2018	Physiology and Pharmacology (Iran)
Ricucci D, Loghin S, Niu LN, Tay FR.	Changes in the radicular pulp-dentine complex in healthy intact teeth and in response to deep caries or restorations: A histological and histobacteriological study	2018	J Dent
Seet R.F.; Chan P.Y.; Khoo S.-T.; Yu V.S.H.; Lui J.-N.	Characteristics of Cracked Teeth with Reversible Pulpitis After Orthodontic Banding—A Prospective Cohort Study	2022	Journal of Endodontics
Zhu Z.-y.; Zhou T.; Zhang B.-w.	Characterization and analysis of matrix metalloproteinases 8 and 20 in the human crown and root dentin	2012	Chinese Journal of Tissue Engineering Research
Le Clerc J.; Pérard M.; Pellen-Mussi P.; Novella A.; Tricot-Doleux S.; Jeanne S.; Pérez F.	Characterization of a programmed necrosis process in 3-dimensional cultures of dental pulp fibroblasts	2013	International Endodontic Journal
Edanami N.; Yoshiba N.; Ohkura N.; Takeuchi R.; Tohma A.; Noiri Y.; Yoshiba K.	Characterization of Dental Pulp Myofibroblasts in Rat Molars after Pulpotomy	2017	Journal of Endodontics
Hattori-Sanuki T.; Karakida T.; Chiba-Ohkuma R.; Miake Y.; Yamamoto R.; Yamakoshi Y.; Hosoya N.	Characterization of Living Dental Pulp Cells in Direct Contact with Mineral Trioxide Aggregate	2020	Cells
Torres A, Jacobs R, Lambrechts P, Brizuela C, Cabrera C, Concha G, Pedemonte ME.	Characterization of mandibular molar root and canal morphology using cone beam computed tomography and its variability in Belgian and Chilean population samples	2015	Imaging Sci Dent
Marin A.; Morales P.; Jiménez M.; Borja E.; Ivanovic-Zuvic D.; Collins M.T.; Florenzano P.	Characterization of Oral Health Status in Chilean Patients with X-Linked Hypophosphatemia	2021	Calcified Tissue International
Zahran S.; Witherden E.; Mannocci F.; Koller G.	Characterization of Root Canal Microbiota in Teeth Diagnosed with Irreversible Pulpitis	2021	Journal of Endodontics
Zayed M.; Iohara K.; Watanabe H.; Ishikawa M.; Tominaga M.; Nakashima M.	Characterization of stable hypoxia-preconditioned dental pulp stem cells compared with mobilized dental pulp stem cells for application for pulp regenerative therapy	2021	Stem Cell Research and Therapy
Mounir M.M.F.; Farsi J.M.A.; Alhazzazi T.Y.; Matar M.A.; El-Housseiny A.A.	Characterization of the apical bridge barrier formed following amelogenin apexification	2018	BMC Oral Health
Bussadori SK, Godoy CH, Alfaya TA, Fernandes KP, Mesquita-Ferrari RA, Motta LJ.	Chemo-mechanical caries removal with Papacarie™: case series with 84 reports and 12 months of follow-up	2014	J Contemp Dent Pract
Zambrano-Achig P, Viteri-García A, Verdugo-Paiva F.	Chemo-mechanical removal versus conventional removal for deep caries lesion	2022	Medwave
Gürcan A.T.; Bayram M.	Children's dental treatment requirements of first permanent molars with poor prognosis	2022	Clinical Oral Investigations
Sari D.S.; Sakinah N.; Nuri; Suswati E.; Widyowati R.; Maduratna E.	Chlorogenic Acid Fractionation in Robusta Green Bean Extract as a Combination Agent of Dental Pulp Stem Cells in Periodontal Tissue Engineering	2022	Research Journal of Pharmacy and Technology
Popovska L.; Zabokova Bilbilova E.; Mircheva E.; Stojanovska V.	Chronic periapical lesions: correlations with clinical finding	2014	Prilozi (Makedonska akademija na naukite i umetnostite. Oddelenie za medicinski nauki)
do Nascimento I.V.; Rodrigues M.I.D.Q.; Isaias P.H.C.; Barros-Silva P.G.; Sousa F.B.; Nunes Alves A.P.N.; Mota M.R.L.	Chronic systemic corticosteroid therapy influences the development of pulp necrosis and experimental apical periodontitis, exacerbating the inflammatory process and bone resorption in rats	2022	International Endodontic Journal

Worsley M.A.; Allen C.E.; Billinton A.; King A.E.; Boissonade F.M.	Chronic tooth pulp inflammation induces persistent expression of phosphorylated ERK (pERK) and phosphorylated p38 (pp38) in trigeminal subnucleus caudalis	2014	Neuroscience
Ge X.; Li Z.; Zhou Z.; Xia Y.; Bian M.; Yu J.	Circular RNA SIPA1L1 promotes osteogenesis via regulating the miR-617/Smad3 axis in dental pulp stem cells	2020	Stem Cell Research and Therapy
Shivanni S.S., Anjaneyulu K., Balakrishna R.N.	CLASS III DENTAL CARIES AND NEED FOR ROOT CANAL TREATMENT IN MAXILLARY ANTERIORS - A HOSPITAL BASED RETROSPECTIVE ANALYSIS	2022	Journal of Pharmaceutical Negative Results
Sarmast N.D.; Wang H.H.; Sajadi A.S.; Angelov N.; Dorn S.O.	Classification and Clinical Management of Retrograde Peri-implantitis Associated with Apical Periodontitis: A Proposed Classification System and Case Report	2017	Journal of Endodontics
Shah A, Lee D, Song M, Kim S, Kang MK, Kim RH.	Clastic cells are absent around the root surface in pulp-exposed periapical periodontitis lesions in mice	2018	Oral Dis
Basir L, Abdi R, Hashemi E.	Cleaning efficacy of various root canal irrigants in primary teeth: a scanning electron microscopic study	2022	Gen Dent
Dikopova N.Zh.; Volkov A.G.; Kopecky I.S.; Nikolskaya I.A.; Margaryan E.G.; Budina T.V.; Samokhlib Y.V.; Kondratiev S.A.; Paramonov Y.O.; Arakelyan M.G.	Clinical and experimental validation of the ozone therapy effectiveness in case of accidental exposure of the dental pulp	2021	New Armenian Medical Journal
Eftimoska M.; Apostolska S.; Rendzhova V.; Gjorgievska E.; Stevanovic M.; Ivanovski K.; Jankulovska M.; Elenchevski S.; Pavlevska M.; Dimkov A.	Clinical and histological analyzes of the response of the pulp after its direct capping with Calxyl, MTA and Biodentine	2015	Research Journal of Pharmaceutical, Biological and Chemical Sciences
Nowicka A.; Łagocka R.; Lipski M.; Parafiniuk M.; Grocholewicz K.; Sobolewska E.; Witek A.; Buczkowska-Radlińska J.	Clinical and Histological Evaluation of Direct Pulp Capping on Human Pulp Tissue Using a Dentin Adhesive System	2016	BioMed Research International
Mehrvarzfar P.; Abbott P.V.; Mashhadiabbas F.; Vatanpour M.; Tour Savadkouhi S.	Clinical and histological responses of human dental pulp to MTA and combined MTA/treated dentin matrix in partial pulpotomy	2018	Australian Endodontic Journal
Liu H, Peng X, Sun H, Li X.	Clinical and histopathological characterization of root resorption in replanted teeth: Two case reports	2020	Medicine (Baltimore)
Montag R, Dietz W, Nietzsche S, Lang T, Weich K, Sigusch BW, Gaengler P.	Clinical and Micromorphologic 29-year Results of Posterior Composite Restorations	2018	J Dent Res
Shaik D., Dadarwal M., Khan M.M., Dubey A., Sainudeen S., Banik A., Tiwari H.D.	Clinical and radiographic assessments of potassium nitrate in polycarboxylate versus mineral trioxide aggregate as pulpotomy biomaterials in immature mandibular first permanent molars- An original research	2022	European Journal of Molecular and Clinical Medicine
Kahvand M.; Mehran M.; Haghgoo R.; Faghihi T.	Clinical and radiographic evaluation of Allium sativum oil (garlic oil) in comparison with formocresol in primary molar pulpotomy	2019	Journal of International Society of Preventive and Community Dentistry
Caruso S., Dinoi T., Marzo G., Campanella V., Giuca M.R., Gatto R., Pasini M.	Clinical and radiographic evaluation of biodentine versus calcium hydroxide in primary teeth pulpotomies: a retrospective study	2018	BMC oral health
AbdelHamid M.B.; Elezz A.F.A.; Fahmy O.M.I.	Clinical and radiographic evaluation of diode laser and chemical disinfection in comparison to selective caries removal in management of patients with deep carious lesions	2022	Lasers in Dental Science
Ansari G.; Chitsazan A.; Fekrazad R.; Javadi F.	Clinical and radiographic evaluation of diode laser pulpotomy on human primary teeth	2018	Laser Therapy
Goel H., Mathur S., Sachdev V.	Clinical and radiographic evaluation of four different zinc-oxide integrated root canal obturating materials used in primary teeth	2018	Pediatric Dental Journal
Elchaghaby MA, Moheb DM, El Shahawy OI, Abd Alsamad AM, Rashed MAM.	Clinical and radiographic evaluation of indirect pulp treatment of young permanent molars using photo-activated oral disinfection versus calcium hydroxide: a randomized controlled pilot trial	2020	BDJ Open
Togaru H.; Muppa R.; Srinivas N.C.; Naveen K.; Reddy V.K.; Rebecca V.C.	Clinical and radiographic evaluation of success of two commercially available pulpotomy agents in primary teeth: An in vivo study	2016	Journal of Contemporary Dental Practice
Yildirim C, Basak F, Akgun OM, Polat GG, Altun C.	Clinical and Radiographic Evaluation of the Effectiveness of Formocresol, Mineral Trioxide Aggregate, Portland Cement, and Enamel Matrix Derivative in Primary Teeth Pulpotomies: A Two Year Follow-Up	2016	J Clin Pediatr Dent

Hemavathi, Nagarathna J, Srinath SK, Hiremath MC.	Clinical and radiographic evaluation of the efficacy of sodium hypochlorite as a haemostatic agent compared with physiologic saline on the success of calcium hydroxide pulpotomies in primary molars: an in vivo study	2018	Eur Arch Paediatr Dent
Frenkel G, Kaufman A, Ashkenazi M.	Clinical and radiographic outcomes of pulpotomized primary molars treated with white or gray mineral trioxide aggregate and ferric sulfate--long-term follow-up	2012	J Clin Pediatr Dent
Fernandes A.P.; Lourenço Neto N.; Teixeira Marques N.C.; Silveira Moretti A.B.; Sakai V.T.; Cruvinel Silva T.; Andrade Moreira Machado M.A.; Marchini Oliveira T.	Clinical and radiographic outcomes of the use of Low-Level Laser Therapy in vital pulp of primary teeth	2015	International Journal of Paediatric Dentistry
Saber A, El Meligy O, Alaki S, Felemban O.	Clinical and Radiographic Success of 2% Chlorhexidine Gluconate with Mineral Trioxide Aggregate in Indirect Pulp Treatment of Primary Molars	2022	J Dent Child (Chic)
Stafuzza TC, Vitor LLR, Rios D, Cruvinel Silva T, Machado MAAM, Oliveira TM.	Clinical and Radiographic Success of Selective Caries Removal to Firm Dentin in Primary Teeth: 18-Month Follow-Up	2018	Case Rep Dent
Ramazani N., Nezhad S.M.	Clinical and radiographic success rate of pulp treated primary molars restored with stainless steel crown (SSC) versus glass ionomer-SSC: A double-blind randomized clinical trial	2020	Iranian Journal of Pediatrics
Ulusoy A.T.; Bayrak S.; Bodrumlu E.H.	Clinical and radiological evaluation of calcium sulfate as direct pulp capping material in primary teeth	2014	European Journal of Paediatric Dentistry
Lili Y., Yan Z., Shijun Z., Shuai Z., Na W., Jie X., Shue H., Zhiyuan X.	Clinical application of cone beam computed tomography combined with micro-ultrasound technique in treating three mesial canals in mandibular first molars	2017	Hua xi kou qiang yi xue za zhi = Huaxi kouqiang yixue zazhi = West China journal of stomatology
Riggs GG, Arzi B, Cissell DD, Hatcher DC, Kass PH, Zhen A, Verstraete FJ.	Clinical Application of Cone-Beam Computed Tomography of the Rabbit Head: Part 1 - Normal Dentition	2016	Front Vet Sci
Purayil T.P., Kumar S.S., Upadhyay S.T., Acharya S.R., Anju P.K.	Clinical applications of glass fiber reinforced composites: A case series	2020	Indian Journal of Public Health Research and Development
Pietrzycka K, Pawlicka H.	Clinical aspects of pulp stones: A case report series	2020	Dent Med Probl
Karteva E.G.; Manchorova N.A.; Vladimirov S.B.; Keskinova D.A.	Clinical Assessment of Endodontically Treated Teeth, Restored with or without Radicular Posts	2018	Folia medica
Vural U.K.; Kiremitçi A.; Gökalp S.	Clinical assessment of mineral trioxide aggregate in the treatment of deep carious lesions	2017	Nigerian Journal of Clinical Practice
Heboyan A.G., Avetisyan A.A., Margaryan M.M.	Clinical case of a rarely diagnosed tooth root internal resorption	2018	New Armenian Medical Journal
Reddy S.P., Prasad M.G., Radhakrishna A.N., Sandeep R.V., Divya D.V., Kumar K.V.K.S.	Clinical comparison of eggshell derived calcium hydroxyapatite with dycal(®) as indirect pulp capping agents in primary molars	2020	Pesquisa Brasileira em Odontopediatria e Clinica Integrada
Ozdemir M, Unverdi GE, Geduk N, Ballikaya E, Cehreli ZC.	Clinical Comparison of Preformed Zirconia and Composite Strip Crowns in Primary Maxillary Incisors: 18-Month Results of a Prospective, Randomized Trial	2022	Pediatr Dent
Kotha S.B., Binhuwaishel H.A., Almuhaydib R.N., Alzeghaibi L.Y., Alhajri M.A.	Clinical decision-making in managing deep carious lesions in primary teeth based on clinical experience among pediatric dentists-A cross-sectional study	2022	Journal of population therapeutics and clinical pharmacology = Journal de la therapeutique des populations et de la pharmacologie clinique
Setzer F.C.; Kataoka S.H.H.; Natrielli F.; Gondim-Junior E.; Caldeira C.L.	Clinical diagnosis of pulp inflammation based on pulp oxygenation rates measured by pulse oximetry	2012	Journal of Endodontics
Wang L., Meng D.-J., Yang Y.-Z., Huang Y.-Q., Cao Q., Yao Z.-H., Tian L.-L.	CLINICAL EFFECT OF ULTRASOUND-COMBINED MINIMALLY INVASIVE ROOT CANAL SURGERY IN THE TREATMENT OF PULPITIS	2022	Acta Medica Mediterranea
Jordan R.A.; Holzner A.L.; Markovic L.; Brueckner I.; Zimmer S.	Clinical effectiveness of basic root canal treatment after 24 months: A randomized controlled trial	2014	Journal of Endodontics
Amend S, Boutsiouki C, Bekes K, Kloukos D, Gizani S, Lygidakis NN, Frankenberger R, Krämer N.	Clinical effectiveness of restorative materials for the restoration of carious lesions in pulp treated primary teeth: a systematic review	2022	Eur Arch Paediatr Dent
Amend S, Boutsiouki C, Bekes K, Kloukos D, Lygidakis NN, Frankenberger R, Krämer N.	Clinical effectiveness of restorative materials for the restoration of carious primary teeth without pulp therapy: a systematic review	2022	Eur Arch Paediatr Dent

Wang J, Chen Y, Zhang B, Ge X, Wang X.	Clinical efficacy of Er:YAG laser application in pulpotomy of primary molars: a 2-year follow-up study	2022	Lasers Med Sci
Heintze SD, Loguercio AD, Hanzen TA, Reis A, Rousson V.	Clinical efficacy of resin-based direct posterior restorations and glass-ionomer restorations - An updated meta-analysis of clinical outcome parameters	2022	Dent Mater
Kusumvalli S, Diwan A, Pasha S, Devale MR, Chowdhary CD, Saikia P.	Clinical evaluation of biodentine: Its efficacy in the management of deep dental caries	2019	Indian J Dent Res
Mohammadzadeh Z.; Parisay I.; Mehrabkhani M.; Madani A.S.; Mazhari F.	Clinical evaluation of fiber-reinforced composite crowns in pulp-treated primary molars: 12-month results	2016	European Journal of Dentistry
Hegde S.; Sowmya B.; Mathew S.; Bhandi S.H.; Nagaraja S.; Dinesh K.	Clinical evaluation of mineral trioxide aggregate and biodentine as direct pulp capping agents in carious teeth	2017	Journal of Conservative Dentistry
Niranjani K.; Prasad M.G.; Vasa A.A.; Divya G.; Thakur M.S.; Saujanya K.	Clinical evaluation of success of primary teeth pulpotomy using mineral trioxide aggregate®, laser and biodentine™-an in vivo study	2015	Journal of Clinical and Diagnostic Research
Phonghanyudh A, Phantumvanit P, Songpaisan Y, Petersen PE.	Clinical evaluation of three caries removal approaches in primary teeth: a randomised controlled trial	2012	Community Dent Health
Pach J, Regulski PA, Tomczyk J, Strużycka I.	Clinical implications of a diagnosis of taurodontism: A literature review	2022	Adv Clin Exp Med
Yu G., Ye L., Huang D.	Clinical investigation of radix entomolaris in mandibular first molars	2012	Hua xi kou qiang yi xue za zhi = Huaxi kouqiang yixue zazhi = West China journal of stomatology
Palanisamy V., Rao A., Rao A.	Clinical management of primary mandibular first molars with atypical morphology: Report of two cases	2016	Dental and Medical Problems
Palanisamy V.; Rao A.; Rao A.	Clinical management of primary mandibular first molars with atypical morphology: Report of two cases; Postępowanie kliniczne w dwóch przypadkach pierwszych mlecznych zębów trzonowych o nietypowej budowie	2016	Dental and Medical Problems
Guang J.; Li J.; Hao L.	Clinical observation and histopathological evaluation of pulp after pulpotomy of primary teeth with formocresol and biodentine	2022	Cellular and Molecular Biology
Kulkarni P, Tiwari S, Agrawal N, Kumar A, Umarekar P, Bhargava S.	Clinical Outcome of Direct Pulp Therapy in Primary Teeth: A Systematic Review and Meta-analysis	2022	J Indian Soc Pedod Prev Dent
Subbiya A.; Kishen A.; Pradeepkumar A.R.; Srinivasan N.; JothiLatha S.; Janani B.	Clinical Outcome of Nonsurgical Root Canal Treatment Using a Matched Single-Cone Obturation Technique with a Calcium Hydroxide–based Sealer: A Retrospective Analysis	2022	Journal of Endodontics
Schmalz G.; Widbiller M.; Galler K.M.	Clinical Perspectives of Pulp Regeneration	2020	Journal of Endodontics
Pandey S.R., Ranjan M.	Clinical practice guidelines in the management of open apex cases	2020	International Journal of Pharmaceutical Research
Galler KM.	Clinical procedures for revitalization: current knowledge and considerations	2016	Int Endod J
Chak R.K.; Singh R.K.; Mutyala J.; Killi N.K.	Clinical Radiographic Evaluation of 3Mixtatin and MTA in Primary Teeth Pulpotomies: A Randomized Controlled	2022	International Journal of Clinical Pediatric Dentistry
Brown J.A.; Murphy B.G.; Clapp K.S.; LaDouceur E.E.B.	Clinical, Diagnostic and Histological Findings Involving Cheek Teeth Hypercementosis in Nine Horses	2022	Journal of Veterinary Dentistry
Mittal M, Gupta N, Kumar A, Chopra R, Barua M.	Clinical, microbiological, and radiographic evaluation of sealed carious dentin after minimal intervention in primary molars	2020	J Indian Soc Pedod Prev Dent
Oliveira T.M.; Moretti A.B.S.; Sakai V.T.; Lourenço Neto N.; Santos C.F.; Machado M.A.A.M.; Abdo R.C.C.	Clinical, radiographic and histologic analysis of the effects of pulp capping materials used in pulpotomies of human primary teeth	2013	European Archives of Paediatric Dentistry
Junqueira M.A.; Cunha N.N.O.; Caixeta F.F.; Marques N.C.T.; Oliveira T.M.; Moretti A.B.S.; Cosme-Silva L.; Sakai V.T.	Clinical, radiographic and histological evaluation of primary teeth pulpotomy using MTA and ferric sulfate	2018	Brazilian Dental Journal
Songsiripradubboon S, Banlunara W, Sangvanich P, Trairatvorakul C, Thunyakitpisal P.	Clinical, radiographic, and histologic analysis of the effects of acemannan used in direct pulp capping of human primary teeth: short-term outcomes	2016	Odontology
Sahin N, Saygili S, Akcay M.	Clinical, radiographic, and histological evaluation of three different pulp-capping materials in indirect pulp treatment of primary teeth: a randomized clinical trial	2021	Clin Oral Investig

Abdul M.; Murali N.; Rai P.; Mirza M.; Salim S.; Aparna M.; Singh S.	Clinico-histological evaluation of dentino-pulpal complex of direct pulp capping agents: A clinical study	2021	Journal of Pharmacy and Bioallied Sciences
Lee YH, Kim HS, Kim JS, Yu MK, Cho SD, Jeon JG, Yi HK.	C-myb Regulates Autophagy for Pulp Vitality in Glucose Oxidative Stress	2016	J Dent Res
Khan M.	Coding guidelines for dentists	2014	SADJ : journal of the South African Dental Association = tydskrif van die Suid-Afrikaanse Tandheelkundige Vereniging
Gund M.P.; Hannig M.; Laschke M.W.; Lehmann A.; Schindler A.; Rupf S.	Cold atmospheric plasma does not affect the regenerative potential of the pulp in rats	2022	Plasma Processes and Polymers
Gover E.; Rakhra D.	Cold Lateral Condensation Root Canal Treatment to Retain Fractured Canines of a Ferret (Mustela Putorius Furo)	2022	Journal of Exotic Pet Medicine
Tavares KIMC, Pinto JC, Santos-Junior AO, Esteves Torres FF, Guerreiro-Tanomaru JM, Tanomaru-Filho M.	Combination of a new ultrasonic tip with rotary systems for the preparation of flattened root canals	2021	Restor Dent Endod
Liu X.; Wang C.; Pang L.; Pan L.; Zhang Q.	Combination of resolvin E1 and lipoxin A4 promotes the resolution of pulpitis by inhibiting NF-κB activation through upregulating sirtuin 7 in dental pulp fibroblasts	2022	Cell Proliferation
Kim C.K.; Hwang J.-Y.; Hong T.H.; Lee D.M.; Lee K.; Nam H.; Joo K.M.	Combination stem cell therapy using dental pulp stem cells and human umbilical vein endothelial cells for critical hindlimb ischemia	2022	BMB Reports
Rahayu R.P.; Pribadi N.; Widjiastuti I.; Nugrahani N.A.	Combinations of propolis and Ca(OH)2 in dental pulp capping treatment for the stimulation of reparative dentin formation in a rat model	2020	F1000Research
Küçükkaya Eren S.; Bahador Zirh E.; Zirh S.; Sharafi P.; Zeybek N.D.	Combined effects of bone morphogenetic protein-7 and mineral trioxide aggregate on the proliferation, migration, and differentiation of human dental pulp stem cells	2022	Journal of Applied Oral Science
Susila A.V.; Sugumar R.; Chandana C.S.; Subbarao C.V.	Combined effects of photodynamic therapy and irrigants in disinfection of root canals	2016	Journal of Biophotonics
Nagahara T., Takeda K., Aida Y., Iwata T., Yagi R., Kurihara H., Shiba H.	Combined endodontic and periodontal management of a class 3 invasive cervical resorption in a mandibular first molar	2018	Clinical Case Reports
Garrido I.; Abella F.; Ordinola-Zapata R.; Duran-Sindreu F.; Roig M.	Combined Endodontic Therapy and Intentional Replantation for the Treatment of Palatogingival Groove	2016	Journal of Endodontics
Castelo-Baz P.; Ramos-Barbosa I.; Martín-Biedma B.; Dablanca-Blanco A.B.; Varela-Patiño P.; Blanco-Carrión J.	Combined endodontic-periodontal treatment of a palatogingival groove	2015	Journal of Endodontics
Lee S.; Park Y.-T.; Setzer F.C.	Combined Regenerative and Vital Pulp Therapies in an Immature Mandibular Molar: A Case Report	2020	Journal of Endodontics
Terauchi Y.; Bakland L.K.; Bogen G.	Combined Root Canal Therapies in Multirooted Teeth with Pulpal Disease	2021	Journal of Endodontics
Vaishnavi Devi B., Ravindran V., Delphine Priscilla Antony S.	Common irrigants used by pediatric dentists for permanent molar root canal therapy	2020	International Journal of Research in Pharmaceutical Sciences
de Almeida T.B.; Zotelli V.L.R.; Wada R.S.; Sousa M.L.R.	Comparative Analgesia Between Acupuncture and Dipyrone in Odontalgia	2019	JAMS Journal of Acupuncture and Meridian Studies
Katge F.A., Patil D.P.	Comparative Analysis of 2 Calcium Silicate-based Cements (Biodentine and Mineral Trioxide Aggregate) as Direct Pulp-capping Agent in Young Permanent Molars: A Split Mouth Study	2017	Journal of endodontics
Jacob J., Paul M., Sara B., Steaphen P., Philip N., Mathew J.	Comparative Analysis of Dentinal Crack Formation Following Root Canal Instrumentation with Hand K-Flex Files, ProTaper Next, and Self-adjusting Files	2019	The journal of contemporary dental practice
Faus-Matoses V., Ibáñez E.B., Faus-Llácer V., Ruiz-Sánchez C., Zubizarreta-Macho Á., Faus-Matoses I.	Comparative Analysis of Ease of Removal of Fractured NiTi Endodontic Rotary Files from the Root Canal System—An In Vitro Study	2022	International Journal of Environmental Research and Public Health
Ren H.; Sang Y.; Zhang F.; Liu Z.; Qi N.; Chen Y.	Comparative Analysis of Human Mesenchymal Stem Cells from Umbilical Cord, Dental Pulp, and Menstrual Blood as Sources for Cell Therapy	2016	Stem Cells International
Abbas O.L.; Özatik O.; Gönen Z.B.; Öğüt S.; Özatik F.Y.; Salkın H.; Musmul A.	Comparative Analysis of Mesenchymal Stem Cells from Bone Marrow, Adipose Tissue, and Dental Pulp as Sources of Cell Therapy for Zone of Stasis Burns	2019	Journal of Investigative Surgery

Yu S.; Diao S.; Wang J.; Ding G.; Yang D.; Fan Z.	Comparative analysis of proliferation and differentiation potentials of stem cells from inflamed pulp of deciduous teeth and stem cells from exfoliated deciduous teeth	2014	BioMed Research International
Loureiro C.; Buzalaf M.A.R.; Pessan J.P.; de Moraes F.R.N.; Pelá V.T.; Ventura T.M.O.; Jacinto R.C.	Comparative analysis of the proteomic profile of the dental pulp in different conditions. A pilot study	2020	Brazilian Dental Journal
Rao H.M., Rajkumar K., Sabnani S., Kumaraswamy M., Vishwanath V., Ramadoss R.	COMPARATIVE ASSESSMENT OF CLINICAL OUTCOMES IN VITAL PULP THERAPY WITH BIOACTIVEMTA:AN INVIVO STUDY	2022	Journal of Pharmaceutical Negative Results
Musale P.K., Jain K.R., Kothare S.S.	Comparative assessment of dentin removal following hand and rotary instrumentation in primary molars using cone-beam computed tomography	2019	Journal of the Indian Society of Pedodontics and Preventive Dentistry
Sanz J.L.; Soler-Doria A.; López-García S.; García-Bernal D.; Rodríguez-Lozano F.J.; Lozano A.; Llena C.; Forner L.; Guerrero-Gironés J.; Melo M.	Comparative Biological Properties and Mineralization Potential of 3 Endodontic Materials for Vital Pulp Therapy: Theracal PT, Theracal LC, and Biodentine on Human Dental Pulp Stem Cells	2021	Journal of Endodontics
Küden C.; Karakaş S.N.; Batmaz S.G.	Comparative chemical properties, bioactivity, and cytotoxicity of resin-modified calcium silicate–based pulp capping materials on human dental pulp stem cells	2022	Clinical Oral Investigations
Lozano-Guillén A.; López-García S.; Rodríguez-Lozano F.J.; Sanz J.L.; Lozano A.; Llena C.; Forner L.	Comparative cytocompatibility of the new calcium silicate-based cement NeoPutty versus NeoMTA Plus and MTA on human dental pulp cells: an in vitro study	2022	Clinical Oral Investigations
Aragão MGB, Costa CAGA, Lima RA, Rodrigues LKA, Duarte S, Zanin ICJ.	Comparative Effect of Two Red Lights on Streptococcus mutans Biofilms and Assessment of Temperature Variances in Human Teeth During In Vitro Photodynamic Antimicrobial Chemotherapy	2019	Photobiomodul Photomed Laser Surg
Vatanpour M., Fazlyab M., Nikzad M.	Comparative effects of erbium-doped yttrium aluminum garnet laser, the shock wave-enhanced emission photoacoustic streaming, and the conventional needle irrigation on apical extrusion of irrigants	2022	Photodiagnosis and Photodynamic Therapy
Attar R.H., Baghdadi Z.D.	Comparative efficacy of active and passive distraction during restorative treatment in children using an iPad versus audiovisual eyeglasses: a randomised controlled trial	2015	European archives of paediatric dentistry : official journal of the European Academy of Paediatric Dentistry
Aggarwal V.; Singla M.; Miglani S.; Kohli S.; Singh S.	Comparative evaluation of 1.8 mL and 3.6 mL of 2% lidocaine with 1:200,000 epinephrine for inferior alveolar nerve block in patients with irreversible pulpitis: A prospective, randomized single-blind study	2012	Journal of Endodontics
Syed GA, Mulay SA.	Comparative Evaluation of Anesthetic Efficacy of 4% Articaine and 2% Lidocaine for Buccal Infiltration in Adult Patients with Irreversible Pulpitis of Maxillary First Molar: A Prospective Randomized Study	2022	Contemp Clin Dent
Afkhami F, Pirmoazen S, Ardestani A, Fard MJK.	Comparative evaluation of anesthetic efficacy of inferior alveolar nerve block and inferior alveolar nerve block plus buccal or lingual infiltration using articaine in mandibular molars with irreversible pulpitis: a preliminary prospective randomized single-blind clinical trial	2021	Quintessence Int
Reddy N.B.N.; Sridhar D.; Rajkumar A.; Murugesan S.; Selvaraj K.; Sankar S.	Comparative Evaluation of Antifungal Activity of Octenidine: An In Vitro Confocal Laser Study	2020	Journal of Contemporary Dental Practice
Bhullar K.K., Kaur S., Malhotra S., Singh H., Handa A., Kaur R.	Comparative Evaluation Of Antimicrobial Efficacy Of Three Endodontic Irrigating Solutions Against Enterococcus Faecalis	2020	European Journal of Molecular and Clinical Medicine
Parikh M, Kishan KV, Shah NC, Parikh M, Saklecha P.	Comparative evaluation of biodentine and endosequence root repair material as direct pulp capping material: A clinical study	2021	J Conserv Dent
Boddeda K.; Rani C.; V Vanga N.; Chandrabhatla S.	Comparative evaluation of biodentine, 2% chlorhexidine with RMGIC and calcium hydroxide as indirect pulp capping materials in primary molars: An in vivo study	2019	Journal of Indian Society of Pedodontics and Preventive Dentistry
Khandelwal A., Palanivelu A.	Comparative evaluation of canal transportation and centering ability of WaveOne Gold and EndoSequence Reciprocating File systems using cone-beam computed tomography	2020	Drug Invention Today

Beegum M.S.F.; George S.; Anandaraj S.; Sumi Issac J.; Khan S.N.; Ali Habibullah M.	Comparative evaluation of diffused calcium and hydroxyl ion release from three different Indirect pulp capping agents in permanent teeth – An in vitro study	2021	Saudi Dental Journal
Aripirala M, Bansal K, Mathur VP, Tewari N, Gupta P, Logani A.	Comparative evaluation of diode laser and simvastatin gel in pulpotomy of primary molars: A randomized clinical trial	2021	J Indian Soc Pedod Prev Dent
Sharma N, Malik N, Garg Y, Singh H, Garg K, Bagaria A.	Comparative Evaluation of Effect of Lasers and Biodentine in Dentine Regeneration: A Clinical Study	2019	J Contemp Dent Pract
Kamath A., Kudva A.R., Kini S.	Comparative evaluation of efficacy of electronic apex locators in determining the working length of the canal which is used during a routine root canal treatment-in vivo study	2019	Indian Journal of Public Health Research and Development
Yadav P.; Indushekar K.R.; Saraf B.G.; Sheoran N.; Sardana D.	Comparative evaluation of ferric sulfate, electrosurgical and diode laser on human primary molars pulpotomy: An "in-vivo" study	2014	Laser Therapy
Airen P.; Shigli A.; Airen B.	Comparative evaluation of formocresol and mineral trioxide aggregate in pulpotomized primary molars-2 year follow up	2012	Journal of Clinical Pediatric Dentistry
İslam A.; Özverel C.S.; Yilmaz H.G.	Comparative evaluation of low-level laser therapy on proliferation of long-term cryopreserved human dental pulp cells isolated from deciduous and permanent teeth	2021	Lasers in Medical Science
Kalaskar R.R., Badhe H.K., Ninawe N.S., Khade A.V., Balasubramanian S., Kamki H.	Comparative Evaluation of Ozonoid Olive Oil and Calcium Hydroxide as an Indirect Pulp Capping Agent in Primary Mandibular Second Molar: A Randomized Controlled Trial	2022	The journal of contemporary dental practice
Altan H.; Belevcikli M.; Coşgun A.; Demir O.	Comparative evaluation of pain perception with a new needle-free system and dental needle method in children: a randomized clinical trial	2021	BMC Anesthesiology
Keswani D.; Pandey R.K.; Ansari A.; Gupta S.	Comparative evaluation of platelet-rich fibrin and mineral trioxide aggregate as pulpotomy Agents in Permanent Teeth with Incomplete Root Development: A Randomized Controlled Trial	2014	Journal of Endodontics
Bhawalkar A., Mulay S., Desai H., Shetty R.	Comparative evaluation of post-operative endodontic pain using various intracanal medicaments after instrumentation with rotary single-file system	2021	Current Pediatric Research
Ramamoorthi S.; Nivedhitha M.S.; Divyanand M.J.	Comparative evaluation of postoperative pain after using endodontic needle and EndoActivator during root canal irrigation: A randomised controlled trial	2015	Australian Endodontic Journal
Teja K.V.; Ramesh S.; Vasundhara K.A.	Comparative Evaluation of Preemptive and Preventive Analgesic Effect of Oral Ibuprofen in Single Visit Root Canal Treatment- A Prospective Randomised Pilot Study	2022	European Endodontic Journal
Ozdemir Y.; Kutukculer N.; Topaloglu-Ak A.; Kose T.; Eronat C.	Comparative evaluation of pro-inflammatory cytokine levels in pulpotomized primary molars	2015	Journal of Oral Science
Amin L.E.; Montaser M.	Comparative evaluation of pulpal repair after direct pulp capping using stem cell therapy and biodentine: An animal study	2021	Australian Endodontic Journal
Mali S.R.; Makandar S.; Mangala T.M.; Phase A.	Comparative evaluation of reparative dentin formation by using different pulp capping agents: An in vivo study	2020	International Journal of Pharmaceutical Research
Khedmat S.; Sarraf P.; Seyedjafari E.; Sanaei-rad P.; Noori F.	Comparative evaluation of the effect of cold ceramic and MTA-Angelus on cell viability, attachment and differentiation of dental pulp stem cells and periodontal ligament fibroblasts: an in vitro study	2021	BMC Oral Health
Satish S.V.; Shetty K.P.; Kilaru K.; Bhargavi P.; Reddy E.S.; Bellutgi A.	Comparative evaluation of the efficacy of 2% lidocaine containing 1:200,000 epinephrine with and without hyaluronidase (75 IU) in patients with irreversible pulpitis	2013	Journal of Endodontics
Mahapatra J.; Nikhade P.P.; Belsare A.	Comparative evaluation of the efficacy of theracal lc, mineral trioxide aggregate and biodentine as direct pulp capping materials in patients with pulpal exposure in posterior teeth-an interventional study	2019	International Journal of Pharmaceutical Research
Hemmanur S., Delphine Priscilla Antony S., Sowmya K.	Comparative evaluation of the type of sealer used for obturation of single visit root canal treated maxillary and mandibular posterior teeth-a retrospective analysis	2020	International Journal of Research in Pharmaceutical Sciences
Nadkarni M.A.; Chen Z.; Wilkins M.R.; Hunter N.	Comparative genome analysis of Lactobacillus rhamnosus clinical isolates from initial stages of dental pulp infection: Identification of a new exopolysaccharide cluster	2014	PLoS ONE
Nelson-Filho P.; Ruviére D.B.; De Queiroz A.M.; De Paula-Silva F.W.G.; Da Silva R.A.B.; Lucisano M.P.; Da Silva L.A.B.	Comparative molecular analysis of gram-negative bacteria in primary teeth with irreversible pulpitis or periapical pathology	2018	Pediatric Dentistry

Jefferies S.R.; Swift E.J., Jr.	Comparative Performance of Mineral Trioxide Aggregate Versus Calcium Hydroxide as a Direct Pulp Capping Agent	2016	Journal of Esthetic and Restorative Dentistry
Yi-Yueh L.; Xin G.; Shi-Hao W.; Hui-Ling W.; Gao-Hua W.	Comparative study of auxiliary effect on dental anxiety, pain and compliance during adult dental root canal treatment under therapeutic Chinese music or western classic music	2014	Physikalische Medizin Rehabilitationsmedizin Kurortmedizin
Lee H.; Shin Y.; Kim S.-O.; Lee H.-S.; Choi H.-J.; Song J.S.	Comparative Study of Pulpal Responses to Pulpotomy with ProRoot MTA, RetroMTA, and TheraCal in Dogs' Teeth	2015	Journal of Endodontics
Bin Alshaibah W.M.; El-Shehaby F.; El-Dokky N.; Reda A.	Comparative study on the microbial adhesion to preveneered and stainless steel crowns	2012	Journal of Indian Society of Pedodontics and Preventive Dentistry
Bashyam R.; Krishnan R.; Murali K.; Selvarajan N.B.; Vasaviah S.K.; Duraisamy V.	Comparative study to evaluate the apical sealing ability of MTA plus and biodentin using a bacterial leakage model: In vitro study	2021	International Journal of Current Research and Review
Faruangsaeng T.; Thaweesapphitak S.; Khamwachirapitak C.; Porntaveetus T.; Shotelersuk V.	Comparative transcriptome profiles of human dental pulp stem cells from maxillary and mandibular teeth	2022	Scientific Reports
Yu Y.-H.; Kushnir L.; Kohli M.; Karabucak B.	Comparing the incidence of postoperative pain after root canal filling with warm vertical obturation with resin-based sealer and sealer-based obturation with calcium silicate-based sealer: a prospective clinical trial	2021	Clinical Oral Investigations
Costa E Silva L.L.; Cosme-Silva L.; Sakai V.T.; Lopes C.S.; da Silveira A.P.P.; Moretti Neto R.T.; Gomes-Filho J.E.; Oliveira T.M.; Moretti A.B.S.	Comparison between calcium hydroxide mixtures and mineral trioxide aggregate in primary teeth pulpotomy: A randomized controlled trial	2019	Journal of Applied Oral Science
Kamel D.O.; Wahba N.A.; Talaat D.M.	Comparison between positive dental images and neutral images in managing anticipatory anxiety of children	2017	Journal of Clinical Pediatric Dentistry
Golbasi F.; Erdemir A.; Kisa U.	Comparison of ADAMTS Levels in Pulp Tissue Samples of Healthy and Symptomatic Irreversible Pulpitis Teeth	2022	Journal of Endodontics
Michetti J, Basarab A, Diemer F, Kouame D.	Comparison of an adaptive local thresholding method on CBCT and µCT endodontic images	2017	Phys Med Biol
Farooq A.; ur Rahman Qazi F.; Siddiqui J.; Faraz S.A.; Rasheed A.	COMPARISON OF AN EXPERIMENTAL ROOT CANAL IRRIGANT (SAPINDUS MUKOROSSI) AND ETHYLENEDIAMINETETRAACETIC ACID ON MICROHARDNESS OF HUMAN DENTIN	2022	Journal of Ayub Medical College
Zain M, Rehman Khattak SU, Sikandar H, Shah SA, Fayyaz.	Comparison of Anaesthetic Efficacy of 4% Articaine Primary Buccal Infiltration Versus 2% Lidocaine Inferior Alveolar Nerve Block in Symptomatic Mandibular First Molar Teeth	2016	J Coll Physicians Surg Pak
Ramzan S., Zaighum M., Iqbal Z., Darshana, Ahmed R., Memon A.	Comparison of anesthetic efficacy of articaine and lidocaine in patients with irreversible pulpitis	2021	Pakistan Journal of Medical and Health Sciences
Sarwar H., Ahmed S., Naeem M.M., Shaikh A.A., Raza S.A., Kamal I.	Comparison of Anesthetic efficacy of two different volumes of 4% articaine for inferior alveolar nerve block during endodontic therapy of mandibular molars with symptomatic irreversible pulpitis	2021	Pakistan Journal of Medical and Health Sciences
Yahata Y, Masuda Y, Komabayashi T.	Comparison of apical centring ability between incisal-shifted access and traditional lingual access for maxillary anterior teeth	2017	Aust Endod J
Low N., Zhen Jie S., Bhatia S., Davamani F., Nagendrababu V.	Comparison of Apical Extrusion of Bacteria After Glide Path Preparation Between Manual K File, One G Rotary, and WaveOne Gold Glider Reciprocation Preparations	2021	European endodontic journal
Tzanetakis G.N.; Azcarate-Peril M.A.; Zachaki S.; Panopoulos P.; Kontakiotis E.G.; Madianos P.N.; Divaris K.	Comparison of Bacterial Community Composition of Primary and Persistent Endodontic Infections Using Pyrosequencing	2015	Journal of Endodontics
Kumar M., Paliwal A., Manish K., Ganapathy S.K., Kumari N., Singh A.R.	Comparison of Canal Transportation in TruNatomy, ProTaper Gold, and Hyflex Electric Discharge Machining File Using Cone-beam Computed Tomography	2021	The journal of contemporary dental practice
Schoppe C.; Hellige M.; Rohn K.; Ohnesorge B.; Bienert-Zeit A.	Comparison of computed tomography and high-field (3.0 T) magnetic resonance imaging of age-related variances in selected equine maxillary cheek teeth and adjacent tissues	2017	BMC Veterinary Research
Li L.; Chen H.J.; Lian Y.; Wang T.	Comparison of dental pulp periodontal therapy and conventional simple periodontal therapy as treatment modalities for severe periodontitis	2021	World Journal of Clinical Cases
Lee S, Lee K, Kim H, An J, Han J, Lee T, Jeong H, Cho Y.	Comparison of dental radiography and computed tomography: measurement of dentoalveolar structures in healthy, small-sized dogs and cats	2020	J Vet Sci

Banga M., Jasuja P., Khurana H., Thakur A., Sadhar S., Yousuf A.	COMPARISON OF DENTINAL DEFECTS FOLLOWING ROOT CANAL PREPARATION USING MANUAL AND ROTARY INSTRUMENTATION IN EXTRACTED MANDIBULAR PREMOLARS– AN IN VITRO STUDY	2022	NeuroQuantology
Valera M.C.; Cardoso F.G.D.R.; Chung A.; Xavier A.C.C.; Figueiredo M.D.; Martinho F.C.; Palo R.M.	Comparison of different irrigants in the removal of endotoxins and cultivable microorganisms from infected root canals	2015	Scientific World Journal
Najmi N., Jameel A., Abidi Y.	Comparison of Efficacy of Mineral Trioxide Aggregate and Calcium Hydroxide as Pulpotomy Agents in Primary Molars	2022	Medical Forum Monthly
Nagi S.E.; Khan F.R.; Rahman M.	Comparison of fracture and deformation in the rotary endodontic instruments: Protaper versus K-3 system	2016	JPMA. The Journal of the Pakistan Medical Association
Salehi F.; Dibaj M.; Mohammadi A.; Sattari M.	Comparison of Gene Expression of Different Isoforms of Osteopontin in Symptomatic Irreversible Pulpitis of Human Dental Pulp	2022	Iranian Endodontic Journal
Sushynski JM, Zealand CM, Botero TM, Boynton JR, Majewski RF, Shelburne CE, Hu JC.	Comparison of gray mineral trioxide aggregate and diluted formocresol in pulpotomized primary molars: a 6- to 24-month observation	2012	Pediatr Dent
Bierma M.M.; McClanahan S.; Baisden M.K.; Bowles W.R.	Comparison of heat-testing methodology	2012	Journal of Endodontics
Ghattas Ayoub C.; Aminoshariae A.; Bakkar M.; Ghosh S.; Bonfield T.; Demko C.; Montagnese T.A.; Mickel A.K.	Comparison of IL-1β, TNF-α, hBD-2, and hBD-3 Expression in the Dental Pulp of Smokers Versus Nonsmokers	2017	Journal of Endodontics
Chen X, Zhang H, Zhong J, Yan W, Lin B, Ding M, Xue S, Xia B.	Comparison of indirect pulp treatment and iRoot BP Plus pulpotomy in primary teeth with extremely deep caries: a prospective randomized trial	2021	Clin Oral Investig
Gregory P, Lea J, Walinski C, Terese-Koch D, Hottel T, Fields D, Rodriguez C.	Comparison of Laser Versus Rotary Instrumentation- A Case Study	2016	J Tenn Dent Assoc
Asopa S.S., Asopa V., Arya A., Attargekar V., Chabra C., Gupta D.	Comparison of long-term outcome of implant supported crowns and root canal treated crowns: An observational study	2022	NeuroQuantology
Madhavan S, Jude SM, Achammada S, Ullattuthodi S, Kuriachan T, Jacob J.	Comparison of Marginal Accuracy of Interim Restoration Fabricated from Self-cure Composite and Autopolymerizing Acrylic Resin: An In Vitro Study	2020	J Pharm Bioallied Sci
Drumond J.P.S.C.; Maeda W.; Nascimento W.M.; Campos D.D.L.; Prado M.C.; de-Jesus-Soares A.; Frozoni M.	Comparison of Postobturation Pain Experience after Apical Extrusion of Calcium Silicate– and Resin–Based Root Canal Sealers	2021	Journal of Endodontics
Tavakoli A.; Tazik M.E.; Abbasi A.	Comparison of Production of Pain and Oxidative Stress after Induction of Local Nerve Block or Use of NSAIDs following Painful Dental Procedures in Dogs	2021	Iranian Journal of Veterinary Surgery
De Rossi A.; Silva L.A.; Gatón-Hernández P.; Sousa-Neto M.D.; Nelson-Filho P.; Silva R.A.; de Queiroz A.M.	Comparison of pulpal responses to pulpotomy and pulp capping with biodentine and mineral trioxide aggregate in dogs	2014	Journal of endodontics
Zeynep E., Gunes B., Bayrakdar I.S.	Comparison of root canal shaping ability of different heat-treated NiTi single files: A micro-CT study	2022	Quintessence International
Boby A.; Pai D.; Ginjupalli K.; Gaur S.	Comparison of shear bond strength of light cure mineral trioxide aggregate and light cure calcium hydroxide with nanofilled composite: A stereomicroscopic and scanning electron microscope analysis	2020	Journal of Indian Society of Pedodontics and Preventive Dentistry
Pereira L.O.; Rubini M.R.; Silva J.R.; Oliveira D.M.; Silva I.C.R.; Poças-Fonseca M.J.; Azevedo R.B.	Comparison of stem cell properties of cells isolated from normal and inflamed dental pulps	2012	International Endodontic Journal
Marty M.; Broutin A.; Vergnes J.-N.; Vaysse F.	Comparison of student's perceptions between 3D printed models versus series models in paediatric dentistry hands-on session	2019	European Journal of Dental Education
AlMotawah F.N., Pani S.C., AlKharashi T., AlKhalaf S., AlKhathlan M., AlSultan F., AlMughirah A.	Comparison of survival rates of stainless-steel crowns placed with and without pulpotomy: A two-year retrospective study	2020	International Journal of Dentistry
Adibi A, Sobhnamayan F, Ostovar Zijerdi N, Tajik M, Paknahad M.	Comparison of the Accuracy of CBCT Images and Apex Locator in Detection of External Root Resorption with Perforation	2022	J Dent (Shiraz)

Jaouni A.W.A., Abboud S.	Comparison of the Bond Strength of Circular and Oval-Shaped FiberReinforced Composite Posts in Oval Shaped Canals	2022	Research Journal of Pharmacy and Technology
Melo Ribeiro M.V.D.; Silva-Sousa Y.T.; Versiani M.A.; Lamira A.; Steier L.; Pécora J.D.; Sousa-Neto M.D.D.	Comparison of the cleaning efficacy of self-adjusting file and rotary systems in the apical third of oval-shaped canals	2013	Journal of Endodontics
Lei X., Liu Q., Qian H., Ren F., Fang J., Liu H., Wu X., Yang J.	Comparison of the clinical efficacy of iRoot BP plus with mineral trioxide aggregate in the pulpotomy of deciduous teeth	2020	International Journal of Clinical and Experimental Medicine
Soukup JW, Drees R, Koenig LJ, Snyder CJ, Hetzel S, Miles CR, Schwarz T.	Comparison of the Diagnostic Image Quality of the Canine Maxillary Dentoalveolar Structures Obtained by Cone Beam Computed Tomography and 64-Multidetector Row Computed Tomography	2015	J Vet Dent
Hajihassani N., Mohammadi N., Karimi Kelayeh A., Aalaei S.	Comparison of the effect of post space preparation time on the apical seal of two different sealers	2022	BMC oral health
Beus C.; Safavi K.; Stratton J.; Kaufman B.	Comparison of the effect of two endodontic irrigation protocols on the elimination of bacteria from root canal system: A prospective, randomized clinical trial	2012	Journal of Endodontics
Kebriaee F.; Sarraf Shirazi A.; Fani K.; Moharreri F.; Soltanifar A.; Khaksar Y.; Mazhari F.	Comparison of the effects of cognitive behavioural therapy and inhalation sedation on child dental anxiety	2015	European Archives of Paediatric Dentistry
Cimilli H.; Karacayli U.; Şişman N.; Kartal N.; Mumcu G.	Comparison of the oral health-related quality of life and dental pain in symptomatic irreversible pulpitis and pericoronitis	2012	Journal of Dental Sciences
Łuczaj-Cepowicz E.; Marczuk-Kolada G.; Pawińska M.; Różycki J.; Chorzewska E.	Comparison of the radiopacity of selected materials used for vital pulp therapy: An in vitro assessment; Porównanie pochłaniania promieni rentgenowskich przez wybrane materiały stosowane w leczeniu biologicznym miazgi – ocena in vitro	2019	Dental and Medical Problems
Teixeira VP, Rodrigues DB, Reis MA, Castro EC, Piccioni DE, Beghini M, de Lima Pereira SA.	Comparison of the total length and areas of upper central incisors between males and females using computer-assisted morphometry	2013	Anat Sci Int
Ayedun O.S.; Oredugba F.A.; Sote E.O.	Comparison of the treatment outcomes of the conventional stainless steel crown restorations and the hall technique in the treatment of carious primary molars	2021	Nigerian Journal of Clinical Practice
Vanitha S, Sherwood IA.	Comparison of three different apex locators in determining the working length of mandibular first molar teeth with irreversible pulpitis compared with an intraoral periapical radiograph: A block randomized, controlled, clinical trial	2019	J Investig Clin Dent
Selvendran K.E.; Ahamed A.S.; Krishnamurthy M.; Kumar V.N.; Raju V.	Comparison of three different materials used for indirect pulp capping in permanent molars: An in vivo study	2022	Journal of Conservative Dentistry
Poludasu M.; Mallela G.M.K.; Puppala R.; Kethineni B.; Dandotikar D.	Comparison of Three Treatment Techniques for Deep Carious Lesions in Primary Teeth: An In Vivo Study	2022	International Journal of Clinical Pediatric Dentistry
Rouhani A, Akbari M, Farhadi-Faz A.	Comparison of Tooth Discoloration Induced by Calcium-Enriched Mixture and Mineral Trioxide Aggregate	2016	Iran Endod J
Beer F.; Buchmair A.; Wernisch J.; Georgopoulos A.; Moritz A.	Comparison of two diode lasers on bactericidity in root canals-an in vitro study	2012	Lasers in Medical Science
Waly A.S., Yamany I., Abbas H.M., A Alsairafi M.A., F Bazzaz R.M., Bogari D.F., Alhazzazi T.Y.	Comparison of two pediatric rotary file systems and hand instrumentation in primary molar: An ex vivo cone-beam computed tomographic study	2021	Nigerian journal of clinical practice
Armand A.; Khani M.; Asnaashari M.; AliAhmadi A.; Shokri B.	Comparison study of root canal disinfection by cold plasma jet and photodynamic therapy	2019	Photodiagnosis and Photodynamic Therapy
Giraud T.; Rufas P.; Chmilewsky F.; Rombouts C.; Dejou J.; Jeanneau C.; About I.	Complement Activation by Pulp Capping Materials Plays a Significant Role in Both Inflammatory and Pulp Stem Cells' Recruitment	2017	Journal of Endodontics
Pultanasarn P., Thaungwilai K., Singhatanadgid P., Prateepsawangwong B., Singhatanadgit W.	Composite core-supported stainless steel crowns enhance fracture resistance of severely damaged primary posterior teeth	2020	Pediatric Dental Journal
Lei F.; Zhang H.; Xie X.	Comprehensive analysis of an lncRNA-miRNA-mRNA competing endogenous RNA network in pulpitis	2019	PeerJ
Liu L.; Wang T.; Huang D.; Song D.	Comprehensive Analysis of Differentially Expressed Genes in Clinically Diagnosed Irreversible Pulpitis by	2021	Journal of Endodontics

	Multiplatform Data Integration Using a Robust Rank Aggregation Approach		
Wang Y, Wu L, Guo H, Qiu T, Huang Y, Lin B, Wang L.	Computation of tooth axes of existent and missing teeth from 3D CT images	2015	Biomed Tech (Berl)
Kurthukoti AJ, Sharma P, Swamy DF, Shashidara R, Swamy EB.	Computed Tomographic Morphometry of the Internal Anatomy of Mandibular Second Primary Molars	2015	Int J Clin Pediatr Dent
Suardita K.; Arundina I.; Tedjosasongko U.; Yuliati A.; Peeters H.H.; Wijaksana I.K.E.; Surboyo M.D.C.	Concanavalin A Enhanced Proliferation and Osteogenic Differentiation of Dental Pulp Stem Cells	2020	European Journal of Dentistry
Setiati H.D., Suprastiwi E., Artiningsih D.A.N.P., Utami L.P.T.B.	Concentration dependent effects of carboxymethyl chitosan on dentin remineralization with amorphous calcium phosphate	2020	International Journal of Applied Pharmaceutics
Evrosimovska B.; Dimova C.; Kovacevska I.; Panov S.	Concentration of collagenases (MMP-1, -8, -13) in patients with chronically inflamed dental pulp tissue	2012	Prilozi / Makedonska akademija na naukite i umetnostite, Oddelenie za biološki i medicinski nauki = Contributions / Macedonian Academy of Sciences and Arts, Section of Biological and Medical Sciences
Lacerda-Pinheiro S.; Dimitrova-Nakov S.; Harichane Y.; Souyri M.; Petit-Cocault L.; Legrès L.; Marchadier A.; Baudry A.; Ribes S.; Goldberg M.; Kellermann O.; Poliard A.	Concomitant multipotent and unipotent dental pulp progenitors and their respective contribution tomineralised tissue formation	2012	European Cells and Materials
Anjali P., Varma B., Suresh Kumar J., Kumaran P., Xavier A.M.	Concurrent Occurrence of Bilateral Hypodontia and Microdontia in a Nonsyndromic Paediatric Patient: A Case Report	2020	Journal of Clinical and Diagnostic Research
Hall B.E.; Zhang L.; Sun Z.J.; Utreras E.; Prochazkova M.; Cho A.; Terse A.; Arany P.; Dolan J.C.; Schmidt B.L.; Kulkarni A.B.	Conditional TNF-α Overexpression in the Tooth and Alveolar Bone Results in Painful Pulpitis and Osteitis	2016	Journal of Dental Research
Krishnan U.; Moule A.J.; Alawadhi A.	Cone beam CT assisted re-treatment of class 3 invasive cervical resorption	2015	BMJ Case Reports
Hasheminia S.M., Farhad A., Sheikhi M., Soltani P., Hendi S.S., Ahmadi M.	Cone-beam Computed Tomographic Analysis of Canal Transportation and Centering Ability of Single-file Systems	2018	Journal of endodontics
Zoya-Farook A.; Abhishek P.; Shahabadi A.	Cone-beam Computed Tomographic Evaluation and Endodontic Management of a Mandibular First Premolar with Type IX Canal Configuration: Case Report	2017	Journal of Endodontics
Zhao L.; Pan J.; Xu L.; Pallikonda Rajasekaran M.	Cone-Beam Computed Tomography Image Features under Intelligent Three-Dimensional Reconstruction Algorithm in the Evaluation of Intraoperative and Postoperative Curative Effect of Dental Pulp Disease Using Root Canal Therapy	2022	Scientific Programming
Celikten B, Orhan K, Aksoy U, Tufenkci P, Kalender A, Basmaci F, Dabaj P.	Cone-beam CT evaluation of root canal morphology of maxillary and mandibular premolars in a Turkish Cypriot population	2016	BDJ Open
Komiya H, Shimizu K, Ishii K, Kudo H, Okamura T, Kanno K, Shinoda M, Ogiso B, Iwata K.	Connexin 43 expression in satellite glial cells contributes to ectopic tooth-pulp pain	2018	J Oral Sci
Sanjari K., Bayani M., Zadeh H.E.	Conservative dental management of a patient with Epidermolysis bullosa. A case report	2020	Pediatric Dental Journal
Taha N.A., About I., Sedgley C.M., Messer H.H.	Conservative Management of Mature Permanent Teeth with Carious Pulp Exposure	2020	Journal of endodontics
Ali A.; Arslan H.; Jethani B.	Conservative management of Type II dens invaginatus with guided endodontic approach: A case series	2019	Journal of Conservative Dentistry
Yong D, Cathro P.	Conservative pulp therapy in the management of reversible and irreversible pulpitis	2021	Aust Dent J
Marinčák D.; Doležel V.; Přibyl M.; Voborná I.; Marek I.; Šedý J.; Žižka R.	Conservative treatment of complicated crown fracture and crown-root fracture of young permanent incisor—A case report with 24-month follow-up	2021	Children
Stratigaki E, Tong HJ, Seremidi K, Kloukos D, Duggal M, Gizani S.	Contemporary management of deep caries in primary teeth: a systematic review and meta-analysis	2022	Eur Arch Paediatr Dent
Fouad A.F.	Contemporary Microbial and Antimicrobial Considerations in Regenerative Endodontic Therapy	2020	Journal of Endodontics

Frozoni M, Marques MR, Hamasaki SK, Mohara NT, de Jesus Soares A, Zaia AA.	Contribution of Bone Marrow-derived Cells to Reparative Dentinogenesis Using Bone Marrow Transplantation Model	2020	J Endod
Nomura R, Matayoshi S, Otsugu M, Kitamura T, Teramoto N, Nakano K.	Contribution of Severe Dental Caries Induced by Streptococcus mutans to the Pathogenicity of Infective Endocarditis	2020	Infect Immun
Nomura R.; Ogaya Y.; Nakano K.	Contribution of the collagen-binding proteins of Streptococcus mutans to bacterial colonization of inflamed dental pulp	2016	PLoS ONE
Matoso F.B., Grecca F.S., Pinheiro L.S., Leitune V.C.B., Guterres S., Collares F.M., Kopper P.M.P.	Controlled drug delivery system endodontic paste as intracanal medication: a bench-to-chair-side case report	2022	Giornale Italiano di Endodonzia
Swanson W.B., Gong T., Zhang Z., Eberle M., Niemann D., Dong R., Rambhia K.J., Ma P.X.	Controlled release of odontogenic exosomes from a biodegradable vehicle mediates dentinogenesis as a novel biomimetic pulp capping therapy	2020	Journal of Controlled Release
Kharel B., Rai A., Suwal P., Parajuli P.K., Limbu I., Basnet B.B.	Conventional tooth supported overdenture: A case report	2021	Journal of Nepalese Prosthodontic Society
Schwendicke F.; Stolpe M.; Innes N.	Conventional treatment, Hall Technique or immediate pulpotomy for carious primary molars: a cost-effectiveness analysis	2016	International Endodontic Journal
Schaefferkoetter J., Yan J., Ortega C., Sertic A., Lechtman E., Eshet Y., Metser U., Veit-Haibach P.	Convolutional neural networks for improving image quality with noisy PET data	2020	EJNMMI Research
Havale R, Rao DG, Latha AM, Nagaraj M, Karobari NM, Tharay N.	Coronal pulp: An age biomarker - A cross-sectional radiographic study in children	2020	J Oral Maxillofac Pathol
Sulaiman AO, Shaba OP, Dosumu OO, Ajayi DM.	Coronal tissue loss in endodontically treated teeth	2012	Afr J Med Med Sci
Kim Y.-B.; Joo W.-H.; Min K.-S.	Coronectomy of a lower third molar in combination with vital pulp therapy	2014	European Journal of Dentistry
Ghousia S., Nyer Firdoose C.S.	Coronoid foramina in a pediatric mandible: An incidental finding of a morphologic and developmental anatomic variant as a distinctive documented feature	2021	National Journal of Clinical Anatomy
Ricucci D, Loghin S, Siqueira JF Jr.	Correlation between clinical and histologic pulp diagnoses	2014	J Endod
Muniz FWMG, Montagner F, Jacinto RC, Rösing CK, Gomes BPFA.	Correlation between crestal alveolar bone loss with intracanal bacteria and apical lesion area in necrotic teeth	2018	Arch Oral Biol
Di Murro B.; Papi P.; Di Murro C.; Pompa G.; Gambarini G.	Correlation between endodontic pulpal/periapical disease and retrograde peri-implantitis: A case series	2021	Australian Endodontic Journal
Naseri M.; Khayat A.; Zamaheni S.; Shojaeian S.	Correlation between histological status of the pulp and its response to sensibility tests	2017	Iranian Endodontic Journal
Rechenberg D.K.; Munir A.; Zehnder M.	Correlation between the clinically diagnosed inflammatory process and periapical index scores in severely painful endodontically involved teeth	2021	International Endodontic Journal
Brüllmann D.D., Schmidtmann I., Hornstein S., Schulze R.K.	Correlation of cone beam computed tomography (CBCT) findings in the maxillary sinus with dental diagnoses: A retrospective cross-sectional study	2012	Clinical Oral Investigations
Uhrich E.; Gautam M.; Hatton J.; Rowland K.	Corticotropin releasing factor receptor expression in painful human dental pulp	2015	Archives of Oral Biology
Abukabbos H, Tomar S, Guelmann M.	Cost Estimates for Bioactive Cement Pulpotomies and Crowns in Primary Molars	2018	Pediatr Dent
Schwendicke F, Rossi JG, Krois J, Basso M, Peric T, Turkun LS, Miletić I.	Cost-effectiveness of glass hybrid versus composite in a multi-country randomized trial	2021	J Dent
Savolainen N, Kvist T, Mannila J.	Cost-effectiveness of partial versus stepwise caries removal of deep caries lesions - a decision-analytic approach	2023	Acta Odontol Scand
Bergamo M.T.; Vitor L.L.R.; Dionísio T.J.; Marques N.C.T.; Oliveira R.C.; Ambrosio E.C.P.; Sakai V.T.; Santos C.F.; Lourenço Neto N.; Machado M.A.A.M.; Oliveira T.M.	Could the photobiomodulation therapy induce angiogenic growth factors expression from dental pulp cells?	2021	Lasers in Medical Science
De Toubes K.M.P.; Tonelli S.Q.; Nunes E.; Silveira F.F.	Cracked teeth: What clinicians and specialists need to know	2021	Dental Press Endodontics
Sadasiva K., Ramalingam S., Rajaram K., Meiyappan A.	Cracked tooth syndrome: A report of three cases	2015	Journal of Pharmacy and Bioallied Sciences

Varghese N.S.; Cherian J.; Thomas A.	Credibility of YouTube™ videos on root canal treatment in children	2022	Journal of Indian Society of Pedodontics and Preventive Dentistry
Crespo-Gallardo I, Hay-Levytska O, Martín-González J, Jiménez-Sánchez MC, Sánchez-Domínguez B, Segura-Egea JJ.	Criteria and treatment decisions in the management of deep caries lesions: Is there endodontic overtreatment?	2018	J Clin Exp Dent
Browning W.D.	Critical appraisal. 2015 Update: Approaches to Caries Removal	2015	Journal of esthetic and restorative dentistry : official publication of the American Academy of Esthetic Dentistry ... [et al.
Blanchard K.; Koehm J.	Crown reduction and vital pulp therapy in a dog with malocclusion	2018	Journal of Veterinary Dentistry
Ma L.; Makino Y.; Yamaza H.; Akiyama K.; Hoshino Y.; Song G.; Kukita T.; Nonaka K.; Shi S.; Yamaza T.	Cryopreserved Dental Pulp Tissues of Exfoliated Deciduous Teeth Is a Feasible Stem Cell Resource for Regenerative Medicine	2012	PLoS ONE
Joshi C, Joshi S.	C-shaped canal in maxillary first molars: a case report	2014	J Dent (Tehran)
Nejaim Y, Gomes AF, Rosado LPL, Freitas DQ, Martins JNR, da Silva EJNL.	C-shaped canals in mandibular molars of a Brazilian subpopulation: prevalence and root canal configuration using cone-beam computed tomography	2020	Clin Oral Investig
Ozcan G., Sekerci A.E., Kocoglu F.	C-shaped mandibular primary first molar diagnosed with cone beam computed tomography: A novel case report and literature review of primary molars' root canal systems	2016	Journal of the Indian Society of Pedodontics and Preventive Dentistry
Martins J.N.R.; Quaresma S.; Quaresma M.C.; Frisbie-Teel J.	C-shaped maxillary permanent first molar: A case report and literature Review	2013	Journal of Endodontics
Zhang Q, Ou YJ, Zhang L.	Curative effects of partial caries removal in permanent molars with deep dental caries	2017	Shanghai Kou Qiang Yi Xue
Surendran S.; Sivamurthy G.	Current applications and future prospects of stem cells in dentistry	2015	Dental Update
Dash S.; Jena D.; Jena S.P.	Current status and call for action: Regenerative endodontics	2019	Indian Journal of Public Health Research and Development
Mattos J, Soares GM, Ribeiro Ade A.	Current status of conservative treatment of deep carious lesions	2014	Dent Update
Anand M.; Karthikeyan K.; Sekar M.	Current trend of restoration of endodontically treated teeth with extensive subgingival caries: A case series	2022	Journal of Conservative Dentistry
Savani G.M.; Sabbah W.; Sedgley C.M.; Whitten B.	Current Trends in Endodontic Treatment by General Dental Practitioners: Report of a United States National Survey	2014	Journal of Endodontics
do Prado M.; Alencastro F.; Athias L.; de Lima C.O.; Marion J.J.C.; Simão R.A.; Campos C.N.	Current trends in irrigation among Brazilian endodontists	2018	Dental Press Endodontics
Malur MH, Chandra A.	Curvature height and distance of MB canal of mandibular molar with Schneider angle and its comparison with canal access angle	2018	J Oral Biol Craniofac Res
Scelza M.Z.; Iorio N.L.P.P.; Scelza P.; Póvoa H.C.C.; Adeodato C.S.R.; Souza A.C.N.; Batista A.C.; Tavares S.; Alves G.	Cytocompatibility and antimicrobial activity of a novel endodontic irrigant combining citric acid and chlorhexidine	2022	Journal of Dentistry
Rodríguez-Lozano F.J.; López-García S.; García-Bernal D.; Sanz J.L.; Lozano A.; Pecci-Lloret M.P.; Melo M.; López-Ginés C.; Forner L.	Cytocompatibility and bioactive properties of the new dual-curing resin-modified calcium silicate-based material for vital pulp therapy	2021	Clinical Oral Investigations
Elsalhy M, Azizieh F, Raghupathy R.	Cytokines as diagnostic markers of pulpal inflammation	2013	Int Endod J
Deluca M.C.D.C.; Scarparo R.K.; Aspesi M.; Matte B.F.; Brand L.M.; Grecca F.S.; Casagrande L.; Kopper P.M.P.	Cytotoxic, Migration, and Angiogenic Effects of Photodynamic Therapy and Photobiomodulation Associated with a Revascularization Protocol	2021	Journal of Endodontics
Kanjevac T.V.; Milovanović M.Z.; Milošević-Djordjević O.; Tešić Ž.; Ivanović M.; Lukić A.	Cytotoxicity of glass ionomer cement on human exfoliated deciduous teeth stem cells correlates with released fluoride, strontium and aluminum ion concentrations	2015	Archives of Biological Sciences
Cosme-Silva L.; Dos Santos A.F.; Lopes C.S.; Dal-Fabbro R.; Benetti F.; Gomes-Filho J.E.; Queiroz I.O.A.; Ervolino E.; Viola N.V.	Cytotoxicity, inflammation, biomineralization, and immunoexpression of il-1β and tnf-α promoted by a new bioceramic cement	2020	Journal of Applied Oral Science

Lee Y.-H.; Kim G.-E.; Song Y.-B.; Paudel U.; Lee N.-H.; Yun B.-S.; Yu M.-K.; Yi H.-K.	Davallialactone reduces inflammation and repairs dentinogenesis on glucose oxidase-induced stress in dental pulp cells	2013	Journal of Endodontics
Alqahtani Q.; Zaky S.H.; Patil A.; Beniash E.; Ray H.; Sfeir C.	Decellularized Swine Dental Pulp Tissue for Regenerative Root Canal Therapy	2018	Journal of Dental Research
Janani K., Ajitha P., Sandhya R.	Decision analysis for vital pulp therapy in mature permanent teeth	2020	Indian Journal of Public Health Research and Development
Ehlinger C.; Ginies E.; Bornert F.; Bahi-Gross S.; Schmittbuhl M.; Minoux M.	Decision criteria influencing the therapeutic approach to invasive cervical resorption: A case series	2019	Quintessence International
Scalzilli P.A.; Jara C.M.; Flores D.; Heinzmann D.; Figueiredo J.A.P.; Scarparo R.K.	Decision-making for dental pulp exposure: a survey in graduate programs at Brazilian universities	2022	Brazilian oral research
Katri C., Sari K.-S., Eero K.	Deep carious lesions and their management among Finnish adolescents: a retrospective radiographic study	2022	Clinical oral investigations
Tong H.J., Seremidi K., Stratigaki E., Kloukos D., Duggal M., Gizani S.	Deep dentine caries management of immature permanent posterior teeth with vital pulp: A systematic review and meta-analysis	2022	Journal of dentistry
Zhang X.; Zhu X.; Xie Z.	Deep learning in cone-beam computed tomography image segmentation for the diagnosis and treatment of acute pulpitis	2022	Journal of Supercomputing
Moskovitz M.; Tickotsky N.; Ashkar H.; Holan G.	Degree of root resorption after root canal treatment with iodoform-containing filling material in primary molars	2012	Quintessence International
Bertrand V., Dhenin C., Déchelotte P., Faerber M.	Delayed avoidant restrictive food intake disorder diagnosis leading to Ogilvie’s syndrome in an adolescent	2022	Eating and Weight Disorders
Ramazani M, Asgary S.	Delayed miniature pulpotomy in a symptomatic mature molar	2018	Dent Res J (Isfahan)
Timmerman A.; Parashos P.	Delayed Root Development by Displaced Mineral Trioxide Aggregate after Regenerative Endodontics: A Case Report	2017	Journal of Endodontics
Oliveira G.C.D.; Silva J.C.D.; Ionta F.Q.; Alencar C.R.B.D.; Gonçalves P.S.P.; Oliveira T.M.D.; Cruvinel T.; Rios D.	Delayed Treatment of Traumatized Primary Teeth with Distinct Pulp Response: Follow-Up until Permanent Successors Eruption	2017	Case Reports in Dentistry
Gandolfi M.G.; Taddei P.; Pondrelli A.; Zamparini F.; Prati C.; Spagnuolo G.	Demineralization, collagen modification and remineralization degree of human dentin after EDTA and citric acid treatments	2019	Materials
Liu Q.; Ma Y.; Wang J.; Zhu X.; Yang Y.; Mei Y.	Demineralized bone matrix used for direct pulp capping in rats	2017	PLoS ONE
Kularatna S.; Lalloo R.; Kroon J.; Tadakamadla S.K.K.; Scuffham P.A.; Johnson N.W.	Demonstration of high value care to improve oral health of a remote Indigenous community in Australia	2020	Health and Quality of Life Outcomes
Turner J.W.; Kluemper G.T.; Chance K.; Long L.S.	Dens evaginatus: The hornet's nest of adolescent orthodontics	2013	American Journal of Orthodontics and Dentofacial Orthopedics
Ickow I.M.; Zinn S.; Stacy J.M., Jr.; Martin B.; Losee J.E.; D’Alesio A.; Soxman J.; Schuster L.A.	Dens Invaginatus in Patients With Cleft Lip and Palate: A Case Series	2021	Cleft Palate-Craniofacial Journal
Siqueira JF Jr, Rôças IN, Hernández SR, Brisson-Suárez K, Baasch AC, Pérez AR, Alves FRF.	Dens Invaginatus: Clinical Implications and Antimicrobial Endodontic Treatment Considerations	2022	J Endod
Molina A, Bravo M, Fonseca GM, Márquez-Grant N, Martín-de-Las-Heras S.	Dental age estimation based on pulp chamber/crown volume ratio measured on CBCT images in a Spanish population	2021	Int J Legal Med
Asif MK, Nambiar P, Mani SA, Ibrahim NB, Khan IM, Sukumaran P.	Dental age estimation employing CBCT scans enhanced with Mimics software: Comparison of two different approaches using pulp/tooth volumetric analysis	2018	J Forensic Leg Med
Asif MK, Nambiar P, Mani SA, Ibrahim NB, Khan IM, Lokman NB.	Dental age estimation in Malaysian adults based on volumetric analysis of pulp/tooth ratio using CBCT data	2019	Leg Med (Tokyo)
Maho T, Reisz RR.	Dental anatomy and replacement patterns in the early Permian stem amniote, Seymouria	2022	J Anat
Šembera M.; Radochová V.; Slezák R.	Dental and Oral Lesions in HIV-Positive Individuals in East Bohemia--Czech Republic, Single Centre Experience	2015	Acta medica (Hradec Králové) / Universitas Carolina, Facultas Medica Hradec Králové

Tolibah Y.A., Kouchaji C., Lazkani T., Abbara M.T., Jbara S., Baghdadi Z.D.	Dental care for a child with congenital hydrocephalus: A case report with 12-month follow-up	2021	International Journal of Environmental Research and Public Health
Costa C.P.S.; Aires B.T.C.; Thomaz E.B.A.F.; Souza S.F.C.	Dental care provided to sickle cell anemia patients stratified by age: A population-based study in Northeastern Brazil	2016	European Journal of Dentistry
Yani R.W.E.; Nugroho H.S.W.	Dental caries based on dmf, sic and pufa index for people living in ampelan and ambulu village, bondowoso, indonesia	2018	Indian Journal of Public Health Research and Development
Kuzhalvaimozhi P., Ravindran V., Subhashini V.C.	Dental caries experience in patients with and without anterior crossbite: A case-control study	2020	International Journal of Research in Pharmaceutical Sciences
Baginska J.; Rodakowska E.; Milewski R.; Kierklo A.	Dental caries in primary and permanent molars in 7-8-year-old schoolchildren evaluated with Caries Assessment Spectrum and Treatment (CAST) index	2014	BMC Oral Health
Turska-Szybka A.; Soika I.; Rożniatowski P.; Gozdowski D.; Olczak-Kowalczyk D.	Dental caries severity and oral hygiene in warsaw preschool children at high risk for caries; Intensywność próchnicy i stan higieny jamy ustnej warszawskich dzieci w wieku przedszkolnym obciążonych dużym ryzykiem wystąpienia próchnicy	2017	Dental and Medical Problems
Mahajan P.	Dental caries status and treatment needs among Tibetan refugees residing in Shimla, Himachal Pradesh, India	2013	International Journal of Migration, Health and Social Care
Takriti M, Alhakim D, Splieth C.	Dental characteristics and according treatments of children under GA in Germany	2019	Eur Arch Paediatr Dent
Davit-Béal T.; Gabay J.; Antoniolli P.; Masle-Farquhar J.; Wolikow M.	Dental complications of rickets in early childhood: Case report on 2 young girls	2014	Pediatrics
Son K, Cho H, Kim H, Lee W, Cho M, Jeong H, Kim KH, Lee DH, Kim SY, Lee KB, Jeon M, Kim J.	Dental diagnosis for inlay restoration using an intraoral optical coherence tomography system: A case report	2023	J Prosthodont Res
Parewe A.M.A.K.; Mahmudy W.F.; Ramdhani F.; Anggodo Y.P.	Dental disease detection using hybrid fuzzy logic and evolution strategies	2018	Journal of Telecommunication, Electronic and Computer Engineering
Garispe A, Sorensen C, Sorensen JR.	Dental Emergencies	2023	StatPearls
Ferrer Y.M.; Collazo M.E.F.; Morales D.V.; Soto A.R.; González D.L.M.	Dental emergencies caused by pulpar lesions; Urgencias estomatológicas por lesiones pulpares	2012	Revista Cubana de Estomatologia
Alves I.S., Vendramini D.F.V., Leite C.C., Gebrim E.M.M.S., Passos U.L.	Dental findings on face and neck imaging	2021	Radiologia Brasileira
Hong H.; Chen X.; Li K.; Wang N.; Li M.; Yang B.; Yu X.; Wei X.	Dental follicle stem cells rescue the regenerative capacity of inflamed rat dental pulp through a paracrine pathway	2020	Stem Cell Research and Therapy
Brener R.; Zeitlin L.; Lebenthal Y.; Brener A.	Dental health of pediatric patients with X-linked hypophosphatemia (XLH) after three years of burosumab therapy	2022	Frontiers in Endocrinology
Siqueira V.D.S.; Castillo A.E.S.; Mateo-Castillo J.F.; Pinto L.D.C.; Garib D.; Pinheiro C.R.	Dental hypersensitivity in individuals with cleft lip and palate: Origin and therapies	2021	Journal of Dental Research, Dental Clinics, Dental Prospects
Abuabara A.; Schramm C.A.; Zielak J.C.; Baratto-Filho F.	Dental infection simulating skin lesion; Infecção dentária simulando uma lesão de pele	2012	Anais Brasileiros de Dermatologia
Gheorghiu I.-M., Perlea P., Mitran L., Iliescu A.A., Scarlatescu S., Suciu I., Mitran M.	Dental lesions and restorative treatment in molars	2017	ARS Medica Tomitana
Jain A.; Suprabha B.S.; Rao A.	Dental management of a child patient with facial palsy: A case report; Postępowanie stomatologiczne u dziecka z porażeniem nerwu twarzowego – Opis przypadku	2016	Dental and Medical Problems
Kameoka R., Kawakami T., Maeda M., Hori T., Yanagisawa A., Shirase T.	Dental management of a childhood cancer survivor with malformed primary teeth	2020	Pediatric Dental Journal
Alqarni M.A., Alharbi A., Merdad L.	Dental management of a patient with 22q11.2 deletion syndrome (22q11.2DS)	2018	BMJ Case Reports
Cheng F.-C.; Wang L.-H.; Ozawa N.; Wang C.-Y.; Chang J.Y.-F.; Chiang C.-P.	Dental manpower and treated dental diseases in department of dentistry, Taipei Hospital (the predecessor of National Taiwan University Hospital) in 1923	2022	Journal of Dental Sciences
Ha W.N.; Kahler B.; Walsh L.J.	Dental material choices for pulp therapy in paediatric dentistry	2017	European Endodontic Journal

Sherene Christina Roshini A.M., Babu N.A., Krupaa R.J., Masthan K.M.K.	Dental materials and its allergic reactions in dentistry— A review	2019	Indian Journal of Public Health Research and Development
Zhou Y.; Zheng L.; Zhou X.; Li J.; Xu X.	Dental mesenchymal stem cells in inflamed microenvironment: Potentials and challenges for regeneration	2015	Current Stem Cell Research and Therapy
Spiller L., Lukefahr J., Kellogg N.	Dental Neglect	2020	Journal of Child and Adolescent Trauma
Kenny K.	Dental pain: Highly prevalent and challenging	2016	Pharmacy Times
Miller HS, Avrahami HM, Zanno LE.	Dental pathologies in lamniform and carcharhiniform sharks with comments on the classification and homology of double tooth pathologies in vertebrates	2022	PeerJ
Inouye J.; McGrew C.	Dental problems in athletes	2015	Current Sports Medicine Reports
Stephens M.B.; Wiedemer J.P.; Kushner G.M.	Dental problems in primary care	2018	American Family Physician
Ibrahim N.A.; Azizi N.Z.; Mohd Nor N.A.	Dental procedures and operating time under day-care general anesthesia among medically compromised and uncooperative pediatric patients	2022	Quintessence International
Kovačič U, Tesovnik B, Molnar N, Cör A, Skalerič U, Gašperšič R.	Dental pulp and gingivomucosa in rats are innervated by two morphologically and neurochemically different populations of nociceptors	2013	Arch Oral Biol
Feitosa V.P.; Mota M.N.G.; Vieira L.V.; de Paula D.M.; Gomes L.L.R.; Solheiro L.K.R.; Aguiar Neto M.A.D.; Carvalho D.A.L.; Silvestre F.A.	Dental Pulp Autotransplantation: A New Modality of Endodontic Regenerative Therapy—Follow-Up of 3 Clinical Cases	2021	Journal of Endodontics
Kierdorf U.; Olsen M.T.; Kahle P.; Ludolphy C.; Kierdorf H.	Dental pulp exposure, periapical inflammation and suppurative osteomyelitis of the jaws in juvenile Baltic grey seals (Halichoerus grypus grypus) from the late 19th century	2019	PLoS ONE
Renard E.; Amiaud J.; Delbos L.; Charrier C.; Montembault A.; Ducret M.; Farges J.-C.; David L.; Alliot-Licht B.; Gaudin A.	Dental pulp inflammatory/immune response to a chitosan-enriched fibrin hydrogel in the pulpotomised rat incisor	2020	European Cells and Materials
Attar A.; Eslaminejad M.-B.; Tavangar M.-S.; Karamzadeh R.; Dehghani-Nazhvani A.; Ghahramani Y.; Malekmohammadi F.; Hosseini S.-M.	Dental pulp polyps contain stem cells comparable to the normal dental pulps	2014	Journal of Clinical and Experimental Dentistry
Caruso S.; Sgolastra F.; Gatto R.	Dental pulp regeneration in paediatric dentistry: The role of stem cells	2014	European Journal of Paediatric Dentistry
Gomez-Sosa J.F.; Diaz-Solano D.; Wittig O.; Cardier J.E.	Dental Pulp Regeneration Induced by Allogenic Mesenchymal Stromal Cell Transplantation in a Mature Tooth: A Case Report	2022	Journal of Endodontics
Kakarla P.; Avula J.S.S.; Mellela G.M.; Bandi S.; Anche S.	Dental pulp response to collagen and pulpotec cement as pulpotomy agents in primary dentition: A histological study	2013	Journal of Conservative Dentistry
Covaci A., Ciocan L.T., Gălbinașu B., Bucur M.V., Matei M., Didilescu A.C.	Dental Pulp Response to Different Types of Calcium-Based Materials Applied in Deep Carious Lesion Treatment—A Clinical Study	2022	Journal of Functional Biomaterials
Wang H.; Sun M.; Sun J.; Gong P.; Liu N.; Wang M.	Dental Pulp Stem Cell Therapy in Ischemic Stroke: A Meta-Analysis of Preclinical Studies	2022	Journal of Stroke and Cerebrovascular Diseases
Yoshimaru K.; Yamaza T.; Kajioka S.; Sonoda S.; Yanagi Y.; Matsuura T.; Yoshizumi J.; Oda Y.; Iwata N.; Takai C.; Nakayama S.; Taguchi T.	Dental pulp stem cells as a therapy for congenital entero-neuropathy	2022	Scientific Reports
Iezzi I.; Cerqueni G.; Licini C.; Lucarini G.; Mattioli Belmonte M.	Dental pulp stem cells senescence and regenerative potential relationship	2019	Journal of Cellular Physiology
Alsaeedi H.A.; Koh A.E.-H.; Lam C.; Rashid M.B.A.; Harun M.H.N.; Saleh M.F.B.M.; Teh S.W.; Luu C.D.; Ng M.H.; Isa H.M.; Leow S.N.; Then K.Y.; Bastion M.-L.C.; Mok P.L.; Muthuvenkatachalam B.S.; Samrot A.V.; Swamy K.B.; Nandakumar J.; Kumar S.S.	Dental pulp stem cells therapy overcome photoreceptor cell death and protects the retina in a rat model of sodium iodate-induced retinal degeneration	2019	Journal of Photochemistry and Photobiology B: Biology
Lan X.; Sun Z.; Chu C.; Boltze J.; Li S.	Dental pulp stem cells: An attractive alternative for cell therapy in ischemic stroke	2019	Frontiers in Neurology

Lambrichts I.; Driesen R.B.; Dillen Y.; Gervois P.; Ratajczak J.; Vangansewinkel T.; Wolfs E.; Bronckaers A.; Hilkens P.	Dental Pulp Stem Cells: Their Potential in Reinnervation and Angiogenesis by Using Scaffolds	2017	Journal of Endodontics
Luo L.; Xing Z.; Liao X.; Li Y.; Luo Y.; Ai Y.; He Y.; Ye Q.	Dental pulp stem cells-based therapy for the oviduct injury via immunomodulation and angiogenesis in vivo	2022	Cell Proliferation
Halperson E, Moss D, Tickotsky N, Weintraub M, Moskovitz M.	Dental pulp therapy for primary teeth in children undergoing cancer therapy	2014	Pediatr Blood Cancer
Ito T.; Kaneko T.; Sueyama Y.; Kaneko R.; Okiji T.	Dental pulp tissue engineering of pulpotomized rat molars with bone marrow mesenchymal stem cells	2017	Odontology
Giuroiu C.L.; Cəruntu I.-D.; Lozneanu L.; Melian A.; Vataman M.; Andrian S.	Dental pulp: Correspondences and contradictions between clinical and histological diagnosis	2015	BioMed Research International
Salim N.A.; ElSa'aideh B.B.; Maayta W.A.; Hassona Y.M.	Dental services provided to Syrian refugee children in Jordan: A retrospective study	2020	Special Care in Dentistry
Morón E.M.; Tomar S.L.; Souza R.; Balzer J.; Savioli C.; Shawkat S.	Dental Status and Treatment Needs of Children in Foster Care	2019	Pediatric dentistry
Yam G.H.-F.; Peh G.S.-L.; Singhal S.; Goh B.-T.; Mehta J.S.	Dental stem cells: A future asset of ocular cell therapy GARY HIN-KAI YAM, GARY SWEE-LIM PEH, SHWfcTA S1NGHAL\ BEE-UN GOH, JODHBIR S. MEHTA	2015	Expert Reviews in Molecular Medicine
Picart G, Pouhaër M, Dautel A, Pérard M, Le Clerc J.	Dental students' observations about teaching of endodontic access cavities in a French dental school	2022	Eur J Dent Educ
Martín-Jiménez M.; Martín-Biedma B.; López-López J.; Alonso-Ezpeleta O.; Velasco-Ortega E.; Jiménez-Sánchez M.C.; Segura-Egea J.J.	Dental students’ knowledge regarding the indications for antibiotics in the management of endodontic infections	2018	International Endodontic Journal
Picart G.; Pouhaër M.; Dautel A.; Pérard M.; Le Clerc J.	Dental students’ observations about teaching of endodontic access cavities in a French dental school	2022	European Journal of Dental Education
Falgás Franco J.	Dental trauma	2019	Pediatria Integral
Elkaiali L, Ratliff K, Oueis H.	Dental Treatment Considerations for Children with Complex Medical Histories: A Case of Townes-Brock Syndrome	2016	J Mich Dent Assoc
Peretz B, Spierer A, Spierer S, Rakocz M.	Dental treatment of patients with systemic diseases compared to patients with developmental disabilities under general anesthesia	2012	Spec Care Dentist
Loch C, Simões-Lopes PC.	Dental wear in dolphins (Cetacea: Delphinidae) from southern Brazil	2013	Arch Oral Biol
Jiang RD, Lin H, Zheng G, Yuan SP, DU Q, Zhang Y.	Dentin barrier cytotoxicity test with three-dimensional cell cultures	2015	Beijing Da Xue Xue Bao Yi Xue Ban
Sumidarti A.; Rovani C.A.; Nugroho J.J.; Thahir B.	Dentin matrix protein-1 (DMP-1) expression after application of haruan fish extract (channa striata) on inflamed wistar rat dental pulp	2020	Systematic Reviews in Pharmacy
Abd-Elmeguid A.; Yu D.C.; Kline L.W.; Moqbel R.; Vliagoftis H.	Dentin matrix protein-1 activates dental pulp fibroblasts	2012	Journal of Endodontics
Jayasree R, Kumar TSS, Mahalaxmi S, Abburi S, Rubaiya Y, Doble M.	Dentin remineralizing ability and enhanced antibacterial activity of strontium and hydroxyl ion co-releasing radiopaque hydroxyapatite cement	2017	J Mater Sci Mater Med
Chuang S.-F.; Chen Y.-H.; Ma P.X.; Ritchie H.H.	Dentin Sialoprotein/Phosphophoryn (DSP/PP) as Bio-Inductive Materials for Direct Pulp Capping	2022	Polymers
Martín-De-Llano J.J.; Mata M.; Peydró S.; Peydró A.; Carda C.	Dentin tubule orientation determines odontoblastic differentiation in vitro: A morphological study	2019	PLoS ONE
Ranjitkar S.; Yong R.; Wu I.-C.; Gully G.; Farmer D.; Watson I.; Heithersay G.	Dentinal dysplasia type 1: A 3D micro-computed tomographic study of enamel, dentine and root canal morphology	2019	Australian Endodontic Journal
Gupta R., Patil S., Mohite P., Gupta D., Dudulwar D.	Dentinal Microcrack Formation by Different Rotary Endodontic File Systems: An In-vitro Study	2022	Journal of Clinical and Diagnostic Research
Kenchappa M.; Gupta S.; Gupta P.; Sharma P.	Dentine in a capsule: Clinical case reports	2015	Journal of Indian Society of Pedodontics and Preventive Dentistry
Guirado E., George A.	DENTINE MATRIX METALLOPROTEINASES AS POTENTIAL MEDIATORS OF DENTINE REGENERATION	2021	European Cells and Materials
Okamoto M., Takahashi Y., Komichi S., Cooper P.R., Hayashi M.	Dentinogenic effects of extracted dentin matrix components digested with matrix metalloproteinases	2018	Scientific reports
Tziafa C.; Koliniotou-Koumpia E.; Papadimitriou S.; Tziafas D.	Dentinogenic responses after direct pulp capping of miniature swine teeth with Biodentine	2014	Journal of Endodontics

Tziafas D.; Kodonas K.	Dentinogenic specificity in the preclinical evaluation of vital pulp treatment strategies: A critical review	2015	Dentistry Journal
Yan H, Oshima M, Raju R, Raman S, Sekine K, Waskitho A, Inoue M, Inoue M, Baba O, Morita T, Miyagi M, Matsuka Y.	Dentin-Pulp Complex Tissue Regeneration via Three-Dimensional Cell Sheet Layering	2021	Tissue Eng Part C Methods
Subekti A.; Subinarto; Sariyem; Ningtyas E.A.E.	Dentist expert system software in dental caries detection	2017	Advanced Science Letters
Crespo-Gallardo I, Martín-González J, Jiménez-Sánchez MC, Cabanillas-Balsera D, Sánchez-Domínguez B, Segura-Egea JJ.	Dentist´s knowledge, attitudes and determining factors of the conservative approach in teeth with reversible pulpitis and deep caries lesions	2018	J Clin Exp Dent
Li M.; Hu X.; Li X.; Lei S.; Cai M.; Wei X.; Deng D.	Dentist-related factors influencing the use of vital pulp therapy: a survey among dental practitioners in China	2019	Journal of International Medical Research
Schwendicke F, Stangvaltaite L, Holmgren C, Maltz M, Finet M, Elhennawy K, Eriksen I, Kuzmiszyn TC, Kerosuo E, Doméjean S.	Dentists' attitudes and behaviour regarding deep carious lesion management: a multi-national survey	2017	Clin Oral Investig
Crespo-Gallardo I.; Martín-González J.; Jiménez-Sánchez M.C.; Cabanillas-Balsera D.; Sánchez-Domínguez B.; Segura-Egea J.J.	Dentists knowledge, attitudes and determining factors of the conservative approach in teeth with reversible pulpitis and deep caries lesions	2018	Journal of Clinical and Experimental Dentistry
Kakudate N, Yokoyama Y, Sumida F, Matsumoto Y, Gordan VV, Gilbert GH.	Dentists' practice patterns of treatment for deep occlusal caries: Findings from a dental practice-based research network	2019	J Dent
Garrido BDTM, Vitor LLR, Cruvinel T, Machado MAAM, Oliveira TM, Lourenço Neto N.	Dentists' Self-evaluated Ability in Diagnosing and Updating About Pulpotomy	2023	Int Dent J
Kakudate N.; Yokoyama Y.; Sumida F.; Matsumoto Y.; Gordan V.V.; Gilbert G.H.	Dentists' practice patterns of treatment for deep occlusal caries: Findings from a dental practice-based research network	2019	Journal of Dentistry
Bjørndal L, Demant S, Dabelsteen S.	Depth and activity of carious lesions as indicators for the regenerative potential of dental pulp after intervention	2014	J Endod
Bitner D.P.; Feldman D.U.; Axx K.; Albandar J.M.	Description and evaluation of an intraoral cervical plexus anesthetic technique	2015	Clinical Anatomy
Ajayi YO, Sofola OO.	Descriptors of permanent teeth with cariously exposed pulp in patients presenting at a Nigerian hospital	2013	Acta Odontol Scand
Pouhaër M., Picart G., Baya D., Michelutti P., Dautel A., Pérard M., Le Clerc J.	Design of 3D-printed macro-models for undergraduates' preclinical practice of endodontic access cavities	2022	European journal of dental education : official journal of the Association for Dental Education in Europe
Ogaya Y.; Nomura R.; Nakano K.; Watanabe Y.	Detection of Helicobacter pylori DNA in inflamed dental pulp specimens from japanese Children and adolescents	2015	Journal of Medical Microbiology
Yuce F, Öziç MÜ, Tassoker M.	Detection of pulpal calcifications on bite-wing radiographs using deep learning	2023	Clin Oral Investig
Lima A.R.; Herrera D.R.; Francisco P.A.; Pereira A.C.; Lemos J.; Abranches J.; Gomes B.P.F.A.	Detection of Streptococcus mutans in symptomatic and asymptomatic infected root canals	2021	Clinical Oral Investigations
Contreras C, Cádiz B, Schmachtenberg O.	Determination of the Severity of Pulpitis by Immunohistological Analysis and Comparison with the Clinical Picture	2023	J Endod
Park K, Ahn J, Kang S, Lee E, Kim S, Park S, Park S, Noh H, Seo K.	Determining the age of cats by pulp cavity/tooth width ratio using dental radiography	2014	J Vet Sci
Anitasari S.; Wahab D.E.; Barlianta B.; Budi H.S.	Determining the effectivity of infrared distance to eliminate dental pain due to pulpitis and periodontitis	2020	European Journal of Dentistry
El-Kishawi M.Y., Khalaf K., Odeh R.M.	Determining the impact of stressors on students' clinical performance in endodontics	2021	Journal of Taibah University Medical Sciences
Doğan S.; Durutürk L.; Orhan A.I.; Batmaz I.	Determining treatibility of primary teeth with pulpal exposure	2013	Journal of Clinical Pediatric Dentistry
Klein-Júnior C.A., Reston E., Plepis A.M., Martins V.C., Pötter I.C., Lundy F., Hentschke G.S., Hentschke V.S., Karim I.E.	Development and evaluation of calcium hydroxide-coated, pericardium-based biomembranes for direct pulp capping	2019	Journal of Investigative and Clinical Dentistry

Moyer JK, Riccio ML, Bemis WE.	Development and microstructure of tooth histotypes in the blue shark, Prionace glauca (Carcharhiniformes: Carcharhinidae) and the great white shark, Carcharodon carcharias (Lamniformes: Lamnidae)	2015	J Morphol
Smaïl-Faugeron V, Fron Chabouis H, Durieux P, Attal JP, Muller-Bolla M, Courson F.	Development of a core set of outcomes for randomized controlled trials with multiple outcomes--example of pulp treatments of primary teeth for extensive decay in children	2013	PLoS One
Okoro C, Vartanian A, Toussaint KC Jr.	Development of a handheld smart dental instrument for root canal imaging	2016	J Biomed Opt
Yoneda N., Noiri Y., Matsui S., Kuremoto K., Maezono H., Ishimoto T., Nakano T., Ebisu S., Hayashi M.	Development of a root canal treatment model in the rat	2017	Scientific reports
Roberts J.L.; Maillard J.-Y.; Waddington R.J.; Denyer S.P.; Lynch C.D.; Sloan A.J.	Development of an ex vivo coculture system to model pulpal infection by streptococcus anginosus group bacteria	2013	Journal of Endodontics
Chang H.-H.; Chang Y.-J.; Yeh C.-L.; Lin T.-A.; Lin C.-P.	Development of calcium phosphate/calcium sulfate biphasic biomedical material with hyaluronic acid containing collagenase and simvastatin for vital pulp therapy	2020	Dental Materials
Chang K.-C.; Chang C.-C.; Chen W.-T.; Hsu C.-K.; Lin F.-H.; Lin C.-P.	Development of calcium phosphate/sulfate biphasic cement for vital pulp therapy	2014	Dental Materials
Alagha A.; Nourallah A.; Alhariri S.	Dexamethasone- loaded polymeric porous sponge as a direct pulp capping agent	2020	Journal of Biomaterials Science, Polymer Edition
Hussien R.M.; Emam D.F.; Shoukry A.A.	Dexmedetomidine compared to ketofol for sedation in pediatric patients undergoing dental procedures: a double-blind, randomized clinical trial	2022	Anaesthesia, Pain and Intensive Care
Lima S.M.F.; Grisi D.C.; Kogawa E.M.; Franco O.L.; Peixoto V.C.; Gonçalves-Júnior J.F.; Arruda M.P.; Rezende T.M.B.	Diabetes mellitus and inflammatory pulpal and periapical disease: A review	2013	International Endodontic Journal
Macho Á.Z.; Ferreiroa A.; Rico-Romano C.; Alonso-Ezpeleta L.Ó.; Mena-Álvarez J.	Diagnosis and endodontic treatment of type II dens invaginatus by using cone-beam computed tomography and splint guides for cavity access: A case report	2015	Journal of the American Dental Association
Alshaya M.S., Sabbagh H.J., El-Housseiny A.A.	Diagnosis and Management Approaches for Non-cavitated Carious Dental Lesions-A Narrative Review	2021	Open Dentistry Journal
Moe J.; Rajan R.; Caltharp S.; Abramowicz S.	Diagnosis and Management of Children With Mycobacterium abscessus Infections in the Head and Neck	2018	Journal of Oral and Maxillofacial Surgery
Tanwir F, Marrone G, Tariq A, Lundborg CS.	Diagnosis and prescribing pattern of antibiotics and painkillers among dentists	2015	Oral Health Prev Dent
Vigil A.; Bharathi S.	Diagnosis of pulpitis from dental panoramic radiograph using histogram of gradients with discrete wavelet transform and multilevel neural network techniques	2021	Traitement du Signal
Mejàre I.A.; Axelsson S.; Davidson T.; Frisk F.; Hakeberg M.; Kvist T.; Norlund A.; Petersson A.; Portenier I.; Sandberg H.; Tranæus S.; Bergenholtz G.	Diagnosis of the condition of the dental pulp: A systematic review	2012	International Endodontic Journal
Janani K.; Palanivelu A.; Sandhya R.	Diagnostic accuracy of dental pulse oximeter with customized sensor holder, thermal test and electric pulp test for the evaluation of pulp vitality: An in vivo study; Precisão diagnóstica do oxímetro de pulso odontológico com suporte de sensor personalizado, teste térmico e teste elétrico da polpa para avaliação da vitalidade pulpar: Um estudo in vivo	2020	Brazilian Dental Science
Pourhajibagher M.; Bahador A.	Diagnostic accuracy of multiplex real-time PCR approaches compared with cultivation -based detection methods: Monitoring the endopathogenic microbiota pre and post photo-activated disinfection	2018	Photodiagnosis and Photodynamic Therapy
Sousa TO, Haiter-Neto F, Nascimento EHL, Peroni LV, Freitas DQ, Hassan B.	Diagnostic Accuracy of Periapical Radiography and Cone-beam Computed Tomography in Identifying Root Canal Configuration of Human Premolars	2017	J Endod
Maserat V., Ebrahimi H.S., Saberi E.A., Pirhaji A., Khosravii N.	Diagnostic accuracy of two conebeam computed tomography systems for detection of strip perforation in the mesial root of mandibular molars	2020	Giornale Italiano di Endodonzia
Chen M.; Zeng J.; Yang Y.; Wu B.	Diagnostic biomarker candidates for pulpitis revealed by bioinformatics analysis of merged microarray gene expression datasets	2020	BMC Oral Health

Mariona R.P., Antony S.D.P.	Diagnostic methods for cracked tooth by two endodontic tools	2018	Drug Invention Today
Sharma V., Gupta N., Srivastava N., Rana V., Chandna P., Yadav S., Sharma A.	Diagnostic potential of inflammatory biomarkers in early childhood caries - A case control study	2017	Clinica Chimica Acta
Pasini M.; Giuca M.R.; Gatto R.; Caruso S.	Difference of Success Rates of Mineral Trioxide Aggregate Pulpotomies Performed Both by Undergraduate Dental Students and by an Expert Operator: A Retrospective Study	2017	Scientific World Journal
ATES A.A.; ALOMARI T.; BHARDWAJ A.; TABNJH A.; GAMBARINI G.	Differences in endodontic emergency management by endodontists and general dental practitioners in COVID-19 times	2020	Brazilian Oral Research
Lan C.; Chen S.; Jiang S.; Lei H.; Cai Z.; Huang X.	Different expression patterns of inflammatory cytokines induced by lipopolysaccharides from Escherichia coli or Porphyromonas gingivalis in human dental pulp stem cells	2022	BMC Oral Health
Jose J.; Ajitha P.; Subbaiyan H.	Different treatment modalities followed by dental practitioners for ellis class 2 fracture – a questionnaire-based survey	2020	Open Dentistry Journal
Kim S.-Y.; Kim S.-H.; Cho S.-B.; Lee G.-O.; Yang S.-E.	Different treatment protocols for different pulpal and periapical diagnoses of 72 cracked teeth	2013	Journal of Endodontics
Germain L.	Differential diagnosis of toothache pain. Part I, odontogenic etiologies.	2012	Dentistry today
Rothermund K.; Calabrese T.C.; Syed-Picard F.N.	Differential Effects of Escherichia coli– Versus Porphyromonas gingivalis–derived Lipopolysaccharides on Dental Pulp Stem Cell Differentiation in Scaffold-free Engineered Tissues	2022	Journal of Endodontics
Huang X.; Chen K.	Differential Expression of Long Noncoding RNAs in Normal and Inflamed Human Dental Pulp	2018	Journal of Endodontics
Zhong S.; Zhang S.; Bair E.; Nares S.; Khan A.A.	Differential expression of MicroRNAs in normal and inflamed human pulps	2012	Journal of Endodontics
Yue W.; Kim S.; Jung H.-S.; Lee J.-M.; Lee S.; Kim E.	Differential protein expression in human dental pulp: Comparison of healthy, inflamed, and traumatic pulp	2019	Journal of Clinical Medicine
Ali M.R.W.; Mustafa M.; Bårdsen A.; Gharaei M.A.; Fristad I.; Bletsa A.	Differential Responses of Human Dental Pulp Stromal Cells to Bioceramic Materials: A Comparative In Vitro Study	2021	Journal of Contemporary Dental Practice
Lim MJ, Kim JA, Choi Y, Hong CU, Min KS.	Differentiating spontaneous vertical root fracture in endodontically treated tooth	2017	Eur J Dent
Zhang L.; Yu Y.; Joubert C.; Bruder G.; Liu Y.; Chang C.-C.; Simon M.; Walker S.G.; Rafailovich M.	Differentiation of dental pulp stem cells on Gutta-percha scaffolds	2016	Polymers
Gonmanee T.; Thonabulsombat C.; Vongsavan K.; Sritanaudomchai H.	Differentiation of stem cells from human deciduous and permanent teeth into spiral ganglion neuron-like cells	2018	Archives of Oral Biology
Alex G.	Direct and Indirect Pulp Capping: A Brief History, Material Innovations, and Clinical Case Report	2018	Compend Contin Educ Dent
Kanada S.; Makino E.; Nakamura N.; Miyabe M.; Ito M.; Hata M.; Yamauchi T.; Sawada N.; Kondo S.; Saiki T.; Minato T.; Miyazawa K.; Goto S.; Matsubara T.; Naruse K.	Direct comparison of therapeutic effects on diabetic polyneuropathy between transplantation of dental pulp stem cells and administration of dental pulp stem cell-secreted factors	2020	International Journal of Molecular Sciences
Ali H, Raslan N.	Direct pulp capping (DPC) in primary molars using (3Mix-MP) and the characteristics of the carious lesion as predictor factors for its success: a randomized controlled trial	2021	Eur Arch Paediatr Dent
Chatzidimitriou K, Vadiakas G, Koletsi D.	Direct pulp capping in asymptomatic carious primary molars using three different pulp capping materials: a prospective clinical trial	2022	Eur Arch Paediatr Dent
Kotsanos N, Arapostathis KN, Arhakis A, Menexes G.	Direct pulp capping of carious primary molars. A specialty practice based study	2014	J Clin Pediatr Dent
Friedlander L.; Mcelroy K.; Daniel B.; Cullinan M.; Hanlin S.	Direct pulp capping of permanent teeth in New Zealand general dental practice- A practice based research study	2015	New Zealand Dental Journal
Dimitraki D.; Papageorgiou S.N.; Kotsanos N.	Direct pulp capping versus pulpotomy with MTA for carious primary molars: a randomised clinical trial	2019	European Archives of Paediatric Dentistry
Brodén J, Heimdal H, Josephsson O, Fransson H.	Direct pulp capping versus root canal treatment in young permanent vital teeth with pulp exposure due to caries. A systematic review	2016	Am J Dent

Ahlawat M, Grewal MS, Goel M, Bhullar HK, Saurabh, Nagpal R.	Direct Pulp Capping with Mineral Trioxide Aggregate and Biodentine in Cariously Exposed Molar Teeth: 1-Year Follow-up - An In vivo Study	2022	J Pharm Bioallied Sci
Ahlawat M., Grewal M.S., Goel M., Bhullar H.K., Saurabh, Nagpal R.	Direct pulp capping with mineral trioxide aggregate and biodentine in cariously exposed molar teeth: 1-year follow-up-An in vivo study	2022	Journal of Pharmacy and Bioallied Sciences
Kermanshah H, Ranjbar Omrani L, Ghabraei S, Fekrazad R, Daneshparvar N, Bagheri P.	Direct Pulp Capping With ProRoot MTA Alone and in Combination With Er:YAG Laser Irradiation: A Clinical Trial	2020	J Lasers Med Sci
Paula A.; Carrilho E.; Laranjo M.; Abrantes A.M.; Casalta-Lopes J.; Botelho M.F.; Marto C.M.; Ferreira M.M.	Direct pulp capping: Which is the most effective biomaterial? A retrospective clinical study	2019	Materials
Bosch B.M.; Salero E.; Núñez-Toldrà R.; Sabater A.L.; Gil F.J.; Perez R.A.	Discovering the Potential of Dental Pulp Stem Cells for Corneal Endothelial Cell Production: A Proof of Concept	2021	Frontiers in Bioengineering and Biotechnology
Cankar K.; Nemeth L.; Bajd F.; Vidmar J.; Serša I.	Discrimination between intact and decayed pulp regions in carious teeth by ADC mapping	2014	Caries Research
Khaladkar S.M., Reddy B.N.	Displaced root of 3(rd)maxillary molar tooth into infratemporal fossa- role of 3D CT scan	2020	Journal of Clinical and Diagnostic Research
Kim J.-H.; Jeon M.; Song J.-S.; Lee J.-H.; Choi B.-J.; Jung H.-S.; Moon S.J.; DenBesten P.K.; Kim S.-O.	Distinctive genetic activity pattern of the human dental pulp between deciduous and permanent teeth	2014	PLoS ONE
Moraes LC, Só MV, Dal Pizzol Tda S, Ferreira MB, Montagner F.	Distribution of genes related to antimicrobial resistance in different oral environments: a systematic review	2015	J Endod
Shrestha R.; Srii R.; Shrestha D.	Diversity of root canal morphology in mandibular first premolar	2019	Kathmandu University Medical Journal
Wang X.; Feng Z.; Li Q.; Yi B.; Xu Q.	DNA methylcytosine dioxygenase ten-eleven translocation 2 enhances lipopolysaccharide-induced cytokine expression in human dental pulp cells by regulating MyD88 hydroxymethylation	2018	Cell and Tissue Research
Nowak A.J.; Casamassimo P.S.; Scott J.; Moulton R.	Do early dental visits reduce treatment and treatment costs for children?	2014	Pediatric Dentistry
Fowler S.; Fullmer S.; Drum M.; Reader A.	Does acetaminophen/hydrocodone affect cold pulpal testing in patients with symptomatic irreversible pulpitis? A prospective, randomized, double-blind, placebo-controlled study	2014	Journal of Endodontics
da Silva B.S., Nicoloso G.F., Ruiz L.F., de Melo T.A.F., Casagrande L.	Does endodontic Re-treatment in primary teeth increase the functional tooth retention? A clinical, retrospective, university-based study	2019	Pesquisa Brasileira em Odontopediatria e Clinica Integrada
Vitor L.L.R.; Prado M.T.O.; Lourenço Neto N.; Oliveira R.C.; Sakai V.T.; Santos C.F.; Dionísio T.J.; Rios D.; Cruvinel T.; Machado M.A.A.M.; Oliveira T.M.	Does photobiomodulation change the synthesis and secretion of angiogenic proteins by different pulp cell lineages?	2020	Journal of Photochemistry and Photobiology B: Biology
Kuharattanachai K, Rangsri W, Jotikasthira D, Khemaleelakul W, Tripuwabhrut K.	Does pulp cavity affect the center of resistance in three-dimensional tooth model? A finite element method study	2022	Clin Oral Investig
Rabello D.G.D.; Corazza B.J.M.; Ferreira L.L.; Santamaria M.P.; Gomes A.P.M.; Martinho F.C.	Does supplemental photodynamic therapy optimize the disinfection of bacteria and endotoxins in one-visit and two-visit root canal therapy? A randomized clinical trial	2017	Photodiagnosis and Photodynamic Therapy
Franceschi D.; Prato G.P.P.; Gianfilippo R.D.	Double Connective Tissue Graft to Treat Deep Coronal-Radicular Abrasion: A 19-Year Follow-Up Case Report	2021	Clinical advances in periodontics
Ke Z.; Qiu Z.; Xiao T.; Zeng J.; Zou L.; Lin X.; Hu X.; Lin S.; Lv H.	Downregulation of miR-224-5p promotes migration and proliferation in human dental pulp stem cells	2019	BioMed Research International
Lee S.; Zhang Q.Z.; Karabucak B.; Le A.D.	DPSCs from inflamed pulp modulate macrophage function via the TNF-α/IDO axis	2016	Journal of Dental Research
Al-Rashdi M.S.; Bakathir A.; Al Balushi K.A.	Drug prescribing practices in dental care patients at a dental and maxillofacial surgery clinic in Oman	2020	Oman Medical Journal
Birjandi AA, Suzano FR, Sharpe PT.	Drug Repurposing in Dentistry; towards Application of Small Molecules in Dentin Repair	2020	Int J Mol Sci
Huang C.-C., Narayanan R., Warshawsky N., Ravindran S.	Dual ECM biomimetic scaffolds for dental pulp regenerative applications	2018	Frontiers in Physiology
Dai Y.; Xuan G.; Yin M.	DUXAP8 Promotes LPS-Induced Cell Injury in Pulpitis by Regulating miR-18b-5p/HIF3A	2022	International Dental Journal

Marugina T.L.; Levenets A.A.; Kiprin D.V.; Cherevatenko A.I.	Dynamic evaluation of the effectiveness of the use of sealers in the preparation of root canals for orthopedic restoration of teeth	2020	Siberian Journal of Life Sciences and Agriculture
Kwon SR, Li Y, Oyoyo U, Aprecio RM.	Dynamic model of hydrogen peroxide diffusion kinetics into the pulp cavity	2012	J Contemp Dent Pract
Leontiev W, Connert T, Weiger R, Krastl G, Magni E.	Dynamic Navigation in Endodontics: Guided Access Cavity Preparation by Means of a Miniaturized Navigation System	2022	J Vis Exp
Huang J.; Lv Y.; Fu Y.; Ren L.; Wang P.; Liu B.; Huang K.; Bi J.	Dynamic Regulation of Delta-Opioid Receptor in Rat Trigeminal Ganglion Neurons by Lipopolysaccharide-induced Acute Pulpitis	2015	Journal of Endodontics
Papi P., Pranno N., Di Murro B., Pompa G.	Early implant placement and peri-implant augmentation with a porcine-derived acellular dermal matrix and synthetic bone in the aesthetic area: a 2-year follow-up prospective cohort study	2021	International Journal of Oral and Maxillofacial Surgery
Paudel U.; Lee Y.-H.; Kwon T.-H.; Park N.-H.; Yun B.-S.; Hwang P.-H.; Yi H.-K.	Eckols reduce dental pulp inflammation through the ERK1/2 pathway independent of COX-2 inhibition	2014	Oral Diseases
Takahashi N, Nyvad B.	Ecological Hypothesis of Dentin and Root Caries	2016	Caries Res
Deery C.	Editorial: is there a place for the formocresol pulpotomy? Indirect pulp capping, the real alterative	2014	Int J Paediatr Dent
Chu T.; Ni X.; Zhu Y.	EDTA Combined with C-Pilot Files and Microultrasound for Root Canal Calcification: Dredging Effect and Safety Analysis	2022	Computational and Mathematical Methods in Medicine
Tziafas D, Kodonas K, Gogos C, Tziafa C, Papadimitriou S.	EDTA conditioning of circumpulpal dentine induces dentinogenic events in pulpotomized miniature swine teeth	2019	Int Endod J
Martínez-Herrera A.; Pozos-Guillén A.; Ruiz-Rodríguez S.; Garrocho-Rangel A.; Vértiz-Hernández A.; Escobar-García D.M.	Effect of 4-Allyl-1-hydroxy-2-methoxybenzene (Eugenol) on Inflammatory and Apoptosis Processes in Dental Pulp Fibroblasts	2016	Mediators of Inflammation
Selvan P.; Malathi; Rajan R.	Effect of 4-allyl-2-methoxyphenol (eugenol) on motor co-ordination in subacute restraint stress induced wistar albino rats	2016	Journal of Applied Pharmaceutical Science
Al-Shamma A.M.W.; Al-Hijazi A.Y.; Hasan D.M.	Effect of A 1 in Traumatic Pulp of Osteoporotic Rat	2020	Systematic Reviews in Pharmacy
Edanami N.; Ibn Belal R.S.; Yoshiba K.; Yoshiba N.; Ohkura N.; Takenaka S.; Noiri Y.	Effect of a resin-modified calcium silicate cement on inflammatory cell infiltration and reparative dentin formation after pulpotomy in rat molars	2022	Australian Endodontic Journal
Gulzar R.; Ajitha P.; Subbaiyan H.	Effect of addition of bismuth oxide, zirconium oxide nanoparticles and niobium oxide nanoparticles to portland cement on the proliferation and migration of dental pulp stem cells	2021	International Journal of Dentistry and Oral Science
Yousaf A., Ali F., Bhangar F., Alam M.	Effect of apical patency on postoperative pain after single-visit endodontic treatment in necrotic teeth with asymptomatic apical periodontitis: A randomised control trial	2021	Journal of the College of Physicians and Surgeons Pakistan
BinMahfooz A.M.; Sindi M.A.; Alsohaibi T.H.; Jabbad H.H.; Johar A.O.	Effect of Apical Root Canal Perforation Size on Push-out Bond Strength of Glass Fiber Dowels	2020	Journal of Contemporary Dental Practice
Wang L., Xie X., Weir M.D., Fouad A.F., Zhao L., Xu H.H.K.	Effect of bioactive dental adhesive on periodontal and endodontic pathogens	2016	Journal of Materials Science: Materials in Medicine
Washio A.; Miura H.; Morotomi T.; Ichimaru-Suematsu M.; Miyahara H.; Hanada-Miyahara K.; Yoshii S.; Murata K.; Takakura N.; Akao E.; Fujimoto M.; Matsuyama A.; Kitamura C.	Effect of bioactive glass-based root canal sealer on the incidence of postoperative pain after root canal obturation	2020	International Journal of Environmental Research and Public Health
Mushtaq A.; Goswami M.; Rahman B.; Sharan S.	Effect of calcium silicate cements on vascular endothelial growth factor release from platelet-rich fibrin and its architectural changes	2021	World Journal of Dentistry
Sharma P.; Garg S.; Dhindsa A.; Jain N.; Joshi S.; Gupta A.	Effect of chlorhexidine gluconate as hemostatic agent in healing and repair after mineral trioxide aggregate vital pulp therapy in young permanent teeth - A clinical study	2021	Indian Journal of Physiology and Pharmacology
Raoof M.; Ashrafganjoui E.; Kooshki R.; Abbasnejad M.; Haghani J.; Amanpour S.; Zarei M.-R.	Effect of chronic stress on capsaicin-induced dental nociception in a model of pulpitis in rats	2018	Archives of Oral Biology
Minhoto GB, Khoury RD, Orozco EIF, Prado RF, Valera MC.	Effect of chronic unpredictable stress on the progression of experimental apical periodontitis in rats	2021	Int Endod J

Kirmizibekmez Ö., Karataş E., Topçuoğlu H.S.	Effect of continuous irrigation on apical transportation, centering ability and volume of removed dentin in curved root canals: a micro computed tomography study	2022	Giornale Italiano di Endodonzia
Shi X.; Li Z.; He Y.; Jiang Q.; Yang X.	Effect of different dental burs for experimental induction of pulpitis in mice	2017	Archives of Oral Biology
Lai H.; Lin X.; Zhang Y.; Gong Q.; Tong Z.	Effect of different endodontic access preparations on the biomechanical behavior of lithium disilicate and resin nanoceramic onlay restorations: An in vitro and 3D finite element analysis study	2022	Journal of Prosthetic Dentistry
Bhandi S.; Patil S.; Boreak N.; Chohan H.; Abumelha A.S.; Alkahtany M.F.; Almadi K.H.; Vinothkumar T.S.; Raj A.T.; Testarelli L.	Effect of Different Intracanal Medicaments on the Viability and Survival of Dental Pulp Stem Cells	2022	Journal of Personalized Medicine
Al Qahtani M., Haralur S.B., Alqahtani M.A., Assiri A.K., Alqahtani A.S.	Effect of different surface treatment on the push out bond strength of polyether ether ketone endodontic post	2018	Journal of Biomaterials and Tissue Engineering
Chang C.-C.; Yeh C.-L.; Chang H.-H.; Kuo Y.-F.; Huang P.-Y.; Lin C.-P.	Effect of different zinc concentrations on partially-stabilized cement for vital pulp therapy	2019	Journal of the Formosan Medical Association
Falkensammer F.; Schaden W.; Krall C.; Freudenthaler J.; Bantleon H.-P.	Effect of extracorporeal shockwave therapy (ESWT) on pulpal blood flow after orthodontic treatment: a randomized clinical trial	2016	Clinical Oral Investigations
Sarra G.; Machado M.E.D.L.; Caballero-Flores H.V.; Moreira M.S.; Pedroni A.C.F.; Marques M.M.	Effect of human dental pulp stem cell conditioned medium in the dentin-pulp complex regeneration: A pilot in vivo study	2021	Tissue and Cell
Read J.K.; McClanahan S.B.; Khan A.A.; Lunos S.; Bowles W.R.	Effect of ibuprofen on masking endodontic diagnosis	2014	Journal of Endodontics
Alshatrat S.M.; Sabarini J.M.; Hammouri H.M.; Al-Bakri I.A.; Al-Omari W.M.	Effect of immersive virtual reality on pain in different dental procedures in children: A pilot study	2022	International Journal of Paediatric Dentistry
Farhad A.; Razavian H.; Shafiee M.	Effect of intraosseous injection versus inferior alveolar nerve block as primary pulpal anaesthesia of mandibular posterior teeth with symptomatic irreversible pulpitis: a prospective randomized clinical trial	2018	Acta Odontologica Scandinavica
Kwon SR, Oyoyo U, Li Y.	Effect of light activation on tooth whitening efficacy and hydrogen peroxide penetration: an in vitro study	2013	J Dent
de Santana D.A.; Fonseca G.F.; Ramalho L.M.P.; Rodriguez T.T.; Aguiar M.C.	Effect of low-level laser therapy (λ780 nm) on the mechanically damaged dentin-pulp complex in a model of extrusive luxation in rat incisors	2017	Lasers in Medical Science
Vera J.; Hernández E.M.; Romero M.; Arias A.; Van Der Sluis L.W.M.	Effect of maintaining apical patency on irrigant penetration into the apical two millimeters of large root canals: An in vivo study	2012	Journal of Endodontics
Olatosi O.O., Sote E.O., Orenuga O.O.	Effect of mineral trioxide aggregate and formocresol pulpotomy on vital primary teeth: a clinical and radiographic study	2015	Nigerian journal of clinical practice
Liu L.; Shu S.; Cheung G.S.; Wei X.	Effect of miR-146a/bFGF/PEG-PEI Nanoparticles on Inflammation Response and Tissue Regeneration of Human Dental Pulp Cells	2016	BioMed Research International
Shafiei F, Dehghani Z, Tavangar MS.	Effect of natural antioxidants on bond strength recovery of resin-modified glass ionomers to the NaOCl-affected pulp chamber dentin	2023	Clin Exp Dent Res
Stanley W.; Drum M.; Nusstein J.; Reader A.; Beck M.	Effect of nitrous oxide on the efficacy of the inferior alveolar nerve block in patients with symptomatic irreversible pulpitis	2012	Journal of Endodontics
Ribeiro-Santos F.R.; Arnez M.F.M.; de Carvalho M.S.; da Silva R.A.B.; Politi M.P.L.; de Queiroz A.M.; Nelson-Filho P.; da Silva L.A.B.; Faccioli L.H.; Paula-Silva F.W.G.	Effect of non-steroidal anti-inflammatory drugs on pulpal and periapical inflammation induced by lipopolysaccharide	2021	Clinical Oral Investigations
Wanachottrakul N.; Chotigeat W.; Kedjarune-Leggat U.	Effect of novel chitosan-fluoroaluminosilicate resin modified glass ionomer cement supplemented with translationally controlled tumor protein on pulp cells	2014	Journal of Materials Science: Materials in Medicine
Vianna E.C.B.; Herkrath F.J.; Martins I.E.B.; Lopes L.P.B.; Marques A.A.F.; Júnior E.C.S.	Effect of occlusal adjustment on postoperative pain after root canal treatment: A randomized clinical trial	2020	Brazilian Dental Journal
Parirokh M.; Rekabi A.R.; Ashouri R.; Nakhaee N.; Abbott P.V.; Gorjestani H.	Effect of occlusal reduction on postoperative pain in teeth with irreversible pulpitis and mild tenderness to percussion	2013	Journal of Endodontics

Yildirim M.D.; Cantekin K.	Effect of palonosetron on postoperative nausea and vomiting in children following dental rehabilitation under general anesthesia	2014	Pediatric dentistry
Günaydln A.; Çaklcl E.B.	Effect of Photobiomodulation Therapy following Direct Pulp Capping on Postoperative Sensitivity by Thermal Stimulus: A Retrospective Study	2021	Medical Principles and Practice
Tennert C.; Feldmann K.; Haamann E.; Al-Ahmad A.; Follo M.; Wrbas K.-T.; Hellwig E.; Altenburger M.J.	Effect of photodynamic therapy (PDT) on Enterococcus faecalis biofilm in experimental primary and secondary endodontic infections	2014	BMC Oral Health
Strazzi Sahyon H.B.; Pereira da Silva P.; Silva de Oliveira M.; Angelo Cintra L.T.; Gomes-Filho J.E.; Henrique dos Santos P.; Sivieri-Araujo G.	Effect of photodynamic therapy on the mechanical properties and bond strength of glass-fiber posts to endodontically treated intraradicular dentin	2018	Journal of Prosthetic Dentistry
Shahi S.; Mokhtari H.; Rahimi S.; Yavari H.R.; Narimani S.; Abdolrahimi M.; Nezafati S.	Effect of premedication with ibuprofen and dexamethasone on success rate of inferior alveolar nerve block for teeth with asymptomatic irreversible pulpitis: A randomized clinical trial	2013	Journal of Endodontics
Suresh N.; Nagendrababu V.; Koteeswaran V.; Haritha J.S.; Swetha S.D.; Varghese A.; Natanasabapathy V.	Effect of preoperative oral administration of steroids in comparison to an anti-inflammatory drug on postoperative pain following single-visit root canal treatment – a double-blind, randomized clinical trial	2021	International Endodontic Journal
Ahangari Z.; Naseri M.; Jalili M.; Mansouri Y.; Mashhadiabbas F.; Torkaman A.	Effect of propolis on dentin regeneration and the potential role of dental pulp stem cell in guinea pigs	2012	Cell Journal
Wayne JS, Chande R, Porter HC, Janus C.	Effect of restoration volume on stresses in a mandibular molar: a finite element study	2014	J Prosthet Dent
Stähli A, Schatt ASJ, Stoffel M, Nietzsche S, Sculean A, Gruber R, Cvikl B, Eick S.	Effect of scaling on the invasion of oral microorganisms into dentinal tubules including the response of pulpal cells-an in vitro study	2021	Clin Oral Investig
Kolhe P.S., Kolhe S.J., Khiyani T.S., Solanki H., Desai U.H., Chavda S.	Effect of silver diamine fluoride as indirect pulp capping agent in permanent dentition	2022	NeuroQuantology
Ahsana A.; Gurunathan D.	Effect of silver diamine fluoride treatment on the quality of life of children	2020	Indian Journal of Forensic Medicine and Toxicology
Saatchi M, Farhad AR, Shenasa N, Haghighi SK.	Effect of Sodium Bicarbonate Buccal Infiltration on the Success of Inferior Alveolar Nerve Block in Mandibular First Molars with Symptomatic Irreversible Pulpitis: A Prospective, Randomized Double-blind Study	2016	J Endod
Rayyan A.; Ather A.; Hargreaves K.M.; Ruparel N.B.	Effect of Sodium Hypochlorite in Dental Unit Waterline on Aerosolized Bacteria Generated from Endodontic Procedures	2022	Journal of Endodontics
Queiroz A.F.; Hidalgo M.M.; Consolaro A.; Panzarini S.; França A.B.; Melo M.E.; Poi W.	Effect of systemic antibiotic therapy on pulp repair following extrusive luxation and avulsion in a murine model: A histomorphological study	2022	Dental Traumatology
Kim E.-C.; Park H.; Lee S.-I.; Kim S.-Y.	Effect of the Acidic Dental Resin Monomer 10-methacryloyloxydecyl Dihydrogen Phosphate on Odontoblastic Differentiation of Human Dental Pulp Cells	2015	Basic and Clinical Pharmacology and Toxicology
Kabil NS, Badran AS, Wassel MO.	Effect of the addition of chlorhexidine and miswak extract on the clinical performance and antibacterial properties of conventional glass ionomer: an in vivo study	2017	Int J Paediatr Dent
Chunhacheevachaloke E, Tanapitchpong R, Ajcharanukul O.	Effect of the pulpal hydrostatic pressure on the morphological data of the fluid droplets emerging from dental enamel in human teeth	2020	Data Brief
Gupta S.; Tewari S.; Tewari S.; Mittal S.	Effect of time lapse between endodontic and periodontal therapies on the healing of concurrent endodontic-periodontal lesions without communication: A prospective randomized clinical trial	2015	Journal of Endodontics
Okamoto M, Takahashi Y, Komichi S, Ali M, Watanabe M, Hayashi M.	Effect of tissue inhibitor of metalloprotease 1 on human pulp cells in vitro and rat pulp tissue in vivo	2019	Int Endod J
Chang K.-C.; Chang C.-C.; Huang Y.-C.; Chen M.-H.; Lin F.-H.; Lin C.-P.	Effect of tricalcium aluminate on the physicochemical properties, bioactivity, and biocompatibility of partially stabilized cements	2014	PLoS ONE
Prasanti E.D.; Margono A.; Djauharie N.	Effect of triple antibiotic paste, calcium hydroxide, ledermix® on viability of pulp mesenchymal stem cells	2019	International Journal of Applied Pharmaceutics
Patil A., Sangle P., Sumanthini M.V., Satpute T., Naik D., Ghosh A.	Effect of Various Concentrations of Sodium Hypochlorite on the Microhardness of Bulk-fill Restorative Composite Resin used as a Pre-endodontic Restoration	2022	Journal of Clinical and Diagnostic Research

Danesh F.; Vahid A.; Jahanbani J.; Mashhadiabbas F.; Arman E.	Effect of white mineral trioxide aggregate compared with biomimetic carbonated apatite on dentine bridge formation and inflammatory response in a dental pulp model	2012	International Endodontic Journal
Kara Tuncer A.; Gerek M.	Effect of working length measurement by electronic apex locator or digital radiography on postoperative pain: A randomized clinical trial	2014	Journal of Endodontics
Jing Q, Wan K, Wang XJ, Ma L.	Effectiveness and safety of computer-controlled periodontal ligament injection system in endodontic access to the mandibular posterior teeth	2014	Chin Med Sci J
Batig V.M.; Borysenko A.V.; Prodanchuk A.I.; Hlushchenko T.A.; Kilmukhametova Y.H.; Batih I.V.; Tokar O.M.	Effectiveness of endodontic treatment in patients with generalized periodontitis with prevention of parasympathetic nervous system reactions	2020	Romanian Journal of Stomatology
Marques-da-Silva B., Alberton C.S., Tomazinho F.S.F., Gabardo M.C.L., Duarte M.A.H., Vivan R.R., Baratto-Filho F.	Effectiveness of five instruments when removing calcium hydroxide paste from simulated internal root resorption cavities in extracted maxillary central incisors	2020	International endodontic journal
Jayaraman J., Nagendrababu V., Pulikkotil S.J., Veettil S.K., Dhar V.	Effectiveness of formocresol and ferric sulfate as pulpotomy material in primary molars: A systematic review and meta-analysis with trial sequential analysis of randomized clinical trials	2020	Quintessence International
Tanwir A.; Ahmed S.; Akhtar H.; Wahid U.; Abbasi M.S.; Ahmed N.	Effectiveness of Single Dose Premedication of Piroxicam and Prednisolone on Post Endodontic Pain in One Visit Root Canal Treatment: A Randomized Clinical Trial	2022	European Endodontic Journal
BaniHani A, Deery C, Toumba J, Duggal M.	Effectiveness, Costs and Patient Acceptance of a Conventional and a Biological Treatment Approach for Carious Primary Teeth in Children	2019	Caries Res
Li W.; Yang H.; Gong Y.; Wang S.; Li Y.; Wei H.	Effects of a chimeric lysin against planktonic and sessile enterococcus faecalis hint at potential application in endodontic therapy	2018	Viruses
Bahrololoomi Z.; Amrollahi N.	Effects of acetaminophen and ibuprofen on pulpal anaesthesia immediately after pulpectomy of primary maxillary molars	2019	Iranian Endodontic Journal
Aslantas E.E.; Buzoglu H.D.; Muftuoglu S.F.; Atilla P.; Karapinar S.P.; Aksoy Y.	Effects of aging and inflammation on catalase activity in human dental pulp	2022	Archives of Oral Biology
Dahake P.T.; Kale Y.J.; Dadpe M.V.; Kendre S.B.	Effects of Biomimetic Materials on Stem Cells from Human Exfoliated Deciduous Teeth	2022	Regenerative Engineering and Translational Medicine
Ajcharanukul O, Matthews B.	Effects of bleaching on laser Doppler blood-flow signals recorded from human teeth in vitro	2015	Arch Oral Biol
Xu H.; Zhao J.; Chen G.; Yuan Z.; Liu J.	Effects of BMAL1 on dentinogenic differentiation of dental pulp stem cells via PI3K/Akt/mTOR pathway	2022	International Endodontic Journal
Michot B.; Casey S.M.; Gibbs J.L.	Effects of Calcitonin Gene-related Peptide on Dental Pulp Stem Cell Viability, Proliferation, and Differentiation	2020	Journal of Endodontics
Chang M.-C.; Lin L.-D.; Wu M.-T.; Chan C.-P.; Chang H.-H.; Lee M.-S.; Sun T.-Y.; Jeng P.-Y.; Yeung S.-Y.; Lin H.-J.; Jeng J.-H.	Effects of camphorquinone on cytotoxicity, cell cycle regulation and prostaglandin E2 production of dental pulp cells: Role of ROS, ATM/Chk2, MEK/ERK and hemeoxygenase-1	2015	PLoS ONE
Basurrah M., Kim D.H., Lee I.H., Kim S.W., Kim S.W.	Effects of Dental Factors on Fungal Sinusitis	2022	ORL
Jiang H., Shen L., Qin D., He S., Wang J.	Effects of dental general anaesthesia treatment on early childhood caries: A prospective cohort study in China	2019	BMJ Open
Cui Q, Wei M, Xiong Z, Hu S, Jiang J, Wang L, Cheng T, Wu X, Jiang H.	Effects of Dentin Ablation by a Q-Switching Er:YSGG Laser with a High Pulse Repetition Rate	2021	Photobiomodul Photomed Laser Surg
Hassan T.; Qiu Y.; Hasan M.R.; Saito T.	Effects of Dentin Phosphophoryn-Derived RGD Peptides on the Differentiation and Mineralization of Human Dental Pulp Stem Cells In Vitro	2022	Biomedicines
Gunes B., Yesildal Yeter K.	Effects of Different Glide Path Files on Apical Debris Extrusion in Curved Root Canals	2018	Journal of endodontics
Yazdanfar I, Gutknecht N, Franzen R.	Effects of diode laser on direct pulp capping treatment : a pilot study	2015	Lasers Med Sci
Sokouti Emamzadeh Hashemi I.; Maleki D.; Ebrahim Seyyed Monir S.; Ebrahimi A.; Tabari R.; Mousavi E.	Effects of Diode Low-Level Laser Therapy of 810 Nm on Pulpal Anesthesia of Maxillary Premolars: A Double-Blind Randomized Clinical Trial	2021	European Endodontic Journal

Niharika P.; Venugopal Reddy N.; Srujana P.; Srikanth K.; Daneswari V.; Sai Geetha K.	Effects of distraction using virtual reality technology on pain perception and anxiety levels in children during pulp therapy of primary molars	2018	Journal of Indian Society of Pedodontics and Preventive Dentistry
Banthitkhunanon P, Chintakanan S, Wanachantararak S, Vongsavan N, Matthews B.	Effects of enamel and dentine thickness on laser Doppler blood-flow signals recorded from the underlying pulp cavity in human teeth in vitro	2013	Arch Oral Biol
Kwon Y.-S.; Kim H.-J.; Hwang Y.-C.; Rosa V.; Yu M.-K.; Min K.-S.	Effects of Epigallocatechin Gallate, an Antibacterial Cross-linking Agent, on Proliferation and Differentiation of Human Dental Pulp Cells Cultured in Collagen Scaffolds	2017	Journal of Endodontics
Yamakawa S.; Niwa T.; Karakida T.; Kobayashi K.; Yamamoto R.; Chiba R.; Yamakoshi Y.; Hosoya N.	Effects of Er:YAG and diode laser irradiation on dental pulp cells and tissues	2018	International Journal of Molecular Sciences
Yaman E., Görken F., Erdem A.P., Sepet E., Aytepe Z.	Effects of folk medicinal plant extract ankaferd blood stopper® in vital primary molar pulpotomy	2012	European Archives of Paediatric Dentistry
Coll J.A., Campbell A., Chalmers N.I.	Effects of glass ionomer temporary restorations on pulpal diagnosis and treatment outcomes in primary molars	2013	Pediatric Dentistry
Luo Z.; Wang Z.; He X.; Liu N.; Liu B.; Sun L.; Wang J.; Ma F.; Duncan H.; He W.; Cooper P.	Effects of histone deacetylase inhibitors on regenerative cell responses in human dental pulp cells	2018	International Endodontic Journal
Zhang J.; Liu X.; Yu W.; Zhang Y.; Shi C.; Ni S.; Liu Q.; Li X.; Sun Y.; Zheng C.; Sun H.	Effects of human vascular endothelial growth factor on reparative dentin formation	2016	Molecular Medicine Reports
Bernardi LG, Favoreto MW, de Souza Carneiro T, Borges CPF, Pulido C, Loguercio AD.	Effects of microabrasion association to at-home bleaching on hydrogen peroxide penetration and color change	2022	J Esthet Restor Dent
Shi BQ, Yuan XJ, Zhao YM.	Effects of mineral trioxide aggregate and ethanolic extracts of Shandong propolis on the biological properties of human dental pulp fibroblasts	2019	Beijing Da Xue Xue Bao Yi Xue Ban
Ameghani B.A.; Tavakoli A.; Tabatabaei M.H.; Valizadeh S.	Effects of mineral trioxide aggregate and platelet-rich fibrin on histological results of direct pulp capping in dogs	2021	Giornale Italiano di Endodonzia
Araújo L.B.; Cosme-Silva L.; Fernandes A.P.; de Oliveira T.M.; Cavalcanti B.N.; Gomes Filho J.E.; Sakai V.T.	Effects of mineral trioxide aggregate, Biodentine™ and calcium hydroxide on viability, proliferation, migration and differentiation of stem cells from human exfoliated deciduous teeth	2018	Journal of Applied Oral Science
Youssef A.-R.; Emara R.; Taher M.M.; Al-Allaf F.A.; Almalki M.; Almasri M.A.; Siddiqui S.S.	Effects of mineral trioxide aggregate, calcium hydroxide, biodentine and Emdogain on osteogenesis, Odontogenesis, angiogenesis and cell viability of dental pulp stem cells	2019	BMC Oral Health
Shi B.; Zhao Y.; Yuan X.	Effects of MTA and Brazilian propolis on the biological properties of dental pulp cells	2019	Brazilian Oral Research
Nivetha R.; Murthykumar K.; Ashwin K.S.; Kumar N.; Priyadharshini R.	Effects of natural products on oral health: A review	2014	Asian Journal of Pharmaceutical and Clinical Research
Garrido P.R.; Pedroni A.C.F.; Cury D.P.; Moreira M.S.; Rosin F.; Sarra G.; Marques M.M.	Effects of photobiomodulation therapy on the extracellular matrix of human dental pulp cell sheets	2019	Journal of Photochemistry and Photobiology B: Biology
Ohkura N.; Edanami N.; Takeuchi R.; Tohma A.; Ohkura M.; Yoshiba N.; Yoshiba K.; Ida-Yonemochi H.; Ohshima H.; Okiji T.; Noiri Y.	Effects of pulpotomy using mineral trioxide aggregate on prostaglandin transporter and receptors in rat molars	2017	Scientific Reports
Liao C.; Wang Y.; Ou Y.; Wu Y.; Zhou Y.; Liang S.	Effects of sclerostin on lipopolysaccharide-induced inflammatory phenotype in human odontoblasts and dental pulp cells	2019	International Journal of Biochemistry and Cell Biology
Moon C.-Y.; Nam O.H.; Kim M.; Lee H.-S.; Kaushik S.N.; Walma D.A.C.; Jun H.-W.; Cheon K.; Choi S.C.	Effects of the nitric oxide releasing biomimetic nanomatrix gel on pulp-dentin regeneration: Pilot study	2018	PLoS ONE
Vanja P.; Nataša P.; Mia R.; Leković V.; Una V.; Stojić Ž.	Effects of the platelet rich plasma on apexogenesis in young monkeys: Radiological and hystologycal evaluation	2012	Acta Veterinaria
Lee M.-S.; Chen Y.-L.; Huang P.-H.; Chiang Y.-C.; Chang H.-H.; Wu J.; Lin C.-P.	Effects of ultrasonic and high-speed air-driven devices on pulp-dentin reactions: An animal study	2014	Journal of Dental Sciences
Su N.; Wang H.; Zhang S.; Liao S.; Yang S.; Huang Y.	Efficacy and safety of bupivacaine versus lidocaine in dental treatments: A meta-analysis of randomised controlled trials	2014	International Dental Journal
Arruda-Vasconcelos R.; Barbosa-Ribeiro M.; Louzada L.M.; Lemos B.I.N.; de-Jesus-Soares A.; Ferraz	Efficacy of 6% Sodium Hypochlorite on Infectious Content of Teeth with Symptomatic Irreversible Pulpitis	2022	Journal of Endodontics

C.C.R.; Almeida J.F.A.; Marciano M.A.; Gomes B.P.F.A.			
Vahedi Z.; Moshari A.; Moshari M.	Efficacy of adding dexmedetomidine to lidocaine to enhance inferior alveolar nerve block in patients with asymptomatic irreversible pulpitis: double-blind randomized clinical trial	2022	Clinical Oral Investigations
Gonna S., Ghoname N., Kabbash A., Yagi A.	Efficacy of aloe vera as a pulpotomy agent in children primary teeth: Clinical and radiographic studies	2019	Journal of Gastroenterology and Hepatology Research
Singhal N, Vats A, Khetarpal A, Ahlawat M, Vijayran VKR, Harshita.	Efficacy of articaine versus mepivacaine administered as different supplementary local anesthetic techniques after a failed inferior alveolar nerve block with lidocaine in patients with irreversible pulpitis: An in vivo study	2022	J Conserv Dent
Jasani B, Musale P, Jasani B.	Efficacy of Biodentine versus formocresol in pulpotomy of primary teeth: a systematic review and meta-analysis	2022	Quintessence Int
Alqahtani A.R.; Yaman P.; McDonald N.; Dennison J.	Efficacy of calcium hydroxide and resin-modified calcium silicate as pulp-capping materials: a retrospective study	2020	General dentistry
Peskersoy C.; Lukarcanin J.; Turkun M.	Efficacy of different calcium silicate materials as pulp-capping agents: Randomized clinical trial	2021	Journal of Dental Sciences
Cushley S, Duncan HF, Lappin MJ, Chua P, Elamin AD, Clarke M, El-Karim IA.	Efficacy of direct pulp capping for management of cariously exposed pulps in permanent teeth: a systematic review and meta-analysis	2021	Int Endod J
Naseri N.; Ghasemi N.; Baherimoghadam T.; Azmi A.	Efficacy of Er,Cr:YSGG laser for debonding of ceramic brackets and prevention of enamel damage and intrapulpal temperature change	2020	Lasers in Dental Science
Saber S.M.; Hashem A.A.; Khalil D.M.; Pirani C.; Ordinola-Zapata R.	Efficacy of four local anaesthesia protocols for mandibular first molars with symptomatic irreversible pulpitis: A randomized clinical trial	2022	International Endodontic Journal
Venkataraman K., Boominathan S., Nagappan R., Abraham C., Kaliyaperumal A., Nachimuthu J., Premkumar M.	Efficacy of glycolic acid on debris and smear removal as a final rinse solution in curved canals: A scanning electron microscope study	2021	Journal of Pharmacy and Bioallied Sciences
Saeed M.A.; El-Rahman M.A.; Helal M.E.; Zaher A.R.; Grawish M.E.	Efficacy of human platelet rich fibrin exudate vs fetal bovine serum on proliferation and differentiation of dental pulp stem cells	2017	International Journal of Stem Cells
Ghoddusi J.; Zarrabi M.H.; Daneshvar F.; Naghavi N.	Efficacy of IANB and gow-gates techniques in mandibular molars with symptomatic irreversible pulpitis: A prospective randomized double blind clinical study	2018	Iranian Endodontic Journal
Liang Y.; Ma R.; Chen L.; Dai X.; Zuo S.; Jiang W.; Hu N.; Deng Z.; Zhao W.	Efficacy of i-PRF in regenerative endodontics therapy for mature permanent teeth with pulp necrosis: study protocol for a multicentre randomised controlled trial	2021	Trials
Akhlaghi N.M.; Hormozi B.; Abbott P.V.; Khalilak Z.	Efficacy of Ketorolac Buccal Infiltrations and Inferior Alveolar Nerve Blocks in Patients with Irreversible Pulpitis: A Prospective, Double-blind, Randomized Clinical Trial	2016	Journal of Endodontics
Yavagal C.; Lal A.; Chavan Patil V.; Yavagal P.; Neelakantappa K.; Hariharan M.	Efficacy of laser photobiomodulation pulpotomy in human primary teeth: A randomized controlled trial	2021	Journal of Indian Society of Pedodontics and Preventive Dentistry
Samiei M.; Yavari H.; Shahi S.; Skandarinezhad M.; Abdollahi A.A.; Abolhasani S.	Efficacy of mental-incisive nerve block in root canal treatment of mandibular first molars with asymptomatic irreversible pulpitis: a randomized controlled trial	2020	General dentistry
Rodríguez-Wong L.; Pozos-Guillen A.; Silva-Herzog D.; Chavarría-Bolaños D.	Efficacy of mepivacaine-tramadol combination on the success of inferior alveolar nerve blocks in patients with symptomatic irreversible pulpitis: A randomized clinical trial	2016	International Endodontic Journal
Kérourédan O.; Jallon L.; Perez P.; Germain C.; Péli J.-F.; Oriez D.; Fricain J.-C.; Arrivé E.; Devillard R.	Efficacy of orally administered prednisolone versus partial endodontic treatment on pain reduction in emergency care of acute irreversible pulpitis of mandibular molars: Study protocol for a randomized controlled trial	2017	Trials
Shobana S.; Kavitha M.; Srinivasan N.	Efficacy of Platelet Rich Plasma and Platelet Rich Fibrin for Direct Pulp Capping in Adult Patients with Carious Pulp Exposure- A Randomised Controlled Trial	2022	European Endodontic Journal
Shafie L.; Esmaili S.; Parirokh M.; Pardakhti A.; Nakhaee N.; Abbott P.V.; Barghi H.	Efficacy of pre-medication with ibuprofen on post-operative pain after pulpotomy in primary molars	2018	Iranian Endodontic Journal
Mustafa M.; Almnea R.; Ajmal M.; Alamri H.M.; Abdulwahed A.; Divakar D.D.	Efficacy of root canal treatment in c-shaped canals with adjunctive photodynamic therapy using micro-CT	2021	Photodiagnosis and Photodynamic Therapy

Patil A.; Mali S.; Hegde D.; Jaiswal H.; Saoji H.; Edake D.N.	Efficacy of rotary and hand instrument in removing gutta-percha and sealer from root canals of endodontically treated teeth	2018	Journal of Contemporary Dental Practice
Atasoy Ulusoy O.I.; Alaçam T.	Efficacy of single buccal infiltrations for maxillary first molars in patients with irreversible pulpitis: A randomized controlled clinical trial	2014	International Endodontic Journal
Parirokh M, Sadr S, Nakhaee N, Abbott PV, Askarifard S.	Efficacy of supplementary buccal infiltrations and intraligamentary injections to inferior alveolar nerve blocks in mandibular first molars with asymptomatic irreversible pulpitis: a randomized controlled trial	2014	Int Endod J
Keefe S.M.; Hoffman-Censits J.; Cohen R.B.; Mamtani R.; Heitjan D.; Eliasof S.; Nixon A.; Turnbull B.; Garmey E.G.; Gunnarsson O.; Waliki M.; Ciconte J.; Jayaraman L.; Senderowicz A.; Tellez A.B.; Hennessy M.; Piscitelli A.; Vaughn D.; Smith A.; Haas N.B.	Efficacy of the nanoparticle-drug conjugate CRLX101 in combination with bevacizumab in metastatic renal cell carcinoma: Results of an investigator-initiated phase I-IIa clinical trial	2016	Annals of Oncology
Rajasekharan S, Martens LC, Vandenbulcke J, Jacquet W, Bottenberg P, Cauwels RG.	Efficacy of three different pulpotomy agents in primary molars: a randomized control trial	2017	Int Endod J
Ismiyatin K.; Subiyanto A.; Suhartono M.; Sari P.T.; Widjaja O.V.; Sari R.P.	Efficacy of topical hydrogel Epigallocatechin-3-gallate against neutrophil cells in perforated dental pulp	2020	Dental Journal
Nagarathna C, Shakuntala BS, Jaiganesh I.	Efficiency and Reliability of Thermal and Electrical Tests to Evaluate Pulp Status in Primary Teeth with Assessment of Anxiety Levels in Children	2015	J Clin Pediatr Dent
Silva C.B.B.; Bronzato J.D.; Herrera D.R.; Montagner F.; Nunes E.L.; Gomes B.P.F.A.	Efficiency of a digital electrofulguration system in contaminated root canals in vitro	2021	Brazilian Dental Journal
Zhang B., Yang B.-B., Gao Z.-Y., Li L., An H.	Efficiency of diode laser-assisted methods in direct pulp capping of carious teeth	2020	Shanghai kou qiang yi xue = Shanghai journal of stomatology
Orimoto A.; Kyakumoto S.; Eitsuka T.; Nakagawa K.; Kiyono T.; Fukuda T.	Efficient immortalization of human dental pulp stem cells with expression of cell cycle regulators with the intact chromosomal condition	2020	PLoS ONE
Richards J.F.; McClanahan S.B.; Bowles W.R.	Electrical Pulp Testing: Sources of Error	2015	Northwest dentistry
Daghrery A.; Aytac Z.; Dubey N.; Mei L.; Schwendeman A.; Bottino M.C.	Electrospinning of dexamethasone/cyclodextrin inclusion complex polymer fibers for dental pulp therapy	2020	Colloids and Surfaces B: Biointerfaces
Ayoub A.A.; Mahmoud A.H.; Ribeiro J.S.; Daghrery A.; Xu J.; Fenno J.C.; Schwendeman A.; Sasaki H.; Dal-Fabbro R.; Bottino M.C.	Electrospun Azithromycin-Laden Gelatin Methacryloyl Fibers for Endodontic Infection Control	2022	International Journal of Molecular Sciences
Du Y.; Yu X.; Wang W.; Zhao X.; Zhang L.; Sun Y.; Wang X.; Yu Q.	Elevated expression of importin 8 in inflamed human dental pulps	2017	International Journal of Clinical and Experimental Pathology
Lloyd A.; Uhles J.P.; Clement D.J.; Garcia-Godoy F.	Elimination of intracanal tissue and debris through a novel laser-activated system assessed using high-resolution micro-computed tomography: A pilot study	2014	Journal of Endodontics
Greenfield B.A.	Enamel defect restoration of the left mandibular first molar tooth	2012	Journal of Veterinary Dentistry
Zhang B.; Xiao M.; Cheng X.; Bai Y.; Chen H.; Yu Q.; Qiu L.	Enamel Matrix Derivative Enhances the Odontoblastic Differentiation of Dental Pulp Stem Cells via Activating MAPK Signaling Pathways	2022	Stem Cells International
Santana Neto M.C.; Lopes Bandeira A.V.; Santos Sousa H.C.; De Moura M.S.; De Fátima Almeida De Deus Moura L.; De Lima M.D.M.	Enamel of Premolars whose Predecessors Presented Rupture of the Follicle Bone Crypt from Periapical Infections	2020	Journal of Clinical Pediatric Dentistry
Rathi C., Chandak M., Mishra S., Chandak M.	Endocrown a new modality of treatment: Management of three cases	2020	European Journal of Molecular and Clinical Medicine
Dash S., Jena D., Govind S., Jena S.P., Singh N.R.	Endocrowns: A comprehensive review	2020	Indian Journal of Forensic Medicine and Toxicology
Bahillo J.; Freire M.; Martin-Biedma B.; Castelo-Baz P.	Endodontic and aesthetic management of a conoid tooth with type IIIA dens invaginatus	2021	European Journal of Paediatric Dentistry

Reis Só M.V.; Bonatto P.; Barreto M.S.; Vivian R.R.; Weidlich P.; Da Rosa R.A.	Endodontic and periodontal treatment of external cervical root resorption: A case report	2015	Dental Press Endodontics
Bahcall J.	Endodontic diagnosis: There's an app for that!	2012	Dentistry Today
Sasser L.	Endodontic Disinfection for Orthograde Root Canal Treatment in Veterinary Dentistry	2020	J Vet Dent
Ţogoe M.-M.; Crăciunescu E.-L.; Topală F.-I.; Sinescu C.; Nica L.M.; Ioniţă C.; Duma V.-F.; Romînu M.; Podoleanu A.G.; Negruțiu M.-L.	Endodontic fillings evaluated using en face OCT, microCT and SEM	2021	Romanian Journal of Morphology and Embryology
Gbadebo S.O.; Sulaiman A.O.; Anifowose O.O.	Endodontic flare up: incidence and association of possible risk factors	2016	African journal of medicine and medical sciences
Kunwar D.; Manandhar A.; Gurung G.; Khadka J.; Nepal M.	Endodontic indications among patients visiting a tertiary care center: A descriptive cross-sectional study	2021	Journal of the Nepal Medical Association
Sebring D., Buhlin K., Norhammar A., Rydén L., Jonasson P., Lund H., Kvist T.	Endodontic inflammatory disease: A risk indicator for a first myocardial infarction	2022	International endodontic journal
Santos B.S.; Silva R.V.; Pereira R.P.; Nunes E.	Endodontic intervention for type II dens invaginatus: A case report	2016	Dental Press Endodontics
Mei X.-H.; Liu J.; Wang W.; Zhang Q.-X.; Hong T.; Bai S.-Z.; Cheng X.-G.; Tian Y.; Jiang W.-K.	Endodontic management of a fused left maxillary second molar and two paramolars using cone beam computed tomography: A case report	2022	World Journal of Clinical Cases
Almutairi W., Alduraibi M.	Endodontic Management of a Fused Mandibular Third Molar with Supernumerary Tooth Using Cone-Beam Computed Tomography: A Case Report	2022	American Journal of Case Reports
Yadav K, De Ataide IN, Fernandes M, Lambor R, Alreja D.	Endodontic Management of a Mandibular First Molar with Radix Entomolaris and Conservative Post-endodontic Restoration with CAD/CAM Onlay: A Novel Clinical Technique	2016	J Clin Diagn Res
Lin G.S.S.; Ghani N.R.N.A.; Mokhtar K.; Halim M.S.	Endodontic management of a mature mandibular first permanent molar that survived for 20 years after complete pulpotomy: A case report	2019	Archives of Orofacial Sciences
Turkyilmaz A, Bulut AC, Hancerliogullari D.	Endodontic management of a patient with dystrophic epidermolysis bullosa: A case report	2021	Aust Endod J
Ramezani M., Asgari S., Adel M.	Endodontic management of a rare case of the geminated maxillary second molar tooth using CBCT	2021	Clinical Case Reports
Parolia A.; Khosla M.; Kundabala M.	Endodontic management of hypo-, meso- and hypertaurodontism: Case reports	2012	Australian Endodontic Journal
Tomar D.; Dhingra A.; Tomer A.; Sharma S.; Sharma V.; Miglani A.	Endodontic management of mandibular third molar with three mesial roots using spiral computed tomography scan as a diagnostic aid: A case report	2013	Oral Surgery, Oral Medicine, Oral Pathology and Oral Radiology
Battula MS, Kaushik M, Mehra N, Singh A.	Endodontic management of maxillary first molar with unusual anatomy	2022	J Conserv Dent
Sundaresh K.J.; Srinivasan R.; Mallikarjuna R.; Rajalbandi S.	Endodontic management of middle mesial canal of the mandibular molar	2013	BMJ Case Reports
Alfahadi H.R.; Alghamdi A.M.	Endodontic management of permanent mandibular canine with Type II canal configuration using cone-beam computed tomography. A Case report	2020	Saudi Endodontic Journal
Howlader M.R.; Chowdhury S.S.; Rahman M.; Hossain A.; Zannat T.A.	Endodontic management of symptomatic pulpal pathosis by single -visit root canal therapy.	2014	Mymensingh medical journal : MMJ
Celikten B., Yalniz H., Huang Y.	Endodontic management of taurodontism with a complex root canal anatomy in mandibular posterior teeth	2017	BMJ Case Reports
Zhang ZH, Yao HL, Zhang Y, Wang X.	Endodontic management of the maxillary first molar with special root canals: A case report and review of the literature	2020	World J Clin Cases
Liu J.; Que K.-H.; Xiao Z.-H.; Wen W.	Endodontic management of the maxillary first molars with two root canals: A case report and review of the literature	2019	World Journal of Clinical Cases
Ramugade M.M., Patel A., Sagale A., Abrar S.	Endodontic management of unusual maxillary first molar with fusion using cone-beam computed tomography as a diagnostic aid	2019	Journal of Clinical and Diagnostic Research
Rodrigues M.X.; Nemec A.; Fiani N.; Bicalho R.C.; Peralta S.	Endodontic Microbiome of Fractured Non-vital Teeth in Dogs Determined by 16S rRNA Gene Sequencing	2019	Frontiers in Veterinary Science
Zubizarreta-Macho Á, Ferreiroa A, Agustín-Panadero R, Rico-Romano C, Lobo-Galindo AB, Mena-Álvarez J.	Endodontic re-treatment and restorative treatment of a dens invaginatus type II through new technologies	2019	J Clin Exp Dent

Shobhana R., Subbiya A., Geethapriya N., Anuradha B.	Endodontic retreatment of mandibular second molar with middle mesial canal: A case report	2019	Indian Journal of Public Health Research and Development
Pinheiro H.H.C.; Assunção L.R.S.; Torres D.K.B.; Miyahara L.A.N.; Arantes D.C.	Endodontic therapy in primary teeth by pediatric dentists; Terapia endodôntica em dentes decíduos por odontopediatras	2014	Pesquisa Brasileira em Odontopediatria e Clinica Integrada
Macgee S.	Endodontic therapy of a mandibular canine tooth with irreversible pulpitis secondary to dentigerous cyst	2014	Journal of Veterinary Dentistry
	Endodontic treatment	2017	Duodecim
Firmino R.T.; Brandt L.M.T.; Ribeiro G.L.; dos Santos K.S.A.; Catão M.H.C.D.V.; Gomes D.Q.D.C.	Endodontic treatment associated with photodynamic therapy: Case report	2016	Photodiagnosis and Photodynamic Therapy
Anuradha B., Sharumathi R., Venkatesh A., Niveditha S.	Endodontic treatment failures and its management—a review	2019	Indian Journal of Public Health Research and Development
Seewald M.; Gohl C.; Egerbacher M.; Handschuh S.; Witter K.	Endodontic Treatment of a Traumatic Tusk Fracture With Exposed Pulp in an Asian Elephant (Elephas maximus)	2021	Journal of Veterinary Dentistry
Buchanan G.D.; Tredoux S.; Nel C.; Gamieldien M.Y.	Endodontic treatment of dentin dysplasia type I D	2021	Australian Endodontic Journal
C do Nascimento A., A F Marques A., C Sponchiado-Júnior E., F R Garcia L., M A de Carvalho F.	Endodontic Treatment of Hypertaurodontic Mandibular Molar Using Reciprocating Single-file System: A Case Report	2016	The Bulletin of Tokyo Dental College
Chauhan R., Singh S.	Endodontic treatment of mandibular molars with atypical root canal anatomy: reports of 4 cases	2015	General dentistry
De Deus Moura L.D.F.A.; De Lima M.D.D.M.; Lima C.C.B.; Machado J.I.A.G.; De Moura M.S.; De Carvalho P.V.	Endodontic treatment of primary molars with antibiotic paste: A report of 38 cases	2016	Journal of Clinical Pediatric Dentistry
Di Giorgio G., Covello F., Salucci A., Bossù M.	Endodontic treatment of the deciduous tooth	2020	Dental Cadmos
Pires M.D., Martins J.N.R.	Endodontic treatment of the mandibular first molar with three distal root canals - Case series	2019	Revista Portuguesa de Estomatologia, Medicina Dentaria e Cirurgia Maxilofacial
Martins J.N.R.; Anderson C.	Endodontic treatment of the maxillary first molar with five root canals - Three case reports	2013	Revista Portuguesa de Estomatologia, Medicina Dentaria e Cirurgia Maxilofacial
Chen K, Ran X, Wang Y.	Endodontic treatment of the maxillary first molar with palatal canal variations: A case report and review of literature	2022	World J Clin Cases
Lu Q, Wang P, Yang B, Liu XJ, Wang SY, Yu Q.	Endodontic treatments of mandibular first molar with middle mesial canal: two case reports	2013	Chin J Dent Res
Lopes C.S.; de Azevedo Moreira S.; Nícoli G.A.; Ramirez I.; Viola N.V.	Endodontical treatment of periapical tooth injury with photodynamic therapy: Case report	2019	Photodiagnosis and Photodynamic Therapy
Prati C., Pelliccioni G.A., Gissi D., Spinelli A., Zamparini F., Gandolfi M.G.	Endodontics in presence of systemic diseases: operative techniques and their importance	2021	Dental Cadmos
Jivoinovici R.; Suciu I.; Dimitriu B.; Perlea P.; Bartok R.; Malita M.; Ionescu C.	Endo-periodontal lesion--endodontic approach	2014	Journal of medicine and life
Yoo C.H.; Na H.-J.; Lee D.-S.; Heo S.C.; An Y.; Cha J.; Choi C.; Kim J.H.; Park J.-C.; Cho Y.S.	Endothelial progenitor cells from human dental pulp-derived iPS cells as a therapeutic target for ischemic vascular diseases	2013	Biomaterials
Ribeiro J.S.; Münchow E.A.; Bordini E.A.F.; Rodrigues N.S.; Dubey N.; Sasaki H.; Fenno J.C.; Schwendeman S.; Bottino M.C.	Engineering of Injectable Antibiotic-Laden Fibrous Microparticles Gelatin Methacryloyl Hydrogel for Endodontic Infection Ablation	2022	International Journal of Molecular Sciences
Mehboob R.; Hassan S.; Gilani S.A.; Hassan A.; Tanvir I.; Waseem H.; Hanif A.	Enhanced Neurokinin-1 Receptor Expression Is Associated with Human Dental Pulp Inflammation and Pain Severity	2021	BioMed Research International
Alqarawi F.K.	Enhancing the esthetics of a maxillary central implant crown with a hybrid-abutment: A case report	2022	Saudi Journal of Medicine and Medical Sciences
Kovac J.; Kovac D.; Slobodnikova L.; Kotulova D.	Enterococcus faecalis and Candida albicans in the dental root canal and periapical infections	2013	Bratislava Medical Journal

Sabogal Á, Asencios J, Robles A, Gamboa E, Rosas J, Ríos J, Mayta-Tovalino F.	Epidemiological Profile of the Pathologies of the Oral Cavity in a Peruvian Population: A 9-Year Retrospective Study of 18,639 Patients	2019	ScientificWorldJournal
Liu Y.; Gan L.; Cui D.-X.; Yu S.-H.; Pan Y.; Zheng L.-W.; Wan M.	Epigenetic regulation of dental pulp stem cells and its potential in regenerative endodontics	2021	World Journal of Stem Cells
Smedley RC, Earley ET, Galloway SS, Baratt RM, Rawlinson JE.	Equine Odontoclastic Tooth Resorption and Hypercementosis: Histopathologic Features	2015	Vet Pathol
Yonehiro J.; Yamashita A.; Yoshida Y.; Yoshizawa S.; Ohta K.; Kamata N.; Okihara T.; Nishimura F.	Establishment of an ex vivo pulpitis model by co-culturing immortalized dental pulp cells and macrophages	2012	International Endodontic Journal
Ma Z.; Zhou M.; Wang L.; Cheng Q.; Hong J.	Establishment of Pulp Damage Repair Models in Miniature Pigs Using Diode Lasers	2021	Photobiomodulation, Photomedicine, and Laser Surgery
Barroso S.D.; Jales T.S.; Corrêa-Faria P.; Alcântara C.E.P.; Botelho A.M.; Tavano K.T.A.	Esthetic and functional recovery of extensively decayed posterior teeth through conservative treatment	2013	Journal of Clinical Pediatric Dentistry
Alkaabi A.M.; AlHumaidan A.A.; AlQarawi F.K.; AlShahrani F.A.	Esthetic smile rehabilitation of enamel hypomineralized teeth with E-max prosthesis: Case report	2019	Saudi Dental Journal
Mashyakhy M, Vinothkumar TS, Arthisri AS, Renugalakshmi A, Alamir A, Alkahtany M, Juraybi A.	Ethnical Anatomical Differences in Mandibular First Permanent Molars between Indian and Saudi Arabian Subpopulations: A Retrospective Cross-sectional Study	2021	J Contemp Dent Pract
Siqueira V.-D.S.; Mateo-Castillo J.-F.; Pinto L.-D.C.; Garib D.; Pinheiro C.-R.	Etiological factors commonly related to the need of endodontic treatment in individuals with orofacial clefts	2021	Journal of Clinical and Experimental Dentistry
Rasera Zotelli V.L.; Grillo C.M.; Rosário de Sousa M.D.L.	Etiology of acute dental pain in patients accepting acupuncture procedures; Etiología del dolor dental agudo en pacientes que aceptan procedimientos de acupuntura	2017	Revista Internacional de Acupuntura
Duncan H.F., Galler K.M., Tomson P.L., Simon S., El-Karim I., Kundzina R., Krastl G., Dammaschke T., Fransson H., Markvart M., Zehnder M., Bjørndal L.	European Society of Endodontology position statement: Management of deep caries and the exposed pulp	2019	International endodontic journal
Idzik S.; Krauss E.	Evaluating and managing dental complaints in primary and urgent care	2013	Journal for Nurse Practitioners
Rao A.C.; Venkatesh K.V.; Nandini V.; Sihivahanan D.; Alamoudi A.; Bahammam H.A.; Bahammam S.A.; Zidane B.; Bahammam M.A.; Chohan H.; Albar N.H.; Yadalam P.K.; Patil S.	Evaluating the Effect of Tideglusib-Loaded Bioactive Glass Nanoparticles as a Potential Dentine Regenerative Material	2022	Materials
Abella F.; Patel S.; Duran-Sindreu F.; Mercadé M.; Bueno R.; Roig M.	Evaluating the periapical status of teeth with irreversible pulpitis by using cone-beam computed tomography scanning and periapical radiographs	2012	Journal of Endodontics
Peng X.; Han S.; Wang K.; Ding L.; Liu Z.; Zhang L.	Evaluating the potential of an amelogenin-derived peptide in tertiary dentin formation	2021	Regenerative Biomaterials
Minavi B.; Youssefi A.; Quock R.; Letra A.; Silva R.; Kirkpatrick T.C.; Tribble G.; van der Hoeven R.	Evaluating the substantivity of silver diamine fluoride in a dentin model	2021	Clinical and Experimental Dental Research
Patel S., Patel P., Patel P., Agnani S., Kumar A., Mangalekar S.B.	Evaluation and comparison of apical bacterial extrusion after instrumentation of the root canals using various endodontic file systems: An in vitro study	2022	Journal of Pharmaceutical Negative Results
Vishanth S.; Sherwood I.A.; Gutmann J.L.; Murugadoss V.; Prince E.	Evaluation of 3 different treatment modalities for conservative management of attrited, sensitive molar teeth – A preliminary 12-week report	2020	Australian Endodontic Journal
Liu S.; Wang S.; Dong Y.	Evaluation of a Bioceramic as a Pulp Capping Agent in Vitro and in Vivo	2015	Journal of Endodontics
Waring E.; Mawardi H.; Woo S.B.; Treister N.; Margalit D.N.; Frustino J.; Villa A.	Evaluation of a community-based dental screening program prior to radiotherapy for head and neck cancer: a single-center experience	2019	Supportive Care in Cancer
Aboujaoude S.; Noueiri B.; Berbari R.; Khairalla A.; Sfeir E.	Evaluation of a modified pulpotec endodontic approach on necrotic primary molars: A one-year follow-up	2015	European Journal of Paediatric Dentistry
Du T.; Ma J.; Yang P.; Xiong Z.; Lu X.; Cao Y.	Evaluation of antibacterial effects by atmospheric pressure nonequilibrium plasmas against enterococcus faecalis biofilms in vitro	2012	Journal of Endodontics
Diniz I.M.; Teixeira K.I.; Araújo P.V.; Araújo M.S.; Marques M.M.; Poletto L.T.; Cortés M.E.	Evaluation of antibacterial photodynamic therapy effects on human dental pulp cell cultures	2014	Photodiagnosis and photodynamic therapy

Divya S., Sujatha S.	Evaluation of antimicrobial effect of triphala versus conventional root canal irrigants in primary teeth - An in vivo study	2019	Research Journal of Pharmacy and Technology
Singh A.; Kaur H.; Soni P.; Choudhary R.; Yeluri R.	Evaluation of biodentine pulpotomy in caries-exposed symptomatic vital mature permanent teeth in 9–13-year-old children: A 24-month clinico-radiographic observation	2022	Journal of Dental Research, Dental Clinics, Dental Prospects
Allabban M.N.M., Youssef S.A., Nejri A.A.M., Qudaih M.A.A.	Evaluation of bond strength of aesthetic type of posts at different regions of root canal after application of adhesive resin cement	2019	Open Access Macedonian Journal of Medical Sciences
Chopra R.; Marwaha M.; Bansal K.; Mittal M.	Evaluation of buccal infiltration with articaine and inferior alveolar nerve block with lignocaine for pulp therapy in mandibular primary molars	2016	Journal of Clinical Pediatric Dentistry
Aggarwal R., Aggarwal R., Aggarwal A.	Evaluation of Canal Transportation and Centering Ability of Three Single File Systems	2022	European Journal of Molecular and Clinical Medicine
Obadiah I., Subramanian E.M.G., Ravindran V.	Evaluation of carious primary teeth that causes swelling in children visiting a private dental institute-an observational study	2020	International Journal of Research in Pharmaceutical Sciences
Buonavoglia A.; Lauritano D.; Perrone D.; Ardito F.; Troiano G.; Dioguardi M.; Candotto V.; Silvestre F.J.; Muzio L.L.O.	Evaluation of chemical-physical properties & cytocompatibility of theracal LC	2017	Journal of Biological Regulators and Homeostatic Agents
Wu S.; Zhou Y.; Yu Y.; Zhou X.; Du W.; Wan M.; Fan Y.; Zhou X.; Xu X.; Zheng L.	Evaluation of Chitosan Hydrogel for Sustained Delivery of VEGF for Odontogenic Differentiation of Dental Pulp Stem Cells	2019	Stem Cells International
Kaushik S.N.; Scoffield J.; Andukuri A.; Alexander G.C.; Walker T.; Kim S.; Choi S.C.; Brott B.C.; Eleazer P.D.; Lee J.-Y.; Wu H.; Childers N.K.; Jun H.-W.; Park J.-H.; Cheon K.	Evaluation of ciprofloxacin and metronidazole encapsulated biomimetic nanomatrix gel on Enterococcus faecalis and Treponema denticola	2015	Biomaterials Research
Li Y.; Sun K.; Ye G.; Liang Y.; Pan H.; Wang G.; Zhao Y.; Pan J.; Zhang J.; Fang J.	Evaluation of Cold Plasma Treatment and Safety in Disinfecting 3-week Root Canal Enterococcus faecalis Biofilm in Vitro	2015	Journal of Endodontics
Scotti N.; Alovisi C.; Comba A.; Ventura G.; Pasqualini D.; Grignolo F.; Berutti E.	Evaluation of Composite Adaptation to Pulpal Chamber Floor Using Optical Coherence Tomography	2016	Journal of Endodontics
Paula A.B.; Laranjo M.; Marto C.-M.; Paulo S.; Abrantes A.M.; Fernandes B.; Casalta-Lopes J.; Marques-Ferreira M.; Botelho M.F.; Carrilho E.	Evaluation of dentinogenesis inducer biomaterials: An in vivo study	2020	Journal of Applied Oral Science
Mandinić Z., Prokić A., Mandić J., Blagojević D., Kanjevac T., Stojković B., Stevanović M., Jovičić O.	Evaluation of dentists’ knowledge about the use of radiology in pediatric dentistry in Serbia	2021	Vojnosanitetski Pregled
Balto H., Salama F., Al-Mofareh S., Al-Yahya F.	Evaluation of different irrigating solutions on smear layer removal of primary root dentin	2015	The journal of contemporary dental practice
Alim B.A., Garip Berker Y.	Evaluation of different root canal filling techniques in severely curved canals by micro-computed tomography	2020	Saudi Dental Journal
Sunil N.S.B.M.A., Duraisamy R., Nasim I.	Evaluation of direct/indirect pulp capping procedures requiring root canal therapy	2020	Indian Journal of Forensic Medicine and Toxicology
Ergesheva E.V., Davidiants A.A., Dorofeev A.E., Sevbitova M.A., Timoshina M.D.	Evaluation of effectiveness of an alternative method in complex treatment of diseases of endodont	2018	Asian Journal of Pharmaceutics
Raheja J.; Tewari S.; Tewari S.; Duhan J.	Evaluation of efficacy of chlorhexidine intracanal medicament on the periodontal healing of concomitant endodontic-periodontal lesions without communication: An interventional study	2014	Journal of Periodontology
Sharma V.; Nawal R.R.; Augustine J.; Urs A.B.; Talwar S.	Evaluation of Endosequence Root Repair Material and Endocem MTA as direct pulp capping agents: An in vivo study	2022	Australian Endodontic Journal
Varshney A.; Shetty A.; Hegde M.N.; Shetty S.; Rao L.N.	Evaluation of factors responsible for failure of root canal treatment in an adult indian population: A cross sectional study	2019	Indian Journal of Public Health Research and Development
Yildiz E.; Tosun G.	Evaluation of formocresol, calcium hydroxide, ferric sulfate, and MTA primary molar pulpotomies	2014	European Journal of Dentistry

Accorsi-Mendonça T.; Silva E.J.N.L.; Marcaccini A.M.; Gerlach R.F.; Duarte K.M.R.; Pardo A.P.S.; Line S.R.P.; Zaia A.A.	Evaluation of gelatinases, tissue inhibitor of matrix metalloproteinase-2, and myeloperoxidase protein in healthy and inflamed human dental pulp tissue	2013	Journal of Endodontics
Tai Z.-W., Peng W.-W., Du R., Zhu Y.-Q.	Evaluation of glide path preparation and shaping ability of two nickel-titanium engine driven systems in moderate curved root canals	2020	Journal of Shanghai Jiaotong University (Medical Science)
Kirilova J., Topalova-Pirinska S., Kirov D.	Evaluation of impact of lining application techniques on marginal microleakage in resin-modified glass ionomer cement in class II composite restorations: An in vitro study	2019	Journal of IMAB - Annual Proceeding (Scientific Papers)
Astudillo-Ortiz E.; Babo P.S.; Reis R.L.; Gomes M.E.	Evaluation of injectable hyaluronic acid-based hydrogels for endodontic tissue regeneration	2021	Materials
Aishuwariya T.; Ramesh S.	Evaluation of interleukin 6, interleukin 8, tnf alpha as biomarkers for pulpitis-in vivo study	2021	International Journal of Dentistry and Oral Science
Altan Şallı G, Egil E.	Evaluation of mesial root canal configuration of mandibular first molars using micro-computed tomography	2021	Imaging Sci Dent
Cavenago B.C., Del Carpio-Perochena A.E., Amoroso-Silva P.A., Alcalde M.P., Fernandes S.L., Vivan R.R., Duarte M.A.H.	Evaluation of Mesial Root Canals of Mandibular Molars Obturated with Gutta-Percha and Resilon Techniques	2017	European endodontic journal
Meslmani W.; Othman R.; Kouchaji C.; Salem Rekab M.; Abo Fakher M.A.	Evaluation of modified portland cement versus mineral trioxide aggregate as a pulpotomy agent in primary molars: Histological study	2020	International Journal of Dentistry and Oral Science
Raja S.A.J.; Raja J.J.; Vijayashree R.; Priya B.M.; Anusuya G.S.; Ravishankar P.	Evaluation of oral and periodontal status of leprosy patients in Dindigul district	2016	Journal of Pharmacy and Bioallied Sciences
Wadhwa J., Gupta A., Hans S.	Evaluation of periapical healing of apicomarginal defect in mandibular first molar treated with platelet rich fibrin: A case report	2017	Journal of Clinical and Diagnostic Research
Daković D., Lekić M., Bokonjić D., Lazić Z., Čutović T., Mladenović R.	Evaluation of periodontal status and treatment needs of the Serbian Armed Forces population	2021	Vojnosanitetski Pregled
Atesci A.A.; Topaloglu-Ak A.; Turan E.; Oncag O.; Kaval M.E.	Evaluation of Postoperative Pain Following Single-Visit Root Canal Treatment with Rotary and Reciprocal Ni–Ti File Systems in Children	2022	Medicina (Lithuania)
Tunc F.; Yildirim C.; Alacam T.	Evaluation of postoperative pain/discomfort after intracanal use of Nd:YAG and diode lasers in patients with symptomatic irreversible pulpitis and asymptomatic necrotic pulps: a randomized control trial	2021	Clinical Oral Investigations
Sankar P., Ravindran V.	Evaluation of primary central incisors requiring restoration and pulpectomy in 2 to 6 year old children-an observational study	2020	Indian Journal of Forensic Medicine and Toxicology
Deepak A., Subramaniam E.M.G., Jeevanandan G., Jeevitha M.	Evaluation of primary teeth with caries requiring pulpectomy and extraction in children between 5-7 years old visiting a university dental hospital	2020	European Journal of Molecular and Clinical Medicine
Lo Giudice A, Leonardi R, Ronsivalle V, Allegrini S, Lagravère M, Marzo G, Isola G.	Evaluation of pulp cavity/chamber changes after tooth-borne and bone-borne rapid maxillary expansions: a CBCT study using surface-based superimposition and deviation analysis	2021	Clin Oral Investig
Minic S, Florimond M, Sadoine J, Valot-Salengro A, Chaussain C, Renard E, Boukpessi T.	Evaluation of Pulp Repair after Biodentine(TM) Full Pulpotomy in a Rat Molar Model of Pulpitis	2021	Biomedicines
Parvin MK, Moral AA, Shikder ZH, Alam MS, Bashar AM.	Evaluation of Radiological Outcomes of Theracal Light Cured (TLC) And Calcium Hydroxide As Indirect Pulp Capping Agents In The Treatment Of Deep Carious Lesion Of Permanent Molar Teeth	2018	Mymensingh Med J
Al-Hezaimi K.; Naghshbandi J.; Alhuzaimi R.; Alonaizan F.; AlQwizany I.; Rotstein I.	Evaluation of recombinant human platelet-derived growth factor or enamel matrix derivative plus calcium hydroxide for pulp capping: A randomized controlled human clinical trial	2020	International Journal of Periodontics and Restorative Dentistry
Vaishali S., Jeevanandan G., Duraisamy R.	Evaluation of risk of periodontitis in children-a retrospective study	2020	International Journal of Research in Pharmaceutical Sciences
Guo J.; Vahidnia A.; Sedghizadeh P.; Enciso R.	Evaluation of Root and Canal Morphology of Maxillary Permanent First Molars in a North American Population by Cone-beam Computed Tomography	2014	Journal of Endodontics

Karthikeson P.S.; Gayathri R.; Vishnu Priya V.	Evaluation of salivary total proteins, albumin, globulin, and A/G ratio among healthy individuals and patients with chronic pulpitis	2018	Drug Invention Today
Timme M, Borkert J, Nagelmann N, Schmeling A.	Evaluation of secondary dentin formation for forensic age assessment by means of semi-automatic segmented ultrahigh field 9.4 T UTE MRI datasets	2020	Int J Legal Med
Sooratgar A.; Ahmadi Z.; Asadi Y.; Dibaji F.; Shamshiri A.R.; Afkhami F.	Evaluation of Secondary Thermal Hyperalgesia Resulting from Pulpal Inflammation in Patients with Symptomatic Irreversible Pulpitis	2021	Journal of Endodontics
Patel M.; Makwani D.; Bhatt R.; Raj V.; Patel C.; Patel F.	Evaluation of silver-modified atraumatic restorative technique versus conventional pulp therapy in asymptomatic deep carious lesion of primary molars - A comparative prospective clinical study	2022	Journal of Indian Society of Pedodontics and Preventive Dentistry
Mandakhbayar N.; El-Fiqi A.; Lee J.-H.; Kim H.-W.	Evaluation of Strontium-Doped Nanobioactive Glass Cement for Dentin-Pulp Complex Regeneration Therapy	2019	ACS Biomaterials Science and Engineering
Mbizah MM, Steenkamp G, Groom RJ.	Evaluation of the Applicability of Different Age Determination Methods for Estimating Age of the Endangered African Wild Dog (Lycaon Pictus)	2016	PLoS One
Kalaskar R, Baliga S, Balasubramanian S.	Evaluation of the average pulp cavity volume of primary second molar and canine for obturation of a single primary tooth: A preliminary cone-beam computed tomographic study	2021	J Indian Soc Pedod Prev Dent
Cheng X.; Guan S.; Lu H.; Zhao C.; Chen X.; Li N.; Bai Q.; Tian Y.; Yu Q.	Evaluation of the bactericidal effect of Nd:YAG, Er:YAG, Er,Cr:YSGG laser radiation, and antimicrobial photodynamic therapy (aPDT) in experimentally infected root canals	2012	Lasers in Surgery and Medicine
Erben P.; Chang A.M.; Darveau R.P.; Fong H.; Johnson J.D.; Paranjpe A.	Evaluation of the bactericidal potential of 2780-nm ER,CR:YSGG and 940-nm diode lasers in the root canal system	2019	Lasers in Dental Science
Akbiyik S.Y.; Bakir E.P.; Bakir S.	Evaluation of the Bond Strength of Different Pulp Capping Materials to Dental Adhesive Systems: An In Vitro Study	2021	Journal of Advanced Oral Research
Bakir Ş.; Bakir E.P.; Akbiyik S.Y.	Evaluation of the bond strength of resin-modified glass ionomer enhanced with bioactive glass to composite resin with different dental adhesive systems	2021	Analytical and Quantitative Cytopathology and Histopathology
Böttcher D.E.; Sehnem N.T.; Montagner F.; Fatturi Parolo C.C.; Grecca F.S.	Evaluation of the Effect of Enterococcus faecalis Biofilm on the 2% Chlorhexidine Substantivity: An in Vitro Study	2015	Journal of Endodontics
Khajehzadeh F., Raoufinejad F., Gorjestani H., Kamali M., Nassaj A.E., Shahravan A.	Evaluation of the effect of furcation perforation on the fracture resistance of endodontically treated mandibular molars	2021	Journal of Kerman University of Medical Sciences
da Mota A.C.C.; Gonçalves M.L.L.; Bortoletto C.; Olivan S.R.; Salgueiro M.; Godoy C.; Altavista O.M.; Pinto M.M.; Horliana A.C.; Motta L.J.; Bussadori S.K.	Evaluation of the effectiveness of photodynamic therapy for the endodontic treatment of primary teeth: Study protocol for a randomized controlled clinical trial	2015	Trials
de Alencar Filho A.V.; dos Santos Junior V.E.; da Silva Calixto M.; Santos N.; Heimer M.V.; Rosenblatt A.	Evaluation of the genotoxic effects of formocresol application in vital pulp therapy of primary teeth: a clinical study and meta-analysis	2018	Clinical Oral Investigations
Click V.; Drum M.; Reader A.; Nusstein J.; Beck M.	Evaluation of the gow-gates and vazirani-Akinosi techniques in patients with symptomatic irreversible pulpitis: A prospective randomized study	2015	Journal of Endodontics
Dongmei G., Fang S., Qi X.	Evaluation of the number of roots and root canal morphology and distribution in chinese children's mandibular first deciduous teeth by cone-beam computed tomography	2019	Chinese Journal of Tissue Engineering Research
Mousivand S, Sheikhnezami M, Moradi S, Koohestanian N, Jafarzadeh H.	Evaluation of the outcome of apexogenesis in traumatised anterior and carious posterior teeth using mineral trioxide aggregate: a 5-year retrospective study	2022	Aust Endod J
Nováková D, Svec P, Kukletová M, Záčková L, Sedláček I.	Evaluation of the strain identity between isolates from caries lesions and root canals in early childhood caries cases	2013	Folia Microbiol (Praha)
Sherwood I.; Divyameena B.; Ramyadharshini T.; Subashri V.; Banerjee A.	Evaluation of two conservative different treatment protocols for symptomatic proximal deep caries management in molar teeth; An 18-month clinical report	2021	Endodontology
Nagaraja S.; Mathew S.; Abraham A.; Ramesh P.; Chandanala S.	Evaluation of vascular endothelial growth factor - A release from platelet-rich fibrin, platelet-rich fibrin matrix, and dental pulp at different time intervals	2020	Journal of Conservative Dentistry

Porto LV, Celestino da Silva Neto J, Anjos Pontual AD, Catunda RQ.	Evaluation of volumetric changes of teeth in a Brazilian population by using cone beam computed tomography	2015	J Forensic Leg Med
Bowler K.E.; Worsley M.A.; Broad L.; Sher E.; Benschop R.; Johnson K.; Yates J.M.; Robinson P.P.; Boissonade F.M.	Evidence for anti-inflammatory and putative analgesic effects of a monoclonal antibody to calcitonin gene-related peptide	2013	Neuroscience
Mahdee A, Eastham J, Whitworth JM, Gillespie JI.	Evidence for changing nerve growth factor signalling mechanisms during development, maturation and ageing in the rat molar pulp	2019	Int Endod J
Lockhart P.B.; Tampi M.P.; Abt E.; Aminoshariae A.; Durkin M.J.; Fouad A.F.; Gopal P.; Hatten B.W.; Kennedy E.; Lang M.S.; Patton L.L.; Paumier T.; Suda K.J.; Pilcher L.; Urquhart O.; O'Brien K.K.; Carrasco-Labra A.	Evidence-based clinical practice guideline on antibiotic use for the urgent management of pulpal- and periapical-related dental pain and intraoral swelling: A report from the American Dental Association	2019	Journal of the American Dental Association
Doméjean S, Grosgogeat B.	Evidence-Based Deep Carious Lesion Management: From Concept to Application in Everyday Clinical Practice	2018	Monogr Oral Sci
Giacaman R.A., Muñoz-Sandoval C., Neuhaus K.W., Fontana M., Chałas R.	Evidence-based strategies for the minimally invasive treatment of carious lesions: Review of the literature	2018	Advances in Clinical and Experimental Medicine
Kurtzman G.M.	Evolution of Comprehensive Care, Part 4. Direct Restorations	2015	Dentistry today
Averianov AO, Archibald JD.	Evolutionary transition of dental formula in Late Cretaceous eutherian mammals	2015	Naturwissenschaften
Miranda R.G.; Santos E.B.; Souto R.M.; Gusman H.; Colombo A.P.V.	Ex vivo antimicrobial efficacy of the EndoVac® system plus photodynamic therapy associated with calcium hydroxide against intracanal Enterococcus faecalis	2013	International Endodontic Journal
Nascimento E.H.L.; Abrahão Elias M.R.; Vasconcelos V.H.F.; Haiter-Neto F.; Mendonça E.F.; Sousa T.O.	Ex Vivo Detection of Apical Delta in Premolars: A Comparative Study Using Periapical Radiography, Cone-beam Computed Tomography, and Micro–computed Tomography	2019	Journal of Endodontics
da Silva Paiva SM, Fontana CE, Pedro Rocha DG, de Martin AS, Delboni MG, da Silveira Bueno CE.	Ex Vivo Evaluation of Bacterial Leakage and Coronal Sealing Capacity of Six Materials in Endodontically Treated Teeth	2022	Iran Endod J
Khan S.I.R., Ramachandran A., Alfadley A., Baskaradoss J.K.	Ex vivo fracture resistance of teeth restored with glass and fiber reinforced composite resin	2018	Journal of the Mechanical Behavior of Biomedical Materials
Nascimento EHL, Abrahão Elias MR, Vasconcelos VHF, Haiter-Neto F, Mendonça EF, Sousa TO.	Ex Vivo Detection of Apical Delta in Premolars: A Comparative Study Using Periapical Radiography, Cone-beam Computed Tomography, and Micro-computed Tomography	2019	J Endod
Li L.; Ge J.	Exosome-derived lncRNA-Ankrd26 promotes dental pulp restoration by regulating miR-150-TLR4 signaling	2022	Molecular Medicine Reports
Yu S.; Chen X.; Liu Y.; Zhuang X.Y.; Wang A.C.; Liu X.M.; Zhu S.	Exosomes derived from stem cells from the apical papilla alleviate inflammation in rat pulpitis by upregulating regulatory T cells	2022	International Endodontic Journal
Carminatti M.; Benetti F.; Siqueira R.L.; Zanotto E.D.; Briso A.L.F.; Chaves-Neto A.H.; Cintra L.T.A.	Experimental gel containing bioactive glass-ceramic to minimize the pulp damage caused by dental bleaching in rats	2020	Journal of Applied Oral Science
Vieira F.P.; Júnior A.G.; Piva E.; Oliveira H.D.L.; da Rosa W.L.D.O.; da Silva A.F.; Vitti R.P.; Zanchi C.H.; Cava S.D.S.	Experimental resin-based dual-cured calcium aluminate and calcium titanate materials for vital pulp therapy	2022	Brazilian Oral Research
Pedano M.S.; Yoshihara K.; Li X.; Camargo B.; Van Landuyt K.; Van Meerbeek B.	Experimental resin-modified calcium-silicate cement containing N-(2-hydroxyethyl) acrylamide monomer for pulp tissue engineering	2021	Materials Science and Engineering C
Taing M.-W.; Firth N.; Ford P.J.; Freeman C.R.	Exploring oral healthcare management across Australian community pharmacies using case vignettes	2019	Community Dentistry and Oral Epidemiology
Almaghrabi M.A.; Albadawi E.A.; Dahlan M.A.; Aljohani H.R.; Ahmed N.M.; Showlag R.A.	Exploring Parent's Satisfaction and the Effectiveness of Preformed Metal Crowns Fitting by Hall Technique for Carious Primary Molars in Jeddah Region, Saudi Arabia: Findings of a Prospective Cohort Study	2022	Patient Preference and Adherence
He L.; Liu H.; Shi L.; Pan S.; Yang X.; Zhang L.; Niu Y.	Expression and localization of special AT-rich sequence binding protein 2 in murine molar development and the pulp-dentin complex of human healthy teeth and teeth with pulpitis	2017	Experimental and Therapeutic Medicine

Qi S.; Qian J.; Chen F.; Zhou P.; Yue J.; Tang F.; Zhang Y.; Gong S.; Shang G.; Cui C.; Xu Y.	Expression of autophagy-associated proteins in rat dental irreversible pulpitis	2019	Molecular Medicine Reports
Castillo-Silva B.E.; Martínez-Jiménez V.; Martínezcastañón G.A.; Medina-Solís C.E.; Aguirre-López E.C.; Castillo-Hernández J.R.; Niño-Martínez N.; Patiño-Marín N.	Expression of calcitonin gene-related peptide and pulp sensitivity tests in irreversible pulpitis	2019	Brazilian Oral Research
Wang L, Sun Z, Liu L, Peng B.	Expression of CX3CL1 and its receptor, CX3CR1, in the development of periapical lesions	2014	Int Endod J
Neto N.L.; Teixeira Marques N.C.; Fernandes A.P.; Rodini C.O.; Silva T.C.; Andrade Moreira Machado M.A.; Oliveira T.M.	Expression of DMP-1 in the human pulp tissue using low level laser therapy	2015	Laser Physics
Le Clerc J.; Tricot-Doleux S.; Pellen-Mussi P.; Pérard M.; Jeanne S.; Pérez F.	Expression of factors involved in dental pulp physiopathological processes by nemotic human pulpal fibroblasts	2018	International Endodontic Journal
Lv S.; Li J.; Feng W.; Liu H.; Du J.; Sun J.; Cui J.; Sun B.; Han X.; Oda K.; Amizuka N.; Xu X.; Li M.	Expression of HMGB1 in the periodontal tissue subjected to orthodontic force application by Waldo's method in mice	2015	Journal of Molecular Histology
He M, Bian Z.	Expression of hypoxia-induced semaphorin 7A correlates with the severity of inflammation and osteoclastogenesis in experimentally induced periapical lesions	2017	Arch Oral Biol
Daltoé M.O.; Paula-Silva F.W.G.; Faccioli L.H.; Gatón-Hernández P.M.; De Rossi A.; Silva L.A.B.	Expression of mineralization markers during pulp response to biodentine and mineral trioxide aggregate	2016	Journal of Endodontics
Sampoerno G.; Sunariani J.; Kuntaman	Expression of NaV-1.7, TNF-α and HSP-70 in experimental flare-up post-extirpated dental pulp tissue through a neuroimmunological approach	2020	Saudi Dental Journal
Kim Y.S.; Jung H.K.; Kwon T.K.; Kim C.S.; Cho J.H.; Ahn D.K.; Bae Y.C.	Expression of transient receptor potential Ankyrin 1 in human dental pulp	2012	Journal of Endodontics
Kim Y.S.; Kim T.H.; McKemy D.D.; Bae Y.C.	Expression of vesicular glutamate transporters in transient receptor potential melastatin 8 (TRPM8)-positive dental afferents in the mouse	2015	Neuroscience
Yang E.S.; Jin M.U.; Hong J.H.; Kim Y.S.; Choi S.Y.; Kim T.H.; Cho Y.S.; Bae Y.C.	Expression of vesicular glutamate transporters VGLUT1 and VGLUT2 in the rat dental pulp and trigeminal ganglion following inflammation	2014	PLoS ONE
Jungbluth H.; Brune L.; Lalaouni D.; Winter J.; Jepsen S.	Expression Profiling of S100 Proteins in Healthy and Irreversibly Inflamed Human Dental Pulps	2022	Journal of Endodontics
Bolhari B, Meraji N, Nosrat A.	Extensive idiopathic external root resorption in first maxillary molar: a case report	2013	Iran Endod J
Zhang S.; Thiebes A.L.; Kreimendahl F.; Ruetten S.; Buhl E.M.; Wolf M.; Jockenhoevel S.; Apel C.	Extracellular vesicles-loaded fibrin gel supports rapid neovascularization for dental pulp regeneration	2020	International Journal of Molecular Sciences
Sebastian M.V.; Leavy P.	Extra-oral cutaneous sinus tract secondary to chronic dental infection in an 8-year-old: An uncommon presentation leading to delayed diagnosis and subsequent treatment	2021	Journal of Paediatrics and Child Health
Ricucci D.; Loghin S.; Siqueira Jr. J.F.	Exuberant biofilm infection in a lateral canal as the cause of short-term endodontic treatment failure: Report of a case	2013	Journal of Endodontics
Hu Z.; Chen Y.; He J.; Liu H.; Hui T.	EZH2 Might Affect Macrophage Chemotaxis and Anti-Inflammatory Factors by Regulating CCL2 in Dental Pulp Inflammation	2021	Stem Cells International
He J.; Qin M.; Chen Y.; Hu Z.; Ye L.; Hui T.	EZH2 Promotes Extracellular Matrix Degradation via Nuclear Factor-κB (NF-κB) and p38 Signaling Pathways in Pulpitis	2021	Inflammation
Hui T.; Peng A.; Zhao Y.; Yang J.; Ye L.; Wang C.	EZH2 regulates dental pulp inflammation by direct effect on inflammatory factors	2018	Archives of Oral Biology
Hui T.; Peng A.; Zhao Y.; Wang C.; Gao B.; Zhang P.; Wang J.; Zhou X.; Ye L.	EZH2, a potential regulator of dental pulp inflammation and regeneration	2014	Journal of Endodontics
Li Y.; Liang Q.; Lin C.; Li X.; Chen X.; Hu Q.	Facile synthesis and characterization of novel rapid-setting spherical sub-micron bioactive glasses cements and their biocompatibility in vitro	2017	Materials Science and Engineering C
Aryal YP, Yeon CY, Kim TY, Lee ES, Sung S, Pokharel E, Kim JY, Choi SY,	Facilitating Reparative Dentin Formation Using Apigenin Local Delivery in the Exposed Pulp Cavity	2021	Front Physiol

Yamamoto H, Sohn WJ, Lee Y, An SY, An CH, Jung JK, Ha JH, Kim JY.			
Golpayegani M.V., Behnia H., Araghi M.A., Ansari G.	Factor XIII deficiency, review of the literature and report of a case	2016	Journal of Comprehensive Pediatrics
El Batawi H.Y.	Factors affecting clinical outcome following treatment of early childhood caries under general anaesthesia: a two-year follow-up	2014	European archives of paediatric dentistry : official journal of the European Academy of Paediatric Dentistry
Nimako-Boateng J.; Owusu-Antwi M.; Nortey P.	Factors affecting dental diseases presenting at the University of Ghana Hospital	2016	SpringerPlus
Lipski M, Nowicka A, Kot K, Postek-Stefańska L, Wysoczańska-Jankowicz I, Borkowski L, Andersz P, Jarząbek A, Grocholewicz K, Sobolewska E, Woźniak K, Droździk A.	Factors affecting the outcomes of direct pulp capping using Biodentine	2018	Clin Oral Investig
Duncan HF, El-Karim I, Dummer PMH, Whitworth J, Nagendrababu V.	Factors that influence the outcome of pulpotomy in permanent teeth	2023	Int Endod J
Lee C., Song M.	Failure of Regenerative Endodontic Procedures: Case Analysis and Subsequent Treatment Options	2022	Journal of endodontics
Sanusi S.Y.; Jamaludin S.A.; Al-Batayneh O.B.; Sinor M.Z.; Khamis M.F.	Fate of pulpotomized teeth in pediatric patients: A 3-year case series in a Malaysian Dental Teaching Hospital	2020	International Journal of Clinical Pediatric Dentistry
Olczak-Kowalczyk D.; Samul M.; Góra J.; Gozdowski D.; Turska-Szybka A.	Ferric Sulfate and Formocresol pulpotomies in paediatric dental practice. A prospective- retrospective study	2019	European Journal of Paediatric Dentistry
El-Habashy L.M.; El Meligy O.A.	Fiberglass crowns versus preformed metal crowns in pulpotomized primary molars: a randomized controlled clinical trial	2020	Quintessence international (Berlin, Germany : 1985)
Ducret M.; Costantini A.; Gobert S.; Farges J.-C.; Bekhouche M.	Fibrin-based scaffolds for dental pulp regeneration: From biology to nanotherapeutics	2021	European Cells and Materials
Leite M.L.; Soares D.G.; Anovazzi G.; Anselmi C.; Hebling J.; de Souza Costa C.A.	Fibronectin-loaded Collagen/Gelatin Hydrogel Is a Potent Signaling Biomaterial for Dental Pulp Regeneration	2021	Journal of Endodontics
Chien PY, Walsh LJ, Peters OA.	Finite element analysis of rotary nickel-titanium endodontic instruments: A critical review of the methodology	2021	Eur J Oral Sci
Cardoso J.G.; de Azevedo C.L.; Barroso A.P.; Marion J.J.C.; Duque T.M.; do Prado M.	First mandibular premolar with three canals: Case report	2017	Dental Press Endodontics
Paul S.; Rao A.; Chalakkal P.; Moni S.	Fixed appliance with expansion screw for the treatment of primary anterior crossbite	2016	Journal of Clinical and Diagnostic Research
Yonehiro J.; Yoshida Y.; Yamashita A.; Yoshizawa S.; Ohta K.; Kamata N.; Okihara T.; Nishimura F.	Flavonol-containing phosphorylated pullulan may attenuate pulp inflammation	2013	International Endodontic Journal
Primožič P.K.; Žagar Ž.; Šmalc K.; Račnik J.; Švara T.; Nemec A.	Follow Up on Simple (Closed) Extraction of Fractured Maxillary Canine Teeth in Domestic Ferrets (Mustela putorius furo)	2021	Frontiers in Veterinary Science
Llena C., Hernández M., Melo M., Sanz J.L., Forner L.	Follow-up of patients subjected to direct and indirect pulp capping of young permanent teeth. A retrospective study	2021	Clinical and experimental dental research
Balata G.F.; Abdelhady M.I.S.; Mahmoud G.M.; Matar M.A.; Abd El-Latif A.N.	Formulation of saudi propolis into biodegradable chitosan chips for vital pulpotomy	2018	Current Drug Delivery
Giorgini E.; Sabbatini S.; Conti C.; Rubini C.; Rocchetti R.; Fioroni M.; Memè L.; Orilisi G.	Fourier Transform Infrared Imaging analysis of dental pulp inflammatory diseases	2017	Oral Diseases
Li Y.; Wang H.; Pei F.; Chen Z.; Zhang L.	FoxO3a Regulates Inflammation-induced Autophagy in Odontoblasts	2018	Journal of Endodontics
Hasna AA, Pinto ABA, Coelho MS, de Andrade GS, Tribst JPM, de Castro Lopes SLP, Carvalho CAT, Borges ALS.	Fracture resistance and biomechanical behavior of different access cavities of maxillary central incisors restored with different composite resins	2022	Clin Oral Investig
Venkataraman K., Venkatapathi A., Balasubramanian S., Palanivelu C., Swaminathan S., Samuel L., Thanapathi S.	Fracture resistance of endodontically treated mandibular permanent first molars reinforced with diagonal horizontal postdesign: A in vitro study	2021	Journal of Pharmacy and Bioallied Sciences

Lucas T.J., Lawson N.C., Englert B., Goldstein K., Goldstein R.	Fracture strength of zirconia and lithium disilicate restorations following endodontic access	2022	Journal of Esthetic and Restorative Dentistry
Umesi D.C., Oremosu O.A., Makanjuola J.O., Nwachukwu N.C.	Frequency and distribution of teeth treated by single- and multiple- visit root canal treatment in a Nigerian population by differently skilled operators	2016	Odonto-stomatologie tropicale = Tropical dental journal
Bernal-Treviño A.; González-Amaro A.M.; Méndez González V.; Pozos-Guillen A.	Frequency of Candida in root canals of teeth with primary and persistent endodontic infections; Frecuencia de Candida en conductos radiculares de dientes con infección endodóntica primaria y persistente	2018	Revista Iberoamericana de Micologia
Boteva E.; Karayasheva D.; Peycheva K.	Frequency of iatrogenic changes caused from overhang restorations	2015	Acta Medica Bulgarica
Falcão C.A.; Albuquerque V.C.; Amorim N.L.; Freitas S.A.; Santos T.C.; Matos F.T.; Ferraz M.A.	Frequency of the mesiopalatal canal in upper first permanent molars viewed through computed tomography	2016	Acta odontologica latinoamericana : AOL
Mouawad S.; Artine S.; Hajjar P.; McConnell R.; Fahd J.-C.; Sabbagh J.	Frequently asked questions in direct pulp capping of permanent teeth	2014	Dental Update
Pedano M.S.; Li X.; Li S.; Sun Z.; Cokic S.M.; Putzeys E.; Yoshihara K.; Yoshida Y.; Chen Z.; Van Landuyt K.; Van Meerbeek B.	Freshly-mixed and setting calcium-silicate cements stimulate human dental pulp cells	2018	Dental Materials
Chevalier V, Le Fur Bonnabesse A, Duncan HF.	Frightened of the pulp? A qualitative analysis of undergraduate student confidence and stress during the management of deep caries and the exposed pulp	2021	Int Endod J
Basutkar N., Alamoudi R.K., Alharbi R.M.	Full mouth rehabilitation of a patient with restorative space issues-a case report	2020	Asian Journal of Pharmaceutical Research and Health Care
Gada S., Nallaswamy D., Jain A.R.	Full mouth rehabilitation of periodontally compromised partially edentulous arches with multiple missing teeth using telescopic crown and bridge - A case report	2018	Drug Invention Today
Taha N.A., Abdulkhader S.Z.	Full Pulpotomy with Biodentine in Symptomatic Young Permanent Teeth with Carious Exposure	2018	Journal of endodontics
Cristofanilli M.; Turner N.C.; Bondarenko I.; Ro J.; Im S.-A.; Masuda N.; Colleoni M.; DeMichele A.; Loi S.; Verma S.; Iwata H.; Harbeck N.; Zhang K.; Theall K.P.; Jiang Y.; Bartlett C.H.; Koehler M.; Slamon D.	Fulvestrant plus palbociclib versus fulvestrant plus placebo for treatment of hormone-receptor-positive, HER2-negative metastatic breast cancer that progressed on previous endocrine therapy (PALOMA-3): final analysis of the multicentre, double-blind, phase 3 randomised controlled trial	2016	The Lancet Oncology
Cui S.-J.; Fu Y.; Yu M.; Zhang L.; Zhao W.-Y.; Zhang T.; Qiu L.-X.; Gu Y.; Zhou Y.-H.; Liu Y.	Functional periodontal regeneration using biomineralized extracellular matrix/stem cell microspheroids	2022	Chemical Engineering Journal
Ahn D.K.; Doutova E.A.; McNaughton K.; Light A.R.; Närhi M.; Maixner W.	Functional properties of tooth pulp neurons responding to thermal stimulation	2012	Journal of Dental Research
Hosokawa Y.; Hirao K.; Yumoto H.; Washio A.; Nakanishi T.; Takegawa D.; Kitamura C.; Matsuo T.	Functional Roles of NOD1 in Odontoblasts on Dental Pulp Innate Immunity	2016	BioMed Research International
Piva E.; Silva A.F.; Nör J.E.	Functionalized scaffolds to control dental pulp stem cell fate	2014	Journal of Endodontics
Ben Salem M.; Chouchene F.; Masmoudi F.; Baaziz A.; Maatouk F.; Ghedira H.	Fusion or Gemination? Diagnosis and Management in Primary Teeth: A Report of Two Cases	2021	Case Reports in Dentistry
Kulakowski D, Phansalkar RM, Leme-Kraus AA, McAlpine J, Chen SN, Pauli GF, Ravindran S, Bedran-Russo AK.	Galloylated proanthocyanidins in dentin matrix exhibit biocompatibility and induce differentiation in dental stem cells	2022	J Bioact Compat Polym
Venkatesh A., Mitthra S., Prakash V., Prasad T.S.	Gemination or fusion?: A case report	2016	Biomedical and Pharmacology Journal
Ohkura N.; Shigetani Y.; Yoshiba N.; Yoshiba K.; Okiji T.	Gene expression analysis of membrane transport proteins in normal and lipopolysaccharide-inflamed rat dental pulp	2012	Journal of Endodontics
Hasan A, Roome T, Wahid M, Ansari SA, Akhtar H, Jilani SNA, Kiyani A.	Gene expression analysis of toll like receptor 2 and 4, Dectin-1, Osteopontin and inflammatory cytokines in human dental pulp ex-vivo	2022	BMC Oral Health
Xiao J.; Zheng Y.; Zhang W.; Zhang Y.; Cao P.; Liang Y.; Bao L.; Shi S.; Feng X.	General Control Nonrepressed Protein 5 Modulates Odontogenic Differentiation Through NF-κB Pathway in Tumor Necrosis Factor-α-Mediated Impaired Human Dental Pulp Stem Cells	2022	Cellular Reprogramming

Ajith A.; Subbiah U.; Subbiah H.	Genetic analysis in pain associated deep caries	2019	Indian Journal of Public Health Research and Development
Lucas Leite A.C.G.; Rosenblatt A.; Da Silva Calixto M.; Da Silva C.M.; Santos N.	Genotoxic effect of formocresol pulp therapy of deciduous teeth	2012	Mutation Research - Genetic Toxicology and Environmental Mutagenesis
Chen G.; Sung P.-T.	Gingival and localized alveolar bone necrosisrelated to the use of arsenic trioxidepaste-Two case reports	2014	Journal of the Formosan Medical Association
Sesiliana M.; Riyanti E.	Giomer s-prg technology as an alternative restoration in early childhood caries	2021	World Journal of Dentistry
Singhal I., Dave A., Arora M., Saluja P.	Glandular odontogenic cyst - An unusual presentation in the maxilla	2021	Journal of Clinical and Diagnostic Research
Xiao N.; Yu W.Y.; Liu D.	Glial cell-derived neurotrophic factor promotes dental pulp stem cell migration	2018	Journal of Tissue Engineering and Regenerative Medicine
Tohma A, Ohkura N, Yoshiba K, Takeuchi R, Yoshiba N, Edanami N, Shirakashi M, Ibn Belal RS, Ohshima H, Noiri Y.	Glucose Transporter 2 and 4 Are Involved in Glucose Supply during Pulpal Wound Healing after Pulpotomy with Mineral Trioxide Aggregate in Rat Molars	2020	J Endod
Piccin A., Tagnin M., Vecchiato C., Al-Khaffaf A., Beqiri L., Kaiser C., Agreiter I., Negri G., Kob M., Di Pierro A., Vittadello F., Mazzoleni G., Eisendle K., Fontanella F.	Graft-versus-host disease (GvHD) of the tongue and of the oral cavity: a large retrospective study	2018	International Journal of Hematology
Sun N.; Yin S.; Lu Y.; Zhang W.; Jiang X.	Graphene oxide-coated porous titanium for pulp sealing: An antibacterial and dentino-inductive restorative material	2020	Journal of Materials Chemistry B
Casey M.B.; Pearson G.R.; Perkins J.D.; Tremaine W.H.	Gross, computed tomographic and histological findings in mandibular cheek teeth extracted from horses with clinical signs of pulpitis due to apical infection	2015	Equine Veterinary Journal
Zhang T., Chen D., Miao L.-Y., Xie S.-J., Tang X.-N.	Guided endodontic access of calcified root canal by laser melting templates	2020	Hua xi kou qiang yi xue za zhi = Huaxi kouqiang yixue zazhi = West China journal of stomatology
Krug R.; Volland J.; Reich S.; Soliman S.; Connert T.; Krastl G.	Guided endodontic treatment of multiple teeth with dentin dysplasia: a case report	2020	Head and Face Medicine
Gonçalves W.F., Garcia L.D.F.R., Vieira-Schuldt D.P., Bortoluzzi E.A., Dias-Júnior L.C.L., Teixeira C.D.S.	Guided Endodontics in Root Canals with Complex Access: Two Case Reports	2021	Brazilian dental journal
Leontiev W, Connert T, Weiger R, Dagassan-Berndt D, Krastl G, Magni E.	Guided Endodontics: Three-dimensional Planning and Template-aided Preparation of Endodontic Access Cavities	2022	J Vis Exp
Loureiro MAZ, Elias MRA, Capeletti LR, Silva JA, Siqueira PC, Chaves GS, Decurcio DA.	Guided Endodontics: Volume of Dental Tissue Removed by Guided Access Cavity Preparation-An Ex Vivo Study	2020	J Endod
Duarte Campos D.F.; Zhang S.; Kreimendahl F.; Köpf M.; Fischer H.; Vogt M.; Blaeser A.; Apel C.; Esteves-Oliveira M.	Hand-held bioprinting for de novo vascular formation applicable to dental pulp regeneration	2020	Connective Tissue Research
El-Zekrid M.H.; Mahmoud S.H.; Ali F.A.; Helal M.E.; Grawish M.E.	Healing Capacity of Autologous Bone Marrow–derived Mesenchymal Stem Cells on Partially Pulpotomized Dogs' Teeth	2019	Journal of Endodontics
Sharma L.A.; Love R.M.; Ali M.A.; Sharma A.; Macari S.; Avadhani A.; Dias G.J.	Healing response of rat pulp treated with an injectable keratin hydrogel	2017	Journal of Applied Biomaterials and Functional Materials
Dahake PT, Thosar N, Hande A, Joshi DA, Bhagat A.	Hematological and Biochemical Responses of Newly Formulated Primary Root Canal Obturating Material: An In Vivo Study	2022	Cureus
Rajasekar P., Mitthra S., Malarvizhi D., Subbiya A.	Hemisection of mandibular first molar: Clinical management—a case report	2019	Indian Journal of Public Health Research and Development
Han Y.; Koohi-Moghadam M.; Chen Q.; Zhang L.; Chopra H.; Zhang J.; Dissanayaka W.L.	HIF-1α Stabilization Boosts Pulp Regeneration by Modulating Cell Metabolism	2022	Journal of Dental Research
Asghari M.; Nasoohi N.; Hodjat M.	High glucose promotes the aging of human dental pulp cells through Wnt/beta-catenin signaling	2021	Dental and Medical Problems

Colombo J.S.; Malik H.; Caranto C.A.; Allen P.K.; Howard J.; Carlson K.	Highly targeted electrochemical disruption of microbes with minimal disruption to pulp cells	2022	Journal of Dentistry
Liu L, Deng J, Ji Q, Peng B.	High-mobility Group Box 1 Is Associated with the Inflammatory Infiltration and Alveolar Bone Destruction in Rats Experimental Periapical Lesions	2017	J Endod
Kushnerev E.; Shawcross S.G.; Hillarby M.C.; Yates J.M.	High-plasticity mesenchymal stem cells isolated from adult-retained primary teeth and autogenous adult tooth pulp - A potential source for regenerative therapies?	2016	Archives of Oral Biology
Wu Y.; Zhou C.; Tong X.; Li S.; Liu J.	Histochemical localization of putative stem cells in irreversible pulpitis	2022	Oral Diseases
Lin L.M.; Shimizu E.; Gibbs J.L.; Loghin S.; Ricucci D.	Histologic and histobacteriologic observations of failed revascularization/revitalization therapy: A case report	2014	Journal of Endodontics
Woodmansey K.F.; Kohout G.D.; Primus C.M.; Schneiderman E.; Opperman L.A.	Histologic assessment of quick-set and mineral trioxide aggregate pulpotomies in a canine model	2015	Journal of Endodontics
Lazzaretti D.N.; Bortoluzzi G.S.; Torres Fernandes L.F.; Rodriguez R.; Grehs R.A.; Martins Hartmann M.S.	Histologic evaluation of human pulp tissue after orthodontic intrusion	2014	Journal of Endodontics
Peng C.; Zhao Y.; Wang W.; Yang Y.; Qin M.; Ge L.	Histologic Findings of a Human Immature Revascularized/Regenerated Tooth with Symptomatic Irreversible Pulpitis	2017	Journal of Endodontics
Shimizu E.; Jong G.; Partridge N.; Rosenberg P.A.; Lin L.M.	Histologic observation of a human immature permanent tooth with irreversible pulpitis after revascularization/regeneration procedure	2012	Journal of Endodontics
Ricucci D.; Grande N.M.; Plotino G.; Tay F.R.	Histologic Response of Human Pulp and Periapical Tissues to Tricalcium Silicate–based Materials: A Series of Successfully Treated Cases	2020	Journal of Endodontics
Mehrdad L, Malekafzali B, Shekarchi F, Safi Y, Asgary S.	Histological and CBCT evaluation of a pulpotomised primary molar using calcium enriched mixture cement	2013	Eur Arch Paediatr Dent
Lourenco-Neto N.; Marques N.C.T.; Prado M.T.O.; Vitor L.L.R.; Rodini C.O.; Machado M.A.A.M.; Oliveira T.M.	Histological and cd31 immunolocalization after pulp therapy using mta or portland cement	2018	International Journal of Dentistry and Oral Science
Puşcaşu C.G.; Ştefănescu C.L.; Murineanu R.M.; Grigorian M.; Petcu L.C.; Dumea E.; Sachelarie L.; Puşcaşu R.A.	Histological aspects regarding dental pulp of diabetic patients	2021	Applied Sciences (Switzerland)
Mohammad S.G.; Raheel S.A.; Baroudi K.	Histological Evaluation of Allium sativum Oil as a New Medicament for Pulp Treatment of Permanent Teeth	2015	The journal of contemporary dental practice
Suzuki M.; Taira Y.; Kato C.; Shinkai K.; Katoh Y.	Histological evaluation of direct pulp capping of rat pulp with experimentally developed low-viscosity adhesives containing reparative dentin-promoting agents	2016	Journal of Dentistry
Hadi R.J.; AL-Ghaban N.M.H.	HISTOLOGICAL EVALUATION OF PULP RESPONSE TO IN-OFFICE BLEACHING TECHNIQUE IN RATS	2020	Biochemical and Cellular Archives
Cupertino R.R.; Fabri F.V.; Veltrini V.C.; Hidalgo M.M.; Bruschi M.L.; de Oliveira R.M.M.W.	Histological evaluation of the rat dental pulp after indirect capping with sildenafil or L-NAME incorporated into a bioadhesive thermoresponsive system; Avaliação histológica da polpa dental do rato após capeamento pulpar indireto usando sildenafil ou L-NAME incorporados a um sistema bioadesivo termorresponsivo	2016	Acta Scientiarum - Health Sciences
Walsh R.M.; Woodmansey K.F.; He J.; Svoboda K.K.; Primus C.M.; Opperman L.A.	Histology of NeoMTA Plus and Quick-Set2 in Contact with Pulp and Periradicular Tissues in a Canine Model	2018	Journal of Endodontics
Li M.; Tian J.; Xu Z.; Zeng Q.; Chen W.; Lei S.; Wei X.	Histology-based profile of inflammatory mediators in experimentally induced pulpitis in a rat model: screening for possible biomarkers	2021	International Endodontic Journal
Madani ZS, Haddadi A, Mesgarani A, Seyedmajidi M, Mostafazadeh A, Bijani A, Ashraphpour M.	Histopathologic Responses of the Dental Pulp to Calcium-Enriched Mixture (CEM) and Mineral Trioxide Aggregate (MTA) in Diabetic and Non-Diabetic Rats	2014	Int J Mol Cell Med
Tobias Duarte P.C.; Gomes-Filho J.E.; Ervolino E.; Marçal Mazza Sundefeld M.L.; Tadahirowayama M.; Lodi C.S.; Dezan-Júnior E.; Angelo Cintra L.T.	Histopathological condition of the remaining tissues after endodontic infection of rat immature teeth	2014	Journal of Endodontics

Oliadarani F.K.; Haghgoo R.; Mashhadiabbas F.; Kahvand M.	Histopathological evaluation of dental pulp of primary teeth pulpotomized with formocresol with/without a capping agent: A randomized clinical trial	2018	Journal of International Society of Preventive and Community Dentistry
Alzoubi H.; Bshara N.; AL-Manadili A.	Histopathological evaluation of pulp response to portland cement compared to MTA after primary canines pulpotomy (in vivo study)	2022	BDJ Open
Sousa M.G.C.; Xavier P.D.; Cantuária A.P.D.C.; Porcino R.A.; Almeida J.A.; Franco O.L.; Rezende T.M.B.	Host defense peptide IDR-1002 associated with ciprofloxacin as a new antimicrobial and immunomodulatory strategy for dental pulp revascularization therapy	2021	Microbial Pathogenesis
Al Masri A, Abudrya MEH, Splieth CH, Schmoeckel J, Mourad MS, Santamaría RM.	How did the COVID-19 pandemic lockdown affect dental emergency care in children? Retrospective study in a specialized pedodontic center	2021	Quintessence Int
Careddu R.; Duncan H.F.	How does the pulpal response to biodentine and proroot mineral trioxide aggregate compare in the laboratory and clinic?	2018	British Dental Journal
Chompu-Inwai P.; Sutharaphan T.; Nirunsittirat A.; Chuveera P.; Srisuwan T.; Sastraruji T.	How effective are inferior alveolar nerve block and supplemental intraligamentary injections in pediatric patients with deep carious permanent mandibular molars?	2018	Pediatric Dentistry
Edwards D, Bailey O, Stone SJ, Duncan H.	How is carious pulp exposure and symptomatic irreversible pulpitis managed in UK primary dental care?	2021	Int Endod J
Splieth CH, Banerjee A, Bottenberg P, Breschi L, Campus G, Ekstrand KR, Giacaman RA, Haak R, Hannig M, Hickel R, Juric H, Lussi A, Machiulskiene V, Manton DJ, Jablonski-Momeni A, Opdam NJM, Paris S, Santamaría RM, Schwendicke F, Tassery H, Ferreira Zandona A, Zero DT, Zimmer S, Doméjean S.	How to Intervene in the Caries Process in Children: A Joint ORCA and EFCD Expert Delphi Consensus Statement	2020	Caries Res
Santamaría RM, Abudrya MH, Gül G, Mourad MS, Gomez GF, Zandona AGF.	How to Intervene in the Caries Process: Dentin Caries in Primary Teeth	2020	Caries Res
Sugiuchi A.; Sano Y.; Furusawa M.; Abe S.; Muramatsu T.	Human Dental Pulp Cells Express Cellular Markers for Inflammation and Hard Tissue Formation in Response to Bacterial Information	2018	Journal of Endodontics
Ahmadi P.; Yan M.; Bauche A.; Smeets R.; Müller C.E.; Koch-Nolte F.; Haag F.; Fliegert R.; Kluwe L.; Schulze zur Wiesch J.; Hartjen P.	Human dental pulp cells modulate CD8+ T cell proliferation and efficiently degrade extracellular ATP to adenosine in vitro	2022	Cellular Immunology
Manaspon C.; Jongwannasiri C.; Chumprasert S.; Sa-Ard-Iam N.; Mahanonda R.; Pavasant P.; Porntaveetus T.; Osathanon T.	Human dental pulp stem cell responses to different dental pulp capping materials	2021	BMC Oral Health
Lam C.; Alsaeedi H.A.; Koh A.E.-H.; Harun M.H.N.; Hwei A.N.M.; Mok P.L.; Luu C.D.; Yong T.K.; Subbiah S.K.; Bastion M.-L.C.	Human Dental Pulp Stem Cells (DPSCs) Therapy in Rescuing Photoreceptors and Establishing a Sodium Iodate-Induced Retinal Degeneration Rat Model	2021	Tissue Engineering and Regenerative Medicine
Al-Serwi R.H.; El-Kersh A.O.F.O.; El-Akabawy G.	Human dental pulp stem cells attenuate streptozotocin-induced parotid gland injury in rats	2021	Stem Cell Research and Therapy
Tran H.L.B.; Doan V.N.	Human dental pulp stem cells cultured onto dentin derived scaffold can regenerate dentin-like tissue in vivo	2015	Cell and Tissue Banking
Janowicz K.; Mozdziak P.; Bryja A.; Kempisty B.; Dyszkiewicz-Konwińska M.	Human Dental Pulp Stem Cells: Recent findings and current research	2019	Medical Journal of Cell Biology
De Souza P.V.; Alves F.B.T.; Costa Ayub C.L.S.; De Miranda Soares M.A.; Gomes J.R.	Human immature dental pulp stem cells (hIDPSCs), their application to cell therapy and bioengineering: An analysis by systematic revision of the last decade of literature	2013	Anatomical Record
Bimstein E, Damm D.	Human Primary Tooth Histology Six Months after Treatment with Silver Diamine Fluoride	2018	J Clin Pediatr Dent
Bakhtiar H.; Nekoofar M.H.; Aminishakib P.; Abedi F.; Naghi Moosavi F.; Esnaashari E.; Azizi A.; Esmailian S.; Ellini M.R.; Mesgarzadeh V.; Sezavar M.; About I.	Human Pulp Responses to Partial Pulpotomy Treatment with TheraCal as Compared with Biodentine and ProRoot MTA: A Clinical Trial	2017	Journal of Endodontics
Umemura N.; Ohkoshi E.; Tajima M.; Kikuchi H.; Katayama T.; Sakagami H.	Hyaluronan induces odontoblastic differentiation of dental pulp stem cells via CD44	2016	Stem Cell Research and Therapy

Haghani J.; Haghani F.; Soleimani A.; Abbasnejad M.; Khodami M.; Kooshki R.; Raoof M.	Hydroalcoholic extracts of three Artemisia species attenuate dental pulp pain and pain-related abnormal feeding behavior of rats	2022	Iranian Journal of Veterinary Science and Technology
Saghiri M.A.; Asatourian A.; Nguyen E.H.; Wang S.; Sheibani N.	Hydrogel Arrays and Choroidal Neovascularization Models for Evaluation of Angiogenic Activity of Vital Pulp Therapy Biomaterials	2018	Journal of Endodontics
Yeh C.-K.; Harris S.E.; Mohan S.; Horn D.; Fajardo R.; Chun Y.-H.P.; Jorgensen J.; MacDougall M.; Abboud-Werner S.	Hyperglycemia and xerostomia are key determinants of tooth decay in type 1 diabetic mice	2012	Laboratory Investigation
Ye X, Zhang J, Yang P.	Hyperlipidemia induced by high-fat diet enhances dentin formation and delays dentin mineralization in mouse incisor	2016	J Mol Histol
Pérez Jardón A., Otero Gayoso N., Otero. Rey E.M., Guerra Caamaño M., Chamorro-Petronacci C.M., Blanco Carrión A., Rivas Mundiña B.	Hyperplastic Pulpitis Management with Endocrown: A Case Report	2022	Open Dentistry Journal
Yamada M.; Nagayama M.; Katsumata A.; Kawano S.; Gen K.; Ehara M.; Nakao J.; Tanuma J.-I.; Yoshida T.	Hypomineralized Enamel of Dens Invaginatus: Its distinct images and pathogenesis of the type III invagination using micro-focusing computed tomography	2014	Journal of Hard Tissue Biology
Zhu S.; Ying Y.; He Y.; Zhong X.; Ye J.; Huang Z.; Chen M.; Wu Q.; Zhang Y.; Xiang Z.; Tu Y.; Ying W.; Xiao J.; Li X.; Ye Q.; Wang Z.	Hypoxia response element-directed expression of bFGF in dental pulp stem cells improve the hypoxic environment by targeting pericytes in SCI rats	2021	Bioactive Materials
Liu Y.; Chen L.; Gong Q.; Jiang H.; Huang Y.	Hypoxia-induced mitophagy regulates proliferation, migration and odontoblastic differentiation of human dental pulp cells through FUN14 domain-containing 1	2022	International Journal of Molecular Medicine
Wu Y.; Huang F.; Zhou X.; Yu S.; Tang Q.; Li S.; Wang J.; Chen L.	Hypoxic Preconditioning Enhances Dental Pulp Stem Cell Therapy for Infection-Caused Bone Destruction	2016	Tissue Engineering - Part A
Issrani R., Prabhu N.K., Alam M.	Iatrogenic injury of facial skin due to formocresol: A case report	2020	Journal of Cutaneous and Aesthetic Surgery
Watts K.; Balzer S.; Drum M.; Nusstein J.; Reader A.; Fowler S.; Beck M.	Ibuprofen and Acetaminophen Versus Intranasal Ketorolac (Sprix) in an Untreated Endodontic Pain Model: A Randomized, Double-blind Investigation	2019	Journal of Endodontics
Sakthivel M.; Ayyasamy P.M.; Arvind Prasanth D.	Identification and antimicrobial susceptibility testing of pathogenic micro-organism from dental patients	2016	Asian Journal of Pharmaceutical and Clinical Research
Wang J.; Wei X.; Ling J.; Huang Y.; Gong Q.; Huo Y.	Identification and characterization of side population cells from adult human dental pulp after ischemic culture	2012	Journal of Endodontics
Ray A.; Kundabala M.; Shetty N.	IDENTIFICATION AND MANAGEMENT OF MESIO-CENTRAL CANAL IN MANDIBULAR FIRST MOLAR: A CASE REPORT	2021	International Journal of Clinical Dentistry
Al-Natour B.; Rankin R.; McKenna R.; McMillan H.; Zhang S.-D.; About I.; Khan A.A.; Galicia J.C.; Lundy F.T.; El-Karim I.A.	Identification and validation of novel biomarkers and therapeutics for pulpitis using connectivity mapping	2021	International Endodontic Journal
Rostinawati T.; Hadisoebroto S.; Iskandar Y.; Nugroho P.H.; Tara A.A.	Identification of bacteria causing necrotic pulp with 16S rRNA gene polymerase chain reaction and antibiotic resistance testing at the dental hospital in sekeloa, bandung, indonesia	2017	Asian Journal of Pharmaceutical and Clinical Research
Benyó B.	Identification of dental root canals and their medial line from micro-CT and cone-beam CT records	2012	BioMedical Engineering Online
Zargar N.; Ashraf H.; Marashi S.M.A.; Sabeti M.; Aziz A.	Identification of microorganisms in irreversible pulpitis and primary endodontic infections with respect to clinical and radiographic findings	2020	Clinical Oral Investigations
Matsumura S.; Quispe-Salcedo A.; Schiller C.M.; Shin J.S.; Locke B.M.; Yakar S.; Shimizu E.	IGF-1 Mediates EphrinBl Activation in Regulating Tertiary Dentin Formation	2017	Journal of Dental Research
Li J.; Diao S.; Yang H.; Cao Y.; Du J.; Yang D.	IGFBP5 promotes angiogenic and neurogenic differentiation potential of dental pulp stem cells	2019	Development Growth and Differentiation
Xiong H.; Wei L.; Peng B.	IL-17 stimulates the production of the inflammatory chemokines IL-6 and IL-8 in human dental pulp fibroblasts	2015	International Endodontic Journal
Limjeerajarus C.N.; Chanarattanubol T.; Trongkij P.; Rujiwanichkul M.; Pavasant P.	Iloprost induces tertiary dentin formation	2014	Journal of Endodontics

Limjeerajarus C.N.; Osathanon T.; Manokawinchoke J.; Pavasant P.	Iloprost up-regulates vascular endothelial growth factor expression in human dental pulp cells in vitro and enhances pulpal blood flow in vivo	2014	Journal of Endodontics
Park M.; Ahn B.D.	Immature permanent teeth with apical periodontitis and abscess treated by regenerative endodontic treatment using calcium hydroxide and MTA: a report of two cases	2014	Pediatric dentistry
Marques N.C.T.; Lourenço Neto N.; Oliveira T.M.	Immediate and mediate furcal perforation treatment in primary molars: 24-month follow-up	2016	European Archives of Paediatric Dentistry
Keles S.; Kocaturk O.	Immediate Postoperative Pain and Recovery Time after Pulpotomy Performed under General Anaesthesia in Young Children	2017	Pain Research and Management
Bashar A.K.M.; Kabir A.K.M.N.; Rizdina R.A.; Shikder A.H.M.Z.H.; Ghosh R.; Rahman M.M.	Immediate response of human dental pulp capped with mineral trioxide aggregate, portland cement and biodentin	2019	Bangladesh Medical Research Council Bulletin
Grandi T., Figliuzzi M.M., Signorini L.	Immediately restored single post extractive implants: clinical outcomes of a retrospective analysis with 8-10 years follow-up	2022	Journal of Biological Regulators and Homeostatic Agents
Renard E.; Gaudin A.; Bienvenu G.; Amiaud J.; Farges J.C.; Cuturi M.C.; Moreau A.; Alliot-Licht B.	Immune Cells and Molecular Networks in Experimentally Induced Pulpitis	2016	Journal of Dental Research
Durutürk L.; Sari Ş.; Şengül A.	Immunocompetent cell level as a diagnostic reference for pulpal pathosis of primary teeth	2013	Archives of Oral Biology
Manolea H.; Vasile N.; Opri M.; Fronie A.; Popescu M.R.	Immunohistochemical and electron microscopy aspects of the nerve structures from the dental pulp	2014	Romanian Journal of Morphology and Embryology
Chisini L.A.; Conde M.C.M.; Alcázar J.C.B.; da Silva A.F.; Nör J.E.; Tarquinio S.B.C.; Demarco F.F.	Immunohistochemical Expression of TGF-β1 and Osteonectin in engineered and Ca(OH)2-repaired human pulp tissues	2016	Brazilian Oral Research
Liu S, Li Q, Liu Y.	Immunohistochemical localization of NALP3 inflammasome in experimental periapical lesions	2014	Int Endod J
Dinakar G.; Ganesh A.; Prem Kumar M.S.; Sabesan M.; Narasimhan M.; Deivanayagam K.	Immunohistochemical quantification of mast cells in inflamed and noninflamed pulp tissue	2018	Journal of Oral and Maxillofacial Pathology
Takeuchi R, Ohkura N, Yoshiba K, Tohma A, Yoshiba N, Edanami N, Shirakashi M, Belal RS, Ohshima H, Noiri Y.	Immunohistochemistry and gene expression of GLUT1, RUNX2 and MTOR in reparative dentinogenesis	2020	Oral Dis
Jamshidi D., Homayouni H., Moradi Majd N., Shahabi S., Arvin A., Ranjbar Omidi B.	Impact and Fracture Strength of Simulated Immature Teeth Treated with Mineral Trioxide Aggregate Apical Plug and Fiber Post Versus Revascularization	2018	Journal of endodontics
Jain N., Garg S., Dhindsa A., Joshi S., Khatria H.	Impact of 6% citric acid and endoactivator as irrigation adjuncts on obturation quality and pulpectomy outcome in primary teeth	2019	Pediatric Dental Journal
Ribeiro I.L.A., Bellissimo-Rodrigues W.T., Mussolin M.G., Innocentini L.M.A.R., Marangoni A.T.D., Macedo L.D., Barbosa-Júnior F., de Souza H.C.C., Menegueti M.G., Pereira A.P.S., Gaspar G.G., Schmidt A., Miranda C.H., Lovato W.J., Puga M.L., Auxiliadora-Martins M., Basile-Filho A., Bellissimo-Rodrigues F.	Impact of a dental care intervention on the hospital mortality of critically ill patients admitted to intensive care units: A quasi-experimental study	2022	American Journal of Infection Control
Silva EJNL, Rover G, Belladonna FG, De-Deus G, da Silveira Teixeira C, da Silva Fidalgo TK.	Impact of contracted endodontic cavities on fracture resistance of endodontically treated teeth: a systematic review of in vitro studies	2018	Clin Oral Investig
dos Santos M.B.F.; Pires A.L.C.; Saporiti J.M.; Kinalski M.D.A.; Marchini L.	Impact of COVID-19 pandemic on oral health procedures provided by the Brazilian public health system: COVID-19 and oral health in Brazil	2021	Health Policy and Technology
Beazoglou T.J.; Bailit H.L.; DeVitto J.; McGowan T.; Myne-Joslin V.	Impact of dental therapists on productivity and finances: II. Federally qualified health centers	2012	Journal of Dental Education
Emfietzoglou R, Pachymanolis E, Piperi C.	Impact of Epigenetic Alterations in the Development of Oral Diseases	2021	Curr Med Chem
Ramos-Jorge J, Pordeus IA, Ramos-Jorge ML, Marques LS, Paiva SM.	Impact of untreated dental caries on quality of life of preschool children: different stages and activity	2014	Community Dent Oral Epidemiol

Xia J.; Wang W.; Li Z.; Lin B.; Zhang Q.; Jiang Q.; Yang X.	Impacts of contracted endodontic cavities compared to traditional endodontic cavities in premolars	2020	BMC Oral Health
Marchesan MA, Lloyd A, Clement DJ, McFarland JD, Friedman S.	Impacts of Contracted Endodontic Cavities on Primary Root Canal Curvature Parameters in Mandibular Molars	2018	J Endod
Sueyama Y.; Kaneko T.; Ito T.; Kaneko R.; Okiji T.	Implantation of Endothelial Cells with Mesenchymal Stem Cells Accelerates Dental Pulp Tissue Regeneration/Healing in Pulpotomized Rat Molars	2017	Journal of Endodontics
Gafforov S.A.; Yarieva O.O.	Importance of medical and social factors in etiology of carious and non-carious diseases of children	2019	International Journal of Pharmaceutical Research
Baghdadi Z.D.	Improving oral health status of children in Tabuk, Saudi Arabia	2014	Dentistry Journal
Cantekin K, Gumus H.	In vitro and clinical outcome of sandwich restorations with a bulk-fill flowable composite liner for pulpotomized primary teeth	2014	J Clin Pediatr Dent
Silva J.C.; Rifane T.O.; Ferreira-Junior A.E.; Alves A.P.; Miron R.; Zhang Y.; Fechine P.B.A.; Carvalho E.V.; Feitosa V.P.	In Vitro and in Vivo Efficacy of New Composite for Direct Pulp Capping	2021	BioMed Research International
De Castilho A.R.F.; Duque C.; Negrini T.D.C.; Sacono N.T.; De Paula A.B.; Costa C.A.D.S.; Spolidório D.M.P.; Puppin-Rontani R.M.	In vitro and in vivo investigation of the biological and mechanical behaviour of resin-modified glass-ionomer cement containing chlorhexidine	2013	Journal of Dentistry
Pourhajibagher M., Ranjbar Omrani L., Noroozian M., Ghorbanzadeh Z., Bahador A.	In vitro antibacterial activity and durability of a nano-curcumin-containing pulp capping agent combined with antimicrobial photodynamic therapy	2021	Photodiagnosis and Photodynamic Therapy
Dimitrova-Nakov S.; Uzunoglu E.; Ardila-Osorio H.; Baudry A.; Richard G.; Kellermann O.; Goldberg M.	In vitro bioactivity of Bioroot™ RCS, via A4 mouse pulpal stem cells	2015	Dental Materials
Elbanna A.; Atta D.; Sherief D.	In vitro bioactivity of newly introduced dual-cured resin-modified calcium silicate cement	2022	Dental Research Journal
Zarean P.; Roozbeh R.; Zarean P.; Jahromi M.Z.; Broujeni P.M.	In vitro comparison of shear bond strength of a flowable composite resin and a single-component glass-ionomer to three different pulp-capping agents; Porównanie in vitro wytrzymałości na ścinanie wiązania płynnej żywicy kompozytowej i jednoskładnikowego szkło-jonomeru z trzema materiałami do pokrycia miazgi	2019	Dental and Medical Problems
Bulbule A.; Mandroli P.; Bhat K.; Bogar C.	In vitro evaluation of cytotoxicity of Emblica officinalis (amla) on cultured human primary dental pulp fibroblasts	2019	Journal of Indian Society of Pedodontics and Preventive Dentistry
Li W.; Mao M.; Hu N.; Wang J.; Huang J.; Gu S.	In vitro evaluation of periapical lesion-derived stem cells for dental pulp tissue engineering	2022	FEBS Open Bio
Ghahramani Y.; Ghaffaripour D.; Mohammadi N.	In vitro evaluation of the fracture resistance of biodentine pulpotomized primary molars restored with different dental materials	2019	World Journal of Dentistry
Al-Haj Ali S.N.; Al-Jundi S.H.; Ditto D.J.	In vitro toxicity of formocresol, ferric sulphate, and grey MTA on human periodontal ligament fibroblasts	2015	European Archives of Paediatric Dentistry
Al-Haj Ali S.N.; Al-Jundi S.H.; Ditto D.J.	In vitro toxicity of grey MTA in comparison to white MTA on human periodontal ligament fibroblasts	2014	European Archives of Paediatric Dentistry
Tanapitchpong R, Chunhacheevachaloke E, Ajcharanukul O.	In vivo and in vitro study of enamel fluid flow in human premolars	2020	Arch Oral Biol
Tampelini FG, Coelho MS, Rios MA, Fontana CE, Rocha DGP, Pinheiro SL, Bueno CEDS.	In vivo assessment of accuracy of Propex II, Root ZX II, and radiographic measurements for location of the major foramen	2017	Restor Dent Endod
Opal S.; Garg S.; Sharma D.; Dhindsa A.; Jatana I.	In vivo effect of calcium hydroxide and resin-modified glass ionomer cement on carious dentin in young permanent molars: An ultrastructural and macroscopic study	2017	Pediatric Dentistry
Nguyen V.; Chen Y.-W.; Johnson J.D.; Paranjpe A.	In Vivo Evaluation of Effect of Preoperative Ibuprofen on Proinflammatory Mediators in Irreversible Pulpitis Cases	2020	Journal of Endodontics
Jaya A.R.; Praveen P.; Anantharaj A.; Venkataraghavan K.; Prathibha Rani S.	In vivo evaluation of lesion sterilization and tissue repair in primary teeth pulp therapy using two antibiotic drug combinations	2012	Journal of Clinical Pediatric Dentistry
Moreira M.S.; Diniz I.M.; Rodrigues M.F.S.D.; de Carvalho R.A.; de Almeida	In vivo experimental model of orthotopic dental pulp regeneration under the influence of photobiomodulation therapy	2017	Journal of Photochemistry and Photobiology B: Biology

Carrer F.C.; Neves I.I.; Gavini G.; Marques M.M.			
Kaneko T.; Sone P.P.; Zaw S.Y.M.; Sueyama Y.; Zaw Z.C.T.; Okada Y.; Murano H.; Gu B.; Okiji T.	In vivo fate of bone marrow mesenchymal stem cells implanted into rat pulpotomized molars	2019	Stem Cell Research
Hsiao J, Wang Y, Zheng L, Liu R, Said R, Hadjiyski L, Cha H, Botero T, Chatzistavrou X, Dong Q, Papagerakis S, Papagerakis P.	In Vivo Rodent Models for Studying Dental Caries and Pulp Disease	2019	Methods Mol Biol
Ramachandran P.; PradeepKumar A.R.; Ravishankar P.; Kishen A.	In Vivo Strain Alterations in Mandibular Molars after Root Canal Treatment Procedures	2020	Journal of Endodontics
Benetti F.; Briso A.L.F.; Ferreira L.L.; Carminatti M.; Álamo L.; Ervolino E.; Dezan-Júnior E.; Cintra L.T.A.	In vivo study of the action of a topical anti-inflammatory drug in rat teeth submitted to dental bleaching	2018	Brazilian Dental Journal
Runnacles P, Arrais CA, Pochapski MT, Dos Santos FA, Coelho U, Gomes JC, De Goes MF, Gomes OM, Rueggeberg FA.	In vivo temperature rise in anesthetized human pulp during exposure to a polywave LED light curing unit	2015	Dent Mater
Del Fabbro M, Afrashtehfar KI, Corbella S, El-Kabbaney A, Perondi I, Taschieri S.	In Vivo and In Vitro Effectiveness of Rotary Nickel-Titanium vs Manual Stainless Steel Instruments for Root Canal Therapy: Systematic Review and Meta-analysis	2018	J Evid Based Dent Pract
Ramachandran P, PradeepKumar AR, Ravishankar P, Kishen A.	In Vivo Strain Alterations in Mandibular Molars after Root Canal Treatment Procedures	2020	J Endod
Kapoor K.; Grewal M.S.; Arya A.; Grewal S.; Prasad Shetty K.	Incidence of Postoperative Pain after Single Visit Root Canal Treatment using XP-endo Shaper, 2Shape and ProTaper Gold Rotary Systems: A Prospective Randomized Clinical Trial	2022	European Endodontic Journal
Wu S.; Lew H.P.; Chen N.N.	Incidence of Pulpal Complications after Diagnosis of Vital Cracked Teeth	2019	Journal of Endodontics
Maltz M, Alves LS.	Incomplete caries removal significantly reduces the risk of pulp exposure and post-operative pulpal symptoms	2013	J Evid Based Dent Pract
Camargo C.H.R.; Gomes L.C.L.; França M.C.M.; Bittencourt T.S.; Valera M.C.; Camargo S.E.A.; Bottino M.C.	Incorporating N-acetylcysteine and tricalcium phosphate into epoxy resin-based sealer improved its biocompatibility and adhesiveness to radicular dentine	2019	Dental Materials
Wang H.S.; Pei F.; Chen Z.; Zhang L.	Increased apoptosis of inflamed odontoblasts is associated with CD47 loss	2016	Journal of Dental Research
Lu M.-C.; Jheng C.-H.; Tsai T.-Y.; Koo M.; Lai N.-S.	Increased dental visits in patients prior to diagnosis of primary Sjögren’s syndrome: a population-based study in Taiwan	2014	Rheumatology International
Juan C.-Y.; Hsu C.-W.; Lu M.-C.	Increased dental visits in patients with rheumatoid arthritis: a secondary cohort analysis of population based claims data	2022	BMC Oral Health
Dong Y.; Lan W.; Wu W.; Huang Z.; Zhao J.; Peng L.; Wang J.	Increased expression of epha7 in inflamed human dental pulp	2013	Journal of Endodontics
Almeida P.N.; Barboza D.D.N.; Luna E.B.; Correia M.C.D.M.; Dias R.B.; Siquara De Sousa A.C.; Duarte M.E.L.; Rossi M.I.D.; Cunha K.S.	Increased extracellular matrix deposition during chondrogenic differentiation of dental pulp stem cells from individuals with neurofibromatosis type 1: An in vitro 2D and 3D study	2018	Orphanet Journal of Rare Diseases
Mehri-Ghahfarrokhi A.; Pourteymourfard-Tabrizi Z.; Farrokhi E.; Chaleshtori M.H.; Jami M.-S.	Increased levels of miR-124 in human dental pulp stem cells alter the expression of neural markers	2019	Journal of Otology
Chuang C.-J.; Hsu C.-W.; Lu M.-C.; Koo M.	Increased risk of developing dental diseases in patients with primary Sjögren's syndrome-A secondary cohort analysis of population-based claims data	2020	PLoS ONE
Sheth P.P., Lolayekar N.V., Hegde A.M.	Indian journal of public health research & development, may 2020, vol. 11, no. 05 341 use of biodentine(tm) for vital pulp therapy in children: Three case reports	2020	Indian Journal of Public Health Research and Development
Sebring D.; Kvist T.; Derks J.	Indications for Extraction before Implant Therapy: Focus on Endodontic Status	2019	Journal of Endodontics
Wigsten E, Jonasson P; EndoReCo; Kvist T.	Indications for root canal treatment in a Swedish county dental service: patient- and tooth-specific characteristics	2019	Int Endod J
Du W, Yang M, Kim T, Kim S, Williams DW, Esmaeili M, Hong C, Shin KH, Kang MK, Park NH, Kim RH.	Indigenous microbiota protects development of medication-related osteonecrosis induced by periapical disease in mice	2022	Int J Oral Sci
Smaïl-Faugeron V, Porot A, Muller-Bolla M, Courson F.	Indirect pulp capping versus pulpotomy for treating deep carious lesions approaching the pulp in primary teeth: a systematic review	2016	Eur J Paediatr Dent

Wu S, Liu YL, Zou J, Zhou XD, Zheng LW.	Indirect pulp therapy for deciduous teeth with deep caries lesions	2018	Hua Xi Kou Qiang Yi Xue Za Zhi
Trairatvorakul C, Sastararuji T.	Indirect pulp treatment vs antibiotic sterilization of deep caries in mandibular primary molars	2014	Int J Paediatr Dent
Angelopoulou MV, Koletsi D, Vadiakas G, Halazonetis DJ.	Induced ankylosis of a primary molar for skeletal anchorage in the mandible as alternative to mini-implants	2015	Prog Orthod
Fonzar F.; Forner L.; Fabian-Fonzar R.; Llena C.	Induced post-traumatic apexification: 20 year follow-up and morphological study after new fracture	2018	Annals of Anatomy
Bacaksiz A, Alaçam A.	Induction of maturogenesis by partial pulpotomy: 1 year follow-up	2013	Case Rep Dent
Koike T.; Polan M.A.A.; Izumikawa M.; Saito T.	Induction of reparative dentin formation on exposed dental pulp by dentin phosphophoryn/collagen composite	2014	BioMed Research International
Camoin A, Tardieu C, Dany L, Saliba-Serre B, Faulks D, Coz PL.	Inequalities in treatment planning for children with intellectual disabilities: A questionnaire study of dentists in Europe	2020	Spec Care Dentist
Kidd E, Fejerskov O, Nyvad B.	Infected Dentine Revisited	2015	Dent Update
Maisonneuve E.; Chevrier J.; Dubus M.; Varin J.; Sergheraert J.; Gangloff S.C.; Reffuveille F.; Mauprivez C.; Kerdjoudj H.	Infection of Human Dental Pulp Stromal Cells by Streptococcus mutans: Shedding Light on Bacteria Pathogenicity and Pulp Inflammation	2020	Frontiers in Cell and Developmental Biology
Cooper P.R.; Holder M.J.; Smith A.J.	Inflammation and regeneration in the dentin-pulp complex: A double-edged sword	2014	Journal of Endodontics
Leng S.; Liu L.; Xu W.; Yang F.; Du J.; Ye L.; Huang D.; Zhang L.	Inflammation down regulates stromal cell-derived factor 1α in the early phase of pulpitis	2022	Cytokine
Erdek Ö.; Bloch W.; Rink-Notzon S.; Roggendorf H.C.; Uzun S.; Meul B.; Koch M.; Neugebauer J.; Deschner J.; Korkmaz Y.	Inflammation of the Human Dental Pulp Induces Phosphorylation of eNOS at Thr495 in Blood Vessels	2022	Biomedicines
Huang X.; Liu F.; Hou J.; Chen K.	Inflammation-induced overexpression of microRNA-223-3p regulates odontoblastic differentiation of human dental pulp stem cells by targeting SMAD3	2019	International Endodontic Journal
Brizuela C.; Meza G.; Mercadé M.; Inostroza C.; Chaparro A.; Bravo I.; Briceño C.; Hernández M.; Giner L.; Ramírez V.	Inflammatory biomarkers in dentinal fluid as an approach to molecular diagnostics in pulpitis	2020	International Endodontic Journal
Esmeraldo M.R.A.; de Carvalho M.G.F.; de Carvalho R.A.; de Freitas Lima R.; de Brito Costa E.M.M.	Inflammatory effect of green propolis on dental pulp in rats	2013	Brazilian Oral Research
Galler KM, Weber M, Korkmaz Y, Widbiller M, Feuerer M.	Inflammatory Response Mechanisms of the Dentine-Pulp Complex and the Periapical Tissues	2021	Int J Mol Sci
Vaz M.M.; Lopes L.G.; Cardoso P.C.; de Souza J.B.; Batista A.C.; Costa N.L.; Torres É.M.; Estrela C.	Inflammatory response of human dental pulp to at-home and in-office tooth bleaching	2016	Journal of Applied Oral Science
Vieira-Andrade R.G.; Drumond C.L.; Alves L.P.A.; Ramos-Jorge M.A.; Marques L.S.	Inflammatory root resorption in primary molars: Prevalence and associated factors	2012	Brazilian Oral Research
Berbari R.; Nassif N.; Sfeir E.	Inflammatory Status of Excavated Pulp Tissue and Internal Root Resorption in Pulpotomized Primary Molars	2022	Journal of Dentistry (Iran)
Galvani L.D.; Costa J.L.D.S.G.; Besegato J.F.; Zaniboni J.F.; Escalante-Otárola W.G.; Kuga M.C.	Influence of agitation methods of irrigants after methylene blue-mediated PDT on the bonding interface of a fiber post cementation system	2022	Photodiagnosis and Photodynamic Therapy
Pawar R.; Alqaied A.; Safavi K.; Boyko J.; Kaufman B.	Influence of an apical negative pressure irrigation system on bacterial elimination during endodontic therapy: A prospective randomized clinical study	2012	Journal of Endodontics
Bamini L.; Anand Sherwood I.; Abbott P.V.; Uthandakalaipandian R.; Velu V.	Influence of anti-inflammatory irrigant on substance P expression for single-visit root canal treatment of teeth with irreversible pulpitis	2020	Australian Endodontic Journal
Jara CM, Hartmann RC, Böttcher DE, Souza TS, Gomes MS, Figueiredo JAP.	Influence of apical enlargement on the repair of apical periodontitis in rats	2018	Int Endod J
Zarpellon DC, Runnacles P, Maucoski C, Gross DJ, Coelho U, Rueggeberg FA, Arrais CAG.	Influence of Class V preparation on in vivo temperature rise in anesthetized human pulp during exposure to a Polywave(®) LED light curing unit	2018	Dent Mater

Vendramini V.O.; Pouraghaei S.; Barbosa R.M.; Aloise A.C.; Muniz J.R.F.; Sperandio M.; Moy P.K.; Pelegrine A.A.; Moshaverinia A.	Influence of Dental Pulp Harvesting Method on the Viability and Differentiation Capacity of Adult Dental Pulp-Derived Mesenchymal Stem Cells	2021	Stem Cells International
Evangelin J.; Sherwood I.A.; Abbott P.V.; Uthandakalaipandian R.; Velu V.	Influence of different irrigants on substance P and IL-8 expression for single visit root canal treatment in acute irreversible pulpitis	2020	Australian Endodontic Journal
de Oliveira Duque C.C.; Soares D.G.; Basso F.G.; Hebling J.; de Souza Costa C.A.	Influence of enamel/dentin thickness on the toxic and esthetic effects of experimental in-office bleaching protocols	2017	Clinical Oral Investigations
De Miranda J.L.C.; Santana C.M.M.; Santana R.B.	Influence of endodontic treatment in the post-surgical healing of human class ii furcation defects	2013	Journal of Periodontology
Brandão P.M., de Figueiredo J.A.P., Morgental R.D., Scarparo R.K., Hartmann R.C., Waltrick S.B.G., Souza R.A.	Influence of foraminal enlargement on the healing of periapical lesions in rat molars	2019	Clinical oral investigations
Simonović D.D.; Janković L.V.; Dačić S.; Petrović A.R.; Veličković S.; Petrović A.; Rakonjac M.	Influence of inflammation to lymphangiogenesis in human dental pulp; Uticaj zapaljenja na limfangiogenezu u zubnoj pulpi ljudi	2013	Acta Facultatis Medicae Naissensis
Bago I., Sandrić A., Beljic-Ivanovic K., Pažin B.	Influence of irrigation and laser assisted root canal disinfection protocols on dislocation resistance of a bioceramic sealer	2022	Photodiagnosis and Photodynamic Therapy
Prada I.; Micó-Muñoz P.; Giner-Lluesma T.; Micó-Martínez P.; Collado-Castellano N.; Manzano-Saiz A.	Influence of microbiology on endodontic failure. Literature review	2019	Medicina Oral Patologia Oral y Cirugia Bucal
R R, Aravind A, Kumar V, Sharma S, Chawla A, Logani A.	Influence of occlusal and proximal caries on the outcome of full pulpotomy in permanent mandibular molar teeth with partial irreversible pulpitis: A prospective study	2021	Int Endod J
Fontana C.E., da Silveira Bueno C.E., de Moura J.D.M., da Rocha Bastida Pinheiro G., Trevensoli V.C., Pelegrine R.A., Rocha D.G.P., De Martin A.S., Pinheiro S.L.	Influence of operator experience on apical debris extrusion after endodontic instrumentation with different single-file systems	2022	Giornale Italiano di Endodonzia
Mese M, Tok YT, Kaya S, Akcay M.	Influence of ozone application in the stepwise excavation of primary molars: a randomized clinical trial	2020	Clin Oral Investig
Abdelgawad L.M.; Nghnughi M.H.; Abdelgwad M.	Influence of photo biomodulation using 980 nm diode laser and exsosomes derived from dental pulp stem cells on pulp regeneration of dogs' teeth	2022	Journal of Medical Pharmaceutical and Allied Sciences
Banci H.A.; Strazzi-Sahyon H.B.; Duarte M.A.H.; Cintra L.T.A.; Gomes-Filho J.E.; Chalub L.O.; Berton S.A.; de Oliveira V.H.D.; dos Santos P.H.; Sivieri-Araujo G.	Influence of photodynamic therapy on bond strength and adhesive interface morphology of MTA based root canal sealer to different thirds of intraradicular dentin	2020	Photodiagnosis and Photodynamic Therapy
Santos J.M.; Marques J.A.; Diogo P.; Messias A.; Sousa V.; Sequeira D.; Palma P.J.	Influence of Preoperative Pulp Inflammation in the Outcome of Full Pulpotomy Using a Dog Model	2021	Journal of Endodontics
Ilgenstein I., Zitzmann N.U., Bühler J., Wegehaupt F.J., Attin T., Weiger R., Krastl G.	Influence of proximal box elevation on the marginal quality and fracture behavior of root-filled molars restored with CAD/CAM ceramic or composite onlays	2015	Clinical oral investigations
Angerame D.; De Biasi M.; Marigo L.; Castagnola R.; Somma F.; Castaldo A.	Influence of simulated apical resorption following orthodontic treatment on working length determination: an in vitro study.	2014	European journal of paediatric dentistry : official journal of European Academy of Paediatric Dentistry
Rosado LPL, Fagundes FB, Freitas DQ, Oliveira ML, Neves FS.	Influence of the Intracanal Material and Metal Artifact Reduction Tool in the Detection of the Second Mesiobuccal Canal in Cone-beam Computed Tomographic Examinations	2020	J Endod
Kovach I.; Buniatian K.; Makarevych A.; Verbyts'ka A.; Gargin V.	INFLUENCE OF TRICALCIUM SILICATE ON COURSE OF TRAUMATIC PULPITIS	2018	Georgian medical news
Gao B.; Chen W.; Hao L.; Zhu G.; Feng S.; Ci H.; Zhou X.; Stashenko P.; Li Y.P.	Inhibiting periapical lesions through AAV-RNAi silencing of cathepsin K	2013	Journal of Dental Research
Lv G.; Zhu G.; Xu M.; Gao X.; Xiao Q.	Inhibition of carrageenan-induced dental inflammatory responses owing to decreased TRPV1 activity by Dexmedetomidine	2020	Journal of Inflammation (United Kingdom)

Kim J.M.; Kang S.W.; Shin S.-M.; Kim D.S.; Choi K.-K.; Kim E.-C.; Kim S.-Y.	Inhibition of matrix metalloproteinases expression in human dental pulp cells by all-trans retinoic acid	2014	International Journal of Oral Science
Luo H.; Wang C.; Liu M.; Yin B.; A P.; Huang D.; Ye L.	Inhibition of SOX9 Promotes Inflammatory and Immune Responses of Dental Pulp	2018	Journal of Endodontics
Wang Y.; Yuan S.; Sun J.; Gong Y.; Liu S.; Guo R.; He W.; Kang P.; Li R.	Inhibitory effect of the TSG-6 on the BMP-4/Smad signaling pathway and odonto/osteogenic differentiation of dental pulp stem cells	2020	Biomedicine and Pharmacotherapy
Hu L.; Shen H.; Guo S.	Inhibitory Effects of Resveratrol on Inflammatory Response in Rat Dental Pulp	2022	Journal of Hard Tissue Biology
Lopes C.S.; Junqueira M.A.; Cosme-Silva L.; Pegoraro C.O.R.; Garbelini C.C.D.; Oliveira T.M.; Martins N.S.; Neves J.S.; Sakai V.T.	Initial inflammatory response after the pulpotomy of rat molars with mta or ferric sulfate	2019	Journal of Applied Oral Science
Takei E.; Shigetani Y.; Yoshiba K.; Hinata G.; Yoshiba N.; Okiji T.	Initial transient accumulation of M2 macrophage-associated molecule-expressing cells after pulpotomy with mineral trioxide aggregate in rat molars	2014	Journal of Endodontics
Silva C.R.; Babo P.S.; Gulino M.; Costa L.; Oliveira J.M.; Silva-Correia J.; Domingues R.M.A.; Reis R.L.; Gomes M.E.	Injectable and tunable hyaluronic acid hydrogels releasing chemotactic and angiogenic growth factors for endodontic regeneration	2018	Acta Biomaterialia
Pankajakshan D.; Voytik-Harbin S.L.; Nör J.E.; Bottino M.C.	Injectable Highly Tunable Oligomeric Collagen Matrices for Dental Tissue Regeneration	2020	ACS Applied Bio Materials
De Almeida Paschoalino M.; Hanan A.A.; Marques A.A.F.; Da Fonseca Roberti Garcia L.; Garrido A.B.; Sponchiado Jr. E.C.	Injection of sodium hypochlorite beyond the apical foramen-A case report	2012	General Dentistry
Zanolli C, Pan L, Dumoncel J, Kullmer O, Kundrát M, Liu W, Macchiarelli R, Mancini L, Schrenk F, Tuniz C.	Inner tooth morphology of Homo erectus from Zhoukoudian. New evidence from an old collection housed at Uppsala University, Sweden	2018	J Hum Evol
Aravinda V.S.S., Madhavi Krishna M., Nikitha B.S., Malathi Y., Jayanth C.H.	Innovative clinical technique of space maintenance using customised functional space maintainer: A case series	2021	Journal of Clinical and Diagnostic Research
Amato A., Caggiano M., Pantaleo G., Amato M.	In-office and walking bleach dental treatments on endodontically-treated teeth: 25 years follow-up	2018	Minerva Stomatologica
da SILVA-COSTA R.S.G.; Ribeiro A.E.L.; de ASSUNÇÃO I.V.; de ARAÚJO JÚNIOR R.F.; de ARAÚJO A.A.; Guerra G.C.B.; Borges B.C.D.	In-office tooth bleaching with 38% hydrogen peroxide promotes moderate/severe pulp inflammation and production of Il-1β, TNF-β, GPX, FGF-2 and osteocalcin in rats	2018	Journal of Applied Oral Science
Yoshida S.; Tomokiyo A.; Hasegawa D.; Hamano S.; Sugii H.; Maeda H.	Insight into the role of dental pulp stem cells in regenerative therapy	2020	Biology
Salama A.A.; Aboul-Ela Y.M.	INSIGHTS INTO DENTAL TREATMENT UNDER GENERAL ANESTHESIA IN A GROUP OF EGYPTIAN PEDIATRIC PATIENTS	2022	International Journal of Clinical Dentistry
Tsujino K.; Shintani S.	Intentional Partial Pulpotomy for Treatment of Immature Permanent Maxillary Incisor with Talon Cusp	2017	The Bulletin of Tokyo Dental College
Javed M.Q., Zaman H., Srivastava S., Khan Z.J.	Intentional Replantation Of Mandibular First Molar With Two Years Follow Up- Case Report	2022	Journal of Ayub Medical College, Abbottabad : JAMC
Anand P.; Mathur S.; Sachdev V.; Jain A.	Inter-comparison of antimicrobial photodynamic therapy, LASER, and an antifungal agent as adjunct intracanal irrigation techniques to standard disinfection protocols in reducing Candida albicans counts in the root canals of primary teeth: A pilot study	2020	Journal of Indian Society of Pedodontics and Preventive Dentistry
Al-Jeaidi Z.	Interdisciplinary approach for full mouth rehabilitation: A case report	2016	Asian Journal of Pharmaceutical and Clinical Research
Zafar K., Nazeer M.R., Ghafoor R.	Interdisciplinary management of gingival recession and pathologic teeth migration-Revisiting dental aesthetics	2019	JPMA. The Journal of the Pakistan Medical Association
Mathews D.P., Knight D.J., O'Connor R.V., Kokich V.G.	Interdisciplinary treatment of a patient with amelogenesis imperfecta: Case report with a 35-year follow-up	2021	Journal of Esthetic and Restorative Dentistry
Sonoda S.; Yamaza H.; Ma L.; Tanaka Y.; Tomoda E.; Aijima R.; Nonaka K.; Kukita T.; Shi S.; Nishimura F.; Yamaza T.	Interferon-gamma improves impaired dentinogenic and immunosuppressive functions of irreversible pulpitis-derived human dental pulp stem cells	2016	Scientific Reports

Šubarić L.; Mitić A.; Matvijenko V.; Jovanović R.; Živković D.; Perić D.; Vlahović Z.	Interleukin 1-beta analysis in chronically inflamed and healthy human dental pulp; Analiza interleukina 1-beta u hronično zapaljenoj i zdravoj zubnoj pulpi	2017	Vojnosanitetski Pregled
Liu M.; Zhao Y.; Wang C.; Luo H.; A P.; Ye L.	Interleukin-17 plays a role in pulp inflammation partly by WNT5A protein induction	2019	Archives of Oral Biology
Nibali L.; Fedele S.; D'Aiuto F.; Donos N.	Interleukin-6 in oral diseases: A review	2012	Oral Diseases
Tomaszewska I.M., Skinningsrud B., Jarzębska A., Pękala J.R., Tarasiuk J., Iwanaga J.	Internal and external morphology of mandibular molars: An original micro-CT study and meta-analysis with review of implications for endodontic therapy	2018	Clinical Anatomy
Schwendicke F, Walsh T, Lamont T, Al-Yaseen W, Bjørndal L, Clarkson JE, Fontana M, Gomez Rossi J, Göstemeyer G, Levey C, Müller A, Ricketts D, Robertson M, Santamaria RM, Innes NP.	Interventions for treating cavitated or dentine carious lesions	2021	Cochrane Database Syst Rev
Correa Maldonado D.; Nicoliche T.; Faber J.; Kerkis I.; Saez D.M.; Sasaki R.T.; Da Silva M.C.P.	Intra-articular human deciduous dental pulp stem cell administration vs. pharmacological therapy in experimental osteoarthritis rat model	2021	European Review for Medical and Pharmacological Sciences
Patro P.P.	Intracanal medicaments in paediatric endodontics	2020	Indian Journal of Forensic Medicine and Toxicology
Costa Y.M.; de Souza P.R.J.; Marques V.A.S.; Conti P.C.R.; Vivan R.R.; Duarte M.A.H.; Bonjardim L.R.	Intraoral Somatosensory Alterations Impact Pulp Sensibility Testing in Patients with Symptomatic Irreversible Pulpitis	2020	Journal of Endodontics
Sixou J.-L.; Marie-Cousin A.	Intraosseous anaesthesia in children with 4 % articaine and epinephrine 1:400,000 using computer-assisted systems	2015	European Archives of Paediatric Dentistry
Pourrahimi A.M.; Abbasnejad M.; Esmaeili-Mahani S.; Kooshki R.; Raoof M.	Intra-periaqueductal gray matter administration of orexin-A exaggerates pulpitis-induced anxiogenic responses and c-fos expression mainly through the interaction with orexin 1 and cannabinoid 1 receptors in rats	2019	Neuropeptides
Haeussler S, Luepke M, Seifert H, Staszyk C.	Intra-pulp temperature increase of equine cheek teeth during treatment with motorized grinding systems: influence of grinding head position and rotational speed	2014	BMC Vet Res
Bhatti U.A., Javed M.Q., Al Attas M.H.	Intra-radicular reinforcement using a modified fiber post customization technique: A case report	2021	Pan African Medical Journal
Espona J., Roig E., Durán-Sindreu F., Abella F., Machado M., Roig M.	Invasive Cervical Resorption: Clinical Management in the Anterior Zone	2018	Journal of endodontics
Singh J.; O'Donnell K.; Nieves D.J.; Adler-Shohet F.C.; Arrieta A.C.; Ashouri N.; Ahuja G.; Cheung M.; Holmes W.N.; Huoh K.; Tran L.; Tran M.T.; Pham N.; Zahn M.	Invasive mycobacterium abscessus outbreak at a pediatric dental clinic	2021	Open Forum Infectious Diseases
Gemmell A.; Stone S.; Edwards D.	Investigating acute management of irreversible pulpitis: a survey of general dental practitioners in North East England	2020	British Dental Journal
Nguyen H.-Q.-D.; Kao C.-Y.; Chiang C.-P.; Hung Y.-H.; Lo C.-M.	Investigating the Immunomodulatory Potential of Dental Pulp Stem Cell Cultured on Decellularized Bladder Hydrogel towards Macrophage Response In Vitro	2022	Gels
Cai M.; Ratnayake J.; Cathro P.; Gould M.; Ali A.	Investigation of a Novel Injectable Chitosan Oligosaccharide—Bovine Hydroxyapatite Hybrid Dental Biocomposite for the Purposes of Conservative Pulp Therapy	2022	Nanomaterials
Ustiashvili M.; Kordzaia D.; Mamaladze M.; Jangavadze M.; Sanodze L.	Investigation of functional activity human dental pulp stem cells at acute and chronic pulpitis	2014	Georgian medical news
Arruda-Vasconcelos R.; Louzada L.M.; Feres M.; Tomson P.L.; Cooper P.R.; Gomes B.P.F.A.	Investigation of microbial profile, levels of endotoxin and lipoteichoic acid in teeth with symptomatic irreversible pulpitis: a clinical study	2021	International Endodontic Journal
Chiu C.-C.; Chang Y.-C.; Huang R.-Y.; Chan J.-S.; Chung C.-H.; Chien W.-C.; Kao Y.-H.; Hsiao P.-J.	Investigation of the impact of endodontic therapy on survival among dialysis patients in taiwan: A nationwide population-based cohort study	2021	International Journal of Environmental Research and Public Health
Fahmy S.H.; Hassanien E.E.S.; Nagy M.M.; El Batouty K.M.; Mekhemar M.; Fawzy El Sayed K.; Hassanein E.H.; Wiltfang J.; Dörfer C.	Investigation of the regenerative potential of necrotic mature teeth following different revascularisation protocols	2017	Australian Endodontic Journal
Hamdan R., Michetti J., Dionnet C., Diemer F., Georgelin-Gurgel M.	In-vitro evaluation of apical microleakage of two obturation methods of immature permanent teeth: orthograde apical	2017	Giornale Italiano di Endodonzia

	plug of Mineral Trioxide Aggregate and root canal filling combining custom gutta-percha cone with Calcium Silicate-based sealer		
van Duinen RN, Shahid S, Hill R, Glavina D.	In-vitro Study on Temperature Changes in the Pulp Chamber Due to Thermo-Cure Glass Ionomer Cements	2016	Acta Stomatol Croat
Watase T, Shimizu K, Komiya H, Ohara K, Iwata K, Ogiso B.	Involvement of transient receptor potential vanilloid 1 channel expression in orofacial cutaneous hypersensitivity following tooth pulp inflammation	2018	J Oral Sci
Cassol D.V., Duarte M.L., Pintor A.V.B., Barcelos R., Primo L.G.	Iodoform Vs Calcium Hydroxide/Zinc Oxide based pastes: 12-month findings of a Randomized Controlled Trial	2019	Brazilian oral research
Kunarti S.; Nuraini N.; Astuti F.W.; Lunardi C.G.J.; Putri L.R.	Irradiation Time of Photodynamic Therapy to the Number of Lactobacillus acidophilus	2021	Archives of Orofacial Sciences
Agnihotry A.; Gill K.S.; Stevenson R.G., III; Fedorowicz Z.; Kumar V.; Sprakel J.; Cohen S.; Thompson W.	Irreversi bl e pulpitis – A source of antibiotic over-prescription?	2019	Brazilian Dental Journal
Koch J.D.; Jaramillo D.E.; DiVito E.; Peters O.A.	Irrigant flow during photon-induced photoacoustic streaming (PIPS) using Particle Image Velocimetry (PIV)	2016	Clinical Oral Investigations
Chen JE, Nurbakhsh B, Layton G, Bussmann M, Kishen A.	Irrigation dynamics associated with positive pressure, apical negative pressure and passive ultrasonic irrigations: a computational fluid dynamics analysis	2014	Aust Endod J
da Rosa W.L.O., Lima V.P., Moraes R.R., Piva E., da Silva A.F.	Is a calcium hydroxide liner necessary in the treatment of deep caries lesions? A systematic review and meta-analysis	2019	International endodontic journal
Fowler S.; Reader A.	Is a volume of 3.6 ml better than 1.8 ml for inferior alveolar nerve blocks in patients with symptomatic irreversible pulpitis?	2013	Journal of Endodontics
İnci M.A., Korkut E.	Is Bioactive Glass an Effective Agent in Pulp-capping Treatments?: A Randomized Controlled Clinical Trial with One-year Follow-up	2022	The journal of contemporary dental practice
Ganss C.	Is erosive tooth wear an oral disease?	2014	Monographs in Oral Science
Ricucci D., Loghin S., Lin L.M., Spångberg L.S., Tay F.R.	Is hard tissue formation in the dental pulp after the death of the primary odontoblasts a regenerative or a reparative process?	2014	Journal of dentistry
Assis S, Casimiro S, Alves Cardoso F.	Is it a cystic or a cyst-like condition? Discussing the etiology of an unusual large mandibular lesion in a Roman skeleton from Quinta da Torrinha/Quinta de Santo António (Almada, Portugal)	2018	Int J Paleopathol
George R.	Is partial pulpotomy in cariously exposed posterior permanent teeth a viable treatment option?	2020	Evid Based Dent
Bjørndal L.	Is pulpotomy preferable to root treatment where there is pulp exposure?	2019	Evidence-Based Dentistry
Malekfar A.; Valli K.S.; Kanafi M.M.; Bhonde R.R.	Isolation and Characterization of Human Dental Pulp Stem Cells from Cryopreserved Pulp Tissues Obtained from Teeth with Irreversible Pulpitis	2016	Journal of Endodontics
Weiss J.B.; Gonçalves F.D.S.; Maranduba C.M.D.C.; de RESENDE L.M.; Do Carmo A.M.R.	Isolation and characterization of stem cells derived by human dental pulp from harvest based in rotary and manual techniques used in endodontic therapy; Isolamento e caracterização de células tronco da polpa dentária humana obtidas em instrumentação mecanizada e manual durante tratamento endodôntico	2020	Brazilian Dental Science
Horibe H.; Murakami M.; Iohara K.; Hayashi Y.; Takeuchi N.; Takei Y.; Kurita K.; Nakashima M.	Isolation of a stable subpopulation of Mobilized Dental Pulp Stem Cells (MDPSCs) with high proliferation, migration, and regeneration potential is independent of age	2014	PLoS ONE
Tsai A.I.; Hong H.-H.; Lin W.-R.; Fu J.-F.; Chang C.-C.; Wang I.-K.; Huang W.-H.; Weng C.-H.; Hsu C.-W.; Yen T.-H.	Isolation of Mesenchymal Stem Cells from Human Deciduous Teeth Pulp	2017	BioMed Research International
Zhang K.; Yang W.	Iterative Noise Reduction Algorithm-Based Cone Beam Computed Tomography Image Analysis for Dental Pulp Disease in Root Canal Therapies	2022	Scientific Programming
Santhosh B.P.; Jethmalani P.	Johanson-Blizzard syndrome: Dental findings and management	2013	Journal of Contemporary Dental Practice
Hayama T.; Kamio N.; Okabe T.; Muromachi K.; Matsushima K.	Kallikrein Promotes Inflammation in Human Dental Pulp Cells Via Protease-Activated Receptor-1	2016	Journal of Cellular Biochemistry
Jeevanandan G.	Kedo-S paediatric rotary files for root canal preparation in primary teeth - Case report	2017	Journal of Clinical and Diagnostic Research

Choi E.-K.; Kim S.-H.; Kang I.-C.; Jeong J.-Y.; Koh J.-T.; Lee B.-N.; Oh W.-M.; Min K.-S.; Nör J.E.; Hwang Y.-C.	Ketoprofen inhibits expression of inflammatory mediators in human dental pulp cells	2013	Journal of Endodontics
Pelagalli P.; Gatto R.; Moscati M.	Kids Digital Crown Technique: an innovative approach to restore primary teeth	2021	European Journal of Paediatric Dentistry
Sun Z, Wang L, Peng B.	Kinetics of glycogen synthase kinase (GSK)3β and phosphorylated GSK3β (Ser 9) expression in experimentally induced periapical lesions	2014	Int Endod J
AlJazairy Y.H., Halawany H.S., AlMaflehi N., Alhussainan N.S., Abraham N.B., Jacob V.	Knowledge about permanent tooth avulsion and its management among dentists in Riyadh, Saudi Arabia	2015	BMC oral health
Santhosh Kumar M.P., Lavanya	Knowledge about post extraction complications among undergraduate dental students	2016	Journal of Pharmaceutical Sciences and Research
Acharya S.	Knowledge and attitude of general and specialist dentist in pediatric dentistry: A pilot study in Odisha, India	2019	Indian Journal of Dental Research
Karthikeson P.S.; Vignesh R.	Knowledge and attitude of general dentists and dentists of other specialties toward endodontic treatment of primary teeth	2019	Drug Invention Today
Bharadwaj B.; Jeevanandan G.	Knowledge and awareness about formocresol used in pulpotomy among dental students	2018	Drug Invention Today
Alex A., Balaji Ganesh S., Prathap L.	Knowledge and awareness of various dental biomaterials used in pediatric patients-a survey	2020	Indian Journal of Forensic Medicine and Toxicology
Mary D.J.; Anjaneyulu K.; Santhosh Kumar M.P.	Knowledge and awareness regarding root canal treatment among the general population	2021	International Journal of Dentistry and Oral Science
Gasqui M.-A., Laforest L., Le Clerc J., Ceinos R., Chemla F., Chevalier V., Colon P., Fioretti F., Gevrey A., Kérourédan O., Maret D., Mocquot C., Ozcan C., Perez F., Terrer E., Turpin Y.-L., Arbab-Chirani R., Doméjean S., Seux D.	Knowledge and Opinions of French Dental Students in Operative Dentistry - Management of Deep Carious Lesions	2021	Oral health & preventive dentistry
Nayak U.A., Wadhwa S., Kashyap N., Prajapati D., Mahuli A.V., Sharma R.	Knowledge and practice of, and attitudes toward, pulp therapy in deciduous teeth among pediatric dentists in India	2018	Journal of investigative and clinical dentistry
Sheriff A.H., Ganapathy D., Rohinikumar S.	Knowledge and practice towards management of space infections among dental practitioners	2020	International Journal of Research in Pharmaceutical Sciences
Juliet S.; Gurunathan D.	Knowledge attitude and practice of general dentist towards pediatric dentistry	2018	Biomedicine (India)
Kumaran A.S.K.; Ramesh S.	KNOWLEDGE AWARENESS AND PERCEPTION OF PAIN MANAGEMENT POSTOPERATIVELY AFTER ROOT CANAL TREATMENT USING HAND AND ROTARY INSTRUMENTATION	2022	Journal of Pharmaceutical Negative Results
Al-Haj Ali S.N.; Algarawi S.A.; Alrubaian A.M.; Alasqah A.I.	Knowledge of General Dental Practitioners and Specialists about Emergency Management of Traumatic Dental Injuries in Qassim, Saudi Arabia	2020	International Journal of Pediatrics (United Kingdom)
Hemmanur S., Nasim I., Sowmya K.	Knowledge, attitude and practice of endodontic emergencies and their management amongst general dental practitioners and endodontists - A questionnaire based survey	2020	International Journal of Pharmaceutical Research
Sudarsan R.; Balaji Ganesh S.; Anjaneyulu K.	Knowledge, attitude and practise of usage of mta and bio dentine as direct pulp capping agents-a questionnaire study	2020	International Journal of Research in Pharmaceutical Sciences
Jain A., Mani G.	Knowledge, attitude, and practice regarding standardized treatment protocol for pulp therapy in deciduous dentition among general dental practitioners of Chennai, India-A questionnaire survey	2020	International Journal of Pharmaceutical Research
Bargale S, Davangere Padmanabh SK, Kariya PB, Shah S, Dave B.	Knowledge, attitude, and practice regarding standardized treatment protocol for pulp therapy in deciduous dentition among general dental practitioners of Vadodara, Gujarat, India	2019	J Indian Soc Pedod Prev Dent
Keerthika R.; Ranjan M.	Laser as an antimicrobial photodynamic therapy in endodontics-literature review	2021	International Journal of Dentistry and Oral Science

Lee H.-N.; Yan D.-Y.; Huang C.-Y.; Chen S.-C.; Pan C.-Y.; Jeng J.-H.; Chen Y.-K.; Chuang F.-H.	Laser doppler for accurate diagnosis of oehler's type iii dens invaginatus: A case report	2021	Applied Sciences (Switzerland)
Pereira L.O.; Longo J.P.F.; Azevedo R.B.	Laser irradiation did not increase the proliferation or the differentiation of stem cells from normal and inflamed dental pulp	2012	Archives of Oral Biology
Abalos C, Herrera M, Bonilla V, San Martin L, Mendoza A.	Laser-induced fluorescence in the diagnosis of pulp exposure and the influence of residual dentin thickness: An in vivo study	2015	Am J Dent
Pagano S.; Lombardo G.; Orso M.; Abraha I.; Capobianco B.; Cianetti S.	Lasers to prevent dental caries: A systematic review	2020	BMJ Open
Kataoka S.H.H.; Setzer F.C.; Gondim-Junior E.; Fregnani E.R.; Moraes C.J.P.; Pessoa O.F.; Gavini G.; Caldeira C.L.	Late Effects of Head and Neck Radiotherapy on Pulp Vitality Assessed by Pulse Oximetry	2016	Journal of Endodontics
Sukkul P.; Kasemsap N.	Lemierre's syndrome with cavernous sinus thrombosis caused by dental infection	2021	BMJ Case Reports
Martín-González J.; Sánchez-Jiménez F.; Pérez-Pérez A.; Carmona-Fernández A.; Sánchez-Margalet V.; Segura-Egea J.J.	Leptin expression in healthy and inflamed human dental pulp	2013	International Endodontic Journal
Ngo V.A.; Jung J.-Y.; Koh J.-T.; Oh W.-M.; Hwang Y.-C.; Lee B.-N.	Leptin Induces Odontogenic Differentiation and Angiogenesis in Human Dental Pulp Cells via Activation of the Mitogen-activated Protein Kinase Signaling Pathway	2018	Journal of Endodontics
Martín-González J.; Pérez-Pérez A.; Sánchez-Jiménez F.; Carmona-Fernández A.; Torres-Lagares D.; Sánchez-Margalet V.; Segura-Egea J.J.	Leptin receptor is up-regulated in inflamed human dental pulp	2013	Journal of Endodontics
Martín-González J.; Pérez-Pérez A.; Cabanillas-Balsera D.; Vilariño-García T.; Sánchez-Margalet V.; Segura-Egea J.J.	Leptin stimulates DMP-1 and DSPP expression in human dental pulp via MAPK 1/3 and PI3K signaling pathways	2019	Archives of Oral Biology
San Diego JP, Newton T, Quinn BF, Cox MJ, Woolford MJ.	Levels of agreement between student and staff assessments of clinical skills in performing cavity preparation in artificial teeth	2014	Eur J Dent Educ
Aguirre-López E.C.; Patiño-Marín N.; Martínez-Castañón G.A.; Medina-Solís C.E.; Castillo-Silva B.E.; Cepeda-Argüelles O.; Aguilera-Galaviz L.A.; Rosales-García P.	Levels of matrix metalloproteinase-8 and cold test in reversible and irreversible pulpitis	2020	Medicine (United States)
Zhang J.; Si J.; Liang R.; Lu Y.; Shang H.; Li X.; Sun S.; Wu L.-A.	Ligand-gated ion channel P2X7 regulates hypoxia-induced factor-1α mediated pain induced by dental pulpitis in the medullary dorsal horn	2022	Frontiers in Molecular Neuroscience
García-Mota LF, Hardan L, Bourgi R, Zamarripa-Calderón JE, Rivera-Gonzaga JA, Hernández-Cabanillas JC, Cuevas-Suárez CE.	LIGHT-CURED CALCIUM SILICATE BASED-CEMENTS AS PULP THERAPEUTIC AGENTS: A META-ANALYSIS OF CLINICAL STUDIES	2022	J Evid Based Dent Pract
Fontana M.	Limited evidence for main reason for failure of partially excavated and restored teeth	2014	Evid Based Dent
Liu Y.; Dong N.; Miao J.; Li C.; Wang X.; Ruan J.	Lin28 promotes dental pulp cell proliferation via upregulation of cyclin-dependent proteins and interaction with let-7a/IGF2BP2 pathways	2019	Biomedicine and Pharmacotherapy
Huang Y.; Jiang H.; Gong Q.; Li X.; Ling J.	Lipopolysaccharide stimulation improves the odontoblastic differentiation of human dental pulp cells	2015	Molecular Medicine Reports
Carrouel F.; Staquet M.-J.; Keller J.-F.; Baudouin C.; Msika P.; Bleicher F.; Alliot-Licht B.; Farges J.-C.	Lipopolysaccharide-binding protein inhibits toll-like receptor 2 activation by lipoteichoic acid in human odontoblast-like cells	2013	Journal of Endodontics
Qiao W.; Huang Y.; Bian Z.; Sun X.; Wang X.; Gao Q.; Peng Y.; Meng L.	Lipopolysaccharide-induced DNA damage response activates nuclear factor κB signalling pathway via GATA4 in dental pulp cells	2019	International Endodontic Journal
Ali M.; Okamoto M.; Komichi S.; Watanabe M.; Huang H.; Takahashi Y.; Hayashi M.	Lithium-containing surface pre-reacted glass fillers enhance hDPSC functions and induce reparative dentin formation in a rat pulp capping model through activation of Wnt/β-catenin signaling	2019	Acta Biomaterialia
Parhizkar A, Asgary S.	Local Drug Delivery Systems for Vital Pulp Therapy: A New Hope	2021	Int J Biomater

Zhuk R, Taylor S, Johnson JD, Paranjpe A.	Locating the MB2 canal in relation to MB1 in Maxillary First Molars using CBCT imaging	2020	Aust Endod J
Krapež J, Fidler A.	Location and dimensions of access cavity in permanent incisors, canines, and premolars	2013	J Conserv Dent
Sun S.Yu., Wang H.	Location, incidence and clinical implications of the root canal isthmus	2021	Journal of Prevention and Treatment for Stomatological Diseases
Danagoulian S., Wilk T.A.	Locking out prevention: Dental care in the midst of a pandemic	2022	Health Economics (United Kingdom)
Liu M.; Chen L.; Wu J.; Lin Z.; Huang S.	Long noncoding RNA MEG3 expressed in human dental pulp regulates LPS-Induced inflammation and odontogenic differentiation in pulpitis	2021	Experimental Cell Research
Xia L.; Wang J.; Qi Y.; Fei Y.; Wang D.	Long Non-coding RNA PVT1 is Involved in the Pathological Mechanism of Pulpitis by Regulating miR-128-3p	2022	Oral Health and Preventive Dentistry
Casagrande L, Seminario AT, Correa MB, Werle SB, Maltz M, Demarco FF, Araujo FB.	Longevity and associated risk factors in adhesive restorations of young permanent teeth after complete and selective caries removal: a retrospective study	2017	Clin Oral Investig
Chaipattanawan N, Chompu-Inwai P, Nirunsittirat A, Phinyo P, Manmontri C.	Longevity of stainless steel crowns as interim restorations on young permanent first molars that have undergone vital pulp therapy treatment in children and factors associated with their treatment failure: A retrospective study of up to 8.5 years	2022	Int J Paediatr Dent
Rawson T.H., Rayes S., Strizich G., Salazar C.R.	Longitudinal Study Comparing Pulpectomy and Pulpotomy Treatments for Primary Molars of Alaska Native Children	2019	Pediatric dentistry
Morita A.; Takahashi H.; Ozawa K.; Imafuku S.; Takekuni N.; Takahashi K.; Matsuyama T.; Okubo Y.; Zhao Y.; Kitamura S.; Takei K.; Yokoyama M.; Hayashi N.; Terui T.	Long-term analysis of adalimumab in Japanese patients with moderate to severe hidradenitis suppurativa: Open-label phase 3 results	2021	Journal of Dermatology
Chaniotis A.; Chanioti A.	Long-term Complications of Previously Successful Regenerative Endodontic Procedures after Orthodontic Movement: A Report of 3 Different Complications after 4, 8, and 11 Years	2022	Journal of Endodontics
Schwendicke F.; Basso M.; Markovic D.; Turkun L.S.; Miletić I.	Long-term cost-effectiveness of glass hybrid versus composite in permanent molars	2021	Journal of Dentistry
Meng H.; Wei F.; Ge Z.; Jin J.; Wang H.; Wang L.-S.; Wu C.-T.	Long-term hypoxia inhibits the passage-dependent stemness decrease and senescence increase of human dental pulp stem cells	2022	Tissue and Cell
Tan S.Y., Yu V.S.H., Lim K.C., Tan B.C.K., Neo C.L.J., Shen L., Messer H.H.	Long-term Pulpal and Restorative Outcomes of Pulpotomy in Mature Permanent Teeth	2020	Journal of endodontics
Hoefler V, Nagaoka H, Miller CS.	Long-term survival and vitality outcomes of permanent teeth following deep caries treatment with step-wise and partial-caries-removal: A Systematic Review	2016	J Dent
Yuan H.; Suzuki S.; Terui H.; Hirata-Tsuchiya S.; Nemoto E.; Yamasaki K.; Saito M.; Shiba H.; Aiba S.; Yamada S.	Loss of IκBζ Drives Dentin Formation via Altered H3K4me3 Status	2022	Journal of Dental Research
Zhong T.-Y.; Zhang Z.-C.; Gao Y.-N.; Lu Z.; Qiao H.; Zhou H.; Liu Y.	Loss of Wnt4 expression inhibits the odontogenic potential of dental pulp stem cells through JNK signaling in pulpitis	2019	American Journal of Translational Research
Gränicher K.A.; Karygianni L.; Attin T.; Thurnheer T.	Low Concentrations of Chlorhexidine Inhibit the Formation and Structural Integrity of Enzyme-Treated Multispecies Oral Biofilms	2021	Frontiers in Microbiology
Marques N.C.T.; Neto N.L.; Rodini C.O.; Fernandes A.P.; Sakai V.T.; Machado M.A.A.M.; Oliveira T.M.	Low-level laser therapy as an alternative for pulpotomy in human primary teeth	2015	Lasers in Medical Science
Huang Y.; Li X.; Liu Y.; Gong Q.; Tian J.; Jiang H.	LPS-induced autophagy in human dental pulp cells is associated with p38	2021	Journal of Molecular Histology
Chen Y.; Zhang L.; Yang J.; Zhang L.; Chen Z.	LPS-induced dental pulp inflammation increases expression of ionotropic purinergic receptors in rat trigeminal ganglion	2014	NeuroReport
Moura J.; Lima M.; Nogueira N.; Castro M.; Lima C.; Moura M.; Moura L.	LSTR Antibiotic Paste Versus Zinc Oxide and Eugenol Pulpectomy for the Treatment of Primary Molars with Pulp Necrosis: A Randomized Controlled Trial	2021	Pediatric dentistry
Pan H.; Cheng L.; Yang H.; Zou W.; Cheng R.; Hu T.	Lysophosphatidic acid rescues human dental pulp cells from ischemia-induced apoptosis	2014	Journal of Endodontics

Watanabe T.; Kamio N.; Okabe T.; Hayama T.; Fukai J.; Watanabe A.; Okada H.; Matsushima K.	Macrophage migration inhibitory factor promotes inflammation in human dental pulp	2020	Journal of Hard Tissue Biology
Gerlach K.; Ludewig E.; Brehm W.; Gerhards H.; Delling U.	Magnetic resonance imaging of pulp in normal and diseased equine cheek teeth	2013	Veterinary Radiology and Ultrasound
Emara R, Krois J, Schwendicke F.	Maintaining pulpal vitality: Cost-effectiveness analysis on carious tissue removal and direct pulp capping	2020	J Dent
Abazarian N.; Milani S.; Hamrah M.H.; Shahrabi M.S.	Management and Follow-Up of Complicated Crown Fractures with Intrusive Luxation of Maxillary Incisors in an 8-Year-Old Boy	2021	Case Reports in Dentistry
Nayak G., Singh I.	Management of a maxillary second molar	2013	Dentistry today
Anilkumar K, Lingeswaran S, Ari G, Thyagarajan R, Logaranjani A.	Management of Chronic Hyperplastic Pulpitis in Mandibular Molars of Middle Aged Adults- A Multidisciplinary Approach	2016	J Clin Diagn Res
Shafuria A.; Dwiarie T.A.; Hidayat W.	Management of Chronic Ulcer Mimicking Oral Squamous Cell Carcinoma in Down Syndrome Child with Motoric Disorders	2022	International Medical Case Reports Journal
Verma P.K.; Srivastava R.; Gupta K.K.; Srivastava A.	Management of combined endodontic - periodontal lesion: Case reports	2012	Medico-Legal Update
Asgary S.; Fazlyab M.	Management of complicated crown fracture with miniature pulpotomy: A case report	2014	Iranian Endodontic Journal
Bjørndal L, Simon S, Tomson PL, Duncan HF.	Management of deep caries and the exposed pulp	2019	Int Endod J
Tedesco T.K., Reis T.M., Mello-Moura A.C.V., Silva G.S.D., Scarpini S., Floriano I., Gimenez T., Mendes F.M., Raggio D.P.	Management of deep caries lesions with or without pulp involvement in primary teeth: a systematic review and network meta-analysis	2020	Brazilian oral research
Rathi S., Nikhade P., Jaiswal A., Jaiswal A., Chandak M., Rathi C.	Management of deep carious lesion with single visit indirect pulp capping: A case report	2020	Indian Journal of Forensic Medicine and Toxicology
Croft K, Kervanto-Seppälä S, Stangvaltaite L, Kerosuo E.	Management of deep carious lesions and pulps exposed during carious tissue removal in adults: a questionnaire study among dentists in Finland	2019	Clin Oral Investig
Pozos-Guillén A, Molina G, Soviero V, Arthur RA, Chavarria-Bolaños D, Acevedo AM.	Management of dental caries lesions in Latin American and Caribbean countries	2021	Braz Oral Res
Timmerman A., Parashos P.	Management of dental pain in primary care	2020	Australian Prescriber
Singh S., Gupta K., Dhull K.S., Ashika B.K., Kudagi V.S., Gupta A.	MANAGEMENT OF EARLY CHILDHOOD CARIES IN A 5-YEAR-OLD CHILD USING THE DISTINCTIVE OMEGA POSTS - A CASE REPORT	2022	Journal of Pharmaceutical Negative Results
Mehra M., Grover R., Pandit I.K., Srivastava N., Gugnani N., Gupta M.	Management of grossly decayed primary anteriors using various intracanal post systems: A clinical study	2016	Journal of the Indian Society of Pedodontics and Preventive Dentistry
Tzanetakis G.N.	Management of Intruded Immature Maxillary Central Incisor with Pulp Necrosis and Severe External Resorption by Regenerative Approach	2018	Journal of Endodontics
Deepak S.; Anjaneyulu K.; Nivedhitha M.S.	MANAGEMENT OF LARGE PERIAPICAL LESION USING PRF MIXED WITH BONE GRAFT – A CASE REPORTS	2021	International Journal of Clinical Dentistry
Kishan K.V., Das D., Chhabra N., Rathore V.P.S., Remy V.	Management of maxillary first molar with six canals using operating microscope	2018	Indian journal of dental research : official publication of Indian Society for Dental Research
Karunakaran J.V.; Shobana R.; Kumar M.; Kumar S.; Mankar S.	Management of middle mesial canal in mandibular second molar	2012	Journal of Pharmacy and Bioallied Sciences
Srinivasan R.; Ravishanker P.	Management of middle mesial canal under dental operating microscope	2015	Medical Journal Armed Forces India
Robles Raya P.; Javierre Miranda A.P.; Moreno Millán N.; Mas Casals A.; de Frutos Echániz E.; Morató Agustí M.L.	Management of odontogenic infections in Primary Care: Antibiotic?; Manejo de las infecciones odontogénicas en las consultas de atención primaria: ¿antibiótico?	2017	Atencion Primaria
Sierra-Lorenzo A.; Herrera-García A.; Alonso-Ezpeleta L.O.; Segura-Egea J.J.	Management of perforating internal root resorption with periodontal surgery and mineral trioxide aggregate: A case report with 5-year follow-up	2013	International Journal of Periodontics and Restorative Dentistry

Stangvaltaite L, Schwendicke F, Holmgren C, Finet M, Maltz M, Elhennawy K, Kerosuo E, Doméjean S.	Management of pulps exposed during carious tissue removal in adults: a multi-national questionnaire-based survey	2017	Clin Oral Investig
Tonini R., Boschi G., Salgarello S.A.	Management of seven external cervical resorptions	2020	Giornale Italiano di Endodonzia
Motwani N., Ikhar A., Nikhade P., Bhonde R., Jaiswal S., Zamare V.	Management of single rooted mandibular second molar with single canal: Two case reports	2020	Indian Journal of Forensic Medicine and Toxicology
Joseph E.J., Anupama Nayak P., Rao A.	Management of talons cusp in a primary maxillary central incisor: A rare case report	2019	Indian Journal of Public Health Research and Development
Bettie N., Kandasamy S., Prasad V.	Management of tooth surface loss of varying etiology with full mouth all ceramic computer-aided design/computer-aided manufacture restorations	2017	Journal of Pharmacy and Bioallied Sciences
Thomas J.; Arumugam E.; Harris A.; Ravi V.	Management of traumatized immature vital tooth: A case report	2019	Journal of Pharmacy and Bioallied Sciences
Kamareh S., Kazem M., Foroozandeh M., Gohari A.	Management of two taurodont primary molars with pulp involvement using calcium-enriched mixture cement pulpotomy in a patient with accompanied drug reaction with eosinophilia and systemic symptoms syndrome: A case report	2019	Biomedical Research and Therapy
Palomino-Delgado M., Coaguila-Llerena H., Mendiola-Aquino C., Faria G.	Management of unfavorable iatrogenic perforations during post placement using mineral trioxide aggregate: Report of two cases	2021	Revista Estomatologica Herediana
Alnahwi TH, Alhamad M, Majeed A, Nazir MA.	Management preferences of deep caries in permanent teeth among dentists in Saudi Arabia	2018	Eur J Dent
Chee Koh S.W., Li C.F., Pheng Loh J.S., Wong M.L., Keong Loh V.W.	Managing tooth pain in general practice	2019	Singapore Medical Journal
Gonçalves N., Pereira B., Meirinhos J., Pires M.D., Vasconcelos I., Martins J.N.R., Ginjeira A.	Mandibular first molar root canal retreatment with the presence of a missed middle mesial root canal: A report of two cases	2021	Giornale Italiano di Endodonzia
Wu D., Zhang J., Wang W., Xin B.-C.	Mandibular first molar with C-shaped root canal system: a two-case report	2018	Hua xi kou qiang yi xue za zhi = Huaxi kouqiang yixue zazhi = West China journal of stomatology
Gaur A.; Trivedi H.P.; Gupta M.; Sharma A.; Likhyani L.; Agarwal M.	Mandibular first molar with vertucci type I canal configuration diagnosed with the help of cone beam computed tomography: A rare case report	2015	Journal of Contemporary Dental Practice
Zhang M.; Xie J.; Wang Y.-H.; Feng Y.	Mandibular first premolar with five root canals: A case report	2020	BMC Oral Health
Du Y.; Lee A.H.; Zhang C.	Mandibular first premolar with four canals.	2013	Journal of investigative and clinical dentistry
Kakkar P, Singh A.	Mandibular first premolar with three roots: a case report	2012	Iran Endod J
Kupczik K, Delezene LK, Skinner MM.	Mandibular molar root and pulp cavity morphology in Homo naledi and other Plio-Pleistocene hominins	2019	J Hum Evol
Thompson P.W.; Williams J.K.	Mandibular Osteomyelitis and Cervical Lymphadenitis Due to Mycobacterium abscessus: Surgical Management of a Pediatric Cohort With a Shared Epidemiologic Exposure	2017	The Journal of craniofacial surgery
Roy A.; Velmurugan N.; Suresh N.	Mandibular second molar with a single root and a single canal: Case series	2013	Journal of Clinical and Diagnostic Research
Niavarzi S, Ghabraei S, Malekpour F.	Mandibular Second Premolar with Four Canals: A Case Report	2022	Iran Endod J
Chen Y, Chen X, Zhang Y, Zhou F, Deng J, Zou J, Wang Y.	Materials for pulpotomy in immature permanent teeth: a systematic review and meta-analysis	2019	BMC Oral Health
Santos P.S.D., Pedrotti D., Braga M.M., Rocha R.O., Lenzi T.L.	Materials used for indirect pulp treatment in primary teeth: a mixed treatment comparisons meta-analysis	2017	Brazilian oral research
Nugroho J.J.; Sumidarti A.; Siri M.; Cangara M.H.; Natsir N.; Tanumihardja M.; Hikmah N.; Asrianti	Matrix metalloproteinase-1 (MMP-1) expression and density of collagen fibers following application of haruan fish (channa striata) extract in inflamed pulp of wistar rat	2020	Systematic Reviews in Pharmacy
Sambandam V, Neelakantan P.	Matrix metalloproteinases (mmp) in restorative dentistry and endodontics	2014	J Clin Pediatr Dent
Atabek D, Sillelioğlu H, Çinar Ç, Ölmez A.	Maturogenesis of an Early Erupted Immature Permanent Tooth: A Case Report With 7-Year Follow-Up	2015	J Clin Pediatr Dent
Sibal A.J.; Singi S.R.	Maxillary 1st molar with three canals in mesiobuccal root	2022	Pan African Medical Journal

Rodrigues E, Braitt AH, Galvão BF, da Silva EJ.	Maxillary first molar with 7 root canals diagnosed using cone-beam computed tomography	2017	Restor Dent Endod
Shi JJ.	Maxillary first molar with two palatal canals: a case report	2013	Shanghai Kou Qiang Yi Xue
Rahimi S, Ghasemi N.	Maxillary first molar with two root canals	2013	Sultan Qaboos Univ Med J
Lea C.; Deblinger J.; Machado R.; Nogueira Leal Silva E.J.; Vansan L.P.	Maxillary premolar with 4 separate canals	2014	Journal of Endodontics
Suresh M., Karthikeyan K., Mahalaxmi S.	Maxillary second molar with fused root and six canals- a case report.	2017	Journal of Clinical and Diagnostic Research
Kalyani Behera A., Nasim I.	Maxillary sinusitis of endodontic origin – decision analysis	2020	International Journal of Pharmaceutical Research
Wyss F, Müller J, Clauss M, Kircher P, Geyer H, von Rechenberg B, Hatt JM.	Measuring Rabbit (Oryctolagus cuniculus) Tooth Growth and Eruption by Fluorescence Markers and Bur Marks	2016	J Vet Dent
Dos Santos Neto A.P.; Maia S.M.A.S.; Leão J.C.; Quidute I.L.; Guimarães C.S.; Alves Júnior S.; Álvares P.R.; Ribeiro M.I.G.; Silva L.B.	Mechanisms involved in apice closure of pulpless teeth – literature review	2021	Open Dentistry Journal
Petrukhina NB, Zorina OA, Venediktova VA.	Mechanisms of age-related changes in the morphology of the pulp system of the first lower molars	2022	Stomatologiia (Mosk)
Kantrong N.; Jit-Armart P.; Arayatrakoollikit U.	Melatonin antagonizes lipopolysaccharide-induced pulpal fibroblast responses	2020	BMC Oral Health
Guerrero-gironés J.; Alcaina-lorente A.; Ortiz-ruiz C.; Ortiz-Ruiz E.; Pecci-lloret M.P.; Rodríguez-lozano F.J.; Martínez C.M.; Ortiz-ruiz A.J.	Melatonin as an agent for direct pulp-capping treatment	2020	International Journal of Environmental Research and Public Health
Li J.-G.; Lin J.-J.; Wang Z.-L.; Cai W.-K.; Wang P.-N.; Jia Q.; Zhang A.-S.; Wu G.-Y.; Zhu G.-X.; Ni L.-X.	Melatonin attenuates inflammation of acute pulpitis subjected to dental pulp injury	2015	American Journal of Translational Research
Inostroza C.; Vega-Letter A.M.; Brizuela C.; Castrillón L.; Saint Jean N.; Duran C.M.; Carrión F.	Mesenchymal Stem Cells Derived from Human Inflamed Dental Pulp Exhibit Impaired Immunomodulatory Capacity In Vitro	2020	Journal of Endodontics
Lim K.-T.; Patil T.V.; Patel D.K.; Dutta S.D.; Ganguly K.; Randhawa A.	Mesenchymal stem cells, the secretome and biomaterials: Regenerative medicine application	2022	Biocell
Corica A, Caprioglio A.	Meta-analysis of the prevalence of tooth wear in primary dentition	2014	Eur J Paediatr Dent
Brodzikowska A.; Gondek A.; Rak B.; Paskal W.; Pełka K.; Cudnoch-Jędrzejewska A.; Włodarski P.	Metalloproteinase 14 (MMP-14) and hsa-miR-410-3p expression in human inflamed dental pulp and odontoblasts	2019	Histochemistry and Cell Biology
Cardoso F.P.; De Faria Amormino S.A.; Dutra W.O.; Ribeiro Sobrinho A.P.; Moreira P.R.	Methylation pattern of the CD14 and TLR2 genes in human dental pulp	2014	Journal of Endodontics
Luo H.; Liu W.; Zhang Y.; Yang Y.; Jiang X.; Wu S.; Shao L.	METTL3-mediated m6A modification regulates cell cycle progression of dental pulp stem cells	2021	Stem Cell Research and Therapy
Gatta V.; Zizzari V.L.; Dd ' Amico V.; Salini L.; D' Aurora M.; Franchi S.; Antonucci I.; Sberna M.T.; Gherlone E.; Stuppia L.; Tetè S.	Microarray evaluation of gene expression profiles in inflamed and healthy human dental pulp: the role of IL1beta and CD40 in pulp inflammation	2012	Journal of biological regulators and homeostatic agents
Pereira R.S.; Rodrigues V.A.A.; Furtado W.T.; Gueiros S.; Pereira G.S.; Avila-Campos M.J.	Microbial analysis of root canal and periradicular lesion associated to teeth with endodontic failure	2017	Anaerobe
Moraes LC, Lang PM, Arcanjo RA, Rampelotto PH, Fatturi-Parolo CC, Ferreira MBC, Montagner F.	Microbial ecology and predicted metabolic pathways in various oral environments from patients with acute endodontic infections	2020	Int Endod J
Fouad A.F.	Microbial Factors and Antimicrobial Strategies in Dental Pulp Regeneration	2017	Journal of Endodontics
Tekbas Atay M, Koray F.	Microbiological and SEM assessment of atraumatic restorative treatment in adult dentition	2021	Clin Oral Investig
Subramaniam P.; Tabrez T.A.; Girish Babu K.L.	Microbiological assessment of root canals following use of rotary and manual instruments in primary molars	2013	Journal of Clinical Pediatric Dentistry
Barbosa-Ribeiro M.; Arruda-Vasconcelos R.; Louzada L.M.; Lima A.R.; Marciano M.A.; Almeida J.F.A.;	Microbiological investigation in teeth with persistent/secondary endodontic infection in different stages of root canal retreatment	2020	European Endodontic Journal

Dejesus-Soares A.; Zaia A.A.; Ferraz C.C.R.; Gomes B.P.F.A.			
Hernández M.; Planells P.; Martínez E.; Mira A.; Carda-Diéguez M.	Microbiology of molar–incisor hypomineralization lesions. A pilot study	2020	Journal of Oral Microbiology
Fouad A.F.; Diogenes A.R.; Torabinejad M.; Hargreaves K.M.	Microbiome Changes during Regenerative Endodontic Treatment Using Different Methods of Disinfection	2022	Journal of Endodontics
Zheng J, Wu Z, Niu K, Xie Y, Hu X, Fu J, Tian D, Fu K, Zhao B, Kong W, Sun C, Wu L.	Microbiome of Deep Dentinal Caries from Reversible Pulpitis to Irreversible Pulpitis	2019	J Endod
Rôças I.N.; Alves F.R.F.; Rachid C.T.C.C.; Lima K.C.; Assunção I.V.; Gomes P.N.; Siqueira J.F., Jr.	Microbiome of Deep Dentinal Caries Lesions in Teeth with Symptomatic Irreversible Pulpitis	2016	PLoS ONE
Aydın Z.U., Keskin N.B., Özyürek T., Geneci F., Ocak M., Çelik H.H.	Microcomputed Assessment of Transportation, Centering Ratio, Canal Area, and Volume Increase after Single-file Rotary and Reciprocating Glide Path Instrumentation in Curved Root Canals: A Laboratory Study	2019	Journal of endodontics
Filpo-Perez C., Bramante C.M., Villas-Boas M.H., Húngaro Duarte M.A., Versiani M.A., Ordinola-Zapata R.	Micro-computed tomographic analysis of the root canal morphology of the distal root of mandibular first molar	2015	Journal of endodontics
Alshehri M., Alamri H.M., Alshwaimi E., Kujan O.	Micro-computed tomographic assessment of quality of obturation in the apical third with continuous wave vertical compaction and single match taper sized cone obturation techniques	2016	Scanning
Xu K., Wang J., Wang K., Gen N., Li J.	Micro-computed tomographic evaluation of the effect of the final apical size prepared by rotary nickel-titanium files on the removal efficacy of hard-tissue debris	2018	Journal of International Medical Research
Berutti E, Moccia E, Lavino S, Multari S, Carpegna G, Scotti N, Pasqualini D, Alovisi M.	Micro-Computed Tomography Evaluation of Minimally Invasive Shaping Systems in Mandibular First Molars	2022	J Clin Med
Jambura PL, Kindlimann R, López-Romero F, Marramà G, Pfaff C, Stumpf S, Türtscher J, Underwood CJ, Ward DJ, Kriwet J.	Micro-computed tomography imaging reveals the development of a unique tooth mineralization pattern in mackerel sharks (Chondrichthyes; Lamniformes) in deep time	2019	Sci Rep
Lin X, Fu Y, Ren G, Yang X, Duan W, Chen Y, Zhang Q.	Micro-Computed Tomography-Guided Artificial Intelligence for Pulp Cavity and Tooth Segmentation on Cone-beam Computed Tomography	2021	J Endod
Kalantar Motamedi M.R., Mortaheb A., Zare Jahromi M., Gilbert B.E.	Micro-CT Evaluation of Four Root Canal Obturation Techniques	2021	Scanning
Phonghanyudh A.; Thana-olarn C.; Teanchai C.; Jirarattanasopha V.	Microhardness of sub-restoration dentine in primary molars after carious tissue removal to soft and firm dentine	2021	Pediatric Dental Journal
Farto J.; Canalda Sahli C.; Boj J.R.	Microleakage of MTA in primary molar pulpotomies	2017	European Journal of Paediatric Dentistry
Donyavi Z., Khoshbin E., Esmaeilzadeh M., Rezaei-Soufi L., Kermani N.	Microleakage of two root-end filling materials in the cavities prepared by laser and ultrasonic technique: An in-vitro study	2017	Italian Journal of Vascular and Endovascular Surgery
Yuan H.; Zhao H.; Wang J.; Zhang H.; Hong L.; Li H.; Che H.; Zhang Z.	MicroRNA let-7c-5p promotes osteogenic differentiation of dental pulp stem cells by inhibiting lipopolysaccharide-induced inflammation via HMGA2/PI3K/Akt signal blockade	2019	Clinical and Experimental Pharmacology and Physiology
Yuan H.; Zhang H.; Hong L.; Zhao H.; Wang J.; Li H.; Che H.; Zhang Z.	MicroRNA let-7c-5p suppressed lipopolysaccharide-induced dental pulp inflammation by inhibiting dentin matrix protein-1-mediated nuclear factor kappa b (NF-κb) pathway in vitro and in vivo	2018	Medical Science Monitor
Jiang L.; Krongbaramee T.; Lin X.; Zhu M.; Zhu Y.; Hong L.	microRNA-126 inhibits vascular cell adhesion molecule-1 and interleukin-1beta in human dental pulp cells	2022	Journal of Clinical Laboratory Analysis
Yang C.; Jia R.; Zuo Q.; Zheng Y.; Wu Q.; Luo B.; Lin P.; Yin L.	microRNA-143-3p regulates odontogenic differentiation of human dental pulp stem cells through regulation of the osteoprotegerin–RANK ligand pathway by targeting RANK	2020	Experimental Physiology
Li B.; Guo L.; He Y.; Tu X.; Zhong J.; Guan H.; Jiang Y.; Jiang Q.	MicroRNA-155 expression is associated with pulpitis progression by targeting SHIP1	2022	Molecular Biology Reports
Jiang W.; Sun S.; Wang D.; Qiu J.; Song Y.; Zhang Q.; He W.; Song B.; Zhang Y.; Wang S.	MicroRNA-22 suppresses NLRP3/CASP1 inflammasome pathway-mediated proinflammatory cytokine production by targeting the HIF-1α and NLRP3 in human dental pulp fibroblasts	2022	International Endodontic Journal

Wang D.; Sun S.; Xue Y.; Qiu J.; Ye T.; Zhang R.; Song B.; He W.; Zhang Y.; Jiang W.	MicroRNA-223 negatively regulates LPS-induced inflammatory responses by targeting NLRP3 in human dental pulp fibroblasts	2021	International Endodontic Journal
Wang J.; Du Y.; Deng J.; Wang X.; Long F.; He J.	MicroRNA-506 is involved in regulation of the occurrence of lipopolysaccharides (LPS)-induced pulpitis by sirtuin 1 (SIRT1)	2019	Medical Science Monitor
Shen Z.; Silva R.M.	Micrornas: Emerging players in apical periodontitis	2021	Journal of Applied Oral Science
Anthrayose P.; Aggarwal A.; Yadav S.; Nawal R.; Talwar S.	Microscopic and elemental characterization of hydrated dental pulp capping agents	2021	Journal of Conservative Dentistry
Kanafi M.; Majumdar D.; Bhonde R.; Gupta P.; Datta I.	Midbrain cues dictate differentiation of human dental pulp stem cells towards functional dopaminergic neurons	2014	Journal of Cellular Physiology
Kirici D.O., Koc S.	Middle distal canal of mandibular first molar: A case report and literature review	2019	Nigerian journal of clinical practice
Jabali A.H.	Middle Mesial and Middle Distal Canals in Mandibular First Molar	2018	The journal of contemporary dental practice
Park Y.H.; Lee Y.S.; Seo Y.M.; Seo H.; Park J.S.; Bae H.S.; Park J.C.	Midkine Promotes Odontoblast-like Differentiation and Tertiary Dentin Formation	2020	Journal of Dental Research
Zain S, Davis GR, Hill R, Anderson P, Baysan A.	Mineral exchange within restorative materials following incomplete carious lesion removal using 3D non-destructive XMT subtraction methodology	2020	J Dent
Daniele L.	Mineral Trioxide Aggregate (MTA) direct pulp capping: 10 years clinical results; Incappucciamento diretto della polpa con Mineral Trioxide Aggregate: risultati clinici a 10 anni	2017	Giornale Italiano di Endodonzia
Subay RK, Ilhan B, Ulukapi H.	Mineral trioxide aggregate as a pulpotomy agent in immature teeth: Long-term case report	2013	Eur J Dent
Wang Y.; Yan M.; Fan Z.; Ma L.; Yu Y.; Yu J.	Mineral trioxide aggregate enhances the odonto/osteogenic capacity of stem cells from inflammatory dental pulps via NF-κB pathway	2014	Oral Diseases
Qudeimat M.A.; Alyahya A.; Hasan A.A.; Barrieshi-Nusair K.M.	Mineral trioxide aggregate pulpotomy for permanent molars with clinical signs indicative of irreversible pulpitis: a preliminary study	2017	International Endodontic Journal
Kim Y.-J.; Kim W.-J.; Bae S.-W.; Yang S.-M.; Park S.-Y.; Kim S.-M.; Jung J.-Y.	Mineral trioxide aggregate-induced AMPK activation stimulates odontoblastic differentiation of human dental pulp cells	2021	International Endodontic Journal
de Melo C.C.D.S.B.; Cassiano F.B.; Bronze-Uhle É.S.; Stuani V.D.T.; Bordini E.A.F.; Gallinari M.D.O.; de Souza Costa C.A.; Soares D.G.	Mineral-induced bubbling effect and biomineralization as strategies to create highly porous and bioactive scaffolds for dentin tissue engineering	2022	Journal of Biomedical Materials Research - Part B Applied Biomaterials
Asgary S.; Nourzadeh M.; Eghbal M.J.	Miniature pulpotomy of symptomatic mature permanent teeth: A report of two cases	2016	Iranian Endodontic Journal
BaniHani A, Hamid A, Van Eeckhoven J, Gizani S, Albadri S.	Minimal Intervention Dentistry (MID) mainstream or unconventional option? Study exploring the impact of COVID-19 on paediatric dentists' views and practices of MID for managing carious primary teeth in children across the United Kingdom and European Union	2022	Eur Arch Paediatr Dent
BaniHani A, Santamaría RM, Hu S, Maden M, Albadri S.	Minimal intervention dentistry for managing carious lesions into dentine in primary teeth: an umbrella review	2022	Eur Arch Paediatr Dent
Freitas M.F.L.; Santos J.M.; Fuks A.; Bezerra A.C.B.; Azevedo T.D.P.L.	Minimal Intervention Dentistry procedures: A ten year retrospective study	2014	Journal of Clinical Pediatric Dentistry
Chaussain C.; Poliard A.	Minimal intervention dentistry: Part 8. Biotherapies for the dental pulp	2014	British Dental Journal
Gatón-Hernandéz P, Serrano CR, da Silva LAB, de Castañeda ER, da Silva RAB, Pucinelli CM, Manton D, Ustrell-Torrent JM, Nelson-Filho P.	Minimally interventive restorative care of teeth with molar incisor hypomineralization and open apex-A 24-month longitudinal study	2020	Int J Paediatr Dent
Opal S, Garg S, Dhindsa A, Taluja T.	Minimally invasive clinical approach in indirect pulp therapy and healing of deep carious lesions	2014	J Clin Pediatr Dent
Philip N.; Suneja B.	Minimally invasive endodontics: a new era for pulpotomy in mature permanent teeth	2022	British Dental Journal
Zhang Y.-F.; Zhou L.; Mao H.-Q.; Yang F.-H.; Chen Z.; Zhang L.	Mitochondrial DNA leakage exacerbates odontoblast inflammation through gasdermin D-mediated pyroptosis	2021	Cell Death Discovery

Zhou L, Zhang YF, Yang FH, Mao HQ, Chen Z, Zhang L.	Mitochondrial DNA leakage induces odontoblast inflammation via the cGAS-STING pathway	2021	Cell Commun Signal
Bushmanova G.M.; Zorina I.G.; Nikityuk D.B.; Nepomnyashchikh R.D.; Lapii G.A.; Postnikova O.A.; Semenov D.E.	Mitral and aortic valvulitis in primary chronic septic endocarditis	2015	Bulletin of Experimental Biology and Medicine
Muromachi K.; Kamio N.; Narita T.; Annen-Kamio M.; Sugiya H.; Matsushima K.	MMP-3 provokes CTGF/CCN2 production independently of protease activity and dependently on dynamin-related endocytosis, which contributes to human dental pulp cell migration	2012	Journal of Cellular Biochemistry
Ballal N.V.; Duncan H.F.; Wiedemeier D.B.; Rai N.; Jalan P.; Bhat V.; Belle V.S.; Zehnder M.	MMP-9 Levels and NaOCl Lavage in Randomized Trial on Direct Pulp Capping	2022	Journal of Dental Research
Nakashima M.; Iohara K.	Mobilized dental pulp stem cells for pulp regeneration: Initiation of clinical trial	2014	Journal of Endodontics
Jawad S., Taylor C., Roudsari R.V., Darcey J., Qualtrough A.	Modern Endodontic Planning Part 1: Assessing Complexity and Predicting Success	2015	Dental update
Takegawa D.; Nakanishi T.; Hirao K.; Yumoto H.; Takahashi K.; Matsuo T.	Modulatory roles of interferon-γ through indoleamine 2, 3-dioxygenase induction in innate immune response of dental pulp cells	2014	Journal of endodontics
Nomura R.; Ogaya Y.; Matayoshi S.; Morita Y.; Nakano K.	Molecular and clinical analyses of Helicobacter pylori colonization in inflamed dental pulp	2018	BMC Oral Health
Alexandru-Andrei I., Perlea P., Irina-Maria G., Mihai M., Loredana M., Iren M., Sînziana S., Erdoğan Ş., Andrei I.	Molecular Mechanisms of Dentine-Pulp Complex Response Induced by Microbiome of Deep Caries	2019	ARS Medica Tomitana
Bhardwaj A.; Kottoor J.; Albequerque D.V.; Velmurugan N.	Morphologic variations in mandibular premolars: A report of three cases	2015	Journal of Contemporary Dental Practice
Lee JH, Lee C, Battulga B, Na JY, Hwang JJ, Kim YH, Han SS.	Morphological analysis of the lower second premolar for age estimation of Korean adults	2017	Forensic Sci Int
Bertonnier-Brouty L, Viriot L, Joly T, Charles C.	Morphological features of tooth development and replacement in the rabbit Oryctolagus cuniculus	2020	Arch Oral Biol
Stefanova V.P.; Tomov G.T.; Tsanova S.Ts.	Morphological Study Of Border Area Of Pulp-Capping Materials And Er:YAG Laser Prepared Hard Dental Surface	2015	Folia medica
Chen L.; Liu G.; Wu J.; Zhou X.; Zhao Y.; Chen Z.; Lin Z.; Xiao Y.	Multi-faceted effects of mesenchymal stem cells (MSCs) determined by immune microenvironment and their implications on MSC/biomaterial-based inflammatory disease therapy	2020	Applied Materials Today
Chi C.-W.; Lohanathan B.P.; Wong C.-C.; Chen C.-L.; Lin H.-C.; Chiang Y.-C.	Multiple growth factors accommodated degradable submicron calcium sulfate hemihydrate/porous hydroxyapatite for dentin-pulp regeneration	2022	Biomaterials Advances
Seung JG, Kim JG, Yang YM, Lee DW.	Multiple Idiopathic Internal Root Resorption in an Adolescent: A Case R Multiple Idiopathic Internal Root Resorption in an Adolescent: A Case Report	2021	J Clin Pediatr Dent
Lee S.-I.; Kang S.-K.; Jung H.-J.; Chun Y.-H.; Kwon Y.-D.; Kim E.-C.	Muramyl dipeptide activates human beta defensin 2 and pro-inflammatory mediators through Toll-like receptors and NLRP3 inflammasomes in human dental pulp cells	2015	Clinical Oral Investigations
Abuzenada B.M.; Souror Y.R.; Waly A.S.; Khlifa Y.H.A.	Mutans streptococci growth on glass ionomer incorporated with chlorhexidine: In-vivo study; Crescimento de estreptococos mutans em ionômero de vidro incorporado com clorexidina: Estudo in vivo	2020	Brazilian Dental Science
Xiong F, Ji Z, Liu Y, Zhang Y, Hu L, Yang Q, Qiu Q, Zhao L, Chen D, Tian Z, Shang X, Zhang L, Wei X, Liu C, Yu Q, Zhang M, Cheng J, Xiong J, Li D, Wu X, Yuan H, Zhang W, Xu X.	Mutation in SSUH2 Causes Autosomal-Dominant Dentin Dysplasia Type I	2017	Hum Mutat
Suwanchai A.; Theerapiboon U.; Chattipakorn N.; Chattipakorn S.C.	Na V1.8, but not Na V1.9, is upregulated in the inflamed dental pulp tissue of human primary teeth	2012	International Endodontic Journal
Chiang Y.-C.; Chang H.-H.; Wong C.-C.; Wang Y.-P.; Wang Y.-L.; Huang W.-H.; Lin C.-P.	Nanocrystalline calcium sulfate/hydroxyapatite biphasic compound as a TGF-β1/VEGF reservoir for vital pulp therapy	2016	Dental Materials
Lee D.-K.; Kim S.V.; Limansubroto A.N.; Yen A.; Soundia A.; Wang C.-Y.; Shi W.; Hong C.; Tetradis S.; Kim Y.; Park N.-H.; Kang M.K.; Ho D.	Nanodiamond-Gutta Percha Composite Biomaterials for Root Canal Therapy	2015	ACS Nano

Kuang R, Zhang Z, Jin X, Hu J, Shi S, Ni L, Ma PX.	Nanofibrous spongy microspheres for the delivery of hypoxia-primed human dental pulp stem cells to regenerate vascularized dental pulp	2016	Acta Biomater
Mendes Soares I.P.; Anselmi C.; Kitagawa F.A.; Ribeiro R.A.D.O.; Leite M.L.; de Souza Costa C.A.; Hebling J.	Nano-hydroxyapatite-incorporated polycaprolactone nanofibrous scaffold as a dentin tissue engineering-based strategy for vital pulp therapy	2022	Dental Materials
Ji Y.; Choi S.K.; Sultan A.S.; Chuncai K.; Lin X.; Dashtimoghadam E.; Melo M.A.; Weir M.; Xu H.; Tayebi L.; Nie Z.; Depireux D.A.; Masri R.	Nanomagnetic-mediated drug delivery for the treatment of dental disease	2018	Nanomedicine: Nanotechnology, Biology, and Medicine
Corral Nunez C.; Altamirano Gaete D.; Maureira M.; Martin J.; Covarrubias C.	Nanoparticles of bioactive glass enhance biodentine bioactivity on dental pulp stem cells	2021	Materials
Lee S.M.; Leem D.H.	Nasal septal abscess with a dental origin: A case report and a review of the literature	2021	Journal of the Korean Association of Oral and Maxillofacial Surgeons
Sun S.; Sun J.; Jiang W.; Wang W.; Ni L.	Nav1.7 via promotion of ERK in the trigeminal ganglion plays an important role in the induction of pulpitis inflammatory pain	2019	BioMed Research International
Deng Z.; Yan W.; Dai X.; Chen M.; Qu Q.; Wu B.; Zhao W.	N-Cadherin Regulates the Odontogenic Differentiation of Dental Pulp Stem Cells via β-Catenin Activity	2021	Frontiers in Cell and Developmental Biology
Cao R.; Wang Q.; Wu J.; Liu M.; Han Q.; Wang X.	Nell-1 attenuates lipopolysaccharide-induced inflammation in human dental pulp cells	2021	Journal of Molecular Histology
Han Q, Wang Q, Wu J, Li M, Fang Y, Zhu H, Wang X.	Nell-1 promotes the neural-like differentiation of dental pulp cells	2019	Biochem Biophys Res Commun
Gomes N.A.; do Valle I.B.; Gleber-Netto F.O.; Silva T.A.; Oliveira H.M.D.C.; de Oliveira R.F.; Ferreira L.D.A.Q.; Castilho L.S.; Reis P.H.R.G.; Prazeres P.H.D.M.; Menezes G.B.; de Magalhães C.S.; Mesquita R.A.; Marques M.M.; Birbrair A.; Diniz I.M.A.	Nestin and NG2 transgenes reveal two populations of perivascular cells stimulated by photobiomodulation	2022	Journal of Cellular Physiology
Sampoerno G.; Bhardwaj A.; Divina P.Y.; Fripertiwi N.N.; Adipradana N.H.	Neurogenic Inflammation Pathway on the Up-Regulation of Voltage-Gated Sodium Channel NaV1.7 in Experimental Flare-Up Post-Dental Pulp Tissue Extirpation	2022	Journal of International Dental and Medical Research
Dagna A.	Nickel-Titanium Single-file System in Endodontics	2015	Journal of Contemporary Dental Practice
Omar O.M.; Khattab N.M.; Khater D.S.	Nigella sativa oil as a pulp medicament for pulpotomized teeth: A histopathological evaluation	2012	Journal of Clinical Pediatric Dentistry
Song Z.; Lin Z.; He F.; Jiang L.; Qin W.; Tian Y.; Wang R.; Huang S.	NLRP3 is expressed in human dental pulp cells and tissues	2012	Journal of Endodontics
Tian X.X.; Li R.; Liu C.; Liu F.; Yang L.J.; Wang S.P.; Wang C.L.	NLRP6-caspase 4 inflammasome activation in response to cariogenic bacterial lipoteichoic acid in human dental pulp inflammation	2021	International Endodontic Journal
Elkhadem A, Sami I.	No clear evidence of superiority regarding pulp medicaments in primary molars	2014	Evid Based Dent
Lee S.-I.; Kim G.-T.; Kim H.J.; Park S.-H.; Kim E.-C.	NOD2 Mediates odontoblast differentiation and RANKL expression	2014	Journal of Dental Research
Okuno M.; Harada K.; Inoue H.; Moroto H.; Zhang H.; Zhang R.; Mu M.; Hosoyama C.; Hosoyama Y.; Fukawa A.; Sugimoto W.; Matsumoto N.; Yamamoto K.; Goda S.	NOD2 Regulates the Production of MMP-3 in Human Deciduous Dental Pulp Fibroblast-like Cells	2021	Nano Biomedicine
Nanjannawar G.; Mulla S.; Gupta D.; Kamat S.	Non surgical management of class I invasive cervical resorption and anterior aesthetic rehabilitation: A case report with 2-years follow-up	2021	Tropical Journal of Pharmaceutical Research
Kunert G.G.; Kunert I.R.; De Figueiredo J.A.P.; Barletta F.B.; Estrela C.	Nonconventional therapeutic protocol for type III dens invaginatus	2017	Journal of Contemporary Dental Practice
Hervella M, Iñiguez MG, Izagirre N, Anta A, de-la-Rúa C.	Nondestructive methods for recovery of biological material from human teeth for DNA extraction	2015	J Forensic Sci
Sinescu C.; Negrutiu M.L.; Bradu A.; Duma V.-F.; Podoleanu A.G.	Noninvasive quantitative evaluation of the dentin layer during dental procedures using optical coherence tomography	2015	Computational and Mathematical Methods in Medicine
Yue W., Kim E.	Nonsurgical Endodontic Management of a Molar-Incisor Malformation-affected Mandibular First Molar: A Case Report	2016	Journal of endodontics

Musale P.K., Kothare S.	Non-surgical endodontic management of immature permanent mandibular first molar: a 3 year follow-up	2018	European archives of paediatric dentistry : official journal of the European Academy of Paediatric Dentistry
Kato H.	Non-surgical endodontic treatment for dens invaginatus type III using cone beam computed tomography and dental operating microscope: a case report	2013	Bull Tokyo Dent Coll
Mashyakhy M., Chourasia H.R., Halboub E., Roges R.A., Gambarini G.	Nonsurgical Management and 2-year Follow-up by means of Cone Beam Computed Tomography of an Invasive Cervical Resorption in a Molar	2018	The journal of contemporary dental practice
Ghorbanzadeh S.; Ashraf H.; Hosseinpour S.; Ghorbanzadeh F.	Nonsurgical management of a large periapical lesion: A case report	2017	Iranian Endodontic Journal
Varghese L.L.; Bhattacharya A.; Sharma P.; Apratim A.	Non-surgical management of an extraoral cutaneous sinus tract of odontogenic origin	2020	BMJ Case Reports
Tamilselvi R., Megavarnan R., Prakash V., Subbiya A.	Nonsurgical management of periapical lesion with external root resorption followed by orthodontic treatment in maxillary lateral incisor	2019	Indian Journal of Public Health Research and Development
Patil S.S.; Yamuna S.	Nonvital Pulp Therapy for Primary Teeth	2021	Pediatric dentistry
Park C.-S.; Cha S.-Y.; Kang M.; Kim J.; Jeong S.-W.; Jang H.-K.	Nonvital pulp therapy of elongation of roots of mandibular cheek teeth in pet rabbits	2012	Journal of Veterinary Clinics
Wang L, Fu H, Wang W, Liu Y, Li X, Yang J, Li L, Wu G, Pan Y.	Notoginsenoside R1 functionalized gelatin hydrogels to promote reparative dentinogenesis	2021	Acta Biomater
Livingstone D.; Manivasakan S.; Shivashankarappa P.G.	Novel Endodontic Simulation Trainer for Dental Pulpotomy and Pulp Capping	2022	Journal of International Oral Health
Okamoto M.; Takahashi Y.; Komichi S.; Ali M.; Yoneda N.; Ishimoto T.; Nakano T.; Hayashi M.	Novel evaluation method of dentin repair by direct pulp capping using high-resolution micro-computed tomography	2018	Clinical Oral Investigations
Wu S.; Weir M.D.; Lei L.; Liu J.; Xu H.H.K.	Novel nanographene oxide-calcium phosphate cement inhibits Enterococcus faecalis biofilm and supports dental pulp stem cells	2021	Journal of Orthopaedic Surgery and Research
Baras B.H.; Sun J.; Melo M.A.S.; Tay F.R.; Oates T.W.; Zhang K.; Weir M.D.; Xu H.H.K.	Novel root canal sealer with dimethylaminohexadecyl methacrylate, nano-silver and nano-calcium phosphate to kill bacteria inside root dentin and increase dentin hardness	2019	Dental Materials
Gug H.R.; Park Y.-H.; Park S.-J.; Jang J.Y.; Lee J.-H.; Lee D.-S.; Shon W.-J.; Park J.-C.	Novel strategy for dental caries by physiologic dentin regeneration with CPNE7 peptide	2022	Archives of Oral Biology
Kumar S., Kiran K.P., Achutha G., Muthe K., Surana P., Sagare S.V.	Nusmile zirconia crowns: Pedodontist perception to restore esthetic smiles	2021	European Journal of Molecular and Clinical Medicine
Wang X.; Sun H.; Hu Z.; Mei P.; Wu Y.; Zhu M.	NUTM2A-AS1 silencing alleviates LPS-induced apoptosis and inflammation in dental pulp cells through targeting let-7c-5p/HMGB1 axis	2021	International Immunopharmacology
Serindere G, Belgin CA, Orhan K.	Observation of the Pulp Chamber of Maxillary First Premolars: A Micro-computed Tomographic Study	2020	Curr Med Imaging
Rosenberg L, Atar M, Daronch M, Honig A, Chey M, Funny MD, Cruz L.	Observational: prospective study of indirect pulp treatment in primary molars using resin-modified glass ionomer and 2% chlorhexidine gluconate: a 12-month Follow-up	2013	Pediatr Dent
Balto H.A.	Obturation Techniques Allow Microbial Leakage Unless Protected	2016	Journal of prosthodontics : official journal of the American College of Prosthodontists
Asif A., Emg S.	Obturation techniques in primary teeth	2020	International Journal of Research in Pharmaceutical Sciences
Carvalho JC, Dige I, Machiulskiene V, Qvist V, Bakhshandeh A, Fatturi-Parolo C, Maltz M.	Occlusal Caries: Biological Approach for Its Diagnosis and Management	2016	Caries Res
Pollaris E.; Staszyk C.; Proost K.; Boone M.N.; Josipovic I.; Pardon B.; Vlaminck L.	Occlusal fissures in equine cheek teeth: μCT and histological findings	2020	Veterinary Journal
Liu L.; Huang R.; Yang R.; Wei X.	OCT4B1 regulates the cellular stress response of human dental pulp cells with inflammation	2017	BioMed Research International
Farges J.-C.; Alliot-Licht B.; Baudouin C.; Msika P.; Bleicher F.; Carrouel F.	Odontoblast control of dental pulp inflammation triggered by cariogenic bacteria	2013	Frontiers in Physiology

Chang S.-W.; Bae W.-J.; Yi J.-K.; Lee S.; Lee D.-W.; Kum K.-Y.; Kim E.-C.	Odontoblastic Differentiation, Inflammatory Response, and Angiogenic Potential of 4 Calcium Silicate-based Cements: Micromega MTA, ProRoot MTA, RetroMTA, and Experimental Calcium Silicate Cement	2015	Journal of Endodontics
Wang H.S.; Yang F.H.; Wang Y.J.; Pei F.; Chen Z.; Zhang L.	Odontoblastic Exosomes Attenuate Apoptosis in Neighboring Cells	2019	Journal of Dental Research
Babaki D.; Matin M.M.	Odontoblast-like Cytodifferentiation of Dental Stem Cells: A review	2020	Iranian Endodontic Journal
Park S.-J.; Heo S.-M.; Hong S.-O.; Hwang Y.-C.; Lee K.-W.; Min K.-S.	Odontogenic effect of a fast-setting pozzolan-based pulp capping material	2014	Journal of Endodontics
Kim K.-A.; Yang Y.-M.; Kwon Y.-S.; Hwang Y.-C.; Yu M.-K.; Min K.-S.	Odontogenic effects of a fast-setting calcium-silicate cement containing zirconium oxide	2015	Dental Materials Journal
Önay E.O.; Yurtcu E.; Terzi Y.K.; Üngör M.; Oguz Y.; Şahin F.I.	Odontogenic effects of two calcium silicate-based biomaterials in human dental pulp cells	2018	Advances in Clinical and Experimental Medicine
Sato K.	Odontogenic maxillary sinusitis caused by dental restoration	2014	Nihon Jibiinkoka Gakkai Kaiho
Fransson H.	On the repair of the dentine barrier.	2012	Swedish dental journal. Supplement
Al-Attiya H.; Schmoeckel J.; Mourad M.S.; Splieth C.H.	One year clinical success of pulpectomy in primary molars with iodoform-calcium hydroxide paste	2021	Quintessence International
Villat C, Attal JP, Brulat N, Decup F, Doméjean S, Dursun E, Fron-Chabouis H, Jacquot B, Muller Bolla M, Plasse-Pradelle N, Roche L, Maucort-Boulch D, Nony P, Gritsch K, Millet P, Gueyffier F, Grosgogeat B.	One-step partial or complete caries removal and bonding with antibacterial or traditional self-etch adhesives: study protocol for a randomized controlled trial	2016	Trials
de Araújo L.P., de Azevedo Kinalski M., Carpena L.P., Ferraz C.C.R., Dos Santos M.B.F.	One-year follow-up of endodontic retreatment in a patient with severe Hemophilia A	2022	Giornale Italiano di Endodonzia
Ricketts D, Lamont T, Innes NP, Kidd E, Clarkson JE.	Operative caries management in adults and children	2013	Cochrane Database Syst Rev
Soares de Toubes KM, Moreira Maia L, Cota Goulart L, de Freitas Teixeira T, Silva NRFA, Isaías Seraidarian P, Silveira FF.	Optimization of Results for Cracked Teeth Using CAD-CAM System: A Case Series	2020	Iran Endod J
Ohshima H, Mishima K, Amizuka N.	Oral biosciences: The annual review 2021	2022	J Oral Biosci
Yip K, Smales R.	Oral diagnosis and treatment planning: part 2. Dental caries and assessment of risk	2012	Br Dent J
Duarte-Rodrigues L, Ramos-Jorge ML, Alves-Duarte AC, Fonseca-Silva T, Flores-Mir C, Marques LS.	Oral disorders associated with the experience of verbal bullying among Brazilian school-aged children: A case-control study	2020	J Am Dent Assoc
Wehby G.L.	Oral Health and Academic Achievement of Children in Low-Income Families	2022	Journal of Dental Research
Shulamithi P.S.; Kulkarni S.; Doshi D.; Reddy M.P.; Srilatha A.; Satyanarayana D.	Oral Health Illness Perception and Dental Caries: A Cross-sectional Study among Adult Dental Patients, Hyderabad, Telangana, India	2022	World Journal of Dentistry
Folwaczny M., Wilberg S., Bumm C., Hollatz S., Oberhofer R., Neidenbach R.C., Kaemmerer H., Frasheri I.	Oral health in adults with congenital heart disease	2019	Journal of Clinical Medicine
Singh D.P.	Oral Health in Leprosy as a Public Health Issue in India: Review of Literature	2021	Indian Journal of Leprosy
Zhong C., Ma K.N., Wong Y.S., So Y., Lee P.C., Yang Y.	Oral Health Knowledge of Pregnant Women on Pregnancy Gingivitis and Children's Oral Health	2015	The Journal of clinical pediatric dentistry
Oh T.J., Nam O.H., Kim M.S., Choi S.C., Lee H.-S.	Oral Health of Patients with Special Health Care Needs After General Anesthesia: A 25- Year Retrospective Study	2018	Pediatric dentistry
Karki S, Laitala ML, Humagain M, Seppänen M, Päkkila J, Anttonen V.	Oral health status associated with sociodemographic factors of Nepalese schoolchildren: a population-based study	2018	Int Dent J
Chen C.-Y.; Chen Y.-W.; Tsai T.-P.; Shih W.-Y.	Oral health status of children with special health care needs receiving dental treatment under general anesthesia at the dental clinic of Taipei Veterans General Hospital in Taiwan	2014	Journal of the Chinese Medical Association
Jain A.; Jayaraj G.; Pradeep Kumar R.	Oral lesions in diabetes mellitus patients visiting a dental hospital	2020	International Journal of Pharmaceutical Research

Cowan G.M.; Lockey R.F.	Oral Manifestations of Allergic, Infectious, and Immune-mediated Disease	2014	Journal of Allergy and Clinical Immunology: In Practice
Brunet-Llobet L.; Lahor-Soler E.; Miranda-Rius J.	Oral pain due to severe pre-eruptive intracoronal resorption in permanent tooth	2014	European Journal of Paediatric Dentistry
Brkić Z., Pijevčević N., Pavlić V., Petronijević M.	Oral rehabilitation of a patient with temporomandibular joint ankylosis caused by ankylosing spondylitis: A case report	2017	Vojnosanitetski Pregled
Zhang W., Wang J., Yu X., Wang W.	Orofacial granulomatosis: A case report of three cases may be caused by apical periodontitis	2017	Medicine (United States)
Nishihara C.; Hatori K.; Hsu Y.-C.; Ozasa K.; Young A.; Imamura Y.; Noma N.	Orofacial pain and menstrually related migraine	2019	Acta Neurologica Taiwanica
Rathore K., Naik D., Lenka S., Nagarajappa R., Das U.	Orthodontic considerations for traumatized teeth: An overview	2020	Indian Journal of Forensic Medicine and Toxicology
Thiesen G, Oliver DR, Araújo EA.	Orthodontic treatment of a patient with maxillary lateral incisors with dens invaginatus: 6-year follow-up	2018	Am J Orthod Dentofacial Orthop
Nikolaeva EP, Cox TC, Flake NM.	Osseous characteristics of mice lacking cannabinoid receptor 2 after pulp exposure	2015	J Endod
Abd-Elmeguid A.; Abdeldayem M.; Kline L.W.; Moqbel R.; Vliagoftis H.; Yu D.C.	Osteocalcin expression in pulp inflammation	2013	Journal of Endodontics
Ajlan S.A.; Ashri N.Y.; Aldahmash A.M.; Alnbaheen M.S.	Osteogenic differentiation of dental pulp stem cells under the influence of three different materials	2015	BMC Oral Health
Sato N.; Isomura M.; Kawai R.; Yoshida W.; Sugita Y.; Kubo K.; Funato A.; Ueno N.; Jinno M.; Maeda H.	Osteogenic potential of rat dental pulp-derived cells on titanium surfaces	2018	Journal of Hard Tissue Biology
Pei S.-L.; Shih W.-Y.; Liu J.-F.	Outcome comparison between diode laser pulpotomy and formocresol pulpotomy on human primary molars	2020	Journal of Dental Sciences
Ricucci D, Rôças IN, Alves FRF, Cabello PH, Siqueira JF Jr.	Outcome of Direct Pulp Capping Using Calcium Hydroxide: A Long-term Retrospective Study	2023	J Endod
Marques M.S., Wesselink P.R., Shemesh H.	Outcome of Direct Pulp Capping with Mineral Trioxide Aggregate: A Prospective Study	2015	Journal of endodontics
Hussain MI, Bashar AM.	Outcome of Mineral Trioxide Aggregate Pulpotomy for Mature Permanent Molars with Symptoms Indicative of Irreversible Pulpitis	2022	Mymensingh Med J
Jahreis M.; Soliman S.; Schubert A.; Connert T.; Schlagenhauf U.; Krastl G.; Krug R.	Outcome of non-surgical root canal treatment related to periodontitis and chronic disease medication among adults in age group of 60 years or more	2019	Gerodontology
Elmsmari F, Ruiz XF, Miró Q, Feijoo-Pato N, Durán-Sindreu F, Olivieri JG.	Outcome of Partial Pulpotomy in Cariously Exposed Posterior Permanent Teeth: A Systematic Review and Meta-analysis	2019	J Endod
Passia N.; Chaar M.S.; Kern M.	Outcome of posterior fixed dental prostheses made from veneered zirconia over an observation period of up to 13 years	2019	Journal of Dentistry
Ather A.; Patel B.; Gelfond J.A.L.; Ruparel N.B.	Outcome of pulpotomy in permanent teeth with irreversible pulpitis: a systematic review and meta-analysis	2022	Scientific Reports
Llena C., Nicolescu T., Perez S., de Pereda S.G., Gonzalez A., Alarcon I., Monzo A., Sanz J.L., Melo M., Forner L.	Outcome of root canal treatments provided by endodontic postgraduate students. A retrospective study	2020	Journal of Clinical Medicine
Garrocho-Rangel A, Esparza-Villalpando V, Pozos-Guillen A.	Outcomes of direct pulp capping in vital primary teeth with cariously and non-cariously exposed pulp: A systematic review	2020	Int J Paediatr Dent
Franzon R, Guimarães LF, Magalhães CE, Haas AN, Araujo FB.	Outcomes of one-step incomplete and complete excavation in primary teeth: a 24-month randomized controlled trial	2014	Caries Res
Khokhar M.; Tewari S.	Outcomes of partial and complete caries excavation in permanent teeth: A 18 month clinical study	2018	Contemporary Clinical Dentistry
Alassadi M., Qazi M., Ravidà A., Siqueira R., Garaicoa-Pazmiño C., Wang H.-L.	Outcomes of root resection therapy up to 16.8 years: A retrospective study in an academic setting	2020	Journal of Periodontology
BaniHani A, Duggal M, Toumba J, Deery C.	Outcomes of the conventional and biological treatment approaches for the management of caries in the primary dentition	2018	Int J Paediatr Dent

Awawdeh L, Al-Qudah A, Hamouri H, Chakra RJ.	Outcomes of Vital Pulp Therapy Using Mineral Trioxide Aggregate or Biodentine: A Prospective Randomized Clinical Trial	2018	J Endod
Cushley S, Duncan HF, Lundy FT, Nagendrababu V, Clarke M, El Karim I.	Outcomes reporting in systematic reviews on vital pulp treatment: A scoping review for the development of a core outcome set	2022	Int Endod J
Tancharoen S.; Tengrungsun T.; Suddhasthira T.; Kikuchi K.; Vechvongvan N.; Tokuda M.; Maruyama I.	Overexpression of receptor for advanced glycation end products and high-mobility group box 1 in human dental pulp inflammation	2014	Mediators of Inflammation
Moreno T., Sanz J.L., Melo M., Llena C.	Overtreatment in restorative dentistry: Decision making by last-year dental students	2021	International Journal of Environmental Research and Public Health
Vengerfeldt V, Mändar R, Saag M, Piir A, Kullisaar T.	Oxidative stress in patients with endodontic pathologies	2017	J Pain Res
Ruparel S.; Hargreaves K.M.; Eskander M.; Rowan S.; De Almeida J.F.A.; Roman L.; Henry M.A.	Oxidized linoleic acid metabolite-cytochrome P450 system (OLAM-CYP) is active in biopsy samples from patients with inflammatory dental pain	2013	Pain
Kumagai T.; Shindo S.; Takeda K.; Shiba H.	Oxytocin suppresses CXCL10 production in TNF-α-stimulated human dental pulp stem cells	2022	Cell Biology International
Nogales C.G.; Ferreira M.B.; Montemor A.F.; Rodrigues M.F.A.; Lage-Marques J.L.; Antoniazzi J.H.	Ozone therapy as an adjuvant for endondontic protocols: Microbiological – Ex vivo study and citotoxicity analyses	2016	Journal of Applied Oral Science
Al-Jundi S.H.; EI Shahawy O.I.; Nazzal H.	Paediatric dentistry undergraduate education across dental schools in the Arabian region: a cross-sectional study	2021	European Archives of Paediatric Dentistry
Olivi G, Caprioglio C, Olivi M, Genovese MD.	Paediatric laser dentistry. Part 2: Hard tissue laser applications	2017	Eur J Paediatr Dent
Porporatti A.L.; Bonjardim L.R.; Stuginski-Barbosa J.; Bonfante E.A.; Costa Y.M.; Conti P.C.R.	Pain from dental implant placement, inflammatory pulpitis pain, and neuropathic pain present different Somatosensory profiles	2017	Journal of Oral and Facial Pain and Headache
Debevc D, Hitij T, Kansky A.	Painful neuropathy caused by compression of the inferior alveolar nerve by focal osteosclerotic lesion of the mandible: A case report	2017	Quintessence Int
Pinheiro TN, Cintra LTA, Azuma MM, Benetti F, Silva CC, Consolaro A.	Palatogingival groove and root canal instrumentation	2020	Int Endod J
Satpathy A., Mohanty G., Datta P., Mohanty R., Nayak R., Panda S.	Palatogingival groove and the perio-endo lesion: Management through endo–perio regenerative (IPRF) approach: A case report	2019	Indian Journal of Public Health Research and Development
Song F.; Sun H.; Wang Y.; Yang H.; Huang L.; Fu D.; Gan J.; Huang C.	Pannexin3 inhibits TNF-α-induced inflammatory response by suppressing NF-κB signalling pathway in human dental pulp cells	2017	Journal of Cellular and Molecular Medicine
Sridhar M.; Mathew M.G.; Meenakshi	PANORAMIC RADIOGRAPHS AND ITS IMPORTANCE IN PEDIATRIC DENTISTRY – A RETROSPECTIVE STUDY	2022	International Journal of Clinical Dentistry
Gibbs J.L.; Urban R.; Basbaum A.I.	Paradoxical surrogate markers of dental injury-induced pain in the mouse	2013	Pain
Prasad P., Ahirwal S., Kumar V., Chauhan M.S., Rathor P.	Paramolar with a para-premolar: A rare occurrence	2017	Journal of Clinical and Diagnostic Research
Al-Batayneh O.B.; Al-Khateeb H.O.; Ibrahim W.M.; Khader Y.S.	Parental Knowledge and Acceptance of Different Treatment Options for Primary Teeth Provided by Dental Practitioners	2019	Frontiers in Public Health
Meenapriya M.; Gurunathan D.; Rajendran D.	Parents concern regarding dental treatment for children during covid-19-a survey	2020	International Journal of Current Research and Review
Manton D.	Partial caries removal may have advantages but limited evidence on restoration survival	2013	Evid Based Dent
Asgary S.; Roghanizadeh L.	Partial necrosis consequence of the infection spreading from an adjacent apical periodontitis: A case report	2018	Iranian Endodontic Journal
Motoki O.; Fergus D.H.; Yusuke T.; Nanako K.; Matsumoto S.; Mikako H.	Partial Pulpotomy to Successfully Treat a Caries-Induced Pulpal Micro-Abscess: A Case Report	2021	Frontiers in Dental Medicine
Tuloglu N.; Bayrak S.	Partial pulpotomy with BioAggregate in complicated crown fractures: Three case reports	2016	Journal of Clinical Pediatric Dentistry
Shchetinin E.V.; Sirak S.V.; Khodzhayan A.B.; Dilekova O.V.; Sirak A.G.; Vafiadi M.Y.; Parazyan L.A.; Arutyunov A.V.	Pathogenetic aspects of dental pulp pathology	2015	Medical News of North Caucasus

Sirak S.V.; Shchetinin E.V.; Vafiadi M.I.; Parazian L.A.	Pathophysiological and morphofunctional reaction of a dental pulp under inflammation	2015	Research Journal of Pharmaceutical, Biological and Chemical Sciences
Schwendicke F., Mostajaboldave R., Otto I., Dörfer C.E., Burkert S.	Patients' preferences for selective versus complete excavation: A mixed-methods study	2016	Journal of dentistry
Di Giuseppe G.; Lanzano R.; Silvestro A.; Napolitano F.; Pavia M.	Pattern and Appropriateness of Antimicrobial Prescriptions for Upper Respiratory Tract and Dental Infections in Male Prisoners in Italy	2021	Antibiotics
Alsaegh M.A., Albadrani A.W.	Pattern and reasons for permanent tooth extractions at dental clinics of the university of science and technology of Fujairah, UAE	2020	Open Dentistry Journal
Liu Y.; Zhang Z.; Li W.; Tian S.	PECAM1 Combines With CXCR4 to Trigger Inflammatory Cell Infiltration and Pulpitis Progression Through Activating the NF-κB Signaling Pathway	2020	Frontiers in Cell and Developmental Biology
da Penha E.S.; Rolim A.K.A.; Dos Santos C.A.O.; Guênes G.M.T.; de Medeiros L.A.D.M.; da Costa C.H.M.	Pediatric dentistry content in civil service examinations in the state of paraÍba: How is this specialty being addressed?; Conteúdo de odontopediatria em concursos públicos na paraíba: De que forma essa especialidade está sendo abordada?	2020	Bioscience Journal
Paul R., Ravindran V., Gurunathan D.	Pediatric dentists preference on using hand or rotary instruments in single visit permanent molars	2020	International Journal of Research in Pharmaceutical Sciences
Vieira AM, Ray HL Jr.	Pediatric Endodontic Treatment of Adolescent Patients	2021	Dent Clin North Am
Akshaya K., Ravindran V., Pandurangan S.M.	Pediatrisc dentists' preference on usage of hand files or rotary files for pulpectomy of primary molars-a retrospective study	2020	International Journal of Research in Pharmaceutical Sciences
Kim Y.; Park J.-S.; Park H.-J.; Kim M.-K.; Kim Y.-I.; Bae S.-K.; Kim H.J.; Jeong C.-H.; Bae M.-K.	Pentraxin 3 Modulates the Inflammatory Response in Human Dental Pulp Cells	2018	Journal of Endodontics
Lee W.; Oh J.-H.; Park J.-C.; Shin H.-I.; Baek J.-H.; Ryoo H.-M.; Woo K.M.	Performance of electrospun poly(-caprolactone) fiber meshes used with mineral trioxide aggregates in a pulp capping procedure	2012	Acta Biomaterialia
Rechenberg D.-K.; Bostanci N.; Zehnder M.; Belibasakis G.N.	Periapical fluid RANKL and IL-8 are differentially regulated in pulpitis and apical periodontitis	2014	Cytokine
Asgary S, Nosrat A, Homayounfar N.	Periapical healing after direct pulp capping with calcium-enriched mixture cement: a case report	2012	Oper Dent
Jersa I.; Kundzina R.	Periapical status and quality of root fillings in a selected adult Riga population.	2013	Stomatologija / issued by public institution "Odontologijos studija" ... [et al.
Duque T.M.; Prado M.; Herrera D.R.; Gomes B.P.F.A.	Periodontal and endodontic infectious/inflammatory profile in primary periodontal lesions with secondary endodontic involvement after a calcium hydroxide-based intracanal medication	2019	Clinical Oral Investigations
Cen R., Wang R., Cheung G.S.P.	Periodontal Blood Flow Protects the Alveolar Bone from Thermal Injury during Thermoplasticized Obturation: A Finite Element Analysis Study	2018	Journal of endodontics
Fontes TV, Ferreira SM, Silva-Júnior A, Dos Santos Marotta P, Noce CW, Ferreira Dde C, Gonçalves LS.	Periradicular lesions in HIV-infected patients attending the faculty of dentistry: clinical findings, socio-demographics status, habits and laboratory data - seeking an association	2014	Clinics (Sao Paulo)
Pang YW, Feng J, Daltoe F, Fatscher R, Gentleman E, Gentleman MM, Sharpe PT.	Perivascular Stem Cells at the Tip of Mouse Incisors Regulate Tissue Regeneration	2016	J Bone Miner Res
Kunert G.G.; Kunert I.R.; Da Costa Filho L.C.; De Figueiredo J.A.P.	Permanent teeth pulpotomy survival analysis: Retrospective follow-up	2015	Journal of Dentistry
Meza G.; Urrejola D.; Saint Jean N.; Inostroza C.; López V.; Khoury M.; Brizuela C.	Personalized Cell Therapy for Pulpitis Using Autologous Dental Pulp Stem Cells and Leukocyte Platelet-rich Fibrin: A Case Report	2019	Journal of Endodontics
Shlezinger M.; Khalifa L.; Houri-Haddad Y.; Coppenhagen-Glazer S.; Resch G.; Que Y.-A.; Beyth S.; Dorfman E.; Hazan R.; Beyth N.	Phage therapy: A new horizon in the antibacterial treatment of oral pathogens	2017	Current Topics in Medicinal Chemistry

Tabuchi H.; Katsurabara T.; Mori M.; Aoyama M.; Obara T.; Yasuda N.; Kawano T.; Imai T.; Ieiri I.; Kumagai Y.	Pharmacokinetics, Pharmacodynamics, and Safety of E6011, a Novel Humanized Antifractalkine (CX3CL1) Monoclonal Antibody: A Randomized, Double-Blind, Placebo-Controlled Single-Ascending-Dose Study	2019	Journal of Clinical Pharmacology
Hartiala P.; Suominen S.; Suominen E.; Kaartinen I.; Kiiski J.; Viitanen T.; Alitalo K.; Saarikko A.M.	Phase 1 Lymfactin® Study: Short-term Safety of Combined Adenoviral VEGF-C and Lymph Node Transfer Treatment for Upper Extremity Lymphedema	2020	Journal of Plastic, Reconstructive and Aesthetic Surgery
Lima AR, Ganguly T, Walker AR, Acosta N, Francisco PA, Pileggi R, Lemos JA, Gomes BPFA, Abranches J.	Phenotypic and Genotypic Characterization of Streptococcus mutans Strains Isolated from Endodontic Infections	2020	J Endod
Nutchoey O, Intarak N, Theerapanon T, Thaweesapphithak S, Boonprakong L, Srijunbarl A, Porntaveetus T, Shotelersuk V.	Phenotypic features of dentinogenesis imperfecta associated with osteogenesis imperfecta and COL1A2 mutations	2021	Oral Surg Oral Med Oral Pathol Oral Radiol
Sun G.; Ren Q.; Bai L.; Zhang L.	Phoenixin-20 suppresses lipopolysaccharide-induced inflammation in dental pulp cells	2020	Chemico-Biological Interactions
Vitor L.L.R.; Bergamo M.T.O.P.; Lourenço-Neto N.; Sakai V.T.; Oliveira R.C.; Cruvinel T.; Rios D.; Garlet G.P.; Santos C.F.; Machado M.A.A.M.; Oliveira T.M.	Photobiomodulation effect on angiogenic proteins produced and released by dental pulp cells	2020	Clinical Oral Investigations
Zaccara I.M.; Mestieri L.B.; Pilar E.F.S.; Moreira M.S.; Grecca F.S.; Martins M.D.; Kopper P.M.P.	Photobiomodulation therapy improves human dental pulp stem cell viability and migration in vitro associated to upregulation of histone acetylation	2020	Lasers in Medical Science
Zaccara I.M.; Mestieri L.B.; Moreira M.S.; Grecca F.S.; Martins M.D.; Kopper P.M.P.	Photobiomodulation therapy improves multilineage differentiation of dental pulp stem cells in three-dimensional culture model	2018	Journal of Biomedical Optics
Sivakumar T.T.; Muruppel A.M.; Joseph A.P.; Reshmi A.; Ramachandran R.; Nair P.D.; Mohan S.P.	Photobiomodulatory effect delivered by low-level laser on dental pulp stem cell differentiation for osteogenic lineage	2019	Lasers in Dental Science
Okamoto C.B., Bussadori S.K., Prates R.A., da Mota A.C.C., Tempestini Horliana A.C.R., Fernandes K.P.S., Motta L.J.	Photodynamic therapy for endodontic treatment of primary teeth: A randomized controlled clinical trial	2020	Photodiagnosis and Photodynamic Therapy
Zorita-García M.; Alonso-Ezpeleta L.Ó.; Cobo M.; del Campo R.; Rico-Romano C.; Mena-Álvarez J.; Zubizarreta-Macho Á.	Photodynamic therapy in endodontic root canal treatment significantly increases bacterial clearance, preventing apical periodontitis	2019	Quintessence International
Libório A.L.M.; Viana B.A.S.; Endo M.S.; Pavan N.N.O.	Photodynamic therapy in the control of endodontic infections	2018	Dental Press Endodontics
Muhammad O.H.; Chevalier M.; Rocca J.-P.; Brulat-Bouchard N.; Medioni E.	Photodynamic therapy versus ultrasonic irrigation: Interaction with endodontic microbial biofilm, an ex vivo study	2014	Photodiagnosis and Photodynamic Therapy
Moreira M.S.; Sarra G.; Carvalho G.L.; Gonçalves F.; Caballero-Flores H.V.; Pedroni A.C.F.; Lascala C.A.; Catalani L.H.; Marques M.M.	Physical and Biological Properties of a Chitosan Hydrogel Scaffold Associated to Photobiomodulation Therapy for Dental Pulp Regeneration: An in Vitro and in Vivo Study	2021	BioMed Research International
Abdalla M.M.; Lung C.Y.K.; Bijle M.N.; Yiu C.K.Y.	Physicochemical Properties and Inductive Effect of Calcium Strontium Silicate on the Differentiation of Human Dental Pulp Stem Cells for Vital Pulp Therapies: An In Vitro Study	2022	Materials
Krishnapriya V, Gaur D, Kumar CS, Shilpa G.	Physiological Root End Closure in a Traumatized Young Permanent Tooth Using Collagen Particles as Pulpal Dressing	2017	J Clin Diagn Res
Nugroho J.J.; Trilaksana A.C.; Rovani C.A.; Natsir N.; Rahim F.	Pizza Technique in Site 1 restoration in lower Second Molar Tooth: A case report	2019	Journal of International Dental and Medical Research
Gasqui MA, Pérard M, Decup F, Monsarrat P, Turpin YL, Villat C, Gueyffier F, Maucort-Boulch D, Roche L, Grosgogeat B.	Place of a new radiological index in predicting pulp exposure before intervention for deep carious lesions	2022	Oral Radiol
Machut K., Zoltowska A., Pawlowska E., Derwich M.	Plasma rich in growth factors in the treatment of endodontic periapical lesions in adult patients: Case reports	2021	International Journal of Molecular Sciences
Da Cunha J.M.; Da Costa-Neves A.; Kerkis I.; Da Silva M.C.P.	Pluripotent stem cell transcription factors during human odontogenesis	2013	Cell and Tissue Research
Gao L.; Fan F.; Wang L.; Tang B.; Wen Z.; Tang J.; Dai T.; Jin H.	Polarization of macrophages in the trigeminal ganglion of rats with pulpitis	2022	Journal of Oral Rehabilitation

Leite M.L.; de Oliveira Ribeiro R.A.; Soares D.G.; Hebling J.; de Souza Costa C.A.	Poly(caprolactone)-aligned nanofibers associated with fibronectin-loaded collagen hydrogel as a potent bioactive scaffold for cell-free regenerative endodontics	2022	International Endodontic Journal
Trilisinskaya Y.; Smrekova E.; Komlosi M.; Ondova P.; Svobodova H.; Voinescu B.	Poor dental health resulting to dental infections among South Sudanese refugees	2016	Lekarsky Obzor
Maroto M.; Barreiro S.; Barbería E.	Portland cement as pulp dressing agent in pulpotomy treatment of primary molars: A 12-month clinical study	2019	European Journal of Paediatric Dentistry
Weber M.-T.; Stratz N.; Fleiner J.; Schulze D.; Hannig C.	Possibilities and limits of imaging endodontic structures with CBCT	2015	Swiss dental journal
Popescu A.D., Popa D.L., Nicola A.G., Dascălu I.T., Petcu C., Tircă T., Tuculina M.J., Mocanu H., Staicu A.N., Gheorghiță L.M.	Post Placement and Restoration of Endodontically Treated Canines: A Finite Element Analysis Study	2022	International Journal of Environmental Research and Public Health
El Sayed M., Gaballah K.	Postanesthetic Cold Sensibility Test as an Indicator for the Efficacy of Inferior Alveolar Nerve Block in Patients with Symptomatic Irreversible Pulpitis of Mandibular Molars	2021	International Journal of Dentistry
Eghbal M.J., Haeri A., Shahravan A., Kazemi A., Moazami F., Mozayeni M.A., Saberi E., Samiei M., Vatanpour M., Akbarzade Baghban A., Fazlyab M., Parhizkar A., Ahmadi M., Akbarian Rad N., Bijari S., Bineshmarvasti D., Davoudi P., Dehghan R., Dehghani M., Ebrahimi H., Emami N., Farajian N., Fereidooni R., Ghobadi G., Ghodrati M., Gohari A., Hashemi A., Hosseini M., Karami E., Kheirabadi N., Kozegari S., Labaf Ghasemi H., Majidi A., Malekzadeh P., Mehrabi V., Mohammadi M., Moradi Eslami L., Noghani A., Omatali N., Pourhatami N., Rahbani Nobar B., Rahmani S., Shafaq P., Soofiabadi S., Teimoori S., Vatandoost F., Asgary S.	Postendodontic Pain after Pulpotomy or Root Canal Treatment in Mature Teeth with Carious Pulp Exposure: A Multicenter Randomized Controlled Trial	2020	Pain Research and Management
Saumya-Rajesh P.; Krithikadatta J.; Velmurugan N.; Sooriaprakas C.	Post-instrumentation pain after the use of either Mtwo or the SAF system: a randomized controlled clinical trial	2017	International Endodontic Journal
Zhang Q, Deng X, Wang Y, Huang R, Yang R, Zou J.	Postoperative complications in Chinese children following dental general anesthesia: A cross-sectional study	2020	Medicine (Baltimore)
Pasqualini D.; Mollo L.; Scotti N.; Cantatore G.; Castellucci A.; Migliaretti G.; Berutti E.	Postoperative pain after manual and mechanical glide path: A Randomized clinical trial	2012	Journal of Endodontics
Erkan E.; Gündoğar M.; Uslu G.; Özyürek T.	Postoperative pain after SWEEPS, PIPS, sonic and ultrasonic-assisted irrigation activation techniques: a randomized clinical trial	2022	Odontology
Morsy D.A., Negm M., Diab A., Ahmed G.	Postoperative pain and antibacterial effect of 980 nm diode laser versus conventional endodontic treatment in necrotic teeth with chronic periapical lesions: A randomized control trial.	2018	F1000Research
Shafie L.; Barghi H.; Parirokh M.; Ebrahimnejad H.; Nakhae N.; Esmaili S.	Postoperative pain following pulpotomy of primary molars with two biomaterials: A randomized split mouth clinical trial	2017	Iranian Endodontic Journal
Kumar A., Jain G., Sharma S., Mishra R., Dhawan J.	Potency Of Manual Instrumentation To The Rotary Instrumentation For Primary Teeth Pulpectomies: A Clinical Comparrative Study	2022	Journal of Pharmaceutical Negative Results
Zhai Y.; Yuan X.; Zhao Y.; Ge L.; Wang Y.	Potential Application of Human β-Defensin 4 in Dental Pulp Repair	2020	Frontiers in Physiology
Chen Y.-J.; Zhao Y.-H.; Zhao Y.-J.; Liu N.-X.; Lv X.; Li Q.; Chen F.-M.; Zhang M.	Potential dental pulp revascularization and odonto-/osteogenic capacity of a novel transplant combined with dental pulp stem cells and platelet-rich fibrin	2015	Cell and Tissue Research
Irusa K.F., Vence B., Donovan T.	Potential oral health effects of e-cigarettes and vaping: A review and case reports	2020	Journal of Esthetic and Restorative Dentistry
Arora S., Cooper P.R., Friedlander L.T., Seo B., Rizwan S.B., Rich A.M., Hussaini H.M.	Potentiality and Inflammatory Marker Expression Are Maintained in Dental Pulp Cell Cultures from Carious Teeth	2022	International Journal of Molecular Sciences

Haywood V.B.	Pre-bleaching exam vital for optimum whitening.	2012	Compendium of continuing education in dentistry (Jamesburg, N.J. : 1995)
Jiang K.; Che C.; Ding Z.; Zeng S.; Wang W.; He X.	Precision diagnosis and antidiastole on supernumerary cusp of tooth by CBCT	2016	Surgical and Radiologic Anatomy
Chevalier V, Dessert M, Fouillen KJ, Lennon S, Duncan HF.	Preclinical 3D-printed laboratory simulation of deep caries and the exposed pulp reduced student anxiety and stress, while increasing confidence and knowledge in vital pulp treatment	2022	Int Endod J
Gomes M.S.; Böttcher D.E.; Scarparo R.K.; Morgental R.D.; Waltrick S.B.G.; Ghisi A.C.; Rahde N.M.; Borba M.G.; Blomberg L.C.; Figueiredo J.A.P.	Predicting pre- and postoperative pain of endodontic origin in a southern Brazilian subpopulation: an electronic database study	2017	International Endodontic Journal
Law A.S.; Nixdorf D.R.; Aguirre A.M.; Reams G.J.; Tortomasi A.J.; Manne B.D.; Harris D.R.	Predicting severe pain after root canal therapy in the national dental PBRN	2015	Journal of Dental Research
Galicia J.C.; Guzzi P.H.; Giorgi F.M.; Khan A.A.	Predicting the response of the dental pulp to SARS-CoV2 infection: a transcriptome-wide effect cross-analysis	2020	Genes and Immunity
Villa-Chávez C.E.; Patiño-Marín N.; Loyola-Rodríguez J.P.; Zavala-Alonso N.V.; Martínez-Castañón G.A.; Medina-Solís C.E.	Predictive values of thermal and electrical dental pulp tests: A clinical study	2013	Journal of Endodontics
Song M, Alshaikh A, Kim T, Kim S, Dang M, Mehrazarin S, Shin KH, Kang M, Park NH, Kim RH.	Preexisting Periapical Inflammatory Condition Exacerbates Tooth Extraction-induced Bisphosphonate-related Osteonecrosis of the Jaw Lesions in Mice	2016	J Endod
Casián-Adem J.; Cobos L.; Waggoner W.F.; Fuks A.B.	Prefabricated zirconia crowns – A solution to treat hypomineralized permanent molars: Report of a case	2021	Journal of Clinical Pediatric Dentistry
Ali A.H.; Mahdee A.F.; Fadhil N.H.	Preferences of treatments and materials used in the management of exPosed PulPs: a web-based questionnaire study	2022	Journal of Stomatology
Mirza A.J., Taqi M., Hatipoglo O., Javaid M.A., Moosa R.	Preferred Technique and Material Choice for exposed pulp among dentists practicing in Karachi	2022	Pakistan Journal of Medical and Health Sciences
Innes NP, Ricketts D, Chong LY, Keightley AJ, Lamont T, Santamaria RM.	Preformed crowns for decayed primary molar teeth	2015	Cochrane Database Syst Rev
Wang Q.L.; Yang P.P.; Ge L.H.; Liu H.	Preliminary Evaluation of Platelet Rich Fibrin-Mediated Tissue Repair in Immature Canine Pulpless Teeth	2016	The Chinese journal of dental research : the official journal of the Scientific Section of the Chinese Stomatological Association (CSA)
Komabayashi T.; Wadajkar A.; Santimano S.; Ahn C.; Zhu Q.; Opperman L.A.; Bellinger L.L.; Yang J.; Nguyen K.T.	Preliminary study of light-cured hydrogel for endodontic drug delivery vehicle	2016	Journal of investigative and clinical dentistry
Nivedha V.; Sherwood I.A.; Abbott P.V.; Ramaprabha B.; Bhargavi P.V.	Pre-operative ketorolac efficacy with different anesthetics, irrigants during single visit root canal treatment of mandibular molars with acute irreversible pulpitis	2020	Australian Endodontic Journal
Alqarni M.A.	Preparation of dental implant site by interim endodontic therapy: A case report	2020	European Journal of Molecular and Clinical Medicine
Rutkauskas J.; Seale N.S.; Casamassimo P.; Rutkauskas J.S.	Preparedness of Entering Pediatric Dentistry Residents: Advanced Pediatric Program Directors' and First-Year Residents' Perspectives	2015	Journal of Dental Education
Alattas H.A.; Alyami S.H.	Prescription of antibiotics for pulpal and periapical pathology among dentists in southern Saudi Arabia	2017	Journal of Global Antimicrobial Resistance
Fraser A.D., Zhang B., Khan H., Ma H., Hersh E.V.	Prescription opioid abuse and its potential role in gross dental decay	2016	Current Drug Safety
Suhaib M., Ahmad F., Ahmad M.	Prescription pattern of antimicrobial agents among dental practitioners in a tertiary care center in North India	2017	Asian Journal of Pharmaceutical and Clinical Research

Keleş A., Keskin C.	Presence of voids after warm vertical compaction and single-cone obturation in band-shaped isthmuses using micro-computed tomography: A phantom study	2020	Microscopy research and technique
Connert T, Weiger R, Krastl G.	Present status and future directions - Guided endodontics	2022	Int Endod J
Duncan HF.	Present status and future directions-Vital pulp treatment and pulp preservation strategies	2022	Int Endod J
Aboujaoude S.; El Noueiri B.	Presentations of Routine Dental Interventions in Children over a Six-Year Period	2022	Scientific World Journal
Villavicencio Espinoza CA, Ordoñez Aguilera JF, Piola Rizzante FA, Maenosono RM, Franco EB, Ishikiriama SK.	Preservation in Young Patients of Pulp Vitality and Anatomical-Functional Restoration of the Lost Structure	2017	Compend Contin Educ Dent
Basso M., Van Duinen R.N.B., Miletic I.	Preservation of dental pulp vitality: A fundamental goal for modern conservative dentistry	2018	Dental Cadmos
Bozbay E.; Dominici F.; Gokbuget A.Y.; Cintan S.; Guida L.; Aydin M.S.; Mariotti A.; Pilloni A.	Preservation of root cementum: a comparative evaluation of power-driven versus hand instruments	2018	International Journal of Dental Hygiene
Edwards D, Stone S, Bailey O, Tomson P.	Preserving pulp vitality: part one - strategies for managing deep caries in permanent teeth	2021	Br Dent J
Edwards D.; Stone S.; Bailey O.; Tomson P.	Preserving pulp vitality: part two - vital pulp therapies	2021	British Dental Journal
Choi MR, Moon YM, Seo MS.	Prevalence and features of distolingual roots in mandibular molars analyzed by cone-beam computed tomography	2015	Imaging Sci Dent
Igic M, Obradovic R, Filipovic G.	Prevalence and progression of early childhood caries in Nis, Serbia	2018	Eur J Paediatr Dent
Dash G., Mishra L., Singh N.R., Behera R., Misra S.R., Kumar M., Sokolowski K., Agarwal K., Behera S.K., Mishra S., Lapinska B.	Prevalence and Quality of Endodontic Treatment in Patients with Cardiovascular Disease and Associated Risk Factors	2022	Journal of Clinical Medicine
Vaishnavi Devi. B.; Delphine Priscilla Antony S.	Prevalence of canal variations in mandibular 1st molar in south indian population and association with age and gender	2020	International Journal of Dentistry and Oral Science
De la Torre-Luna R.; Domínguez-Pérez R.A.; Guillén-Nepita A.L.; Ayala-Herrera J.L.; Martínez-Martínez R.E.; Romero-Ayala M.E.; Pérez-Serrano R.M.; Vázquez-Garcidueñas M.S.	Prevalence of Candida albicans in primary endodontic infections associated with a higher frequency of apical periodontitis in type two diabetes mellitus patients	2020	European Journal of Clinical Microbiology and Infectious Diseases
Mubaraki S, AlOlyan R, AlBrekeit J, AlFouzan S, Abosharkh M, AlSaeri N, Baseer MA.	Prevalence of caries in first permanent molar among children in Saudi Arabia: a retrospective study	2022	Eur Rev Med Pharmacol Sci
Ghaffar A., Ahmed Z., Munir M., Saeed A., Parveen N., Rehman S.	Prevalence of complicated and uncomplicated crown fracture in permanent teeth children age 7-12years	2021	Pakistan Journal of Medical and Health Sciences
Naziazareen I., Gheena S., Leelavathi L.	Prevalence of dental caries among outpatients attending a dental college in Chennai	2019	Drug Invention Today
de Alencar Fernandes J.M.F.; de Oliveira Paredes S.; Bezerra de Almeida D.; Correia Sampaio F.; Soares Forte F.D.	Prevalence of dental caries and treatment needs in preschool children in a recently fluoridated Brazilian town	2013	Brazilian Journal of Oral Sciences
Keshaav Krishnaa P., Nasim I., Sandeep A.H.	Prevalence of different reasons for a tooth to undergo root canal retreatment	2020	International Journal of Research in Pharmaceutical Sciences
Sousa H.C.S.; de Lima M.D.M.; Lima C.C.B.; de Moura M.S.; Bandeira A.V.L.; de Deus Moura L.F.A.	Prevalence of enamel defects in premolars whose predecessors were treated with extractions or antibiotic paste	2020	Oral Health and Preventive Dentistry
de Oliveira B.P.; Câmara A.C.; Aguiar C.M.	Prevalence of endodontic diseases: An epidemiological evaluation in a Brazilian subpopulation	2016	Brazilian Journal of Oral Sciences
Stojanović N.; Krunić J.; Popović B.; Stojičić S.; Živković S.	Prevalence of Enterococcus faecalis and Porphyromonas gingivalis in infected root canals and their susceptibility to endodontic treatment procedures: A molecular study	2014	Srpski Arhiv za Celokupno Lekarstvo
Ilangovan S., Ravindran V., Mani G.	Prevalence of gropers appliance in pediatric dentistry	2020	International Journal of Research in Pharmaceutical Sciences
Nivethitha R., Ganapathy D., Dinesh S.P.S.	Prevalence of hypertension in completely edentulous patients	2020	Indian Journal of Forensic Medicine and Toxicology

Gabor C.; Tam E.; Shen Y.; Haapasalo M.	Prevalence of internal inflammatory root resorption	2012	Journal of Endodontics
Dalabilia V.; Gambin D.J.; De-Carli J.P.; Zanette F.	Prevalence of odontogenic pain in a public dental urgency service in Southern Brazil	2020	Dental Press Endodontics
Baig M.S., Zahid M., Shaheen J.A., Bhutto R.A.	Prevalence of odontogenic sinus tracts among dental patients and its oral surgery management at Bahawal Victoria Hospital & Quaid-e-Azam Medical College Bahawalpur	2016	Pakistan Journal of Medical and Health Sciences
Silva E.J.N.L., Prado M.C., Duarte M.A.H., Versiani M.A., Marques D., Martins J.N.R.	Prevalence of root canal system configurations in the brazilian population analyzed by cone-beam computed tomography - a systematic review	2021	Revista Portuguesa de Estomatologia, Medicina Dentaria e Cirurgia Maxilofacial
Khosrozadeh M., Mostafavi M., Hamrah M.H., Niknejad E.	Prevalence of Three-Rooted Deciduous Mandibular Molars in the Children of Northwestern Iran	2021	International Journal of Dentistry
Gopal T.M.; Subhashree R.	Prevalence of vitality in fixed partial denture done by the undergraduates - A retrospective study	2020	Indian Journal of Forensic Medicine and Toxicology
Martins J.N.R., Marques D., Silva E.J.N.L., Caramês J., Versiani M.A.	Prevalence Studies on Root Canal Anatomy Using Cone-beam Computed Tomographic Imaging: A Systematic Review	2019	Journal of endodontics
Dube K, Jain P, Rai A, Paul B.	Preventive endodontics by direct pulp capping with restorative dentin substitute-biodentine: A series of fifteen cases	2018	Indian J Dent Res
Kranz A.M., Rozier R.G., Preisser J.S., Stearns S.C., Weinberger M., Lee J.Y.	Preventive Services by Medical and Dental Providers and Treatment Outcomes	2014	Journal of dental research
de Lima BR, Nicoloso GF, Fatturi-Parolo CC, Ferreira MBC, Montagner F, Casagrande L.	Prevotella strains and lactamic resistance gene distribution in different oral environments of children with pulp necrosis	2018	Int Endod J
Fatahzadeh M.	Primary diffuse large B-cell lymphoma of mandible masquerading as a toothache	2020	Quintessence International
Atasever G, Keceli TI, Uysal S, Gungor HC, Olmez S.	Primary molar pulpotomies with different hemorrhage control agents and base materials: A randomized clinical trial	2019	Niger J Clin Pract
Coll JA, Seale NS, Vargas K, Marghalani AA, Al Shamali S, Graham L.	Primary Tooth Vital Pulp Therapy: A Systematic Review and Meta-analysis	2017	Pediatr Dent
Cannon M.; Gerodias N.; Viera A.; Percinoto C.; Jurado R.	Primate pulpal healing after exposure and TheraCal application	2014	The Journal of clinical pediatric dentistry
Nagashima K.; Miwa T.; Soumiya H.; Ushiro D.; Takeda-Kawaguchi T.; Tamaoki N.; Ishiguro S.; Sato Y.; Miyamoto K.; Ohno T.; Osawa M.; Kunisada T.; Shibata T.; Tezuka K.-I.; Furukawa S.; Fukumitsu H.	Priming with FGF2 stimulates human dental pulp cells to promote axonal regeneration and locomotor function recovery after spinal cord injury	2017	Scientific Reports
Pirani C.; Tinarelli V.; Gatto M.R.; Iacono F.; Gandolfi M.G.; Prati C.	Prognosis of root canal treatments filled with Thermafil system: A 5-year retrospective study	2016	Giornale Italiano di Endodonzia
Kim C.H.; Bae J.S.; Kim I.-H.; Song J.S.; Choi H.-J.; Kang C.-M.	Prognostic factors for the survival of primary molars following pulpotomy with mineral trioxide aggregate: a retrospective cohort study	2021	Clinical Oral Investigations
Dutra T.T.B.; Bezerra T.M.M.; Chaves F.N.; Feitosa S.G.; Costa F.W.G.; Pereira K.M.A.	Progressive ossificans fibrodysplasia endodontic management: Case report	2019	Special Care in Dentistry
Likitpongpipat N, Sangmaneedet S, Klanrit P, Noisombut R, Krisanaprakornkit S, Chailertvanitkul P.	Promotion of Dental Pulp Wound Healing in New Zealand White Rabbits' Teeth by Thai Propolis Product	2019	J Vet Dent
Likitpongpipat N.; Sangmaneedet S.; Klanrit P.; Noisombut R.; Krisanaprakornkit S.; Chailertvanitkul P.	Promotion of Dental Pulp Wound Healing in New Zealand White Rabbits’ Teeth by Thai Propolis Product	2019	Journal of Veterinary Dentistry
Gandolfi M.G.; Siboni F.; Prati C.	Properties of a novel polysiloxane-guttapercha calcium silicate-bioglass-containing root canal sealer	2016	Dental Materials
Patel V.; Gadiwalla Y.; Sassoon I.; Sproat C.; Kwok J.; McGurk M.	Prophylactic use of pentoxifylline and tocopherol in patients who require dental extractions after radiotherapy for cancer of the head and neck	2016	British Journal of Oral and Maxillofacial Surgery
Petrini M.; Ferrante M.; Ciavarelli L.; Ceccarini A.; Brunetti L.; Vacca M.; Spoto G.	Prostaglandin E2to diagnose between reversible and irreversible pulpitis	2012	International Journal of Immunopathology and Pharmacology

Ohkura N.; Shigetani Y.; Yoshiba N.; Yoshiba K.; Okiji T.	Prostaglandin transporting protein-mediated prostaglandin E2 transport in lipopolysaccharide-inflamed rat dental pulp	2014	Journal of Endodontics
Braga V.S., Kramer P.F., Ceccato C.J., Ferreira S.H.	Prosthetic Rehabilitation of a Patient with Severe Early Childhood Caries: A Case Report	2020	Journal of Clinical and Diagnostic Research
Türp L., Kern M., Chaar M.S.	Prosthetic restoration after atraumatic surgical extrusion: a report of two cases	2022	Quintessence International
Feng Q.; Sun X.; Ren Q.; Liu J.	Protective effect of SIRT6 against LPS-induced human dental pulp cell apoptosis via regulating Ku70 deacetylation	2017	International Journal of Clinical and Experimental Pathology
Zhang L.; Bai L.; Ren Q.; Sun G.; Si Y.	Protective effects of SIRT6 against lipopolysaccharide (LPS) are mediated by deacetylation of Ku70	2018	Molecular Immunology
Silva P.A.O.; Lima S.M.F.; Freire M.S.; Murad A.M.; Franco O.L.; Rezende T.M.B.	Proteomic analysis of human dental pulp in different clinical diagnosis	2021	Clinical Oral Investigations
Eckhardt A.; Jágr M.; Pataridis S.; Mikšík I.	Proteomic analysis of human tooth pulp: Proteomics of human tooth	2014	Journal of Endodontics
Coll J, Seale NS, Vargas K, Chi DL, Marghalani AA, Graham L.	Protocol for a Systematic Review and Meta-analysis of Vital Pulp Therapy for Children with Deep Caries in the Primary Dentition	2015	Pediatr Dent
Kumar R.A., Venkatesh B., Karumaran C.S., Rajasekaran M.S., Shankar P.	Protocol for dental management in a patient with glucose-6-phosphate dehydrogenase deficiency	2017	Journal of Clinical and Diagnostic Research
Sonoda S.; Yamaza H.; Yoshimaru K.; Taguchi T.; Yamaza T.	Protocol to generate xenogeneic-free/serum-free human dental pulp stem cells	2022	STAR Protocols
Boderé PJ, Calberson F, De Bruyne M, De Moor R, Meire M.	Protocols for cleaning the incisor access cavity contaminated with epoxy resin sealer	2022	Eur J Oral Sci
St Paul A.; Phillips C.; Lee J.Y.; Khan A.A.	Provider Perceptions of Treatment Options for Immature Permanent Teeth	2017	Journal of Endodontics
Hashim Nainar SM.	Provision of Pulp Therapy and Stainless Steel Crowns for Young Children by General Dentists	2016	Pediatr Dent
Ramnarain P.; Singh S.	Public healthcare practitioners' knowledge, attitudes and practices related to oral antibiotic prescriptions for dental use in Pietermaritzburg, KwaZulu-Natal	2022	Health SA Gesondheid
Ricucci D, Siqueira JF Jr, Loghin S, Lin LM.	Pulp and apical tissue response to deep caries in immature teeth: A histologic and histobacteriologic study	2017	J Dent
Ricucci D., Siqueira J.F., Rôças I.N., Lipski M., Shiban A., Tay F.R.	Pulp and dentine responses to selective caries excavation: A histological and histobacteriological human study	2020	Journal of dentistry
Chalmers N.I., Oh K., Hughes C.V., Pradhan N., Kanasi E., Ehrlich Y., Dewhirst F.E., Tanner A.C.R.	Pulp and plaque microbiotas of children with severe early childhood caries	2015	Journal of Oral Microbiology
Aaminabadi N.A.; Parto M.; Emamverdizadeh P.; Jamali Z.; Shirazi S.	Pulp bleeding color is an indicator of clinical and histohematologic status of primary teeth	2017	Clinical Oral Investigations
de Sousa Reis M.; Scarparo R.K.; Signor B.; Bolzan J.T.; Steier L.; de Figueiredo J.A.P.	Pulp capping with mineral trioxide aggregate or Biodentine: a comparison of mineralized barrier formation and inflammatory and degenerative events	2021	Brazilian Oral Research
Pedroza J.A.Y., Villasante R.A.Ñ., Kohatsu A.K.N., Gamboa M.M.R.N.	Pulp capping: Calcium silicate-based cements. Report of a clinical case	2020	Revista Estomatologica Herediana
Souron J.-B.; Petiet A.; Decup F.; Tran X.V.; Lesieur J.; Poliard A.; Le Guludec D.; Letourneur D.; Chaussain C.; Rouzet F.; Vital S.O.	Pulp cell tracking by radionuclide imaging for dental tissue engineering	2014	Tissue Engineering - Part C: Methods
Vilca Velazco M.F., Lavado García L., Torres Ramos G., López Ramos R.P.	Pulp chamber access alternative in pulpectomies in a child with Wolff-Parkinson-White syndrome. A case report	2022	Special care in dentistry : official publication of the American Association of Hospital Dentists, the Academy of Dentistry for the Handicapped, and the American Society for Geriatric Dentistry
Frisk F.; Kvist T.; Axelsson S.; Bergenholtz G.; Davidson T.; Mejare I.; Norlund A.; Petersson A.; Sandberg H.; Tranæus S.; Hakeberg M.	Pulp exposures in adults - Choice of treatment among Swedish dentists	2013	Swedish Dental Journal

Murano H.; Kaneko T.; Zaw S.Y.M.; Sone P.P.; Zaw Z.C.T.; Okada Y.; Sunakawa M.; Katsube K.-I.; Okiji T.	Pulp inflammation induces Kv1.1 K+ channel down-regulation in rat thalamus	2022	Oral Diseases
Duncan H.F.; Cooper P.R.	Pulp Innate Immune Defense: Translational Opportunities	2020	Journal of Endodontics
Stafuzza TC, Vitor LLR, Lourenço Neto N, Rios D, Cruvinel T, Sakai VT, Moretti ABS, Machado MAAM, Oliveira TM.	Pulp liner materials in selective caries removal: study protocol for a randomised controlled trial	2021	BMJ Open
Martens L.; Rajasekharan S.; Cauwels R.	Pulp management after traumatic injuries with a tricalcium silicate-based cement (Biodentine™): a report of two cases, up to 48 months follow-up	2015	European Archives of Paediatric Dentistry
Jabbar N.S.A.; Aldrigui J.M.; Braga M.M.; Wanderley M.T.	Pulp polyp in traumatized primary teeth - A case-control study	2013	Dental Traumatology
Wang G.; Wang C.; Qin M.	Pulp prognosis following conservative pulp treatment in teeth with complicated crown fractures—A retrospective study	2017	Dental Traumatology
Itoh Y.; Sasaki J.I.; Hashimoto M.; Katata C.; Hayashi M.; Imazato S.	Pulp Regeneration by 3-dimensional Dental Pulp Stem Cell Constructs	2018	Journal of Dental Research
Nakashima M.; Iohara K.; Murakami M.; Nakamura H.; Sato Y.; Ariji Y.; Matsushita K.	Pulp regeneration by transplantation of dental pulp stem cells in pulpitis: a pilot clinical study	2017	Stem Cell Research and Therapy
Jang J.-H.; Moon J.-H.; Kim S.G.; Kim S.-Y.	Pulp regeneration with hemostatic matrices as a scaffold in an immature tooth minipig model	2020	Scientific Reports
Nakashima M.; Iohara K.; Zayed M.	Pulp Regeneration: Current Approaches, Challenges, and Novel Rejuvenating Strategies for an Aging Population	2020	Journal of Endodontics
Nakashima M.; Fukuyama F.; Iohara K.	Pulp Regenerative Cell Therapy for Mature Molars: A Report of 2 Cases	2022	Journal of Endodontics
Machado M.A.A.M., Stafuzza T.C., Vitor L.L.R., da Costa S.A., da Costa S.M., Neto N.L., Oliveira T.M.	Pulp repair response after the use of a dentin-pulp biostimulation membrane (BBio) in primary teeth: study protocol for a randomized clinical trial	2020	Trials
de Oliveira Gallinari M.; Ângelo Cintra L.T.; Benetti F.; Rahal V.; Ervolino E.; Fraga Briso A.L.	Pulp response of rats submitted to bleaching and the use of different anti-inflammatory drugs	2019	PLoS ONE
Ricucci D.; Siqueira J.F., Jr.; Rôças I.N.	Pulp Response to Periodontal Disease: Novel Observations Help Clarify the Processes of Tissue Breakdown and Infection	2021	Journal of Endodontics
Dimitrova-Nakov S.; Baudry A.; Harichane Y.; Kellermann O.; Goldberg M.	Pulp stem cells: Implication in reparative dentin formation	2014	Journal of Endodontics
Sato T, Matsuyama Y, Fujiwara T, Tagami J.	Pulp survival after composite resin restoration of caries lesions in adults	2020	J Oral Sci
Shivanni S.S.; Bhagya Lakshmi T.; Balakrishna R.N.	Pulp therapies in young permanent maxillary molar with class I caries	2019	International Journal of Dentistry and Oral Science
Shah A.; Peacock R.; Eliyas S.	Pulp therapy and root canal treatment techniques in immature permanent teeth: an update	2022	British Dental Journal
	Pulp therapy for primary and immature permanent teeth	2018	Pediatric Dentistry
Kratunova E, Silva D.	Pulp therapy for primary and immature permanent teeth: an overview	2018	Gen Dent
Marques N.; Lourenço Neto N.; Fernandes A.P.; Rodini C.; Hungaro Duarte M.; Rios D.; Machado M.A.; Oliveira T.	Pulp tissue response to Portland cement associated with different radio pacifying agents on pulpotomy of human primary molars	2015	Journal of Microscopy
Smaïl-Faugeron V, Courson F, Durieux P, Muller-Bolla M, Glenny AM, Fron Chabouis H.	Pulp treatment for extensive decay in primary teeth	2014	Cochrane Database Syst Rev
Han G.; Hu M.; Zhang Y.; Jiang H.	Pulp vitality and histologic changes in human dental pulp after the application of moderate and severe intrusive orthodontic forces	2013	American Journal of Orthodontics and Dentofacial Orthopedics
Pereira J.T., Knorst J.K., Ardenghi T.M., Piva F., Imparato J.C.P., Olegário I.C., Hermoza R.A.M., Armas-Vega A.D.C., de Araujo F.B.	Pulp Vitality and Longevity of Adhesive Restorations Are Not Affected by Selective Carious Removal: A Multicenter Clinical Trial	2021	Caries research

Hatrom AA, Howait MS, Zawawi KH, Al-Turki GA, Alansari RA, Almehayawi NF, Alammari SH, Mohammed RA, Hassan AH.	Pulp volume changes after piezocision-assisted tooth movement: a randomized clinical trial	2021	BMC Oral Health
Hamre H.J.; Mittag I.; Glockmann A.; Kiene H.; Tröger W.	Pulpa dentis D30 for acute reversible pulpitis: A prospective cohort study in routine dental practice	2012	Alternative Therapies in Health and Medicine
Chompu-Inwai P, Bua-On P, Nirunsittirat A, Chuveera P, Louwakul P, Sastraruji T.	Pulpal anesthesia in pediatric patients following supplemental mandibular buccal infiltration in vital permanent mandibular molars with deep caries	2020	Clin Oral Investig
Kiho K.; Sumitomo S.; Tanaka M.; Hasegawa T.; Sakai C.; Takitani Y.; Yoshida T.; Kawano S.	Pulpal Disease Arising from Medication-related Osteonecrosis of the Jaw: A Case Report	2020	Journal of Endodontics
Walia T., Alzayer W.F.A., Nemer M.N.A.A.	Pulpal dressing condensation methods in pulpotomy for primary molars: An in-vitro comparative study	2021	Saudi Dental Journal
Swarup S.; Rao A.; Boaz K.; Srikant N.; Shenoy R.	Pulpal response to nano hydroxyapatite, mineral trioxide aggregate and calcium hydroxide when used as a direct pulp capping agent: An in vivo study	2014	Journal of Clinical Pediatric Dentistry
He Y.; Gan Y.; Lu J.; Feng Q.; Wang H.; Guan H.; Jiang Q.	Pulpal Tissue Inflammatory Reactions after Experimental Pulpal Exposure in Mice	2017	Journal of Endodontics
Lim W.Y.; Madden L.E.; Becker D.L.	Pulpal upregulation of connexin 43 during pulpitis	2021	Clinical Oral Investigations
Bhatnagar NB, Mantri SP, Dube KA, Jaiswal NU, Singh VJ.	Pulpal-anesthesia of a mandibular first molar with irreversible pulpitis by inferior alveolar nerve block plus buccal infiltration using articaine or lignocaine	2020	J Conserv Dent
Peycheva K.	Pulp-capping with mineral trioxide aggregate	2015	Acta Medica Bulgarica
Rasidi M.Q.Z.B.M., Bhagya Lakhsmi T., Prabu D.	Pulpectomy in maxillary first molars with distal caries	2020	International Journal of Research in Pharmaceutical Sciences
Teja K.V.; Ramesh S.	Pulpotomy as an alternative to root canal treatment in mature permanent teeth with closed apex: A review	2020	Biomedicine (India)
Li Y, Sui B, Dahl C, Bergeron B, Shipman P, Niu L, Chen J, Tay FR.	Pulpotomy for carious pulp exposures in permanent teeth: A systematic review and meta-analysis	2019	J Dent
Hamilton A., Clarkson J.E., Ramsay C.R., Mannocci F., Jarad F., Albadri S., Ricketts D., Tait C., Banerjee A., Deery C., Boyers D., Marshman Z., Goulao B., Hamilton A.R., Banister K., Bell R., Brown L., Conway D.I., Donaldson P., Duncan A., Dunn K., Fee P., Forrest M., Glenny A.-M., Gouick J., Gupta E., Jacobsen E., Kettle J., MacLennan G., Macpherson L., McGuff T., Mitchell F., van der Pol M., Moazzez R., Roberston D., Wojewodka G., Young L., Lamont T.	Pulpotomy for the Management of Irreversible Pulpitis in Mature Teeth (PIP): a feasibility study	2022	Pilot and Feasibility Studies
Ngoc V.T.N.; Van Nga T.D.; Chu D.-T.; Anh L.Q.	Pulpotomy management using laser diode in pediatric patient with severe hemophilia A under general anesthesia—A case report	2018	Special Care in Dentistry
Howley B, Seale NS, McWhorter AG, Kerins C, Boozer KB, Lindsey D.	Pulpotomy versus pulpectomy for carious vital primary incisors: randomized controlled trial	2012	Pediatr Dent
Gadallah L, Hamdy M, El Bardissy A, Abou El Yazeed M.	Pulpotomy versus pulpectomy in the treatment of vital pulp exposure in primary incisors. A systematic review and meta-analysis	2018	F1000Res
Prabhakar A.R.; Mandroli P.; Bhat K.	Pulpotomy with curcumin: Histological comparison with mineral trioxide aggregate in rats	2019	Indian Journal of Dental Research
Vu T.T., Nguyen M.T., Sangvanich P., Thunyakitpisal P.	Pulse Oximetry and Three-Dimensional Analysis in Evaluating Immature Permanent Teeth Apexogenesis: Two Case Reports	2022	Open Dentistry Journal
Caldeira C.L.; Barletta F.B.; Ilha M.C.; Abrão C.V.; Gavini G.	Pulse oximetry: a useful test for evaluating pulp vitality in traumatized teeth	2016	Dental Traumatology
Hamdy M., Elgendi H., Sharaan M.	Push-Out Bond Strength and Dentinal Penetration of a Novel Herbal-Based Pulp Capping Agent: An In vitro Study	2022	Open Access Macedonian Journal of Medical Sciences

Silva E.J.N.L.; Carvalho N.K.; Prado M.C.; Zanon M.; Senna P.M.; Souza E.M.; De-Deus G.	Push-out Bond Strength of Injectable Pozzolan-based Root Canal Sealer	2016	Journal of Endodontics
Bullard S, Mona M, Pereira AC, Kajfasz J, Lemos JA, Abranches J, Wallet SM, Pileggi R.	Quantitative Analysis of Biofilm Removal Following Instrumentation with TRUShape and Vortex Blue File Systems: Microbiological Study	2022	Front Biosci (Schol Ed)
Carneiro ALE, Spin-Neto R, Zambrana NRM, Zambrana JRM, de Andrade Salgado DMR, Costa C.	Quantitative and qualitative comparisons of pulp cavity volumes produced by cone beam computed tomography and micro-computed tomography through semiautomatic segmentation: An ex vivo investigation	2023	Oral Surg Oral Med Oral Pathol Oral Radiol
Ong T.K.; Lim G.S.; Singh M.; Fial A.V.	Quantitative Assessment of Root Development after Regenerative Endodontic Therapy: A Systematic Review and Meta-Analysis	2020	Journal of Endodontics
Priyadharshini S.; Mahesh	RADIOGRAPHIC ANALYSIS OF ROOT CANAL TREATMENT DONE BY POSTGRADUATE STUDENTS IN AN UNIVERSITY DENTAL HOSPITAL	2022	European Chemical Bulletin
Moreira S.D.A.; Nunes J.B.; Colombo F.A.; Fonseca N.D.S.M.; Viola N.V.	Radiographic and antimicrobial evaluation of enterococcus Faecalis and Actinomyces Israelii micro-organisms after photodynamic therapy (aPDT)	2021	Photodiagnosis and Photodynamic Therapy
Siva Kumar M., Hari Kumar M., Vishalakshi K., Sabitha H.	Radiographic assessment of bone formation using rhBMP2 at maxillary periapical surgical defects: A case series	2016	Journal of Clinical and Diagnostic Research
Alfouzan K., Baskaradoss J.K., Geevarghese A., Alzahrani M., Alhezaimi K.	Radiographic Diagnosis of Periapical Status and Quality of Root Canal Fillings in a Saudi Arabian Subpopulation	2016	Oral health & preventive dentistry
Kiran N.K., Chowdhary N., Kumar M., Pavana M.P., Sridhara A.	Radiographic evaluation of different combinations of zinc oxide as an obturating material in pulpectomy: A comparative in vivo study	2020	Indian Journal of Public Health Research and Development
Wu J.; Li X.; Xu L.; Tang Z.; Zhao J.; Xiang Y.; Zhang Y.; Yang J.; Ye L.	Radiographic evaluation of immature traumatized incisors following different endodontic treatments	2021	Dental Traumatology
Bane K.; Charpentier E.; Bronnec F.; Descroix V.; Gaye-N'Diaye F.; Kane A.W.; Toledo R.; Machtou P.; Azérad J.	Randomized Clinical Trial of Intraosseous Methylprednisolone Injection for Acute Pulpitis Pain	2016	Journal of Endodontics
Franzon R, Opdam NJ, Guimarães LF, Demarco FF, Casagrande L, Haas AN, Araujo FB.	Randomized controlled clinical trial of the 24-months survival of composite resin restorations after one-step incomplete and complete excavation on primary teeth	2015	J Dent
Ghoul-Mazgar S.	RANK, RANKL and OPG expressions in a permanent molar with a replacement resorption	2013	Odontology
Tan K.S.; Yu V.S.H.; Quah S.Y.; Bergenholtz G.	Rapid method for the detection of root canal bacteria in endodontic therapy	2015	Journal of Endodontics
Rajasekaran S.; Rao S.S.; Dalavi P.A.; Prabhu A.; Anil S.; Venkatesan J.; Bhat S.S.	Rapid microwave-assisted biosynthesis of chitooligosaccharide coated silver nanoparticles: Assessments of antimicrobial activity for paediatric pulp therapy	2021	Advances in Natural Sciences: Nanoscience and Nanotechnology
Sothornwit J.; Charoensri S.; Pongchaiyakul C.	Rapid onset of osteonecrosis of the jaw in an osteoporosis patient treated with denosumab: A case report	2020	Journal of the Medical Association of Thailand
Patil PB, Chaudhari SG, Goel A, Agarwal P.	Rare association of dens invaginatus with impacted mesiodens - A case report	2012	J Oral Biol Craniofac Res
Sato T.; Suenaga H.; Igarashi M.; Hoshi K.; Takato T.	Rare case of external dental fistula of the submental region misdiagnosed as inverted follicular keratosis and thyroglossal duct cyst	2015	International Journal of Surgery Case Reports
Ring J.; Ring K.C.	Rare Root Canal Configuration of Mandibular Second Premolar Using Cone-beam Computed Tomographic Scanning	2017	Journal of Endodontics
Monea M.; Moldovan C.	Rare root morphology of a maxillary central incisor associated with gingival hyperplasia an endodontic case report	2016	Medicine (United States)
Koyuncuoglu C.Z.; Aydin M.; Kirmizi N.I.; Aydin V.; Aksoy M.; Isli F.; Akici A.	Rational use of medicine in dentistry: do dentists prescribe antibiotics in appropriate indications?	2017	European Journal of Clinical Pharmacology
Ribeiro CC, de Oliveira Lula EC, da Costa RC, Nunes AM.	Rationale for the partial removal of carious tissue in primary teeth	2012	Pediatr Dent
Krechina EK, Volkov AV, Abdurakhmanova ZU.	Rationale for the use of bioactive cements by in vitro simulation of accidental pulp opening	2021	Stomatologiia (Mosk)

Sabino C.P.; Garcez A.S.; Núñez S.C.; Ribeiro M.S.; Hamblin M.R.	Real-time evaluation of two light delivery systems for photodynamic disinfection of Candida albicans biofilm in curved root canals	2015	Lasers in Medical Science
Kamalova M.K., Fomenko I.V., Dmitrienko D.S., Matvienko N.V., Arjenovskaya E.N., Gevorkyan A.G., Nikitina K.V., Maslak E.E.	Reasons for 1-17-year-old children to visit A dentist during the covid-19 pandemic	2020	European Journal of Molecular and Clinical Medicine
Hsu U, Hui BK, Pourat N.	Recall compliance and incidence of dental caries among underserved children	2015	J Calif Dent Assoc
Saber AM, El Meligy OA, Alaki SM.	Recent Advances in Indirect Pulp Treatment Materials for Primary Teeth: A Literature Review	2021	Int J Clin Pediatr Dent
Nakashima M.; Iohara K.	Recent Progress in Translation from Bench to a Pilot Clinical Study on Total Pulp Regeneration	2017	Journal of Endodontics
Daher A, Abreu MH, Costa LR.	Recognizing preschool children with primary teeth needing dental treatment because of caries-related toothache	2015	Community Dent Oral Epidemiol
Al-Khanati NM, Kara Beit Z.	Reconsidering some standards in immediate autotransplantation of teeth: Case report with 2-year follow-up	2022	Ann Med Surg (Lond)
Zareiyan M., Molaasadolah F., Haghgoo R., Ahmadi R., Kahvand M.	Reconstruction of pulpotomized primary molar and retention of stainless-steel crowns: An in-vitro study	2020	Open Dentistry Journal
Farhin K., Viral P.M., Thejokrishna P., Sajjad M.	Reduction in Bacterial Loading Using MTAD as an Irrigant in Pulpectomized Primary Teeth	2015	The Journal of clinical pediatric dentistry
Duan W, Chen Y, Zhang Q, Lin X, Yang X.	Refined tooth and pulp segmentation using U-Net in CBCT image	2021	Dentomaxillofac Radiol
Al-Asmar A.A., Al-Hiyasat A.S., Abu-Awwad M., Mousa H.N., Salim N.A., Almadani W., Rihan F., Sawair F.A., Pitts N.B.	Reframing Perceptions in Restorative Dentistry: Evidence-Based Dentistry and Clinical Decision-Making	2021	International Journal of Dentistry
Saoud T.M.A.; Ricucci D.; Lin L.M.; Gaengler P.	Regeneration and repair in endodontics-a special issue of the regenerative endodontics-a new era in clinical endodontics	2016	Dentistry Journal
Roshene R.	Regeneration of dental pulp- A review	2015	Journal of Pharmaceutical Sciences and Research
Ling L.; Zhao Y.M.; Wang X.T.; Wen Q.; Ge L.H.	Regeneration of Dental Pulp Tissue by Autologous Grafting Stem Cells Derived from Inflammatory Dental Pulp Tissue in Immature Premolars in a Beagle Dog	2020	The Chinese journal of dental research : the official journal of the Scientific Section of the Chinese Stomatological Association (CSA)
Na S.; Zhang H.; Huang F.; Wang W.; Ding Y.; Li D.; Jin Y.	Regeneration of dental pulp/dentine complex with a three-dimensional and scaffold-free stem-cell sheet-derived pellet	2016	Journal of Tissue Engineering and Regenerative Medicine
Sabeti M.; Golchert K.; Torabinejad M.	Regeneration of Pulp-Dentin Complex in a Tooth with Symptomatic Irreversible Pulpitis and Open Apex Using Regenerative Endodontic Procedures	2021	Journal of Endodontics
Chen H.; Fu H.; Wu X.; Duan Y.; Zhang S.; Hu H.; Liao Y.; Wang T.; Yang Y.; Chen G.; Li Z.; Tian W.	Regeneration of pulpo-dentinal-like complex by a group of unique multipotent CD24a+ stem cells	2020	Science Advances
Al-Hezaimi K.; Naghshbandi J.; Alhuzaimi R.; Alonaizan F.; AlQwizany I.; Rotstein I.	Regeneration of secondary dentin using recombinant human platelet-derived growth factor and MTA for pulp capping: A randomized controlled human clinical trial	2020	International Journal of Periodontics and Restorative Dentistry
Lu J.; Kahler B.	Regenerative endodontic procedures for two traumatized mature anterior teeth with transverse root fractures	2022	BMC Oral Health
Tong H.J.; Sim Y.F.; Berdouses E.; Al-Jundi S.; El Shahawy O.; Nazzal H.	Regenerative endodontic therapy (RET) for managing immature non-vital teeth: experiences and opinions of paediatric dental practitioners in the European and Arabian regions	2021	European Archives of Paediatric Dentistry
Topçuoğlu G.; Topçuoğlu H.S.	Regenerative Endodontic Therapy in a Single Visit Using Platelet-rich Plasma and Biodentine in Necrotic and Asymptomatic Immature Molar Teeth: A Report of 3 Cases	2016	Journal of Endodontics
Kim S.G.; Solomon C.S.	Regenerative Endodontic Therapy in Mature Teeth Using Human-Derived Composite Amnion-Chorion Membrane as a Bioactive Scaffold: A Pilot Animal Investigation	2021	Journal of Endodontics
Tzanetakis G.N.; Giannakoulas D.G.; Papanakou S.; Gizani S.; Lygidakis N.	Regenerative endodontic therapy of immature permanent molars with pulp necrosis: a cases series and a literature review	2021	European Archives of Paediatric Dentistry

Pace R.; Giuliani V.; Di Nasso L.; Pagavino G.; Franceschi D.; Franchi L.	Regenerative Endodontic Therapy using a New Antibacterial Root Canal Cleanser in necrotic immature permanent teeth: Report of two cases treated in a single appointment	2021	Clinical Case Reports
Çalışkan M.K.; Demirci G.K.; Güneri P.	Regenerative endodontic therapy with platelet rich fibrin: Case series	2020	Journal of Clinical Pediatric Dentistry
Elfrink M.E.C.; Heijdra J.S.C.; Krikken J.B.; Kouwenberg-Bruring W.H.; Kouwenberg H.; Weerheijm K.L.; Veerkamp J.S.J.	Regenerative endodontic therapy: a follow-up of 47 anterior traumatised teeth	2021	European Archives of Paediatric Dentistry
Al-Haddad A.Y., Al-Namnam N.M.	Regenerative endodontic treatment in mature teeth: a systematic review and meta-analysis	2022	Giornale Italiano di Endodonzia
Paryani K.; Kim S.G.	Regenerative endodontic treatment of permanent teeth after completion of root development: A report of 2 cases	2013	Journal of Endodontics
Natera M.; Mukherjee P.M.	Regenerative Endodontic Treatment with Orthodontic Treatment in a Tooth with Dens Evaginatus: A Case Report with a 4-year Follow-up	2018	Journal of Endodontics
Diogenes A.; Ruparel N.B.; Shiloah Y.; Hargreaves K.M.	Regenerative endodontics A way forward	2016	Journal of the American Dental Association
He L.; Kim S.G.; Gong Q.; Zhong J.; Wang S.; Zhou X.; Ye L.; Ling J.; Mao J.J.	Regenerative Endodontics for Adult Patients	2017	Journal of Endodontics
Ambu E., Caruso S., Gatto R., Tecco S., Severino M.	Regenerative endodontics procedure of an immature permanent mandibular molar with a necrotic pulp using biodentine: A 16 months radiographic follow-up	2020	Journal of Biological Regulators and Homeostatic Agents
Rojas-Gutiérrez W.J.; Pineda-Vélez E.; Agudelo-Suárez A.A.	Regenerative Endodontics Success Factors and their Overall Effectiveness: An Umbrella Review	2022	Iranian Endodontic Journal
Tabatabayi M.H.; Tavakoli A.; Ameghani B.A.	Regenerative property of PRF used as capping material in pulpotomy in dogs	2017	Biomedical Research (India)
Yuan F, Zheng J, Sun Y, Wang Y, Lyu P.	Regulation and Measurement of the Heat Generated by Automatic Tooth Preparation in a Confined Space	2017	Photomed Laser Surg
Chang M.-C.; Lin L.-D.; Zwei-Ching Chang J.; Huang C.-F.; Chuang F.-H.; Lee J.-J.; Jeng P.-Y.; Wang T.-M.; Jeng J.-H.	Regulation of vascular cell adhesion molecule-1 in dental pulp cells by interleukin-1β: The role of prostanoids	2012	Journal of Endodontics
Sun C, Xie Y, Hu X, Fu J, Zhou J, Wu L.	Relationship between Clinical Symptoms and the Microbiota in Advanced Caries	2020	J Endod
Duruk G, Laloglu E.	Relationship Between Dental Caries and YKL-40 Levels in Saliva	2022	J Clin Pediatr Dent
Ashwatha Pratha A.; Jayalakshmi S.	Relationship between diabetes mellitus and pulpal infection & periapical diseases	2016	Journal of Pharmaceutical Sciences and Research
Pei J, Liu J, Chen Y, Liu Y, Liao X, Pan J.	Relationship between maxillary posterior molar roots and the maxillary sinus floor: Cone-beam computed tomography analysis of a western Chinese population	2020	J Int Med Res
Khairani A.; Fauziah E.; Budiardjo S.B.	Relationship between oral health-related quality of life and salivary cortisol levels in children with caries	2017	International Journal of Applied Pharmaceutics
Zhang W.; Dai Y.-B.; Wan P.-C.; Xu D.-D.; Guo Y.; Li Z.	Relationship between post-extraction pain and acute pulpitis: a randomised trial using third molars	2016	International Dental Journal
Sui H, Lv Y, Xiao M, Zhou L, Qiao F, Zheng J, Sun C, Fu J, Chen Y, Liu Y, Zhou J, Wu L.	Relationship between the difference in electric pulp test values and the diagnostic type of pulpitis	2021	BMC Oral Health
Alomari Q.D.; Khalaf M.E.; Al-Shawaf N.M.	Relative contribution of restorative treatment to tooth extraction in a teaching institution	2013	Journal of Oral Rehabilitation
Boutsiouki C, Frankenberger R, Krämer N.	Relative effectiveness of direct and indirect pulp capping in the primary dentition	2018	Eur Arch Paediatr Dent
Ha W.N.; Chaves G.S.; Decurcio D.; Estrela C.; Peters O.A.; Rossi-Fedele G.	Remaining dentinal thickness after simulated post space preparation and the fit of prefabricated posts to root canal preparation shapes	2021	Journal of the American Dental Association
Schwendicke F.	Removing Carious Tissue: Why and How?	2018	Monogr Oral Sci
Gnanasegaran N.; Govindasamy V.; Musa S.; Abu Kasim N.H.	ReNCell VM conditioned medium enhances the induction of dental pulp stem cells into dopaminergic like cells	2016	Cytotechnology
Li F.-C.; Hung W.-C.	Repair of a perforating internal resorption: two case reports	2016	Journal of Dental Sciences
Herrera D.R.; Herrera C.M.; Lima A.R.; Nagata J.Y.; Pereira A.C.; Silva E.J.; Soares A.J.; Gomes B.P.	Repair of apical root resorption associated with periodontitis using a new intracanal medicament protocol	2014	Journal of Oral Science

Wen B.; Huang Y.; Qiu T.; Huo F.; Xie L.; Liao L.; Tian W.; Guo W.	Reparative Dentin Formation by Dentin Matrix Proteins and Small Extracellular Vesicles	2021	Journal of Endodontics
Abdelaz P.; El Zoghbi A.; Shokry M.; Ahmed A.-Z.; Rasha H.	Reparative dentin formation using stem cell therapy versus calcium hydroxide in direct pulp capping: An animal study	2019	Brazilian Dental Journal
Tran X.V.; Salehi H.; Truong M.T.; Sandra M.; Sadoine J.; Jacquot B.; Cuisinier F.; Chaussain C.; Boukpessi T.	Reparative mineralized tissue characterization after direct pulp capping with calcium-silicate-based cements	2019	Materials
Jayaraman J, Dhar V, Donly KJ, Priya E, Raggio DP, Childers NK, Wright TJ, Nagendrababu V, Clarke M, King N, Clarkson J, Innes NPT.	Reporting stAndards for research in PedIatric Dentistry (RAPID): an expert consensus-based statement	2021	BMC Oral Health
Obadiah I., Gurunathan D., Ravindran V.	Requirements of back to back crowns in 2 to 6 year old children in maxillary arch - an observational study	2020	European Journal of Molecular and Clinical Medicine
Obadiah I.; Gurunathan D.; Ravindran V.	Requirements of back to back crowns in children aged 2 to 6 years in mandibular arch-an observational study	2020	International Journal of Research in Pharmaceutical Sciences
Luo YX, Sun ML, Shi PL, Liu P, Chen YY, Peng X.	Research progress in the relationship between Veillonella and oral diseases	2020	Hua Xi Kou Qiang Yi Xue Za Zhi
Chen JQ, Dong YM.	Research progress in vital pulp therapy in mature permanent teeth with carious pulp exposure	2022	Zhonghua Kou Qiang Yi Xue Za Zhi
Chu G, Zhang ZY, Zhou H, Yan CX, Chen T, Guo YC.	Research Progress of Age Estimation Based on Age-related Changes of Dentin-pulp Complex	2018	Fa Yi Xue Za Zhi
de Castro Kruly P.; Alenezi H.E.H.M.; Manogue M.; Devine D.A.; Dame-Teixeira N.; Garcia F.C.P.; Do T.	Residual Bacteriome after Chemomechanical Preparation of Root Canals in Primary and Secondary Infections	2022	Journal of Endodontics
Farahi F., Saberi E., Bijari S.	Residual dentin thickness at the apical third of mandibular first molar mesial root instrumented by nickel-titanium rotary and manual files with different tapers: an ex vivo study	2022	Giornale Italiano di Endodonzia
Bezzon OL, Rivera DS, Silva RA, Oliveira DS, Silva-Herzog D, Nelson-Filho P, Lucisano MP, Silva LA.	Resin luting materials: Tissue response in dog's teeth	2015	Microsc Res Tech
Cardoso F.G.R.; Valera M.C.; Khoury R.D.; Martinho F.C.	Resolution of Nasal Sinus Tract after Endodontic Therapy: A Case Report with Microbial Analysis	2021	Journal of Endodontics
Siddiqui Y.D.; Omori K.; Ito T.; Yamashiro K.; Nakamura S.; Okamoto K.; Ono M.; Yamamoto T.; Dyke T.E.V.; Takashiba S.	Resolvin D2 induces resolution of periapical inflammation and promotes healing of periapical lesions in rat periapical periodotitis	2019	Frontiers in Immunology
Chen J.; Xu H.; Xia K.; Cheng S.; Zhang Q.	Resolvin E1 accelerates pulp repair by regulating inflammation and stimulating dentin regeneration in dental pulp stem cells	2021	Stem Cell Research and Therapy
Xu H.; Chen J.; Ge J.; Xia K.; Tao S.; Su Y.; Zhang Q.	Resolvin E1 Ameliorates Pulpitis by Suppressing Dental Pulp Fibroblast Activation in a Chemerin Receptor 23-dependent Manner	2019	Journal of Endodontics
Nowicka A.; Lipski M.; Parafiniuk M.; Sporniak-Tutak K.; Lichota D.; Kosierkiewicz A.; Kaczmarek W.; Buczkowska-Radlińska J.	Response of human dental pulp capped with biodentine and mineral trioxide aggregate	2013	Journal of Endodontics
Louwakul P.; Lertchirakarn V.	Response of inflamed pulps of rat molars after capping with pulp-capping material containing fluocinolone acetonide	2015	Journal of Endodontics
Mahgoli H.A.; Arshad M.; Rasouli K.	Restoration of endodontically treated cracked maxillary teeth: A case series	2019	Clinical Case Reports
Guruprasada	Restoration of fractured endodontically treated mandibular first molar using custom made cast post and core	2015	Medical Journal Armed Forces India
Jain A., Bhat V., Sridevi U.	Restoration of highly mutilated endodontically treated multi-rooted teeth using customized interlocking post & core: A case report	2015	Nitte University Journal of Health Science
Memarpour M, Shafiei F.	Restoration of primary anterior teeth using intracanal polyethylene fibers and composite: an in vivo study	2013	J Adhes Dent
Salah A.; Raghad H.; Khalid A.; Hamid A.	Restoration of Primary Anterior Teeth with Glass Fiber-Reinforced Post and Core: 3-Year Follow-Up Case Report	2021	Case Reports in Dentistry
Jardim JJ, Mestrinho HD, Koppe B, de Paula LM, Alves LS, Yamaguti PM, Almeida JCF, Maltz M.	Restorations after selective caries removal: 5-Year randomized trial	2020	J Dent

Ferrari M., Pontoriero D.I.K., Ferrari Cagidiaco E., Carboncini F.	Restorative difficulty evaluation system of endodontically treated teeth	2022	Journal of Esthetic and Restorative Dentistry
Okuno T., Suzuki H., Inoue A., Kusukawa J.	Restricted Mandibular Movement Attributed to Ossification of Mandibular Depressors and Medial Pterygoid Muscles in Patients With Fibrodysplasia Ossificans Progressiva: A Report of 3 Cases	2017	Journal of Oral and Maxillofacial Surgery
Wang F.-M.; Hu Z.; Liu X.; Feng J.Q.; Augsburger R.A.; Gutmann J.L.; Glickman G.N.	Resveratrol represses tumor necrosis factor α/c-Jun N-terminal kinase signaling via autophagy in human dental pulp stem cells	2019	Archives of Oral Biology
Chen X, Zhang H, Zhong J, Yan W, Lin B, Ding M, Xue S, Xia B.	Retraction Note: Comparison of indirect pulp treatment and iRoot BP Plus pulpotomy in primary teeth with extremely deep caries: a prospective randomized trial	2022	Clin Oral Investig
Chawla A., Sujlana A., Dixit A.	Re-treating a maxillary second molar with 6 root canals assisted by cone beam computed tomography	2015	General dentistry
Burdurlu M.C., Dagasan V.C., Tunc O., Güler N.	Retrograde peri-implantitis: Evaluation and treatment protocols of a rare lesion	2021	Quintessence International
Ortega-Verdugo P, Warren JJ, Kolker JL, Carter KD, Guzmán-Armstrong S, Gomez MR.	Retrospective analysis of factors associated with the success of stepwise excavation procedure in deep carious lesions	2018	J Am Dent Assoc
Yu L.; Kahler B.; Nanayakkara S.; Prabhu N.	Retrospective analysis of the outcomes of pulpotomies in traumatised permanent anterior teeth	2022	Dental Traumatology
Bansal A.; Parihar A.S.; Sethi A.; Majety K.K.; Panjabi J.; Choudhury B.K.	Retrospective assessment of healing outcome of endodontic treatment for mandibular molars with C-shaped root canal	2017	Journal of Contemporary Dental Practice
Hui-Derksen E.K.; Chen C.F.; Majewski R.; Tootla R.G.; Boynton J.R.	Retrospective record review: reinforced zinc oxide-eugenol pulpotomy: a retrospective study.	2013	Pediatric dentistry
Wang N, Zhao YM.	Retrospective study of dental treatment under general anesthesia of 62 disabled children and adolescents	2018	Beijing Da Xue Xue Bao Yi Xue Ban
Wunsch P.B., Kuhnen M.M., Best A.M., Brickhouse T.H.	Retrospective Study of the Survival Rates of Indirect Pulp Therapy Versus Different Pulpotomy Medicaments	2016	Pediatric dentistry
Xie Y.; Lu F.; Hong Y.; He J.; Lin Y.	Revascularisation versus apexification for treatment of immature teeth based on periapical healing and root development: A systematic review and meta-analysis	2021	European Journal of Paediatric Dentistry
Archana M.S.; Sujana V.; Nagesh B.; Babu P.J.K.	Revascularization - An overview	2012	Journal of International Dental and Medical Research
Ramezani M., Sanaei-rad P., Hajihassani N.	Revascularization and vital pulp therapy in immature molars with necrotic pulp and irreversible pulpitis: A case report with two-year follow-up	2020	Clinical Case Reports
Nagaveni N.B.; Pathak S.; Poornima P.; Joshi J.S.	Revascularization induced maturogenesis of non-vital immature permanent tooth using platelet-rich-fibrin: A case report	2016	Journal of Clinical Pediatric Dentistry
Nagaveni N.B.; Poornima P.; Joshi J.S.; Pathak S.; Nandini D.B.	Revascularization of immature, nonvital permanent tooth using platelet-rich fibrin in children	2015	Pediatric dentistry
Sumidarti A.; Moersidi S.N.M.	Reversible pulpitis accompanied with sinus tract on buccal side of the right maxillary first premolar teeth: case report	2019	Journal of Dentomaxillofacial Science
Gudkina J, Mindere A, Locane G, Brinkmane A.	Review of the success of pulp exposure treatment of cariously and traumatically exposed pulps in immature permanent incisors and molars	2012	Stomatologija
Srikawnawan W.; Songsaad A.; Gonmanee T.; Thonabulsombat C.; Phruksaniyom C.; White K.L.; Ruangsawasdi N.	Rho kinase inhibitor induced human dental pulp stem cells to differentiate into neurons	2022	Life Sciences
Tankova H., Mitova N., Rashkova M., Popova H.	Risk factors and gingival inflammation in children aged 10 to 14 years-an epidemiological study	2021	Journal of IMAB - Annual Proceeding (Scientific Papers)
Ma J.; Chen W.; Zhang L.; Tucker B.; Zhu G.; Sasaki H.; Hao L.; Wang L.; Ci H.; Jiang H.; Stashenko P.; Li Y.-P.	RNA interference-mediated silencing of Atp6i prevents both periapical bone erosion and inflammation in the mouse model of endodontic disease	2013	Infection and Immunity
Hu J.; Chen W.; Qiu Z.; Lv H.	Robust expression of SIRT6 inhibits pulpitis via activation of the TRPV1 channel	2020	Cell Biochemistry and Function
Lv K, Wang G, Shen C, Zhang X, Yao H.	Role and mechanism of the nod-like receptor family pyrin domain-containing 3 inflammasome in oral disease	2019	Arch Oral Biol

Tavangar M.S.; Hosseini S.-M.; Dehghani-Nazhvani A.; Monabati A.	Role of CD146 enrichment in purification of stem cells derived from dental pulp polyp	2017	Iranian Endodontic Journal
Huang Y.; Qiao W.; Wang X.; Gao Q.; Peng Y.; Bian Z.; Meng L.	Role of Ku70 in the apoptosis of inflamed dental pulp stem cells	2018	Inflammation Research
Javed F.; Kellesarian S.V.; Abduljabbar T.; Gholamiazizi E.; Feng C.; Aldosary K.; Vohra F.; Romanos G.E.	Role of laser irradiation in direct pulp capping procedures: a systematic review and meta-analysis	2017	Lasers in Medical Science
Kanno K, Shimizu K, Shinoda M, Hayashi M, Takeichi O, Iwata K.	Role of macrophage-mediated Toll-like receptor 4-interleukin-1R signaling in ectopic tongue pain associated with tooth pulp inflammation	2020	J Neuroinflammation
Kanno K.; Shimizu K.; Shinoda M.; Hayashi M.; Takeichi O.; Iwata K.	Role of macrophage-mediated Toll-like receptor 4–interleukin-1R signaling in ectopic tongue pain associated with tooth pulp inflammation	2020	Journal of Neuroinflammation
Komiya H, Shimizu K, Noma N, Tsuboi Y, Honda K, Kanno K, Ohara K, Shinoda M, Ogiso B, Iwata K.	Role of Neuron-Glial Interaction Mediated by IL-1β in Ectopic Tooth Pain	2018	J Dent Res
Suzuki-Barrera K.; Makishi S.; Nakatomi M.; Saito K.; Ida-Yonemochi H.; Ohshima H.	Role of osteopontin in the process of pulpal healing following tooth replantation in mice	2022	Regenerative Therapy
Di Tinco R.; Bertani G.; Pisciotta A.; Bertoni L.; Pignatti E.; Maccaferri M.; Bertacchini J.; Sena P.; Vallarola A.; Tupler R.; Croci S.; Bonacini M.; Salvarani C.; Carnevale G.	Role of PD-L1 in licensing immunoregulatory function of dental pulp mesenchymal stem cells	2021	Stem Cell Research and Therapy
Cha M.; Sallem I.; Jang H.W.; Jung I.Y.	Role of transient receptor potential vanilloid type 1 in the trigeminal ganglion and brain stem following dental pulp inflammation	2020	International Endodontic Journal
De Pablo Ó.V.; Estevez R.; Heilborn C.; Cohenca N.	Root anatomy and canal configuration of the permanent mandibular first molar: Clinical implications and recommendations	2012	Quintessence International
Pedemonte E, Cabrera C, Torres A, Jacobs R, Harnisch A, Ramírez V, Concha G, Briner A, Brizuela C.	Root and canal morphology of mandibular premolars using cone-beam computed tomography in a Chilean and Belgian subpopulation: a cross-sectional study	2018	Oral Radiol
Ravi R., Rajesh R., Nama R., Alankrutha G., Nallabolu R.R., Singh R.	Root as a novel post and core material for primary teeth: A case report	2020	Journal of Pharmacy and Bioallied Sciences
Nascimento EHL, Nascimento MCC, Gaêta-Araujo H, Fontenele RC, Freitas DQ.	Root canal configuration and its relation with endodontic technical errors in premolar teeth: a CBCT analysis	2019	Int Endod J
Karunakaran J., Samuel L., Rishal Y., Joseph M., Suresh K., Varghese S.	Root canal configuration of human permanent mandibular first molars of an indo-dravidian population based in Southern India: An in vitro study	2017	Journal of Pharmacy and Bioallied Sciences
Zhang X.; Chen Y.; Li C.; Xue Z.; Wu H.; Li J.; Ou H.; Shen J.; Ding D.	Root Canal Disinfection Using Highly Effective Aggregation-Induced Emission Photosensitizer	2021	ACS Applied Bio Materials
Dalzell O.; Mohd Ariffin S.; Patrick C.J.; Hardiman R.; Manton D.J.; Parashos P.; Rajan S.	Root canal instrumentation efficacy of non-fused and fused primary molar roots: a micro-computed tomography study	2021	European Archives of Paediatric Dentistry
Kyaw Moe M.M., Ha J.H., Jin M.U., Kim Y.K., Kim S.K.	Root Canal Shaping Effect of Instruments with Offset Mass of Rotation in the Mandibular First Molar: A Micro-computed Tomographic Study	2018	Journal of endodontics
Landw D.J.	Root canal shaping using a reciprocating file system	2013	Dentistry Today
Hoshyari N., Shakeri F., Kohsar A.H.	Root canal treatment of a geminated maxillary lateral incisor: A case report	2020	Journal of Mazandaran University of Medical Sciences
Mathew J.; Devadathan A.; Syriac G.; Shamini S.	Root canal treatment of a maxillary first premolar with three roots	2015	Journal of Pharmacy and Bioallied Sciences
Shalavi S, Mohammadi Z, Abdolrazzaghi M.	Root canal treatment of maxillary and mandibular three-rooted premolars: case reports	2012	Iran Endod J
Olivieri JG, Duran-Sindreu F.	Root Dentine Thickness and Concavity Depth in Mandibular Molars: A Cone Beam Computed Tomography Population Study	2018	Eur Endod J
Senan E.M.; Alhadainy H.A.; Genaid T.M.; Madfa A.A.	Root form and canal morphology of maxillary first premolars of a Yemeni population	2018	BMC Oral Health
Witt CV, Hirt T, Rutz G, Luder HU.	Root malformation associated with a cervical mineralized diaphragm--a distinct form of tooth abnormality?	2014	Oral Surg Oral Med Oral Pathol Oral Radiol

Ozdogan M.S.; Gungormus M.; Ince Yusufoglu S.; Ertem S.Y.; Sonmez C.; Orhan M.	Salivary opiorphin in dental pain: A potential biomarker for dental disease	2019	Archives of Oral Biology
Kosch JCD, Zanno LE.	Sampling impacts the assessment of tooth growth and replacement rates in archosaurs: implications for paleontological studies	2020	PeerJ
Hassona Y., Rajab L., Taimeh D., Scully C.	Sanjad-Sakati Syndrome: Oral Health Care	2018	Medical Principles and Practice
Syed-Picard F.N.; Ray Jr. H.L.; Kumta P.N.; Sfeir C.	Scaffoldless tissue-engineered dental pulp cell constructs for endodontic therapy	2014	Journal of Dental Research
Colombo J.S.; Moore A.N.; Hartgerink J.D.; D'Souza R.N.	Scaffolds to control inflammation and facilitate dental pulp regeneration	2014	Journal of Endodontics
Jones CA, Bracewell T.	Scanning electron microscopy (SEM) and macroscopic analysis of immature human permanent molar immersion in hydrochloric acid (HCL, 38%)	2022	J Forensic Leg Med
Wang D.; Lyu Y.; Yang Y.; Zhang S.; Chen G.; Pan J.; Tian W.	Schwann cell-derived EVs facilitate dental pulp regeneration through endogenous stem cell recruitment via SDF-1/CXCR4 axis	2022	Acta Biomaterialia
Liao C, Liang S, Wang Y, Zhong T, Liu X.	Sclerostin is a promising therapeutic target for oral inflammation and regenerative dentistry	2022	J Transl Med
Watson E.E.; Metcalfe J.E.; Kreher M.R.; Maxymiw W.G.; Glogauer M.; Schimmer A.D.	Screening for dental infections achieves 6-fold reduction in dental emergencies during induction chemotherapy for acute myeloid leukemia	2020	JCO Oncology Practice
Descamps E.; Gorlier C.; Ottaviani S.; Palazzo E.; Dieudé P.; Forien M.	Screening of dental and sinus infections in rheumatoid arthritis	2021	European Journal of Clinical Investigation
Li M.; Sun X.; Ma L.; Jin L.; Zhang W.; Xiao M.; Yu Q.	SDF-1/CXCR4 axis induces human dental pulp stem cell migration through FAK/PI3K/Akt and GSK3β/β-catenin pathways	2017	Scientific Reports
Küçükkaya Eren S., Görduysus M.Ö., Şahin C.	Sealing ability and adaptation of root-end filling materials in cavities prepared with different techniques	2017	Microscopy research and technique
Ramalingam K.; Kaliyamurthy S.D.; Govindarajan M.; Swathi S.	Seckel syndrome: A report of a case	2012	Journal of Indian Society of Pedodontics and Preventive Dentistry
Prati C., Zamparini F., Spinelli A., Pelliccioni G.A., Pirani C., Gandolfi M.G.	Secondary root canal treatment with reciproc blue and K-file: Radiographic and esem-edx analysis of dentin and root canal filling remnants	2020	Journal of Clinical Medicine
Hiremath H.; Saikalyan S.; Kulkarni S.S.; Hiremath V.	Second-generation platelet concentrate (PRF) as a pulpotomy medicament in a permanent molar with pulpitis: A case report	2012	International Endodontic Journal
Steiner R.; Fischer-Colbrie R.; Bletsa A.; Laimer J.; Troger J.	Secretoneurin and PE-11 immunoreactivity in the human dental pulp	2018	Archives of Oral Biology
Lin X, Fu YJ, Ren GQ, Wen JH, Chen YF, Zhang Q.	Segmentation and accuracy validation of mandibular molar and pulp cavity on cone-beam CT images by U-net neural network	2022	Shanghai Kou Qiang Yi Xue
Clarkson JE, Ramsay CR, Ricketts D, Banerjee A, Deery C, Lamont T, Boyers D, Marshman Z, Goulao B, Banister K, Conway D, Dawett B, Baker S, Sherriff A, Young L, van der Pol M, MacLennan G, Floate R, Braid H, Fee P, Forrest M, Gouick J, Mitchell F, Gupta E, Dakri R, Kettle J, McGuff T, Dunn K.	Selective Caries Removal in Permanent Teeth (SCRiPT) for the treatment of deep carious lesions: a randomised controlled clinical trial in primary care	2021	BMC Oral Health
Schwendicke F, Leal S, Schlattmann P, Paris S, Dias Ribeiro AP, Gomes Marques M, Hilgert LA.	Selective carious tissue removal using subjective criteria or polymer bur: study protocol for a randomised controlled trial (SelecCT)	2018	BMJ Open
Verdugo-Paiva F, Zambrano-Achig P, Simancas-Racines D, Viteri-García A.	Selective removal compared to complete removal for deep carious lesions	2020	Medwave
Gözetici-Çil B, Erdem-Hepşenoğlu Y, Tekin A, Özcan M.	Selective removal to soft dentine or selective removal to firm dentine for deep caries lesions ın permanent posterior teeth: a randomized controlled clinical trial up to 2 years	2023	Clin Oral Investig
Labib M.E., Hassanein O.E., Moussa M., Yassen A., Schwendicke F.	Selective versus stepwise removal of deep carious lesions in permanent teeth: A randomised controlled trial from Egypt - An interim analysis	2019	BMJ Open
Yao Y, Luo A, Hao Y.	Selective versus stepwise removal of deep carious lesions: A meta-analysis of randomized controlled trials	2023	J Dent Sci

Elhennawy K, Finke C, Paris S, Reda S, Jost-Brinkmann PG, Schwendicke F.	Selective vs stepwise removal of deep carious lesions in primary molars: 12-Months results of a randomized controlled pilot trial	2018	J Dent
Barros MMAF, De Queiroz Rodrigues MI, Muniz FWMG, Rodrigues LKA.	Selective, stepwise, or nonselective removal of carious tissue: which technique offers lower risk for the treatment of dental caries in permanent teeth? A systematic review and meta-analysis	2020	Clin Oral Investig
Nguyen P.K.; Gao W.; Patel S.D.; Siddiqui Z.; Weiner S.; Shimizu E.; Sarkar B.; Kumar V.A.	Self-Assembly of a Dentinogenic Peptide Hydrogel	2018	ACS Omega
Ali A.H., Koller G., Foschi F., Andiappan M., Bruce K.D., Banerjee A., Mannocci F.	Self-Limiting versus Conventional Caries Removal: A Randomized Clinical Trial	2018	Journal of dental research
Ali A.H.; Thani F.B.; Foschi F.; Banerjee A.; Mannocci F.	Self-limiting versus rotary subjective carious tissue removal: A randomized controlled clinical trial—2-year results	2020	Journal of Clinical Medicine
Perdoncini N.N., Furquim C.P., Bonfim C.M.S., Soares G.M.S., Torres-Pereira C.C.	Self-perception of periodontal health status among individuals with Fanconi anemia	2021	Hematology, Transfusion and Cell Therapy
Singh A.K.; Khateeb S.U.; Pathrose S.P.; Kumar A.S.; Haribaskar S.; Thota G.	SEM Evaluation of Various Intracanal Irrigation Devices on Smear Layer Removal: A Comparative Study	2021	Journal of Contemporary Dental Practice
Shindo S.; Kumagai T.; Shirawachi S.; Takeda K.; Shiba H.	Semaphorin3A released from human dental pulp cells inhibits the increase in interleukin-6 and CXC chemokine ligand 10 production induced by tumor necrosis factor-α through suppression of nuclear factor-κB activation	2021	Cell Biology International
Wahby M.A.R., Taha S.E.E., Masry E.S.E., Abd Al Gawad R.Y.	Severe root resorption after obturating a primary molar using zinc oxide and eugenol at different follow-up periods – a case report	2020	Open Access Macedonian Journal of Medical Sciences
Pawar A.M., Bhardwaj A., Zanza A., Wahjuningrum D.A., Arora S., Luke A.M., Karobari M.I., Reda R., Testarelli L.	Severity of Post-Operative Pain after Instrumentation of Root Canals by XP-Endo and SAF Full Sequences Compared to Manual Instrumentation: A Randomized Clinical Trial	2022	Journal of Clinical Medicine
Cuadros-Fernández C.; Lorente Rodríguez A.I.; Sáez-Martínez S.; García-Binimelis J.; About I.; Mercadé M.	Short-term treatment outcome of pulpotomies in primary molars using mineral trioxide aggregate and Biodentine: a randomized clinical trial	2016	Clinical Oral Investigations
Simon S, Perard M, Zanini M, Smith AJ, Charpentier E, Djole SX, Lumley PJ.	Should pulp chamber pulpotomy be seen as a permanent treatment? Some preliminary thoughts	2013	Int Endod J
Tajmehr N, Graham A, Deery C.	Should we root treat children's first permanent molars?	2020	Evid Based Dent
Ferreira S.B.P.; Tavares W.L.F.; da Rosa M.A.C.; de Brito L.C.N.; Vieira L.Q.; Junior H.M.; Sobrinho A.P.R.	Sickle Cell Anemia In Brazil: Personal, Medical And Endodontic Patterns	2016	Brazilian Oral Research
Ventä I., Vehkalahti M.M., Huumonen S., Suominen A.L.	Signs of disease occur in the majority of third molars in an adult population	2017	International Journal of Oral and Maxillofacial Surgery
Zhu N.; Chatzistavrou X.; Papagerakis P.; Ge L.; Qin M.; Wang Y.	Silver-Doped Bioactive Glass/Chitosan Hydrogel with Potential Application in Dental Pulp Repair	2019	ACS Biomaterials Science and Engineering
Xue D.; Gong Z.; Zhu F.; Qiu Y.; Li X.	Simvastatin increases cell viability and suppresses the expression of cytokines and vascular endothelial growth factor in inflamed human dental pulp stem cells in vitro	2018	Advances in Clinical and Experimental Medicine
Jung J.Y.; Woo S.M.; Kim W.J.; Lee B.N.; Nör J.E.; Min K.S.; Choi C.H.; Koh J.T.; Lee K.J.; Hwang Y.C.	Simvastatin inhibits the expression of inflammatory cytokines and cell adhesion molecules induced by LPS in human dental pulp cells	2017	International Endodontic Journal
Jia W.; Zhao Y.; Yang J.; Wang W.; Wang X.; Ling L.; Ge L.	Simvastatin promotes dental pulp stem cell-induced coronal pulp regeneration in pulpotomized teeth	2016	Journal of Endodontics
Karthick A.; Pia J.C.; Tamilselvi R.; Mensudar R.	Single rooted mandibular first and second molars with single canal—A case report	2019	Indian Journal of Public Health Research and Development
Pulcini M.G., Vitelli C., Dian A., Radaelli K., Basso M.	Single tooth prosthetic restoration through surgical crown lengthening, conservative therapies and cad-cam milled restoration in lithium-disilicate: A case report	2019	Acta Stomatologica Croatica
Mergoni G.; Ganim M.; Lodi G.; Figini L.; Gagliani M.; Manfredi M.	Single versus multiple visits for endodontic treatment of permanent teeth	2022	Cochrane Database of Systematic Reviews
Edionwe J.I.; Shaba O.P.; Umesi D.C.	Single visit root canal treatment: A prospective study	2014	Nigerian Journal of Clinical Practice

Haridoss S.; Swaminathan K.; Rajendran V.; Rajendran B.	Single-rooted primary first mandibular molar	2014	BMJ Case Reports
Persic Bukmir R., Vidas J., Mance D., Pezelj-Ribaric S., Spalj S., Brekalo Prso I.	Socio-economic and health status as a predictor of apical periodontitis in adult patients in Croatia	2019	Oral Diseases
Jakšic Gvozdic D.J.; Milovanovic J.R.	Socioeconomic significance and prevention of early childhood caries; Socio-ekonomski znacaj i prevencija karijesa ranog detinjstva	2014	Acta Stomatologica Naissi
Deshwal R., Salaria S.K., Dahiya R., Deshwal N.	Socket-shield technique with minimally invasive osteotomy preparation as well as simultaneous sinus lift utilizing Densah Bur and platelet-rich fibrin membrane followed by immediate implant placement in a symptomatic posterior endodontically treated decayed root stumps site: A case report with 1-year follow-up	2022	Journal of Indian Society of Periodontology
Klein U.; Kleier D.J.	Sodium hypochlorite accident in a pediatric patient	2013	Pediatric Dentistry
Al-Mutairi M.A.; Bawazir O.A.	Sodium Hypochlorite versus Formocresol in primary molars pulpotomies: A randomized clinical trial	2013	European Journal of Paediatric Dentistry
Palone M.; Casella S.; De Sbrocchi A.; Siciliani G.; Lombardo L.	Space closure by miniscrew-assisted mesialization of an upper third molar and partial vestibular fixed appliance: A case report	2022	International Orthodontics
Ivanova EV, Shamkhalov GS, Dmitrieva NA, Akhmedova ZR.	Specific features of materials for initial pulpitis treatment	2014	Stomatologiia (Mosk)
Kwon SR, Dawson DV, Schenck DM, Fiegel J, Wertz PW.	Spectrophotometric Evaluation of Potassium Nitrate Penetration Into the Pulp Cavity	2015	Oper Dent
Smaïl-Faugeron V.; Muller-Bolla M.; Sixou J.-L.; Courson F.	Split-mouth and parallel-arm trials to compare pain with intraosseous anaesthesia delivered by the computerised Quicksleeper system and conventional infiltration anaesthesia in paediatric oral healthcare: Protocol for a randomised controlled trial	2015	BMJ Open
Sagomonyants K.; Mina M.	Stage-specific effects of fibroblast growth factor 2 on the differentiation of dental pulp cells	2014	Cells Tissues Organs
Ozorio J.E.V.; De Oliveira E Silva Carvalho L.F.; De Oliveira D.A.; De Sousa-Neto M.D.; Da Cruz Perez D.E.	Standardized propolis extract and calcium hydroxide as pulpotomy agents in primary pig teeth	2012	Journal of Dentistry for Children
Hsieh S.-C.; Tsao J.-T.; Lew W.-Z.; Chan Y.-H.; Lee L.-W.; Lin C.-T.; Huang Y.-K.; Huang H.-M.	Static magnetic field attenuates lipopolysaccharide-induced inflammation in pulp cells by affecting cell membrane stability	2015	Scientific World Journal
Cai J, Palamara J, Manton DJ, Burrow MF.	Status and progress of treatment methods for root caries in the last decade: a literature review	2018	Aust Dent J
Liang C, Liao L, Tian W.	Stem Cell-based Dental Pulp Regeneration: Insights From Signaling Pathways	2021	Stem Cell Rev Rep
Bjørndal L.	Stepwise Excavation	2018	Monogr Oral Sci
Massón M, Viteri-García A, Verdugo-Paiva F.	Stepwise removal compared to complete removal for deep carious lesions	2022	Medwave
Morales R.; Trujillo E.; Cantín M.	Stereological characterization of odontoblasts in normal healthy and reversible pulpitis in human dental pulps; Caracterización estereológica de odontoblastos en pulpas dentarias humanas sanas y con pulpitis reversible	2014	International Journal of Morphology
Kim J.-G.; Son K.M.; Park H.C.; Zhu T.; Kwon J.H.; Yang H.-C.	Stimulating effects of quercetin and phenamil on differentiation of human dental pulp cells	2013	European Journal of Oral Sciences
Songsiripradubboon S.; Kladkaew S.; Trairatvorakul C.; Sangvanich P.; Soontornvipart K.; Banlunara W.; Thunyakitpisal P.	Stimulation of Dentin Regeneration by Using Acemannan in Teeth with Lipopolysaccharide-induced Pulp Inflammation	2017	Journal of Endodontics
Pan L, Dumoncel J, Mazurier A, Zanolli C.	Structural analysis of premolar roots in Middle Pleistocene hominins from China	2019	J Hum Evol
Chang Y.; Zhu Z.; Shen N.; Zhao G.	STUDY OF BACTERIAL LEVELS AND ENDOTOXIN CONTENT IN REVERSIBLE PULPITIS CARIES	2022	Acta Medica Mediterranea
Wang M, Yin S, Wang Q, Gao Y, Wang Y, Zhang L.	Study of molar furcal perforation repaired with iRoot BP	2013	Hua Xi Kou Qiang Yi Xue Za Zhi
Todea C.; Igna A.; Ogodescu E.; Zetu I.; Ogodescu A.	Study of the compatibility of new biomaterials in vital pulp therapy in pediatric dentistry	2018	Revista de Chimie
Weimann D, Morgenthal A, Schwendicke F, Fleck C, Razi H.	Substantial regional differences in the biomechanical behavior of molar treated with selective caries tissue removal technique: a finite element study	2021	Dent Mater

Suhag K, Duhan J, Tewari S, Sangwan P.	Success of Direct Pulp Capping Using Mineral Trioxide Aggregate and Calcium Hydroxide in Mature Permanent Molars with Pulps Exposed during Carious Tissue Removal: 1-year Follow-up	2019	J Endod
Taylor G.D., Vernazza C.R., Abdulmohsen B.	Success of endodontic management of compromised first permanent molars in children: A systematic review	2020	International journal of paediatric dentistry
Tewari N, Goel S, Mathur VP, O'Connell AC, Johnson RM, Rahul M, Sultan F, Goswami M, Srivastav S, Ritwik P.	Success of medicaments and techniques for pulpotomy of primary teeth: An overview of systematic reviews	2022	Int J Paediatr Dent
Santos-Junior A.; De Castro Pinto L.; Mateo-Castillo J.; Pinheiro C.	Success or failure of endodontic treatments: A retrospective study	2019	Journal of Conservative Dentistry
Abd Al Gawad R.Y.; Hanafy R.M.H.	Success rate of three capping materials used in pulpotomy of primary molars: A randomized clinical trial	2021	Saudi Dental Journal
Amin M, Nouri MR, Hulland S, ElSalhy M, Azarpazhooh A.	Success Rate of Treatments Provided for Early Childhood Caries under General Anesthesia: A Retrospective Cohort Study	2016	Pediatr Dent
Trairatvorakul C, Detsomboonrat P.	Success rates of a mixture of ciprofloxacin, metronidazole, and minocycline antibiotics used in the non-instrumentation endodontic treatment of mandibular primary molars with carious pulpal involvement	2012	Int J Paediatr Dent
Lin GSS, Hisham ARB, Ch Er CIY, Cheah KK, Ghani NRNA, Noorani TY.	Success rates of coronal and partial pulpotomies in mature permanent molars: a systematic review and single-arm meta-analysis	2021	Quintessence Int
Clark W, Geneser M, Owais A, Kanellis M, Qian F.	Success rates of Hall technique crowns in primary molars: a retrospective pilot study	2017	Gen Dent
Midani R, Splieth CH, Mustafa Ali M, Schmoeckel J, Mourad SM, Santamaria RM.	Success rates of preformed metal crowns placed with the modified and standard hall technique in a paediatric dentistry setting	2019	Int J Paediatr Dent
Guven Y, Aksakal SD, Avcu N, Unsal G, Tuna EB, Aktoren O.	Success Rates of Pulpotomies in Primary Molars Using Calcium Silicate-Based Materials: A Randomized Control Trial	2017	Biomed Res Int
Yepes J.F.; Schweppe J.; Jones J.; Tang Q.; Eckert G.J.; Downey T.; Maupome G.	Success Rates of Pulpotomies Performed by General Dentists Versus Pediatric Dentists: A Claims Data Analysis	2020	Pediatric dentistry
Alshawwa H., Wang J.-F., Liu M., Sun S.-F.	Successful management of a tooth with endodontic-periodontal lesion: A case report	2020	World Journal of Clinical Cases
Behdad S., Caramês G., Pereira B., Pires M.D., Vasconcelos I., Ginjeira A.	Successful management of mandibular first molars with endodontic-periodontal lesions – Two case reports	2021	Revista Portuguesa de Estomatologia, Medicina Dentaria e Cirurgia Maxilofacial
El Shahawy O.I., O'Connell A.C.	Successful Restoration of Severely Mutilated Primary Incisors Using a Novel Method to Retain Zirconia Crowns - Two Year Results	2016	The Journal of clinical pediatric dentistry
Okaguchi M., Kuo T., Ho Y.-C.	Successful treatment of vertical root fracture through intentional replantation and root fragment bonding with 4-META/MMA-TBB resin	2019	Journal of the Formosan Medical Association
Ceperuelo D.; Lozano M.; Duran-Sindreu F.; Mercadé M.	Supernumerary fourth molar and dental pathologies in a Chalcolithic individual from the El Mirador Cave site (Sierra de Atapuerca, Burgos, Spain)	2015	HOMO
Satheesh S.L., Jain S., Bhuyan A.C., Devi L.S.	Surgical management of a separated endodontic instrument using second generation platelet concentrate and hydroxyapatite	2017	Journal of Clinical and Diagnostic Research
Zhukhovitskaya A.; Chang D.T.; Huoh K.C.; Pham N.S.; Singh J.; Ahuja G.S.	Surgical management of atypical mycobacterial cervical lymphadenitis in an outbreak of odontological infection	2020	International Journal of Pediatric Otorhinolaryngology
Modesto F.; Heimann M.; Donnelly T.M.	SURGICAL MANAGEMENT OF PANCREATIC NODULAR HYPERPLASIA BY PARTIAL PANCREATECTOMY IN A BLACK-TAILED PRAIRIE DOG (CYNOMYS LUDOVICIANUS)	2018	Journal of Exotic Pet Medicine
Chauhan P.S., Vellore K.P., Challa S.K., Ganesh M., Saigeeta K., Niharika S.	SURGICAL MANAGEMENT OF RADICULAR CYST AMONG ADOLESCENTS: CASE SERIES	2022	NeuroQuantology
Masgutova G.; Mukhamedshina Y.; Sergeev M.; Shulman I.; Ogurtsov S.; Masgutov R.; Rizvanov A.	Surgical Procedure for Extracting Pig Teeth for Isolation and Cultivation of Mesenchymal Stem Cells from Dental Pulp for Regenerative Therapy Applications	2017	BioNanoScience

Lin Y.-T.; Lin Y.-T.J.	Survey of comprehensive restorative treatment for children under general anesthesia	2015	Journal of Dental Sciences
Wisniewski J.F., Norooz S., Callahan D., Mohajeri A.	Survey of Vital Pulp Therapy Treatment in Permanent Dentition Being Taught at U.S. Dental Schools	2022	Journal of endodontics
Xie Y.; Wang Y.; Ma Q.; Li J.; Chen Y.; Yang R.; Huang R.; Zhang Q.; Zou J.	Survival analysis of pulpectomy in primary molars performed under dental general anaesthesia: a two-year retrospective study	2022	BMC Oral Health
Campagna P, Pinto LT, Lenzi TL, Ardenghi TM, de Oliveira Rocha R, Oliveira MDM.	Survival and Associated Risk Factors of Composite Restorations in Children with Early Childhood Caries: A Clinical Retrospective Study	2018	Pediatr Dent
Pedano M.S.; Li X.; Jeanneau C.; Ghosh M.; Yoshihara K.; Van Landuyt K.; About I.; Van Meerbeek B.	Survival of human dental pulp cells after 4-week culture in human tooth model	2019	Journal of Dentistry
Tseveenjav B, Furuholm J, Mulic A, Valen H, Maisala T, Turunen S, Varsio S, Auero M, Tjäderhane L.	Survival of primary molars with pulpotomy interventions: public oral health practice-based study in Helsinki	2021	Acta Odontol Scand
Yoshino K, Ito K, Kuroda M, Sugihara N.	Survival Rate of 3-unit Fixed Partial Dentures Replacing First Molars: A Retrospective Cohort Study	2023	Bull Tokyo Dent Coll
Strbac G.D., Giannis K., Mittlböck M., Fuerst G., Zechner W., Stavropoulos A., Ulm C.	Survival rate of autotransplanted teeth after 5 years – A retrospective cohort study	2017	Journal of Cranio-Maxillofacial Surgery
Abanto J.; Olegário I.C.; Mendes F.M.; Bönecker M.J.S.; Pires Corrêa M.S.N.	Survival rate of pulpectomy in primary teeth using Feapex® paste: A clinical study in infants; Sobrevida de pulpectomia em dentes decíduos utilizando pasta Feapex®: Um estudo clínico em bebês	2021	Brazilian Dental Science
Al-Hijazi A.Y.; Al-Khafaji L.K.; Raheem N.N.	Syndecan-4 and alkaline phosphatase enhancement by local application of exogenous growth factors on traumatic pulp of osteoporotic rats: Immuno-histochemical analysis	2020	International Journal of Pharmaceutical Research
Howard J.; Gardner L.; Saifee Z.; Geleil A.; Nelson I.; Colombo J.S.; Naleway S.E.; Carlson K.	Synthesis and characterization of novel calcium phosphate glass-derived cements for vital pulp therapy	2020	Journal of Materials Science: Materials in Medicine
Alipour M.; Fadakar S.; Aghazadeh M.; Salehi R.; Samadi Kafil H.; Roshangar L.; Mousavi E.; Aghazadeh Z.	Synthesis, characterization, and evaluation of curcumin-loaded endodontic reparative material	2021	Journal of Biochemical and Molecular Toxicology
Wang J.; Zuzzio K.; Walker C.L.	Systemic dental pulp stem cell secretome therapy in a mouse model of amyotrophic lateral sclerosis	2019	Brain Sciences
Butt R.; Aspinall A.; Tanday A.; Brown C.J.	Talon Cusp Management: A Case Series	2022	Dental Update
Mohan R.P.S.; Verma S.; Agarwal N.; Singh U.	Taurodontism	2013	BMJ Case Reports
Li Y, Jiang X, Hao J, Zhang Y, Huang R.	Tea polyphenols: application in the control of oral microorganism infectious diseases	2019	Arch Oral Biol
Hincapié S.; Fuks A.; Mora I.; Bautista G.; Socarras F.	Teaching and practical guidelines in pulp therapy in primary teeth in Colombia - South America	2015	International Journal of Paediatric Dentistry
Chhay S, Nguyen C.	Techniques for Preserving Pulp Vitality in an Asymptomatic Deep Carious Lesion: A Clinical Case Study	2022	Compend Contin Educ Dent
Vacchiano V.; Frattaruolo N.; Mancinelli L.; Foschi M.; Carotenuto A.; Scandellari C.; Piattelli M.; Brescia Morra V.; Lugaresi A.	Teeth loss after teriflunomide treatment: Casual or causal? A short case series	2018	Multiple Sclerosis and Related Disorders
Shruthi C.S., Poojya R., Ram S., Anupama	Telescopic overdenture: A case report	2017	International Journal of Biomedical Science
Yilmaz Y.; Keles S.; Mete A.	Temperature changes in the pulpal chamber and the sealing performance of various methods of direct pulp capping of primary teeth	2013	European Journal of Paediatric Dentistry
Jamshidy L., Parvaz A.	Tensile Strength of non-precious gold, cobalt-chromium, and fiber Posts cemented with panavia F2 resin cement in root canals of endodontically-treated teeth	2020	Pakistan Journal of Medical and Health Sciences
Feitosa V.P.; Mota M.N.; Savoldi R.; Rifane T.; de Paula D.; Borges L.; Solheiro L.K.; Aguiar Neto M.; Vieira L.; Moreira A.C.; Sauro S.	The Allogenic Dental Pulp Transplantation from Son/Daughter to Mother/Father: A Follow-Up of Three Clinical Cases	2022	Bioengineering
Ko Y.-J.; Kwon K.-Y.; Kum K.-Y.; Lee W.-C.; Baek S.-H.; Kang M.K.; Shon W.-J.	The Anti-Inflammatory Effect of Human Telomerase-Derived Peptide on P. gingivalis Lipopolysaccharide-Induced	2015	Mediators of Inflammation

	Inflammatory Cytokine Production and Its Mechanism in Human Dental Pulp Cells		
Eba H.; Murasawa Y.; Iohara K.; Isogai Z.; Nakamura H.; Nakamura H.; Nakashima M.	The Anti-Inflammatory Effects of Matrix Metalloproteinase-3 on Irreversible Pulpitis of Mature Erupted Teeth	2012	PLoS ONE
Jurič I.B.; Plečko V.; Pandurić D.G.; Anić I.	The antimicrobial effectiveness of photodynamic therapy used as an addition to the conventional endodontic re-treatment: A clinical study	2014	Photodiagnosis and Photodynamic Therapy
Siqueira J.F., Jr.; Antunes H.S.; Pérez A.R.; Alves F.R.F.; Mdala I.; Silva E.J.N.L.; Belladonna F.G.; Rôças I.N.	The Apical Root Canal System of Teeth with Posttreatment Apical Periodontitis: Correlating Microbiologic, Tomographic, and Histopathologic Findings	2020	Journal of Endodontics
Chen Y, Ma Y, Yang X, Chen J, Yang B, Tian W.	The Application of Pulp Tissue Derived-Exosomes in Pulp Regeneration: A Novel Cell-Homing Approach	2022	Int J Nanomedicine
Ptak D.M.; Finkelman M.D.; Amato R.B.	The Association between Choice of Diagnostic Imaging Modality and Long-term Treatment Outcomes for Patients Undergoing Nonsurgical Root Canal Treatment on Maxillary First Molars	2021	Journal of Endodontics
Petersen J.; Glaßl E.-M.; Nasseri P.; Crismani A.; Luger A.K.; Schoenherr E.; Bertl K.; Glodny B.	The association of chronic apical periodontitis and endodontic therapy with atherosclerosis	2014	Clinical Oral Investigations
Özverel C.S.; Islam A.; Yllmaz H.G.	The biostimulative effectiveness of photobiomodulation therapy application on thawed dental pulp stem cells	2021	Journal of Innovative Optical Health Sciences
Bani M.; Aktaş N.; Çinar Ç.; Odabaş M.E.	The clinical and radiographic success of primary molar pulpotomy using biodentine" and mineral trioxide aggregate: A 24-month randomized clinical trial	2017	Pediatric Dentistry
Jia L.; Zhang X.; Shi H.; Li T.; Lv B.; Xie M.	The clinical effectiveness of calcium hydroxide in root canal disinfection of primary teeth: A meta-analysis	2019	Medical Science Monitor
Consolaro A.; Cardoso M.A.; De Almeida C.D.C.M.; Souza I.A.O.; Filho L.C.	The clinical meaning of external cervical resorption in maxillary canine: Transoperative dental trauma	2014	Dental Press Journal of Orthodontics
Moradi Eslami L.; Vatanpour M.; Aminzadeh N.; Mehrvarzfar P.; Taheri S.	The comparison of intracanal medicaments, diode laser and photodynamic therapy on removing the biofilm of Enterococcus faecalis and Candida albicans in the root canal system (ex-vivo study)	2019	Photodiagnosis and Photodynamic Therapy
Sobral A.P.T., Santos E.M., Aranha A.C., Soares P.V., Moriyama C.M., Gonçalves M.L.L., Ribeiro R.A., Motta L.J., Horliana A.C.R.T., Fernandes K.P.S., Mesquita-Ferrari R.A., Bussadori S.K.	The control of pain due to dentin hypersensitivity in individuals with molar-incisor hypomineralisation: a protocol for a randomised controlled clinical trial	2021	BMJ Open
Khosrozadeh M.; Ghadimi S.; Kazemzadeh Gharghabi M.; Kharrazifard M.J.; Hamrah M.H.; Baghalian A.	The Correlation between Children's Intelligence Quotient and Their Behavior in Dental Setting: A Cross-Sectional Study	2022	BioMed Research International
Friedman M.E.; Quiñonez C.; Barrett E.J.; Boutis K.; Casas M.J.	The Cost of Treating Caries-Related Complaints at a Children's Hospital Emergency Department	2018	Journal (Canadian Dental Association)
Kunert M.; Rozpedek-Kaminska W.; Galita G.; Sauro S.; Bourgi R.; Hardan L.; Majsterek I.; Lukomska-Szymanska M.	The Cytotoxicity and Genotoxicity of Bioactive Dental Materials	2022	Cells
Kopke S, Angrisani N, Staszyk C.	The dental cavities of equine cheek teeth: three-dimensional reconstructions based on high resolution micro-computed tomography	2012	BMC Vet Res
Almanea S.M.; Alhadlaq M.A.; Albuqmi N.M.; Algomaiz S.S.	The Dental Management of Pediatric Patient Diagnosed with Myasthenia Gravis: A Case Report	2022	European Journal of Dentistry
Wu Q.; Li S.; Li R.; Chen X.; Guo L.; Zheng Y.	The detection of pro-inflammatory cytokines in exudates from dental pulp tissues	2022	Cytokine
Zhang M.; Kokabu S.; Nakatomi C.; Sugiyama G.; Matsuo K.; Jimi E.	The Distinct Distributions of Immunocompetent Cells in Rat Dentin Pulp After Pulpotomy	2015	Anatomical Record
Costa-Santos L.; Silva-Junior Z.S.; Sfalcin R.A.; da Mota A.C.C.; Tempestini Horliana A.C.R.; Motta L.J.; Mesquita-Ferrari R.A.; Santos Fernandes K.P.; Prates R.A.; Teixeira Silva D.F.; Deana A.; Bussadori S.K.	The effect of antimicrobial photodynamic therapy on infected dentin in primary teeth	2019	Medicine (United States)

Üstün Y., Topçuoğlu H.S., Akpek F., Aslan T.	The effect of blood contamination on dislocation resistance of different endodontic reparative materials	2015	Journal of oral science
Khdairah H.R.; Al-Gharrawi H.A.	The effect of canal preparation using 2shape, protaper gold and protaper next file systems on the fracture resistance of obturated roots	2020	Journal of International Dental and Medical Research
Abada H.M.; Hashem A.A.R.; Abu-Seida A.M.; Nagy M.M.	The effect of changing apical foramen diameter on regenerative potential of mature teeth with necrotic pulp and apical periodontitis	2022	Clinical Oral Investigations
Kalogeropoulos K., Xiropotamou A., Koletsi D., Tzanetakis G.N.	The Effect of Cone-Beam Computed Tomography (CBCT) Evaluation on Treatment Planning after Endodontic Instrument Fracture	2022	International Journal of Environmental Research and Public Health
Cintra L.T.A.; Ferreira L.L.; Benetti F.; Gastélum A.A.; Gomes-Filho J.E.; Ervolino E.; Briso A.L.F.	The effect of dental bleaching on pulpal tissue response in a diabetic animal model	2017	International Endodontic Journal
Stefanovic V.; Taso E.; Kanjevac T.; Abazovic D.; Rakic M.; Petkovic-Curcin A.; Acovic A.; Vojvodic D.	The effect of dental caries and restorative biomaterials on IL-1 β and TNF-α levels in the gingival crevicular fluid	2021	Vojnosanitetski Pregled
Oliveira D, Rocha MG, Zoidis P, Pereira P, Ribeiro AP.	The effect of different pulp capping methods on the intrapulpal temperature when using light-cured procedures	2022	J Clin Exp Dent
Omidi S.; Bagheri M.; Fazli M.; Ahmadiankia N.	The effect of different pulp-capping materials on proliferation, migration and cytokine secretion of human dental pulp stem cells	2020	Iranian Journal of Basic Medical Sciences
Akashi Y.; Nemoto A.; Nakajima K.; Kokubun K.; Murakami S.; Inoue T.; Matsuzaka K.	The effect of fibroblast growth factor 7 on human dental pulp stem cells for differentiation to AQP5-positive and αSMA-positive cells in vitro and in vivo	2021	Clinical and Experimental Dental Research
Zaen El-Din A.M.; Hamama H.H.; Abo El-Elaa M.A.; Grawish M.E.; Mahmoud S.H.; Neelakantan P.	The effect of four materials on direct pulp capping: An animal study	2020	Australian Endodontic Journal
El-Meligy O., Maashi M., Al-Mushayt A., Al-Nowaiser A., Al-Mubark S.	The Effect of Full-Mouth Rehabilitation on Oral Health-Related Quality of Life for Children with Special Health Care Needs	2016	The Journal of clinical pediatric dentistry
Ahmadian E, Eftekhari A, Dizaj SM, Sharifi S, Mokhtarpour M, Nasibova AN, Khalilov R, Samiei M.	The effect of hyaluronic acid hydrogels on dental pulp stem cells behavior	2019	Int J Biol Macromol
Veličkovic Z.; Živkovic D.; Bubalo M.; Živkovic M.; Mitic A.; Miladinovic M.; Duka M.; Lazic D.	The effect of hydroxyapatite and growth factors on reparative dentine formation in the therapy of injured pulp	2021	Vojnosanitetski Pregled
Akhlaghi N., Azarshab M., Akhoundi N., Meraji N.	The effect of ketorolac buccal infiltration on postoperative endodontic pain: A prospective, double-blind, randomized, controlled clinical trial	2019	Quintessence International
Fekrazad R.; Seraj B.; Ghadimi S.; Dehghan M.-M.	The effect of low-level laser therapy (810 nm) on root development of immature permanent teeth in dogs	2015	Lasers in Medical Science
Asnaashari M.; Shojaeian S.; Mesgharani A.; Mehrabinia P.	The Effect of Low-Level Laser Therapy on the Viability of Human Dental Pulp Stem Cells	2022	Journal of Lasers in Medical Sciences
Moradi Askari E, Parirokh M, Nakhaee N, Hosseini HR, Abbott PV.	The Effect of Maxillary First Molar Root Length on the Success Rate of Buccal Infiltration Anesthesia	2016	J Endod
Tahira T.; Jouhar R.; Ghani H.; Ahmed N.; Rao A.; Jamil S.	The effect of mineral trioxide aggregate as a direct pulp capping agent in permanent teeth	2018	Journal of International Oral Health
Bshara N.G.; Ataya J.	The effect of naocl gel activated by ultrasonic device on bovine dental pulp dissolution: An in vitro study	2020	Journal of Stomatology
Kong Q.; Liu L.; Huang Y.; Zhang F.; Wei X.; Ling J.	The effect of octamer-binding transcription factor 4B1 on microRNA signals in human dental pulp cells with inflammatory response	2014	Journal of Endodontics
Keles S., Kocaturk O.	The Effect of Oral Dexmedetomidine Premedication on Preoperative Cooperation and Emergence Delirium in Children Undergoing Dental Procedures	2017	BioMed Research International
Ashraf A., Hussein W., Hashem A., Soliman A.	The Effect of Passive Ultrasonic Irrigation and XP-Endo Finisher on Post-operative Pain after Endodontic Retreatment on Patients (Randomized Controlled Clinical Trial)	2022	Open Access Macedonian Journal of Medical Sciences
Elsaka S., Hussein W., Hashem A., Soliman A.	The Effect of Passive Ultrasonic Irrigation or XP-Endo Finisher on Post-Operative Pain in Patients with Necrotic Teeth with Apical Periodontitis (Randomized Controlled Clinical Trial)	2022	Open Access Macedonian Journal of Medical Sciences
Alves-Silva E.G.; Arruda-Vasconcelos R.; Louzada L.M.; de-Jesus-Soares A.;	The effect of photodynamic therapy on postoperative pain in teeth with primary endodontic infection	2022	Photodiagnosis and Photodynamic Therapy

Ferraz C.C.R.; Almeida J.F.A.; Marciano M.A.; Steiner-Oliveira C.; Bello-Silva M.S.; Shemesh H.; Gomes B.P.F.D.A.			
Hsu C.-L.; Lin W.-S.; Lin C.-H.; Liu J.	The effect of professional fluoride application program for preschool children in Taiwan: An analysis using the National Health Insurance Research Database (NHIRD)	2018	Journal of Dental Sciences
van der Weijden F.N.; Hesse D.; Americano G.C.A.; Soviero V.M.; Bonifacio C.C.	The effect of pulp inflammation and premature extraction of primary molars on the successor permanent teeth. A retrospective study	2020	International Journal of Paediatric Dentistry
Akcay M, Sari S.	The effect of sodium hypochlorite application on the success of calcium hydroxide and mineral trioxide aggregate pulpotomies in primary teeth	2014	Pediatr Dent
Ozlek E.; Gunduz H.; Kadi G.; Taşan A.; Akkol E.	The effect of solution and gel forms of sodium hypochlorite on postoperative pain: A randomized clinical trial	2021	Journal of Applied Oral Science
Gümüş H.; Delikan E.	The effect of sonic activation of irrigant on postoperative pain after root canal treatment in primary molar teeth: a randomized, clinical study	2020	Clinical Oral Investigations
Yavari H.R.; Jafari F.; Jamloo H.; Hallaj-Nezhadi S.; Jafari S.	The Effect of Submucosal Injection of Corticosteroids on Pain Perception and Quality of Life after Root Canal Treatment of Teeth with Irreversible Pulpitis: A Randomized Clinical Trial	2019	Journal of Endodontics
Khalighinejad N, Aminoshariae A, Kulild JC, Williams KA, Wang J, Mickel A.	The Effect of the Dental Operating Microscope on the Outcome of Nonsurgical Root Canal Treatment: A Retrospective Case-control Study	2017	J Endod
Li J.-Y.; Wang S.-N.; Dong Y.-M.	The effect of topical application of meloxicam on inflamed dental pulp	2021	Journal of Dental Sciences
Drukteinis S., Bilvinaite G., Shemesh H., Tusas P., Peciuliene V.	The effect of ultrasonic agitation on the porosity distribution in apically perforated root canals filled with different bioceramic materials and techniques: A micro-ct assessment	2021	Journal of Clinical Medicine
Dawood A.E.; Manton D.J.; Parashos P.; Wong R.H.	The effect of working time on the displacement of Biodentine™ beneath prefabricated stainless steel crown: a laboratory study	2016	Journal of investigative and clinical dentistry
Dou L.; Luo J.; Yang D.; Wang Y.	The effectiveness of an additional lingual infiltration in the pulpal anesthesia of mandibular teeth: A systematic review	2013	Quintessence International
Oliveira LSJ, de Bragança RMF, Sarkis-Onofre R, Faria-E-Silva AL.	The effectiveness of the supplementary use of the XP-endo Finisher on bacteria content reduction: a systematic review and meta-analysis	2021	Restor Dent Endod
Tüfenkçi P., Yılmaz K.	The Effects of Different Endodontic Access Cavity Design and Using XP-endo Finisher on the Reduction of Enterococcus faecalis in the Root Canal System	2020	Journal of endodontics
Li D, Fu L, Zhang Y, Yu Q, Ma F, Wang Z, Luo Z, Zhou Z, Cooper PR, He W.	The effects of LPS on adhesion and migration of human dental pulp stem cells in vitro	2014	J Dent
Coelho M.S.; Vilas-Boas L.; Tawil P.Z.	The effects of photodynamic therapy on postoperative pain in teeth with necrotic pulps	2019	Photodiagnosis and Photodynamic Therapy
Zhang M.; Jiang F.; Zhang X.; Wang S.; Jin Y.; Zhang W.; Jiang X.	The Effects of Platelet-Derived Growth Factor-BB on Human Dental Pulp Stem Cells Mediated Dentin-Pulp Complex Regeneration	2017	Stem Cells Translational Medicine
Diananda; Kamizar; Margono A.; Asrianti D.; Meydiawati R.	The efficacy of advanced platelet-rich fibrin (a-prf) on fibroblast cell regeneration	2017	Journal of International Dental and Medical Research
Maljaei E.; Pourkazemi M.; Ghanizadeh M.; Ranjbar R.	The efficacy of buccal infiltration of 4% articaine and psa injection of 2% lidocaine on anesthesia of maxillary second molars	2017	Iranian Endodontic Journal
Makeeva IM, Volkov AG, Prikuls VF, Dikopova NZ, Arakelyan MG, Makeeva MK, Ruchkin DN.	The efficacy of electroodontodiagnosis by means of various types of current	2018	Stomatologiia (Mosk)
King E.M.; Cerajewska T.L.; Locke M.; Claydon N.C.A.; Davies M.; West N.X.	The Efficacy of Plasma Rich in Growth Factors for the Treatment of Alveolar Osteitis: A Randomized Controlled Trial	2018	Journal of Oral and Maxillofacial Surgery
Marsa R.D.; Asrianti D.; Margono A.	The efficacy of platelet-rich fibrin lysate (PRF-L) for fibroblast cell proliferation	2017	Journal of International Dental and Medical Research
Ramachandran A, Khan SI, Mohanavelu D, Kumar KS.	The efficacy of pre-operative oral medication of paracetamol, ibuprofen, and aceclofenac on the success of maxillary infiltration anesthesia in patients with irreversible pulpitis: A double-blind, randomized controlled clinical trial	2012	J Conserv Dent

Kaiwar A.; Usha H.; Meena N.; Ashwini P.; Murthy C.	The efficiency of root canal disinfection using a diode laser: In vitro study	2013	Indian Journal of Dental Research
Roßgardt J, Heilen LB, Büttner K, Dern-Wieloch J, Vogelsberg J, Staszyk C.	The Equine Dental Pulp: Histomorphometric Analysis of the Equine Dental Pulp in Incisors and Cheek Teeth	2022	Vet Sci
Pourhajibagher M.; Ghorbanzadeh R.; Parker S.; Chiniforush N.; Bahador A.	The evaluation of cultivable microbiota profile in patients with secondary endodontic infection before and after photo-activated disinfection	2017	Photodiagnosis and Photodynamic Therapy
Çelik B.N., Mutluay M.S., Arıkan V., Sarı Ş.	The evaluation of MTA and Biodentine as a pulpotomy materials for carious exposures in primary teeth	2019	Clinical oral investigations
Trope M.	The expanding role of vital pulp therapy	2016	Dentistry Today
Grzeczkowicz A, Granicka LH, Maciejewska I, Strawski M, Szklarczyk M, Borkowska M.	The Experimental Study of the Performance of Nano-Thin Polyelectrolyte Shell for Dental Pulp Stem Cells Immobilization	2015	J Nanosci Nanotechnol
Cho Y.S.; Kim Y.S.; Moozhayil S.J.; Yang E.S.; Bae Y.C.	The expression of hyperpolarization-activated cyclic nucleotide-gated channel 1 (HCN1) and HCN2 in the rat trigeminal ganglion, sensory root, and dental pulp	2015	Neuroscience
Liu L, Peng B.	The expression of macrophage migration inhibitory factor is correlated with receptor activator of nuclear factor kappa B ligand in induced rat periapical lesions	2013	J Endod
Ismiyatin K.; Wahluyo S.; Soetojo A.; Rahayu R.; Utomo H.; Anindya C.	The expression of pulpal substance P after dentinal application of Escherichia coli lipopolysaccharide	2019	Saudi Endodontic Journal
Xing S, Martinón-Torres M, Bermúdez de Castro JM.	The fossil teeth of the Peking Man	2018	Sci Rep
Wang S.-M.; Lee C.-H.; Lin C.-H.; Chen G.-S.; Liu J.-C.; Li C.-H.	The growth of dental pulp stem cells in portland cement micro-environment	2015	Journal of Medical Sciences (Taiwan)
Fontana M, Gooch BF, Junger ML.	The Hall technique may be an effective treatment modality for caries in primary molars	2012	J Evid Based Dent Pract
Abuarqoub D.; Aslam N.; Zaza R.; Jafar H.; Zalloum S.; Atoom R.; Alshaer W.; Al-Mrahleh M.; Awidi A.	The Immunomodulatory and Regenerative Effect of Biodentine™ on Human THP-1 Cells and Dental Pulp Stem Cells: In Vitro Study	2022	BioMed Research International
Sinjari B.; Rexhepi I.; Santilli M.; D'addazio G.; Chiacchiaretta P.; Di Carlo P.; Caputi S.	The impact of covid-19 related lockdown on dental practice in central italy—outcomes of a survey	2020	International Journal of Environmental Research and Public Health
Chang C.-T., Liu S.-P., Muo C.-H., Liao Y.-F., Chiu K.-M., Tsai C.-H., Huang Y.-F.	The impact of dental therapy timelines and irradiation dosages on osteoradionecrosis in oral cancer patients: A population-based cohort study	2022	Oral Oncology
Moca A.E.; Ţig I.A.; Ciavoi G.; Iurcov R.; Şipoş L.R.; Todor L.	The Impact of the COVID-19 Pandemic on the Dental Emergency Service from Oradea, Romania: A Retrospective Study	2022	Healthcare (Switzerland)
Bolette A., Truong S., Guéders A., Geerts S.	The importance of pulp therapy in deciduous teeth	2016	Revue Medicale de Liege
Kallel I.; Douki N.; Amaidi S.; Ben Amor F.	The Incidence of Complications of Dental Trauma and Associated Factors: A Retrospective Study	2020	International Journal of Dentistry
Tian X.; Liu C.; Wang Z.	The induction of inflammation by the cGAS–STING pathway in human dental pulp cells: A laboratory investigation	2022	International Endodontic Journal
Petrović V.; Pejčić N.; Čakić S.	The influence of different therapeutic modalities and platelet rich plasma on apexogenesis - A preliminary study in monkeys	2013	Advances in Clinical and Experimental Medicine
Marek E.; Łagocka R.; Kot K.; Woźniak K.; Lipski M.	The influence of two forms of chlorhexidine on the accuracy of contemporary electronic apex locators	2019	BMC Oral Health
Chen D., Yue H., Liu S., Meng L., Yin W.	The introduction of team-based learning into the clinical pharmacology section of the endodontics clinical course	2022	Clinical and Experimental Pharmacology and Physiology
Doumani M.; Almutairi S.T.; Alshammari N.T.T.; Alshami A.N.; Alharbi A.S.; Habib A.	The knowledge about vital pulp therapy of permanent teeth among Saudi internship dentists and dental students	2020	Saudi Endodontic Journal
Edwards D, Bailey O, Stone S, Duncan H.	The management of deep caries in UK primary care: A nationwide questionnaire-based study	2021	Int Endod J
Careddu R, Plotino G, Cotti E, Duncan HF.	The management of deep carious lesions and the exposed pulp amongst members of two European endodontic societies: a questionnaire-based study	2021	Int Endod J
Beshkenadze E.; Chipashvili N.	The maxillary second molar - anatomical variations (case report)	2015	Georgian medical news

Sun P.; Guo Z.; Guo D.; Wang J.; Wu T.; Li T.; Liu J.; Liu X.	The Microbiota Profile Analysis of Combined Periodontal-Endodontic Lesions Using 16S rRNA Next-Generation Sequencing	2021	Journal of Immunology Research
Zanolli C, Martinón-Torres M, Bernardini F, Boschian G, Coppa A, Dreossi D, Mancini L, Martínez de Pinillos M, Martín-Francés L, Bermúdez de Castro JM, Tozzi C, Tuniz C, Macchiarelli R.	The Middle Pleistocene (MIS 12) human dental remains from Fontana Ranuccio (Latium) and Visogliano (Friuli-Venezia Giulia), Italy. A comparative high resolution endostructural assessment	2018	PLoS One
Acharya N., Humagain R., Dahal S., Kafle D.	The Need of Endodontic Therapy among Patients Attending Tertiary Care Center in Central Nepal	2022	Kathmandu University Medical Journal
Cintra L.T.A.; Benetti F.; Da Silva Facundo A.C.; Ferreira L.L.; Gomes-Filho J.E.; Ervolino E.; Rahal V.; Briso A.L.F.	The number of bleaching sessions influences pulp tissue damage in rat teeth	2013	Journal of Endodontics
Ye L, Liu L, Deng M, Liu N, Wu X, Dong Z.	The observation of surface morphology of enamal and temperature of dental pulp cavity in vitro after irradiated by Er:YAG laser	2012	Hua Xi Kou Qiang Yi Xue Za Zhi
Heidari E, Andiappan M, Banerjee A, Newton JT.	The oral health of individuals with dental phobia: a multivariate analysis of the Adult Dental Health Survey, 2009	2017	Br Dent J
Castagnola R.; Minciacchi I.; Rupe C.; Marigo L.; Grande N.M.; Contaldo M.; Pesce A.; Lajolo C.	The Outcome of Primary Root Canal Treatment in Postirradiated Patients: A Case Series	2020	Journal of Endodontics
Vu H.T.; Yoon J.-Y.; Park J.-H.; Lee H.-H.; Dashnyam K.; Kim H.-W.; Lee J.-H.; Shin J.-S.; Kim J.-B.	The Potential Application of Human Gingival Fibroblast-Conditioned Media in Pulp Regeneration: An In Vitro Study	2022	Cells
Elline E.; Ismiyatin K.; Indah Budhy T.; Bhardwaj A.	The potential of eggshell hydroxyapatite, collagen, and EGCG (HAp-Col-EGCG) scaffold as a pulp regeneration material	2022	Saudi Dental Journal
Dou L.; Vanschaayk M.M.; Zhang Y.; Fu X.; Ji P.; Yang D.	The prevalence of dental anxiety and its association with pain and other variables among adult patients with irreversible pulpitis	2018	BMC Oral Health
Verzak Z, Celap B, Modrić VE, Sorić P, Karlović Z.	The prevalence of idiopathic osteosclerosis and condensing osteitis in Zagreb population	2012	Acta Clin Croat
Zhang P.; Cui Z.; Li S.	The protective effects of S14G-humanin (HNG) against lipopolysaccharide (LPS)- induced inflammatory response in human dental pulp cells (hDPCs) mediated by the TLR4/MyD88/NF-κB pathway	2021	Bioengineered
Guo X.; Chen J.	The protective effects of saxagliptin against lipopolysaccharide (LPS)-induced inflammation and damage in human dental pulp cells	2019	Artificial Cells, Nanomedicine and Biotechnology
Palatyńska-Ulatowska A, Fernandes MC, Pietrzycka K, Koprowicz A, Klimek L, Souza RA, Pradebon M, de Figueiredo JAP.	The Pulp Stones: Morphological Analysis in Scanning Electron Microscopy and Spectroscopic Chemical Quantification	2021	Medicina (Kaunas)
Saito K.; Ohshima H.	The putative role of insulin-like growth factor (IGF)-binding protein 5 independent of IGF in the maintenance of pulpal homeostasis in mice	2019	Regenerative Therapy
Pustułka K.; Trzcionka A.; Dziedzic A.; Skaba D.; Tanasiewicz M.	The radiological assessment of root features and periodontal structures in endodontically treated teeth subjected to forces generated by fixed orthodontic appliances. A prospective, clinical cohort study	2021	Journal of Clinical Medicine
Abrami S.	The Radix Entomolaris: management of the distolingual root canal	2016	Giornale Italiano di Endodonzia
Kwon SR, Wertz PW, Dawson DV, Cobb DS, Denehy G.	The relationship of hydrogen peroxide exposure protocol to bleaching efficacy	2013	Oper Dent
Kamal E.M.; Nabih S.M.; Obeid R.F.; Abdelhameed M.A.	The reparative capacity of different bioactive dental materials for direct pulp capping; Zdolności reparacyjne bioaktywnych materiałów stosowanych w bezpośrednim pokryciu miazgi	2018	Dental and Medical Problems
Hara M, Horibe K, Mori H, Nakamura H.	The role of canonical Wnt signaling in dentin bridge formation	2021	J Oral Biosci
Tsai C.-L.; Hung S.-L.; Lee Y.-Y.; Ho Y.-C.; Yang S.-F.	The role of fibroblasts in the modulation of dental pulp inflammation	2022	Journal of the Formosan Medical Association

Irmaleny; Sitam S.; Pribadi S.; Dewi P.Y.	The Role of HBOT on Pulp Capping Treatment in Enhancing TGF-β Levels: Scoping Review	2022	Journal of International Dental and Medical Research
Zaky S.H.; Shehabeldin M.; Ray H.; Sfeir C.	The role of inflammation modulation in dental pulp regeneration	2021	European Cells and Materials
Choung HW, Lee JH, Lee DS, Choung PH, Park JC.	The role of preameloblast-conditioned medium in dental pulp regeneration	2013	J Mol Histol
Tscymbalystov A.V.; Kopytov A.A.; Kuzmina E.A.; Gontarev S.N.; Oganesyan A.A.	The study of destruction of the compositions restoring the dentition integrity under uniaxial tension	2016	International Journal of Pharmacy and Technology
Ludwig K.H.; Fontana M.; Vinson L.A.; Platt J.A.; Dean J.A.	The success of stainless steel crowns placed with the Hall technique :A retrospective study	2014	Journal of the American Dental Association
Ludwig KH, Fontana M, Vinson LA, Platt JA, Dean JA.	The success of stainless steel crowns placed with the Hall technique: a retrospective study	2014	J Am Dent Assoc
Wu S.; Liu Y.; Zhang H.; Lei L.	The Susceptibility to Calcium Hydroxide Modulated by the Essential walR Gene Reveals the Role for Enterococcus faecalis Biofilm Aggregation	2019	Journal of Endodontics
Monteiro J.; Ní Chaollaí A.; Duggal M.	The teaching of management of the pulp in primary molars across Europe	2017	European Archives of Paediatric Dentistry
Pan J.; Wang J.; Hao L.; Zhu G.; Nguyen D.N.; Li Q.; Liu Y.; Zhao Z.; Li Y.-P.; Chen W.	The Triple Functions of D2 Silencing in Treatment of Periapical Disease	2017	Journal of Endodontics
Janiszewska-Olszowska J, Socha A, Bińczak P.	The use of cortical screw anchorage for closing a space resulting from the loss of a lower molar--a case report	2013	Ann Acad Med Stetin
Garcez A.S.; Fregnani E.R.; Rodriguez H.M.; Nunez S.C.; Sabino C.P.; Suzuki H.; Ribeiro M.S.	The use of optical fiber in endodontic photodynamic therapy. Is it really relevant?	2013	Lasers in Medical Science
Bonsor S.J.	The use of the operating microscope in general dental practice. Part 2: If you can see it, you can treat it!	2015	Dental update
Doumani M.D.; Arnous W.A.; Alsafadi M.F.; Alnazer H.A.; Alanazi S.M.; Alotaibi K.S.; Al-Ammari A.I.	The vital pulp therapy of permanent teeth: A dental practitioner's perspective from Saudi Arabia	2020	Journal of International Society of Preventive and Community Dentistry
Jiang HW.	Theory and practice of minimally invasive endodontics	2016	Zhonghua Kou Qiang Yi Xue Za Zhi
Lu Y.; Liu Z.; Huang J.; Liu C.	Therapeutic effect of one-time root canal treatment for irreversible pulpitis	2019	Journal of International Medical Research
Kley P, Frentzen M, Küpper K, Braun A, Kecsmar S, Jäger A, Wolf M.	Thermotransduction and heat stress in dental structures during orthodontic debonding : Effectiveness of various cooling strategies	2016	J Orofac Orthop
Duncan HF, Bjørndal L, van der Sluis L, Rechenberg DK, Simon S, Cooper PR, Ricucci D, Galler K.	Third European Society of Endodontology (ESE) research meeting: ACTA, Amsterdam, The Netherlands, 26th October 2018: Deep caries and the exposed pulp: current and emerging therapeutic perspectives	2019	Int Endod J
Chompu-inwai P, Boonsongsawat K, Sastraruji T, Sophasri T, Mankaen S, Nondon S, Tunlek S, Katwong S.	Three Incomplete Caries Removal Techniques Compared Over Two Years in Primary Molars with Asymptomatic Deep Caries or Reversible Pulpitis	2015	Pediatr Dent
Markvart M, Bjørndal L, Darvann TA, Larsen P, Dalstra M, Kreiborg S.	Three-dimensional analysis of the pulp cavity on surface models of molar teeth, using X-ray micro-computed tomography	2012	Acta Odontol Scand
Diéguez-Pérez M, Ticona-Flores JM.	Three-Dimensional Analysis of the Pulp Chamber and Coronal Tooth of Primary Molars: An In Vitro Study	2022	Int J Environ Res Public Health
Hofmann E, Schmid M, Steinhäuser-Andresen S, Hirschfelder U.	Three-dimensional CT evaluation of oculoauriculovertebral spectrum patients use of Katsumata's asymmetry index	2016	J Orofac Orthop
Widbiller M.; Lindner S.R.; Buchalla W.; Eidt A.; Hiller K.-A.; Schmalz G.; Galler K.M.	Three-dimensional culture of dental pulp stem cells in direct contact to tricalcium silicate cements	2016	Clinical Oral Investigations
Sun W, Chen H, Zhong Y, Zhang W, Chu F, Li L, Chen Y, Wang X, Wang Q, Wang Y, Wei Y, Liu L, Xu Y.	Three-Dimensional Tooth Models with Pulp Cavity Enhance Dental Anatomy Education	2022	Anat Sci Educ
Kuharattanachai K, Jotikasthira D, Sirabanchongkran S, Srisuwan T, Rangsri W, Tripuwabhrut K.	Three-dimensional volumetric evaluation of dental pulp cavity/tooth ratio in anterior open bite malocclusion using cone beam computed tomography	2022	Clin Oral Investig
Raedel M., Hartmann A., Bohm S., Walter M.H.	Three-year outcomes of root canal treatment: Mining an insurance database	2015	Journal of dentistry

Kwon SR, Dawson DV, Wertz PW.	Time Course of Potassium Nitrate Penetration into the Pulp Cavity and the Effect of Penetration Levels on Tooth Whitening Efficacy	2016	J Esthet Restor Dent
Chinajitphan N.; Ajcharanukul O.; Kijsamanmith K.; Vongsavan N.; Matthews B.	Time-course of the effect of potassium oxalate in the treatment of hypersensitive dentine in man	2021	Archives of Oral Biology
Liu M.; Mu H.; Peng W.; Zhao L.; Hu W.; Jiang Z.; Gao L.; Cao X.; Li N.; Han J.	Time-dependent C5a and C5aR expression in dental pulp cells following stimulation with LTA and LPS	2019	International Journal of Molecular Medicine
Nam O.H.; Kim J.-H.; Choi S.C.; Kim Y.	Time-dependent response of human deciduous tooth-derived dental pulp cells treated with theraCal LC: Functional analysis of gene interactions compared to MTA	2020	Journal of Clinical Medicine
Santanna J.P.C.; Faria R.R.; Assad I.P.; Pinheiro C.C.G.; Aiello V.D.; Albuquerque-Neto C.; Bortolussi R.; Cestari I.A.; Maizato M.J.S.; Hernandez A.J.; Bueno D.F.; Fernandes T.L.	Tissue Engineering and Cell Therapy for Cartilage Repair: Preclinical Evaluation Methods	2022	Tissue Engineering - Part C: Methods
Ahmed G.M.; Abouauf E.A.; Abubakr N.; Dörfer C.E.; El-Sayed K.F.	Tissue Engineering Approaches for Enamel, Dentin, and Pulp Regeneration: An Update	2020	Stem Cells International
Singh R.; Singh R.; Kavita K.; Kommula A.; Kulkarni G.; Jois H.S.	To compare mineral trioxide aggregate, platelet-rich fibrin, and calcium hydroxide in teeth with irreversible pulpitis: A clinical study	2020	Journal of Pharmacy and Bioallied Sciences
Sahito A.H.; Kuhuawar S.R.; Jokhio A.L.; Tagar M.R.; Shaikh M.A.; Kalwar M.R.	To Determine Clinical Outcome of Platelet Rich Fibrin in Pulpotomy of Permanent Teeth in Irreversible Pulpitis	2022	Journal of the Liaquat University of Medical and Health Sciences
Plessas A.	To what extent do patients' racial characteristics affect our clinical decisions?	2019	Evid Based Dent
Lin J.-J.; Du Y.; Cai W.-K.; Kuang R.; Chang T.; Zhang Z.; Yang Y.-X.; Sun C.; Li Z.-Y.; Kuang F.	Toll-like receptor 4 signaling in neurons of trigeminal ganglion contributes to nociception induced by acute pulpitis in rats	2015	Scientific Reports
Ohara K.; Shimizu K.; Matsuura S.; Ogiso B.; Omagari D.; Asano M.; Tsuboi Y.; Shinoda M.; Iwata K.	Toll-like receptor 4 signaling in trigeminal ganglion neurons contributes tongue-referred pain associated with tooth pulp inflammation	2013	Journal of Neuroinflammation
Nagpal A.; Kremer K.L.; Hamilton-Bruce M.A.; Kaidonis X.; Milton A.G.; Levi C.; Shi S.; Carey L.; Hillier S.; Rose M.; Zacest A.; Takhar P.; Koblar S.A.	TOOTH (The Open study Of dental pulp stem cell Therapy in Humans): Study protocol for evaluating safety and feasibility of autologous human adult dental pulp stem cell therapy in patients with chronic disability after stroke	2016	International Journal of Stroke
Albuquerque M.T.P., Abreu L.C., Martim L., Münchow E.A., Nagata J.Y.	Tooth- and Patient-Related Conditions May Influence Root Canal Treatment Indication	2021	International Journal of Dentistry
Ali A.A.B., Qooz F.A., Mustafa O.S.	Tooth avulsion: Etiology and management	2020	Bahrain Medical Bulletin
Jain S, Nagi R, Daga M, Shandilya A, Shukla A, Parakh A, Laheji A, Singh R.	Tooth coronal index and pulp/tooth ratio in dental age estimation on digital panoramic radiographs-A comparative study	2017	Forensic Sci Int
Griffis E., Abd Alraheam I., Boushell L., Donovan T., Fasbinder D., Sulaiman T.A.	Tooth-cusp preservation with lithium disilicate onlay restorations: A fatigue resistance study	2022	Journal of Esthetic and Restorative Dentistry
Baccouche C, Ghoul-Mazgar S, Baaziz A, Said F, Ben Salem K.	Topography of the pulp chamber in the maxillary primary molars of a Tunisian children	2013	Indian J Dent Res
Emrick J.J.; von Buchholtz L.J.; Ryba N.J.P.	Transcriptomic Classification of Neurons Innervating Teeth	2020	Journal of Dental Research
Limone LE, Baratt RM.	Transcutaneous Lateral Alveolar Ostectomy for Standing Surgical Extraction of Mandibular First Molar in an 8-Year-Old Miniature Horse	2020	J Vet Dent
Bakri M.M.; Yahya F.; Munawar K.M.M.; Kitagawa J.; Hossain M.Z.	Transient receptor potential vanilloid 4 (TRPV4) expression on the nerve fibers of human dental pulp is upregulated under inflammatory condition	2018	Archives of Oral Biology
Peters O.A.	Translational opportunities in stem cell-based endodontic therapy: Where are we and what are we missing?	2014	Journal of Endodontics
Diogenes A.R.; Ruparel N.B.; Teixeira F.B.; Hargreaves K.M.	Translational science in disinfection for regenerative endodontics	2014	Journal of Endodontics
Sasaki R, Takanashi N, Chigono T.	Transplantation of a tooth involved in dentigerous cyst	2012	Eur J Paediatr Dent
Nito C.; Sowa K.; Nakajima M.; Sakamoto Y.; Suda S.; Nishiyama Y.; Nakamura-Takahashi A.; Nitahara-	Transplantation of human dental pulp stem cells ameliorates brain damage following acute cerebral ischemia	2018	Biomedicine and Pharmacotherapy

Kasahara Y.; Ueda M.; Okada T.; Kimura K.			
Feitosa M.L.T.; Sarmento C.A.P.; Bocabello R.Z.; Beltrão-Braga P.C.B.; Pignatari G.C.; Giglio R.F.; Miglino M.A.; Orlandin J.R.; Ambrósio C.E.	Transplantation of human immature dental pulp stem cell in dogs with chronic spinal cord injury	2017	Acta Cirurgica Brasileira
Kaptan R.F.; Haznedaroglu F.; Basturk F.B.; Kayahan M.B.	Treatment approaches and antibiotic use for emergency dental treatment in Turkey	2013	Therapeutics and Clinical Risk Management
Radwan A.; Kim S.G.	Treatment of a hypertaurodontic maxillary second molar in a patient with 10 taurodonts: A case report	2014	Journal of Endodontics
Saoud T.M.A.; Sigurdsson A.; Rosenberg P.A.; Lin L.M.; Ricucci D.	Treatment of a large cystlike inflammatory periapical lesion associated with mature necrotic teeth using regenerative endodontic therapy	2014	Journal of Endodontics
de Albuquerque M.S.; Rizuto A.V.; de Araújo Silva Tavares Á.F.; Nascimento A.S.; de Souza Araújo P.R.; de Lima E.A.; Braz da Silva R.	Treatment of an Acute Apical Abscess in a Patient With Autoimmune Hepatitis Taking Alendronate: A Case Report	2019	Journal of Endodontics
Arias Z.; Cazas I.; Siddiqui Y.D.; Yamashiro K.; Takashiba S.; Alam M.K.	Treatment of bucco-accessory root canal of a maxillary incisor with a combination of cone beam computed tomography and continuous supersonic wave condensation	2019	International Medical Journal
Kiefner P.; Connert T.; ElAyouti A.; Weiger R.	Treatment of calcified root canals in elderly people: a clinical study about the accessibility, the time needed and the outcome with a three-year follow-up	2017	Gerodontology
Ghezzi C.; Virzì M.; Schupbach P.; Broccaioli A.; Simion M.	Treatment of combined endodontic-periodontic lesions using guided tissue regeneration: Clinical case and histology	2012	International Journal of Periodontics and Restorative Dentistry
Kim J.C.; Park J.-C.; Kim S.-H.; Im G.-I.; Kim B.-S.; Lee J.-B.; Choi E.-Y.; Song J.-S.; Cho K.-S.; Kim C.-S.	Treatment of FGF-2 on stem cells from inflamed dental pulp tissue from human deciduous teeth	2014	Oral Diseases
Saoud T.M.; Martin G.; Chen Y.-H.M.; Chen K.-L.; Chen C.-A.; Songtrakul K.; Malek M.; Sigurdsson A.; Lin L.M.	Treatment of Mature Permanent Teeth with Necrotic Pulps and Apical Periodontitis Using Regenerative Endodontic Procedures: A Case Series	2016	Journal of Endodontics
Amoah G., Moola S., Newman-Nartey M.	Treatment of primary teeth using formocresol pulpotomy for patients attending a University Dental School Clinic: Best practice implementation project	2014	JBI Database of Systematic Reviews and Implementation Reports
Bergenholtz G, Axelsson S, Davidson T, Frisk F, Hakeberg M, Kvist T, Norlund A, Petersson A, Portenier I, Sandberg H, Tranæus S, Mejare I.	Treatment of pulps in teeth affected by deep caries - A systematic review of the literature	2013	Singapore Dent J
Ahmed T.; Kaushal N.	Treatment of Radicular Cyst with Marsupialization in Children: Report of Two Rare Cases	2022	International Journal of Clinical Pediatric Dentistry
Altner S., Ebel M., Ritschl V., Stamm T., Hirsch C., Bekes K.	Treatment of Severe Caries and Molar Incisor Hypomineralization and Its Influence on Oral Health-Related Quality of Life in Children: A Comparative Study	2022	International Journal of Environmental Research and Public Health
Dogan S., Ozturk G., Gumus H.	Treatment of severely decayed anterior primary teeth with short-post technique (Mushroom Restorations) under general anesthesia	2020	Nigerian journal of clinical practice
Al-Omiri M.K., Alqahtani N.M., Alahmari N.M., Hassan R.A., Al Nazeh A.A., Lynch E.	Treatment of symptomatic, deep, almost cariously exposed lesions using ozone	2021	Scientific reports
Linu S., Lekshmi M.S., Varunkumar V.S., Sam Joseph V.G.	Treatment Outcome Following Direct Pulp Capping Using Bioceramic Materials in Mature Permanent Teeth with Carious Exposure: A Pilot Retrospective Study	2017	Journal of endodontics
Makanjuola JO, Umesi DC, Oderinu OH.	TREATMENT OUTCOME OF MANUAL VERSUS ROTARY TECHNIQUES IN SINGLE-VISIT ENDODONTICS FOR PATIENTS IN A NIGERIAN TEACHING HOSPITAL: A RANDOMIZED CLINICAL TRIAL	2018	J West Afr Coll Surg
Linsuwanont P, Wimonsutthikul K, Pothimoke U, Santiwong B.	Treatment Outcomes of Mineral Trioxide Aggregate Pulpotomy in Vital Permanent Teeth with Carious Pulp Exposure: The Retrospective Study	2017	J Endod
Re D., Cerutti F., Consonni D., Gorni F.G.	Treatment planning of damaged teeth: to recover or to extract?	2017	Minerva stomatologica
Chai B, Tay B, Chow C, Fuss J, Krishnan U.	Treatment preferences for deep caries lesions among Australian dentists	2020	Aust Dent J

Stangvaltaite L.; Kundzina R.; Eriksen H.M.; Kerosuo E.	Treatment preferences of deep carious lesions in mature teeth: Questionnaire study among dentists in Northern Norway	2013	Acta Odontologica Scandinavica
Yoo B.J., Jung S.M., Lee H.N., Kim H.G., Chung J.H., Jeong J.H.	Treatment Strategy for Odontogenic Sinusitis	2021	American Journal of Rhinology and Allergy
Wong A.W.; Zhu X.; Zhang S.; Li S.K.; Zhang C.; Chu C.-H.	Treatment time for non-surgical endodontic therapy with or without a magnifying loupe	2015	BMC Oral Health
Liu F, Yang K, Wang P, Wu T, Li J, Guo Q.	Trends, Characteristics, and Success Rates of Treatment for Severe Early Childhood Caries Under General Anesthesia: A Retrospective Study in Northwest China	2021	J Clin Pediatr Dent
Huang S.-M.; Huang J.-Y.; Yu H.-C.; Su N.-Y.; Chang Y.-C.	Trends, demographics, and conditions of emergency dental visits in Taiwan 1997–2013: A nationwide population-based retrospective study	2019	Journal of the Formosan Medical Association
Santamaria R, Innes N.	Trial shows partial caries removal is an effective technique in primary molars	2014	Evid Based Dent
Huang J.I.-S.; Chang H.-H.; Lin C.-P.; Liao W.-C.; Kao C.-T.; Huang T.-H.	Trigeminocardiac reflex during non-surgical root canal treatment of teeth with irreversible pulpitis	2018	Journal of the Formosan Medical Association
Derchi G.; Marchio V.; Borgia V.; Özcan M.; Giuca M.R.; Barone A.	Twelve-year longitudinal clinical evaluation of bonded indirect composite resin inlays	2019	Quintessence International
Hosoya A, Yukita A, Yoshiba K, Yoshiba N, Takahashi M, Nakamura H.	Two distinct processes of bone-like tissue formation by dental pulp cells after tooth transplantation	2012	J Histochem Cytochem
Shakouie S, Mokhtari H, Ghasemi N, Gholizadeh S.	Two-rooted maxillary first molars with two canals: a case series	2013	Iran Endod J
You WZ, Dou GL, Xia B.	Two-year outcomes and the influence factors of indirect pulp treatment in primary teeth: a retrospective study	2019	Beijing Da Xue Xue Bao Yi Xue Ban
Khorakian F, Mazhari F, Asgary S, Sahebnasagh M, Alizadeh Kaseb A, Movahhed T, Sarraf Shirazi AR.	Two-year outcomes of electrosurgery and calcium-enriched mixture pulpotomy in primary teeth: a randomised clinical trial	2014	Eur Arch Paediatr Dent
Haripriya S.	Type of caries excavation performed during pulp capping procedures in adult population – institutional-based study	2020	International Journal of Clinical Dentistry
Nazemisalman B.; Farsadeghi M.; Sokhansanj M.	Types of lasers and their applications in pediatric dentistry	2015	Journal of Lasers in Medical Sciences
El-Bialy T.; Alhadlaq A.; Wong B.; Kucharski C.	Ultrasound effect on neural differentiation of gingival stem/progenitor cells	2014	Annals of Biomedical Engineering
Patel N, Patel S, Cotti E, Bardini G, Mannocci F.	Unconscious Racial Bias May Affect Dentists' Clinical Decisions on Tooth Restorability: A Randomized Clinical Trial	2019	JDR Clin Trans Res
Aziz K.; Hoover T.; Sidhu G.	Understanding root resorption with diagnostic imaging.	2014	Journal of the California Dental Association
Hallak B., Teiga P., Bühler J.-P., Bouayed S.	Unexpected foreign body induced refractory maxillary sinusitis	2021	Clinical Case Reports
Gondak R.O.; Rocha A.C.; Neves Campos J.G.; Vargas P.A.; De Almeida O.P.; Lopes M.A.; Santos-Silva A.R.	Unicystic ameloblastoma mimicking apical periodontitis: A case series	2013	Journal of Endodontics
Marchan S.M., Coppin E., Balkaran R.	Unmet Dental Treatment Needs and Barriers to Dental Care of Patients with Special Needs Attending a Dental Teaching Hospital	2022	Portuguese Journal of Public Health
Al-Amery SM, Nambiar P, John J, Purmal K, Ngeow WC, Mohamed NH, Vellayan S.	Unusual Dental Morphology in a Chimpanzee: A Case Report Utilizing Cone-Beam Computed Tomography	2018	J Vet Dent
Ashqar N.M., Ali F.M.	Unusual foreign object in a tooth: A case report and review of literature	2019	Clinics and Practice
Ricucci D.; Milovidova I.; Siqueira J.F., Jr.	Unusual Location of Dens Invaginatus Causing a Difficult-to-Diagnose Pulpal Involvement	2020	Journal of Endodontics
Nie S.-C.; Yang K.; Luan N.-N.; Lian X.-L.; Dai X.-H.; Liang S.-X.; Yan Y.-B.	Unveiling the Differences in Biological Properties of Dental Pulp Stem Cells from Normal and Inflamed Pulp: A Comprehensive Comparative Study	2022	Medical Science Monitor
Short R.	Update on apexogenesis: case reports: achieving predictable root maturation in young patients	2013	Dent Today
Kaneko T, Chokechanachaisakul U, Kawamura J, Yamanaka Y, Ito T, Sunakawa M, Suda H, Okiji T.	Up-regulation of p38 mitogen-activated protein kinase during pulp injury-induced glial cell/neuronal interaction in the rat thalamus	2013	J Endod
Shang Y.; Li Y.; Yang Z.; Zhou Z.	Upregulation of TACAN in the trigeminal ganglion affects pain transduction in acute pulpitis	2022	Archives of Oral Biology

Mahmoudi J.; Sabermarouf B.; Baradaran B.; Sadat-Hatamnezhad L.; Shotorbani S.S.	Up-regulation of TLR2 and TLR4 in high mobility group Box1-stimulated macrophages in pulpitis patients	2017	Iranian Journal of Basic Medical Sciences
Lee P.R.; Lee J.-H.; Park J.M.; Oh S.B.	Upregulation of toll-like receptor 2 in dental primary afferents following pulp injury	2021	Experimental Neurobiology
Gok E, Fedakar R, Kafa IM.	Usability of dental pulp visibility and tooth coronal index in digital panoramic radiography in age estimation in the forensic medicine	2020	Int J Legal Med
Lamé G.; Yannou B.; Cluzel F.	Usage-driven problem design for radical innovation in healthcare	2018	BMJ Innovations
Sim R.R.; Stringer E.; Donovan D.; Chappell R.; Flora P.; Hall J.; Pillay S.; Willis B.G.; McCain S.	USE of COMPOSITE MATERIALS AS A COMPONENT of TUSK FRACTURE MANAGEMENT in AN ASIAN ELEPHANT (ELEPHAS MAXIMUS) and AN AFRICAN ELEPHANT (LOXODONTA AFRICANA)	2017	Journal of Zoo and Wildlife Medicine
Gulsahi A.; Ates U.; Tirali R.E.; Cehreli S.B.	Use of cone-beam computed tomography in diagnosis of an otherwise undetected periapical lesion in an anomalous tooth	2014	Oral Radiology
de Oliveira E.E.G.; Pinheiro T.P.; Miyahara L.A.N.; Maneschy A.G.F.; Pontes H.A.R.; Khayat A.I.	Use of endodontic guide for resolution of calcified root canals: case report	2019	Dental Press Endodontics
Coll J.A.; Dhar V.; Vargas K.; Chen C.-Y.; Crystal Y.O.; AlShamali S.; Marghalani A.A.	Use of Non-Vital Pulp Therapies in Primary Teeth	2020	Pediatric dentistry
Kovach I.V.; Dychko E.N.; Kopchak O.V.; Buniatian Kh.A.; Khotimska Yu.V.; Gargin V.V.; Lavreniuk Y.V.	Use of odontotropic material in treatment of traumatic pulpitis in experiment.; Застосування одонтотропного матеріалу при лікуванні травматичного пульпіту в експерименті.	2021	Medicni Perspektivi
Biezanek T.; Strycharz-Dudziak M.; Bachanek T.	Use of ozone in endodontic treatment - Clinical observations; Zastosowanie ozonu w leczeniu endodontycznym - Obserwacje kliniczne	2012	Dental and Medical Problems
Paterson A, Franco V, Patel S, Foschi F.	Use of preoperative cone-beam computed tomography to aid in establishment of endodontic working length: A systematic review and meta-analysis	2020	Imaging Sci Dent
Luz Segundo A.C.S.; Silva R.V.; Pereira R.P.; Nunes E.	Use of reciproc in mandibular premolar with double-curved root canals: A case report	2015	Dental Press Endodontics
Silva R.V.; Santos F.L.A.A.; Ravazzi T.P.Q.; Rodrigues C.T.; Pereira R.P.	Use of the Reciproc Blue instrument associated with photodynamic therapy: case report	2021	Dental Press Endodontics
Nawaya FR, Burhan AS.	Use of the Tooth Coronal Pulp Index for Recognition of the Pubertal Growth Period	2016	J Contemp Dent Pract
Dhar V, Marghalani AA, Crystal YO, Kumar A, Ritwik P, Tulunoglu O, Graham L.	Use of Vital Pulp Therapies in Primary Teeth with Deep Caries Lesions	2017	Pediatr Dent
Mathieu S.; Jeanneau C.; Sheibat-Othman N.; Kalaji N.; Fessi H.; About I.	Usefulness of controlled release of growth factors in investigating the early events of dentin-pulp regeneration	2013	Journal of Endodontics
Rashkova M.; MItova N.; Lazarova Z.; Gateva N.	Using a dental operating microscope in the treatment of reversible pulpitis in primary teeth	2020	Cumhuriyet Dental Journal
Gurudutt Nayak B.; Singh I.	Using spiral computed tomography for endodontic management of a mandibular first molar with a middle mesial canal: A case report	2013	General Dentistry
Da Silva K, Kunzel C, Yoon RK.	Utilization of emergency services for non-traumatic dental disease	2013	J Clin Pediatr Dent
Kumar V.; Price J.B.; Brooks J.K.	Value of Performing Routine Vascular Mapping Synchronous with Radiographic Assessment of Endodontic Lesions: Case Series	2021	Journal of Endodontics
Kim G., Lee J.	Variability of dental abnormalities according to the timing of anticancer therapy: A report of two cases	2022	Pediatric Dental Journal
Rønneberg A, Skaare AB, Hofmann B, Espelid I.	Variation in caries treatment proposals among dentists in Norway: the best interest of the child	2017	Eur Arch Paediatr Dent
Usha G, Muddappa SC, Venkitachalam R, Singh V P P, Rajan RR, Ravi AB.	Variations in root canal morphology of permanent incisors and canines among Asian population: A systematic review and meta-analysis	2021	J Oral Biosci
Al-Hassiny A.; Hussaini H.; Milne T.; Seo B.; Rich A.M.; Friedlander L.T.	Vascularity and angiogenic signaling in the dentine-pulp complex of immature and mature permanent teeth	2019	European Endodontic Journal

Liang Q.; Liang C.; Liu X.; Xing X.; Ma S.; Huang H.; Liang C.; Liu L.; Liao L.; Tian W.	Vascularized dental pulp regeneration using cell-laden microfiber aggregates	2022	Journal of Materials Chemistry B
Yuan X.; Yuan Z.; Wang Y.; Wan Z.; Wang X.; Yu S.; Han J.; Huang J.; Xiong C.; Ge L.; Cai Q.; Zhao Y.	Vascularized pulp regeneration via injecting simvastatin functionalized GelMA cryogel microspheres loaded with stem cells from human exfoliated deciduous teeth	2022	Materials Today Bio
Haueisen H.; Gärtner K.; Kaiser L.; Trohorsch D.; Heidemann D.	Vertical root fracture: Prevalence, etiology, and diagnosis	2013	Quintessence International
Zhong S.; Naqvi A.; Bair E.; Nares S.; Khan A.A.	Viral MicroRNAs Identified in Human Dental Pulp	2017	Journal of Endodontics
Zafar S.; Lai Y.; Sexton C.; Siddiqi A.	Virtual Reality as a novel educational tool in pre-clinical paediatric dentistry training: Students' perceptions	2020	International Journal of Paediatric Dentistry
Hernández Vigueras S.; Donoso Zúñiga M.; Jané-Salas E.; Salazar Navarrete L.; Segura-Egea J.J.; Velasco-Ortega E.; López-López J.	Viruses in pulp and periapical inflammation: a review	2016	Odontology
Szopinski KT, Regulski P.	Visibility of dental pulp spaces in dental ultrasound	2014	Dentomaxillofac Radiol
Chisini L.A.; Conde M.C.M.; Correa M.B.; Dantas R.V.F.; Silva A.F.; Pappen F.G.; Demarco F.F.	Vital pulp therapies in clinical practice: Findings from a survey with dentist in southern Brazil	2015	Brazilian Dental Journal
Hanna SN, Perez Alfayate R, Prichard J.	Vital Pulp Therapy an Insight Over the Available Literature and Future Expectations	2020	Eur Endod J
Asgary S.; Nourzadeh M.; Verma P.; Hicks M.L.; Nosrat A.	Vital Pulp Therapy as a Conservative Approach for Management of Invasive Cervical Root Resorption: A Case Series	2019	Journal of Endodontics
Vafaei A, Nikookhesal M, Erfanparast L, Løvschall H, Ranjkesh B.	Vital pulp therapy following pulpotomy in immature first permanent molars with deep caries using novel fast-setting calcium silicate cement: A retrospective clinical study	2022	J Dent
Leong D.J.X., Yap A.U.	Vital pulp therapy in carious pulp-exposed permanent teeth: an umbrella review	2021	Clinical oral investigations
Luotonen N.; Kuntsi-Vaattovaara H.; Sarkiala-Kessel E.; Junnila J.J.T.; Laitinen-Vapaavuori O.; Verstraete F.J.M.	Vital pulp therapy in dogs: 190 cases (2001-2011)	2014	Journal of the American Veterinary Medical Association
Gizani S, Seremidi K, Stratigaki E, Tong HJ, Duggal M, Kloukos D.	Vital Pulp Therapy in Primary Teeth with Deep Caries: An Umbrella Review	2021	Pediatr Dent
Kisby L.	Vital Pulp Therapy in Primary Teeth: An Update	2016	Dent Today
Wang F.-M.; Charunmethee P.; Augsburger R.A.; Gutmann J.L.	Vital pulp therapy of a dens evaginatus-affected tooth with an immature apex and inflamed pulpal and periapical tissue: A case report	2020	General Dentistry
Asgary S, Kemal Çalışkan M.	Vital Pulp Therapy of a Mature Molar with Concurrent Hyperplastic Pulpitis, Internal Root Resorption and Periradicular Periodontitis: A Case Report	2015	Iran Endod J
Iaculli F, Rodríguez-Lozano FJ, Briseño-Marroquín B, Wolf TG, Spagnuolo G, Rengo S.	Vital Pulp Therapy of Permanent Teeth with Reversible or Irreversible Pulpitis: An Overview of the Literature	2022	J Clin Med
Ashraf H, Rahmati A, Amini N.	Vital Pulp Therapy with Calcium-Silicate Cements: Report of Two Cases	2017	Iran Endod J
Strassler H.E.; Levin R.	Vital pulp therapy with pulp capping.	2012	Dentistry today
Ricucci D, Siqueira JF Jr, Li Y, Tay FR.	Vital pulp therapy: histopathology and histobacteriology-based guidelines to treat teeth with deep caries and pulp exposure	2019	J Dent
Matoug-Elwerfelli M, ElSheshtawy AS, Duggal M, Tong HJ, Nazzal H.	Vital pulp treatment for traumatized permanent teeth: A systematic review	2022	Int Endod J
Venkatesh S, Ajmera S, Ganeshkar SV.	Volumetric pulp changes after orthodontic treatment determined by cone-beam computed tomography	2014	J Endod
Hamouda M, Deery C.	What is the best caries removal strategy for primary molars?	2021	Evid Based Dent
Hunter DJ, Bardet C, Mouraret S, Liu B, Singh G, Sadoine J, Dhamdhere G, Smith A, Tran XV, Joy A, Rooker S, Suzuki S, Vuorinen A, Miettinen S, Chaussain C, Helms JA.	Wnt Acts as a Prosurvival Signal to Enhance Dentin Regeneration	2015	J Bone Miner Res
Vijaykumar A.; Root S.H.; Mina M.	Wnt/β-Catenin Signaling Promotes the Formation of Preodontoblasts In Vitro	2021	Journal of Dental Research

Zhao Y.; Wang C.-L.; Li R.-M.; Hui T.-Q.; Su Y.-Y.; Yuan Q.; Zhou X.-D.; Ye L.	Wnt5a promotes inflammatory responses via nuclear factor κB (NF-κB) and mitogen-activated protein kinase (MAPK) pathways in human dental pulp cells	2014	Journal of Biological Chemistry
Zhao Y, Yuan X, Liu B, Tulu US, Helms JA.	Wnt-Responsive Odontoblasts Secrete New Dentin after Superficial Tooth Injury	2018	J Dent Res
Smadi L., Sumadi A.A.	Women's oral and dental health aspects in humanitarian missions and disasters: Jordanian experience	2016	American journal of disaster medicine
Pandranki J.; Vanga N.R.V.; Chandrabhatla S.K.	Zinc oxide eugenol and Endoflas pulpectomy in primary molars: 24-month clinical and radiographic evaluation	2018	Journal of Indian Society of Pedodontics and Preventive Dentistry
Gonzalez-Lara A, Ruiz-Rodriguez MS, Pierdant-Perez M, Garrocho-Rangel JA, Pozos-Guillen AJ.	Zinc Oxide-Eugenol Pulpotomy in Primary Teeth: A 24-Month Follow-up	2016	J Clin Pediatr Dent
Schaffner M., Stich H., Megert B., Lussi A.		2016	Swiss dental journal
