# Supplementary material for: Partial Pulpotomy in Young Permanent Teeth: A Systematic Review and Meta-Analysis
Source: Children (Basel). 2023 Aug 24;10(9):1447. doi: 10.3390/children10091447 (PMC10527738; doi:10.3390/children10091447)
Supplement: Supplementary file 1 [file children-10-01447-s001.zip › Table S5.pdf]

Table S5. Papers discarded after full text evaluation.

Author Names	Title	Year	Journal	Reason of exclusion
Abiraamasri B.L.; Deepa D.G.	Comparison of ferric sulphate and calcium hydroxide as a pulpotomy agent	2018	Research Journal of Pharmacy and Technology	Not specifically about partial pulpotomy
Abuelniel G.M.; Duggal M.S.; Duggal S.; Kabel N.R.	Evaluation of Mineral Trioxide Aggregate and Biodentine as pulpotomy agents in immature first permanent molars with carious pulp exposure: A randomised clinical trial	2021	European Journal of Paediatric Dentistry	Not specifically about partial pulpotomy
Ahmed M.I.; El Hilaly Mohamed Eid G.; Youssef H.A.	Clinical and Radiographic Assessments of Potassium Nitrate in Polycarboxylate Versus Mineral Trioxide Aggregate as Pulpotomy Biomaterials in Immature Mandibular First Permanent Molars: A Randomized Clinical Trial	2021	Journal of Endodontics	Not specifically about partial pulpotomy
Al-Abdullah A., Edris S., Abu Hasna A., De Carvalho L.S., Al-Nahlawi T.	The Effect of Aloe vera and Chlorhexidine as Disinfectants on the Success of Selective Caries Removal Technique: A Randomized Controlled Trial	2022	International Journal of Dentistry	Not specifically about partial pulpotomy
Alawwad M., Altinawi M., Rekab M.S., Kosyreva T., Almokaddam H., Katbeh I.	A comparative clinical radiological study using platelet Rich Fibrin and MTA in pulpotomy of first permanent immature molars	2020	Journal of Clinical and Diagnostic Research	Not specifically about partial pulpotomy
Alinejad D., Amrollahi N.	Report a case of beauty regeneration in a child with premature tooth decay	2019	Journal of Babol University of Medical Sciences	Primary theeth
Aljandan B.; Alhassan H.; Saghah A.; Rasheed M.; Ali A.A.	The effectiveness of using different pulp-capping agents on the healing response of the pulp	2012	Indian Journal of Dental Research	Not specifically about partial pulpotomy
Alqaderi H.E., Al-Mutawa S.A., Qudeimat M.A.	MTA pulpotomy as an alternative to root canal treatment in children's permanent teeth in a dental public health setting	2014	Journal of dentistry	Not specifically about partial pulpotomy
Al-Saudi K.W.; Nabih S.M.; Farghaly A.M.; AboHager E.A.-A.	Pulpal repair after direct pulp capping with new bioceramic materials: A comparative histological study	2019	Saudi Dental Journal	Not specifically about partial pulpotomy
Alsofi L.; Khalil W.; Binmadi N.O.; Al-Habib M.A.; Alharbi H.	Pulpal and periapical tissue response after direct pulp capping with endosequence root repair material and low-level laser application	2022	BMC Oral Health	Not specifically about partial pulpotomy
Asgary S, Hassanizadeh R, Torabzadeh H, Eghbal MJ.	Treatment Outcomes of 4 Vital Pulp Therapies in Mature Molars	2018	J Endod	Not specifically about partial pulpotomy
Asgary S.	Micro-computed Tomography Assessment of Full Pulpotomy in a Mature Molar after Five Years: A Case Report	2022	Iranian Endodontic Journal	Not specifically about partial pulpotomy
Asgary S.; Ahmadyar M.	Can miniature pulpotomy procedure improve treatment outcomes of direct pulp capping?	2012	Medical Hypotheses	Not specifically about partial pulpotomy
Ashwin Kumar S.P., Haripriya S., Dharman S.	Association of age, gender and teeth distribution in patients undergoing treatment for management of open apex	2020	International Journal of Pharmaceutical Research	Not specifically about partial pulpotomy
Azimi S.; Fazlyab M.; Sadri D.; Saghiri M.A.; Khosravanifard B.; Asgary S.	Comparison of pulp response to mineral trioxide aggregate and a bioceramic paste in partial pulpotomy of sound human premolars: A randomized controlled trial	2014	International Endodontic Journal	Intentional partial pulpotomy

Bagheri M.; Khimani H.; Pishbin L.; Shahabinejad H.	Effect of pulpotomy procedures with mineral trioxide aggregate and dexamethasone on post-endodontic pain in patients with irreversible pulpitis: A randomized clinical trial	2019	European Endodontic Journal	Not specifically about partial pulpotomy
Bakhtiar H.; Aminishakib P.; Ellini M.R.; Mosavi F.; Abedi F.; Esmailian S.; Esnaashari E.; Nekoofar M.H.; Sezavar M.; Mesgarzadeh V.; About I.	Dental Pulp Response to RetroMTA after Partial Pulpotomy in Permanent Human Teeth	2018	Journal of Endodontics	Not specifically about partial pulpotomy
Ballal N.V., Duncan H.F., Rai N., Jalan P., Zehnder M.	Sodium hypochlorite reduces postoperative discomfort and painful early failure after carious exposure and direct pulp capping—initial findings of a randomized controlled trial	2020	Journal of Clinical Medicine	Not specifically about partial pulpotomy
Baraka M., Tekeya M., Bakry N.S., Fontana M.	Twelve-month randomized controlled trial of 38% silver diamine fluoride with or without potassium iodide in indirect pulp capping of young permanent molars	2022	Journal of the American Dental Association (1939)	Not specifically about partial pulpotomy
Barngkgei I.H.; Halboub E.S.; Alboni R.S.	Pulpotomy of symptomatic permanent teeth with carious exposure using mineral trioxide aggregate	2013	Iranian Endodontic Journal	Not specifically about partial pulpotomy
Bjørndal L, Fransson H, Bruun G, Markvart M, Kjældgaard M, Näsman P, Hedenbjörk-Lager A, Dige I, Thordrup M.	Randomized Clinical Trials on Deep Carious Lesions: 5-Year Follow-up	2017	J Dent Res	Not specifically about partial pulpotomy
Blagojević D.; Petrović B.; Marković D.; Vujkov S.; Rihter I.D.	Pulp vitality preservation after traumatic dental injuries to permanent teeth	2013	Medicinski pregled	Not specifically about partial pulpotomy
Brizuela C., Ormeño A., Cabrera C., Cabezas R., Silva C.I., Ramírez V., Mercade M.	Direct Pulp Capping with Calcium Hydroxide, Mineral Trioxide Aggregate, and Biodentine in Permanent Young Teeth with Caries: A Randomized Clinical Trial	2017	Journal of endodontics	Not specifically about partial pulpotomy
Brodén J, Davidson T, Fransson H.	Cost-effectiveness of pulp capping and root canal treatment of young permanent teeth	2019	Acta Odontol Scand	Not specifically about partial pulpotomy
Cengiz E.; Yilmaz H.G.	Efficacy of erbium, chromium-doped:yttrium, scandium, gallium, and garnet laser irradiation combined with resin-based tricalcium silicate and calcium hydroxide on direct pulp capping: A randomized clinical trial	2016	Journal of Endodontics	Not specifically about partial pulpotomy
Cunha N.N.D.O.; Junqueira M.A.; Cosme-Silva L.; Santos L.D.S.T.; De Oliveira G.A.V.; Neto R.T.M.; Nogueira D.A.; Brigagão M.R.P.L.; Moretti A.B.D.S.	Expression of matrix metalloproteinases-8 and myeloperoxidase in pulp tissue after pulpotomy with calcium silicate cements	2021	Pesquisa Brasileira em Odontopediatria e Clinica Integrada	Not specifically about partial pulpotomy
Daniele L.	Mineral Trioxide Aggregate (MTA) direct pulp capping: 10 years clinical results	2017	Giornale Italiano di Endodonzia	Not specifically about partial pulpotomy
de Almeida Santo Ramos I.F.; Biz M.T.; Paulino N.; Scremin A.; Della Bona A.; Barletta F.B.; de Figueiredo J.A.P.	Histopathological analysis of corticosteroid-antibiotic preparation and propolis paste formulation as intracanal medication after pulpectomy: An in vivo study	2012	Journal of Applied Oral Science	Not specifically about partial pulpotomy
Durmus N, Tok YT, Kaya S, Akcay M.	Effectiveness of the ozone application in two-visit indirect pulp therapy of permanent molars with deep carious lesion: a randomized clinical trial	2019	Clin Oral Investig	Not specifically about partial pulpotomy
Eggmann F, Gasser TJW, Hecker H, Amato M, Weiger R, Zaugg LK.	Partial pulpotomy without age restriction: a retrospective assessment of permanent teeth with carious pulp exposure	2022	Clin Oral Investig	Wrong design
Galani M, Tewari S, Sangwan P, Mittal S, Kumar V, Duhan J.	Comparative Evaluation of Postoperative Pain and Success Rate after Pulpotomy and Root Canal Treatment in Cariously Exposed Mature Permanent Molars: A Randomized Controlled Trial	2017	J Endod	Not specifically about partial pulpotomy

Ghoddusi J.; Shahrami F.; Alizadeh M.; Kianoush K.; Forghani M.	Clinical and radiographic evaluation of vital pulp therapy in open apex teeth with MTA and ZOE.	2012	The New York state dental journal	Not specifically about partial pulpotomy
Guan X., Zhou Y., Yang Q., Zhu T., Chen X., Deng S., Zhang D.	Vital pulp therapy in permanent teeth with irreversible pulpitis caused by caries: A prospective cohort study	2021	Journal of Personalized Medicine	Wrong design
Hashem D, Mannocci F, Patel S, Manoharan A, Brown JE, Watson TF, Banerjee A.	Clinical and radiographic assessment of the efficacy of calcium silicate indirect pulp capping: a randomized controlled clinical trial	2015	J Dent Res	Not specifically about partial pulpotomy
Hashem D., Mannocci F., Patel S., Manoharan A., Watson T.F., Banerjee A.	Evaluation of the efficacy of calcium silicate vs. glass ionomer cement indirect pulp capping and restoration assessment criteria: a randomised controlled clinical trial-2-year results	2019	Clinical oral investigations	Not specifically about partial pulpotomy
Hernandéz-Gatón P., Serrano C.R., Nelson Filho P., De Castañeda E.R., Lucisano M.P., Silva R.A., Silva L.A.	Stepwise Excavation Allows Apexogenesis in Permanent Molars with Deep Carious Lesions and Incomplete Root Formation	2015	Caries research	Not specifically about partial pulpotomy
Hilton T.J.; Ferracane J.L.; Mancl L.	Comparison of CaOH with MTA for Direct Pulp Capping: A PBRN Randomized Clinical Trial	2013	Journal of Dental Research	Not specifically about partial pulpotomy
Huth K.C.; Hajek-Al-Khatar N.; Wolf P.; Ilie N.; Hickel R.; Paschos E.	Long-term effectiveness of four pulpotomy techniques: 3-year randomised controlled trial	2012	Clinical Oral Investigations	Not specifically about partial pulpotomy
Jespersen J.J., Hellstein J., Williamson A., Johnson W.T., Qian F.	Evaluation of dental pulp sensibility tests in a clinical setting	2014	Journal of endodontics	Not specifically about partial pulpotomy
Kalyan K.S.D.R., Vinay C., Arunbhupathi, Uloopi K.S., Chandrasekhar R., RojaRamya K.S.	Preclinical Evaluation and Clinical Trial of Chlorhexidine Polymer Scaffold for Vital Pulp Therapy	2019	The Journal of clinical pediatric dentistry	Primary theeth
Keerthana T., Sindhu R., Deepak S.	Comparative analysis of the success rate of direct and indirect pulp capping procedures-a retrospective analysis	2020	International Journal of Research in Pharmaceutical Sciences	Not specifically about partial pulpotomy
Koc Vural U, Kiremitci A, Gokalp S.	Randomized Clinical Trial to Evaluate MTA Indirect Pulp Capping in Deep Caries Lesions After 24-Months	2017	Oper Dent	Not specifically about partial pulpotomy
Kumar K.; Naz S.; Memon P.; Arshad M.M.; Rajput F.; Shaikh M.A.	Efficacy of bio-dentine and mineral trioxide aggregate in pulpotomies of cariously exposed vital permanent teeth	2020	Rawal Medical Journal	Not specifically about partial pulpotomy
Li J., Wang E.-H., Wang Y.	Effectiveness and safety of pulp regeneration and revascularization therapy for pulpal necrosis of the immature permanent tooth: Study protocol for a single-center, randomized, controlled, clinical trial	2017	Chinese Journal of Tissue Engineering Research	Not specifically about partial pulpotomy
Liu S.Y., Gong W.Y., Liu M.Q., Long Y.Z., Dong Y.M.	Clinical efficacy observation of direct pulp capping using iRoot BP Plus therapy in mature permanent teeth with carious pulp exposure	2020	Zhonghua kou qiang yi xue za zhi = Zhonghua kouqiang yixue zazhi = Chinese journal of stomatology	Not specifically about partial pulpotomy
Mahapatra J., Nikhade P.P., Sukhtankar S.	Comparative evaluation of the efficacy of light cure calcium hydroxide and theracal LC as indirect pulp capping materials in patients with deep carious lesion-An interventional study	2020	European Journal of Molecular and Clinical Medicine	Not specifically about partial pulpotomy
Maltz M, Jardim JJ, Mestrinho HD, Yamaguti PM, Podestá K, Moura MS, de Paula LM.	Partial removal of carious dentine: a multicenter randomized controlled trial and 18-month follow-up results	2013	Caries Res	Not specifically about partial pulpotomy
Mangione F.; EzEldeen M.; Bardet C.; Lesieur J.; Bonneau M.; Decup F.; Salmon B.; Jacobs R.; Chaussain C.; Opsahl-Vital S.	Implanted Dental Pulp Cells Fail to Induce Regeneration in Partial Pulpotomies	2017	Journal of Dental Research	Not specifically about partial pulpotomy

Mathur V.P., Dhillon J.K., Logani A., Kalra G.	Evaluation of indirect pulp capping using three different materials: A randomized control trial using cone-beam computed tomography	2016	Indian journal of dental research : official publication of Indian Society for Dental Research	Not specifically about partial pulpotomy
Moura-Netto C, Kerber Tedesco T, Werner S, Volpi Mello-Moura AC, Prócida Raggio D.	Influence of preoperative pain in the success rate of indirect pulp capping: a retrospective study	2017	Minerva Stomatol	Not specifically about partial pulpotomy
Mutluay M, Arıkan V, Sarı S, Kısa Ü.	Does Achievement of Hemostasis After Pulp Exposure Provide an Accurate Assessment of Pulp Inflammation?	2018	Pediatr Dent	Primary theeth
Noor A., Afzal J., Mahmood A., Ullah M.S., Munawar M., Mehdi M.M.	Effectiveness of Calcium Hydroxide (CaOH) as agent for Direct Pulp Capping in the Mandibular Molars	2021	Pakistan Journal of Medical and Health Sciences	Not specifically about partial pulpotomy
Nosrat A, Seifi A, Asgary S.	Pulpotomy in caries-exposed immature permanent molars using calcium-enriched mixture cement or mineral trioxide aggregate: a randomized clinical trial	2013	Int J Paediatr Dent	Not specifically about partial pulpotomy
Odabaş M.E.; Alaçam A.; Sillelioğlu H.; Deveci C.	Clinical and radiographic success rates of mineral trioxide aggregate and ferric sulphate pulpotomies performed by dental students	2012	European Journal of Paediatric Dentistry	Not specifically about partial pulpotomy
Oz F.D., Bolay S., Bayazit E.O., Bicer C.O., Isikhan S.Y.	Long-term survival of different deep dentin caries treatments: A 5-year clinical study	2019	Nigerian journal of clinical practice	Not specifically about partial pulpotomy
Parinyaprom N, Nirunsittirat A, Chuveera P, Na Lampang S, Srisuwan T, Sastraruji T, Bua-On P, Simprasert S, Khoipanich I, Sutharaphan T, Theppimarn S, Ue-Srichai N, Tangtrakooljaroen W, Chompu-Inwai P.	Outcomes of Direct Pulp Capping by Using Either ProRoot Mineral Trioxide Aggregate or Biodentine in Permanent Teeth with Carious Pulp Exposure in 6- to 18-Year-Old Patients: A Randomized Controlled Trial	2018	J Endod	Not specifically about partial pulpotomy
Pratima B, Chandan GD, Nidhi T, Nitish I, Sankriti M, Nagaveni S, Shweta S.	Postoperative assessment of diode laser zinc oxide eugenol and mineral trioxide aggregate pulpotomy procedures in children: A comparative clinical study	2018	J Indian Soc Pedod Prev Dent	Primary theeth
Rahman B.; Goswami M.	Comparative Evaluation of Indirect Pulp Therapy in Young Permanent Teeth using Biodentine and Theracal: A Randomized Clinical Trial	2021	Journal of Clinical Pediatric Dentistry	Not specifically about partial pulpotomy
Reetu S.; Shrestha D.; Kayastha R.	Post-operative pain and associated factors in patients undergoing single visit root canal treatment on teeth with vital pulp	2018	Kathmandu University Medical Journal	Not specifically about partial pulpotomy
Schwendicke F., Stolpe M.	Direct pulp capping after a carious exposure versus root canal treatment: a cost-effectiveness analysis	2014	Journal of endodontics	Not specifically about partial pulpotomy
Sharma A.; Thomas M.; Shetty N.; Srikant N.	Evaluation of indirect pulp capping using pozzolan-based cement (ENDOCEM-Zr®) and mineral trioxide aggregate - A randomized controlled trial	2020	Journal of Conservative Dentistry	Not specifically about partial pulpotomy
Singh S., Mittal S., Tewari S.	Effect of Different Liners on Pulpal Outcome after Partial Caries Removal: A Preliminary 12 Months Randomised Controlled Trial	2019	Caries research	Not specifically about partial pulpotomy
Taha N.A.; Abdelkhader S.Z.	Outcome of full pulpotomy using Biodentine in adult patients with symptoms indicative of irreversible pulpitis	2018	International Endodontic Journal	Not specifically about partial pulpotomy
Taha N.A.; Al-Rawash M.H.; Imran Z.A.	Outcome of full pulpotomy in mature permanent molars using 3 calcium silicate-based materials: A parallel, double blind, randomized controlled trial	2022	International Endodontic Journal	Not specifically about partial pulpotomy
Taha N.A.; Khazali M.A.	Partial Pulpotomy in Mature Permanent Teeth with Clinical Signs Indicative of Irreversible Pulpitis: A Randomized Clinical Trial	2017	Journal of Endodontics	Not specifically about partial pulpotomy
Torabzadeh H, Asgary S.	Indirect pulp therapy in a symptomatic mature molar using calcium enriched mixture cement	2013	J Conserv Dent	Not specifically about partial pulpotomy

Trairatvorakul C, Koothiratrakarn A.	Calcium hydroxide partial pulpotomy is an alternative to formocresol pulpotomy based on a 3-year randomized trial	2012	Int J Paediatr Dent	Primary theeth
Tsujino K.; Shintani S.	Intentional partial pulpotomy to talon cusp for tooth crown morphology correction in orthodontic treatment	2021	Pediatric Dental Journal	Intentional partial pulpotomy
Tüzüner T., Alacam A., Altunbas D.A., Gokdogan F.G., Gundogdu E.	Clinical and radiographic outcomes of direct pulp capping therapy in primary molar teeth following haemostasis with various antiseptics: a randomised controlled trial.	2012	European journal of paediatric dentistry : official journal of European Academy of Paediatric Dentistry	Not specifically about partial pulpotomy
Vignesh S., Nasim I., Rajasekar A.	A retrospective analysis of the correlation between the type of pulp exposure and the choice of pulp capping agents	2020	International Journal of Research in Pharmaceutical Sciences	Not specifically about partial pulpotomy
Wang J.	Treatment of decayed primary teeth with vital and non-vital pulp conditions	2019	Zhonghua kou qiang yi xue za zhi = Zhonghua kouqiang yixue zazhi = Chinese journal of stomatology	Not specifically about partial pulpotomy
Yazdanfar I, Barekatain M, Zare Jahromi M.	Combination effects of diode laser and resin-modified tricalcium silicate on direct pulp capping treatment of caries exposures in permanent teeth: a randomized clinical trial	2020	Lasers Med Sci	Not specifically about partial pulpotomy
Kang C.-M., Sun Y., Song J.S., Pang N.-S., Roh B.-D., Lee C.-Y., Shin Y.	A randomized controlled trial of various MTA materials for partial pulpotomy in permanent teeth	2017	Journal of dentistry	Wrong population
